# Building Programs to Eradicate Toxoplasmosis Part I: Introduction and Overview

**DOI:** 10.1007/s40124-022-00269-w

**Published:** 2022-08-22

**Authors:** Mariangela Soberón Felín, Kanix Wang, Aliya Moreira, Andrew Grose, Karen Leahy, Ying Zhou, Fatima Alibana Clouser, Maryam Siddiqui, Nicole Leong, Perpetua Goodall, Morgan Michalowski, Mahmoud Ismail, Monica Christmas, Stephen Schrantz, Zuleima Caballero, Ximena Norero, Dora Estripeaut, David Ellis, Catalina Raggi, Catherine Castro, Davina Moossazadeh, Margarita Ramirez, Abhinav Pandey, Kevin Ashi, Samantha Dovgin, Ashtyn Dixon, Xuan Li, Ian Begeman, Sharon Heichman, Joseph Lykins, Delba Villalobos-Cerrud, Lorena Fabrega, José Luis Sanchez Montalvo, Connie Mendivil, Mario R. Quijada, Silvia Fernández-Pirla, Valli de La Guardia, Digna Wong, Mayrene Ladrón de Guevara, Carlos Flores, Jovanna Borace, Anabel García, Natividad Caballero, Claudia Rengifo-Herrera, Maria Theresa Moreno de Saez, Michael Politis, Kristen Wroblewski, Theodore Karrison, Stephanie Ross, Mimansa Dogra, Vishan Dhamsania, Nicholas Graves, Marci Kirchberg, Kopal Mathur, Ashley Aue, Carlos M. Restrepo, Alejandro Llanes, German Guzman, Arturo Rebellon, Kenneth Boyer, Peter Heydemann, A. Gwendolyn Noble, Charles Swisher, Peter Rabiah, Shawn Withers, Teri Hull, Chunlei Su, Michael Blair, Paul Latkany, Ernest Mui, Daniel Vitor Vasconcelos-Santos, Alcibiades Villareal, Ambar Perez, Carlos Andrés Naranjo Galvis, Mónica Vargas Montes, Nestor Ivan Cardona Perez, Morgan Ramirez, Cy Chittenden, Edward Wang, Laura Lorena Garcia-López, Juliana Muñoz-Ortiz, Nicolás Rivera-Valdivia, María Cristina Bohorquez-Granados, Gabriela Castaño de-la-Torre, Guillermo Padrieu, Juan David Valencia Hernandez, Daniel Celis-Giraldo, Juan Alejandro Acosta Dávila, Elizabeth Torres, Manuela Mejia Oquendo, José Y. Arteaga-Rivera, Dan L. Nicolae, Andrey Rzhetsky, Nancy Roizen, Eileen Stillwaggon, Larry Sawers, Francois Peyron, Martine Wallon, Emanuelle Chapey, Pauline Levigne, Carmen Charter, Migdalia De Frias, Jose Montoya, Cindy Press, Raymund Ramirez, Despina Contopoulos-Ioannidis, Yvonne Maldonado, Oliver Liesenfeld, Carlos Gomez, Kelsey Wheeler, Ellen Holfels, David Frim, David McLone, Richard Penn, William Cohen, Samantha Zehar, James McAuley, Denis Limonne, Sandrine Houze, Sylvie Abraham, Raphael Piarroux, Vera Tesic, Kathleen Beavis, Ana Abeleda, Mari Sautter, Bouchra El Mansouri, Adlaoui El Bachir, Fatima Amarir, Kamal El Bissati, Alejandra de-la-Torre, Gabrielle Britton, Jorge Motta, Eduardo Ortega-Barria, Isabel Luz Romero, Paul Meier, Michael Grigg, Jorge Gómez-Marín, Jagannatha Rao Kosagisharaf, Xavier Sáez Llorens, Osvaldo Reyes, Rima McLeod

**Affiliations:** 1 Toxoplasmosis Programs and Initiatives in Panamá, Ciudad de Panamá, Panamá; 2 Institute for Genomics and Systems Biology, The University of Chicago, Chicago, IL USA; 3 Pritzker School of Medicine, The University of Chicago, Chicago, IL USA; 4 Instituto de Investigaciones Científicas y Servicios de Alta Tecnología AIP (INDICASAT-AIP), Ciudad de Panamá, Panamá; 5 Department of Pediatrics Infectious Diseases/Department of Neonatology, Hospital del Niño doctor José Renán Esquivel, Ciudad de Panamá, Panamá; 6 Department of Ophthalmology and Visual Sciences, The University of Chicago, Chicago, IL USA; 7 The College, The University of Chicago, Chicago, IL USA; 8 The Global Health Center, The University of Chicago, Chicago, IL USA; 9 Department of Statistics, The University of Chicago, Chicago, IL USA; 10 Rush University Medical School/Rush University Medical Center, Chicago, IL USA; 11 Academia Interamericana de Panamá, Ciudad de Panamá, Panamá; 12 Hospital Santo Tomás, Ciudad de Panamá, Panamá; 13 Hospital San Miguel Arcángel, Ciudad de Panamá, Panamá; 14 Universidad de Panamá, Ciudad de Panamá, Panamá; 15 Department of Public Health Sciences, The University of Chicago, Chicago, IL USA; 16 Harris School of Public Policy, The University of Chicago, Chicago, IL USA; 17 Sanofi Aventis de Panamá S.A., University of South Florida, Ciudad de Panamá, Panamá; 18 Northwestern University Feinberg School of Medicine, Chicago, IL USA; 19 NorthShore Evanston Hospital, Evanston, IL USA; 20 Department of Microbiology, The University of Tennessee, Knoxville, TN USA; 21 Universidad de Federal de Minas Gerais, Belo Horizonte, Minas Gerais Brazil; 22 Universidad Autónoma de Manizales, Manizales, Colombia; 23 Universidad del Quindío, Armenia, Colombia; 24 Grupo de Investigación en Neurociencias, Universidad del Rosario, Bogotá, Colombia; 25 The University of South Florida College of Public Health, Tampa, FL USA; 26 Department of Economics, Gettysburg College, Gettysburg, PA USA; 27 Department of Economics, American University, Washington, D.C. USA; 28 Institut des agents infectieux, Hôpital de la Croix-Rousse, Lyon, France; 29 Remington Specialty Laboratory, Palo Alto, CA USA; 30 Department of Pediatrics, Division of Infectious Diseases, Stanford University College of Medicine, Stanford, CA USA; 31 Roche Molecular Diagnostics, Pleasanton, CA USA; 32 LDBioDiagnostics, Lyon, France; 33 Laboratory of Parasitologie, Bichat-Claude Bernard Hospital, Paris, France; 34 INH, Rabat, Morocco; 35 Faculty of Sciences Ain Chock, University Hassan II, Casablanca, Morocco; 36 Member of the Sistema Nacional de investigadores de Panamá (SNI), Ciudad de Panamá, Panama; 37 Secretaría Nacional de Ciencia, Tecnología e Innovación (SENACYT), Ciudad de Panamá, Panamá; 38 GSK Vaccines, Panamá, Panamá; 39 Molecular Parasitology, NIAID, NIH, Bethesda, MD USA; 40 Toxoplasmosis Center, The University of Chicago and Toxoplasmosis Research Institute, Chicago, IL USA; 41 Department of Pediatrics, Division of Infectious Diseases, The University of Chicago, Chicago, IL USA

**Keywords:** review, foundational work, toxoplasmosis, congenital toxoplasmosis, Panama, Colombia, United States, France, Brazil, Morocco, medical care, pyrimethamine, sulfadiazine, public health, student research

## Abstract

**Purpose of Review:**

Review building of programs to eliminate *Toxoplasma* infections.

**Recent Findings:**

Morbidity and mortality from toxoplasmosis led to programs in USA, Panama, and Colombia to facilitate understanding, treatment, prevention, and regional resources, incorporating student work.

**Summary:**

Studies foundational for building recent, regional approaches/programs are reviewed. Introduction provides an overview/review of programs in Panamá, the United States, and other countries. High prevalence/risk of exposure led to laws mandating testing in gestation, reporting, and development of broad-based teaching materials about *Toxoplasma.* These were tested for efficacy as learning tools for high-school students, pregnant women, medical students, physicians, scientists, public health officials and general public. Digitized, free, smart phone application effectively taught pregnant women about toxoplasmosis prevention. Perinatal infection care programs, identifying true regional risk factors, and point-of-care gestational screening facilitate prevention and care. When implemented fully across all demographics, such programs present opportunities to save lives, sight, and cognition with considerable spillover benefits for individuals and societies.

**Supplementary Information:**

The online version contains supplementary material available at 10.1007/s40124-022-00269-w.

## Introduction

**Fig. 1:**
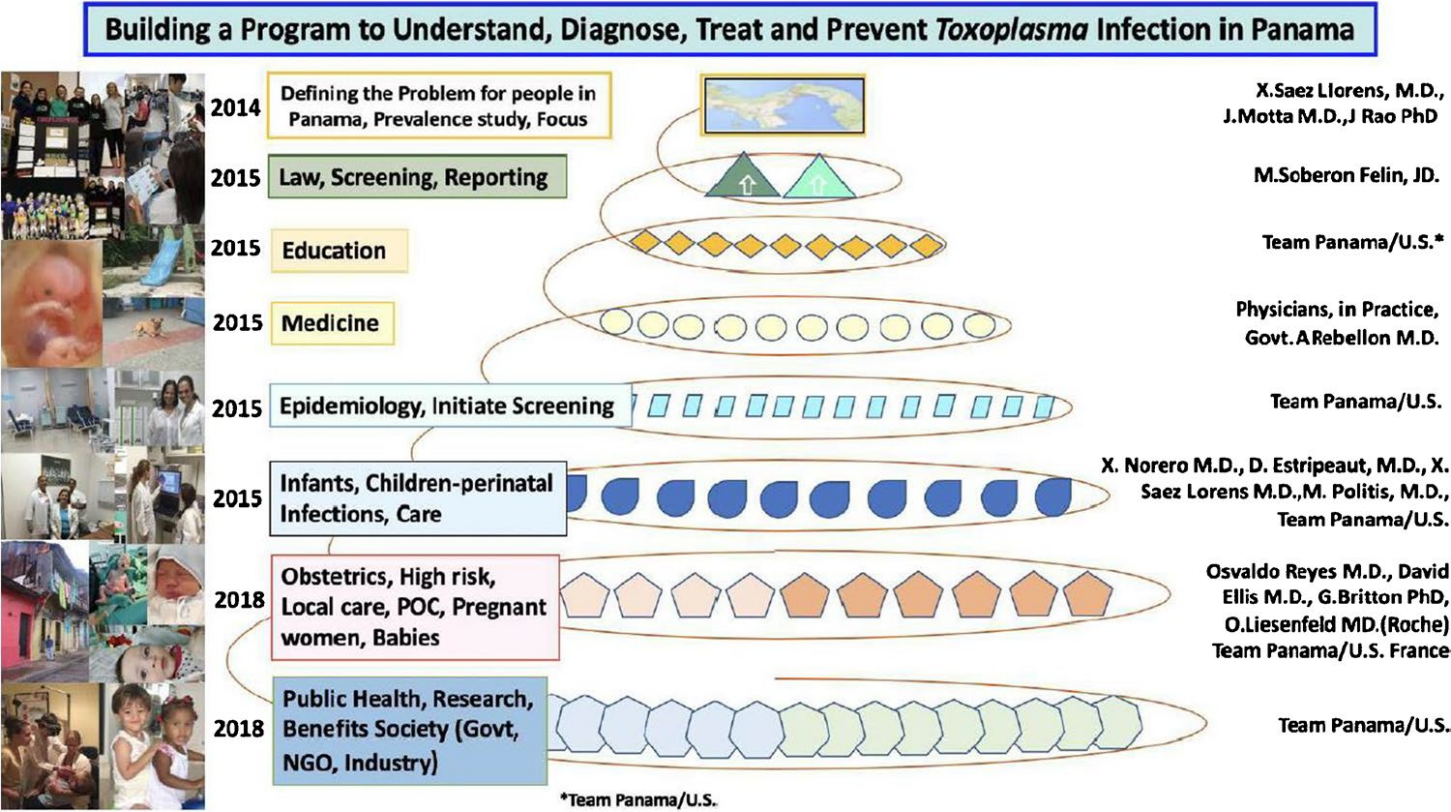
Timeline of the projects and areas of research in which “Team Panama” has participated since 2014

**Fig. 2:**
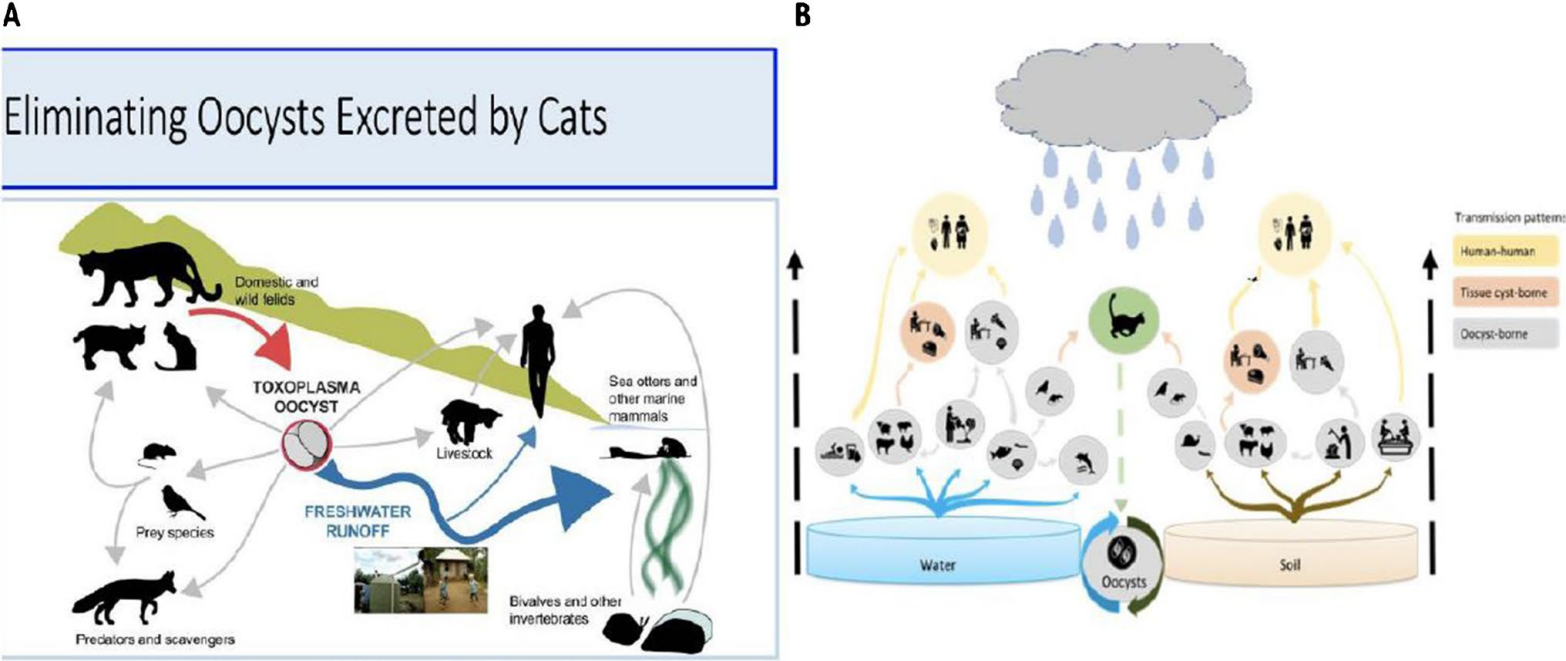
A. Model of *Toxoplasma gondii* transmission between domestic and wild felids, freshwater runoff, livestock, predatory and scavenger animals, marine mammals, bivalves and other invertebrates, and humans. This model is subject to change depending on the setting, but it presents a general overview of how *Toxoplasma* circulates through an ecosystem and suggests ways in which transmission to humans can be stopped or limited. Adapted from Van Wormer et al. B. Relationship of *Toxoplasma* transmission patterns to water and soil; designed by Jorge Gómez-Marin and Lilian Bahia Oliveira, also in McLeod et al. 2022.

Herein, we present an introduction to, and an overview and review of work [1••, 2, 3••, 4••, 5••, 6••, 7••, 8••, 9••, 10••, 11••, 12, 13, 14••, 15••, 16••, 17••, 18•, 19•, 20•, 21•, 22•, 23, 24••, 25, 26, 27•, 28•, 29, 30, 31•, 32•, 33••, 34•, 35•, 36, 37•, 38•, 39••, 40•, 41•, 42••, 43••, 44••, 45, 46•, 47••, 48••, 49, 50•, 51•, 52•, 53••, 54, 55••, 56••, 57•, 58••, 59•, 60••, 61, 62••, 63•, 64••, 65, 66••, 67••, 68••, 69, 70, 71••, 72••, 73••, 74••, 75••, 76–78, 79••, 80••, 81••, 82, 83, 84••, 85••, 86••, 87–98, 99••, 100••, 101–119, 120••, 121••, 122••, 123••, 124••, 125••, 126••, 127••, 128••, 129••, 130, 131, 132••, 133••, 134–137, 138••, 139, 140••, 141, 142••, 143–151] to provide a basis for public health programs addressing toxoplasmosis. Students began by reviewing pertinent, available literature which is summarized in part in Tables 1, 2, 3 and references [1••, 2, 3••, 4••, 5••, 6••, 7••, 8••, 9••, 10••, 11••, 12, 13, 14••, 15••, 16••, 17••, 18•, 19•, 20•, 21•, 22•, 23, 24••, 25, 26, 27•, 28•, 29, 30, 31•, 32•, 33••, 34•, 35•, 36, 37•, 38•, 39••, 40•, 41•, 42••, 43••, 44••, 45, 46•, 47••, 48••, 49, 50•, 51•, 52•, 53••, 54, 55••, 56••, 57•, 58••, 59•, 60••, 61, 62••, 63•, 64••, 65, 66••, 67••, 68••, 69, 70, 71••, 72••, 73••, 74••, 75••, 76–78, 79••, 80••, 81••, 82, 83, 84••, 85••, 86••, 87–98, 99••, 100••, 101–119, 120••, 121••, 122••, 123••, 124••, 125••, 126••, 127••, 128••, 129••, 130, 131, 132••, 133••, 134–137, 138••, 139, 140••, 141, 142••, 143–151] with commentary about each manuscript for this Review. This initially was focused, as reviewed here, on work in France, Austria, the USA, Brazil, and Colombia (Fig. 1). Student-generated research then contributed to this initiative, which encompassed three different countries including Panama, Colombia and the USA, seven different educational levels (high school, university, medical school, residency, graduate school, post-graduate, practicing physicians and scientists), patients of all educational backgrounds, and more than a dozen institutions. Each individual research project generated useful data and discussions at poster sessions and presentations that utilized and then updated our body of research. In addition, each student’s work provided original information concerning a certain aspect of toxoplasmosis and its prevention and treatment, which could then be expanded upon by other groups in the USA, Panama, Colombia, and other countries (e.g., Brazil, France, Morocco). For example, there were six groups of US students and additional in-country students, scientists, and practitioners who, over the course of eight years, devised and updated spatial epidemiologic analyses of *Toxoplasma* seroprevalence and risk factors in Panama and Colombia. Some of this work provided a foundation for and part was incorporated into now published, relevant work [41•, 42••, 43••, 44••, 45, 46•, 47••, 48••, 49, 50•, 52•, 53••, 54, 55••, 56••, 57•, 59•, 62••, 63•, 65]. As a fundamental part of our global initiative, students have established new routes of communication between multiple academic and medical institutions. Together, we have created a tradition of, and paradigm for, research contributions that can be passed down and modified by new generations who take an interest in our expanding toxoplasmosis initiative. This introduction is the first in a series of four papers, which describe work begun in Panama in 2014 to build a comprehensive public health program (Fig. 1). This initiative is structured to provide education, information, and improvements meant to benefit healthcare centered around eliminating toxoplasmosis. The importance of exposure to oocyst contaminated water sources and soil became apparent from this and other work (Fig. 2) [57•, 62••, 63•, 64••, 65, 77, 99••].

“Toxoplasmosis” refers to the disease caused by the protozoan parasite *Toxoplasma gondii* [138••]. The parasite can be acquired through a variety of pathways; while a common way of acquisition is ingestion of tissue cysts found in raw or undercooked meat, Figure 2 demonstrates how oocysts can also be transmitted via feces from cats, which are *T. gondii*’s natural host, as well as through water and soil cycles [64••, 138••].

### Toxoplasma

is acquired congenitally when a pregnant woman contracts the parasite for the first time, which is marked by seroconversion including IgM and IgG antibodies. About 8 weeks after infection, serum IgM antibody levels usually decrease, while IgG levels become high and stable, as measured by the Sabin Feldman dye test. This pattern of test results signals subacute/chronic infection, indicating that the individual is now partially immune to subsequent infection. With established maternal seropositivity prior to pregnancy, the fetus usually is not at risk for congenital toxoplasmosis (CT). IgG antibodies persist for life, so their presence in serum is a marker of either acute or chronic infection with the parasite. Meanwhile, IgM antibodies might signify a case of recent infection, in which parasite tachyzoites may be vertically transmitted to the fetus. This can result in ocular and neurologic damage—such as chorioretinitis, loss of sight, psychomotor impediments, seizures, microcephaly, hydrocephalus, and intracranial calcifications, among other manifestations—as well as prematurity and pregnancy loss [71••, 79••, 137]. As multiple studies have observed, the best way to prevent these adverse effects is through prompt diagnosis and treatment of acutely infected mothers; this can greatly decrease risk of mother-to-child transmission and overall damage the parasite can do if it manages to cross the placenta [4••, 6••, 14••, 71••].

The devastating effects of toxoplasmosis—especially the congenital form—are well documented. However, despite decades of estimates of disease burden—such as a 2013 World Health Organization report on CT—the actual prevalence of toxoplasmosis is generally not well defined, even in countries with some of the highest estimated rates of infection [141]. The starting point for the present study described herein is Panama, one of many tropical and sub-tropical countries where high prevalence and severe overall disease burden have been noted. While there are few current published results on the seroprevalence of *T. gondii* in Panama, a previous study estimated that this country has one of the highest rates of *Toxoplasma* infection in Latin America, with a seroprevalence of 50% in 10-year-olds and 90% in 60-year-olds [142••]. Additionally, the neighboring countries of Colombia and Costa Rica have estimated seroprevalences of 43–67% and 49-61%, respectively [143–147]. When it comes to the congenital form of toxoplasmosis in Panama, estimated annual incidence is 1.8 cases per 1000 live births, and the estimated number of disability-adjusted life-years (DALYs), i.e., loss of the equivalent of one year of full health, resulting from congenital infection is 840 [141]. With this high seroprevalence and significant burden, Panama would be expected to have a significant risk for the development of congenital and ocular toxoplasmosis. Toxoplasmosis has traditionally been a neglected disease in Panama, despite significant morbidity and mortality. Given this need, a team of scientists, physicians, and students from Panama, Colombia, and the USA —known as “Team Panama” —made an effort to establish a healthcare program in Panama that could reduce the adverse impacts of this infection. In this paper, we describe the multiple facets of our public health project: building educational programs and understanding regional prevalence of toxoplasmosis, evaluating the efficacy of a mandate to screen for and report CT, studying risk factors for toxoplasmosis, building spatial epidemiology maps, creating mathematical models to predict risk, and improving availability of care for symptomatic illness, among other items. We also describe some of our parallel projects in neighboring Colombia, a country that has a more robust mandatory gestational screening program for CT, and in the United States, where a large part of our research is based. We conclude with an evaluation of our research and initiatives and comments on the next steps to take to improve care for toxoplasmosis (especially the congenital form) in all three countries.

## Review of Foundational Studies and Updates Concerning Clinical Manifestations of Congenitally Infected Children and Treatment in Programs in France, Austria, the USA, Brazil, Colombia and Morocco

### *France, Austria*

The important work from France [46•, 51•, 52•, 56••, 60••, 62••, 66••, 67••] and Austria [53••] with some data from the USA [1••, 2, 3••, 4••, 5••, 6••, 7••, 8••, 9••, 10••, 11••, 12, 13, 14••, 15••, 16••, 17••, 18•, 19•, 20•, 21•, 22•, 23, 24••, 25, 26, 27•, 28•, 29, 30, 31•, 32•, 33••, 34•, 35•, 36, 37•, 38•, 39••, 40•, 41•, 42••, 43••, 44••, 45, 46•, 47••, 48••, 49, 50•, 51•, 52•, 53••, 54, 55••, 56••, 57•, 58••, 59•] has demonstrated that congenital toxoplasmosis is a treatable and preventable disease. Like other infectious diseases the sooner treatment is initiated the better the outcome.

**Fig. 3:**
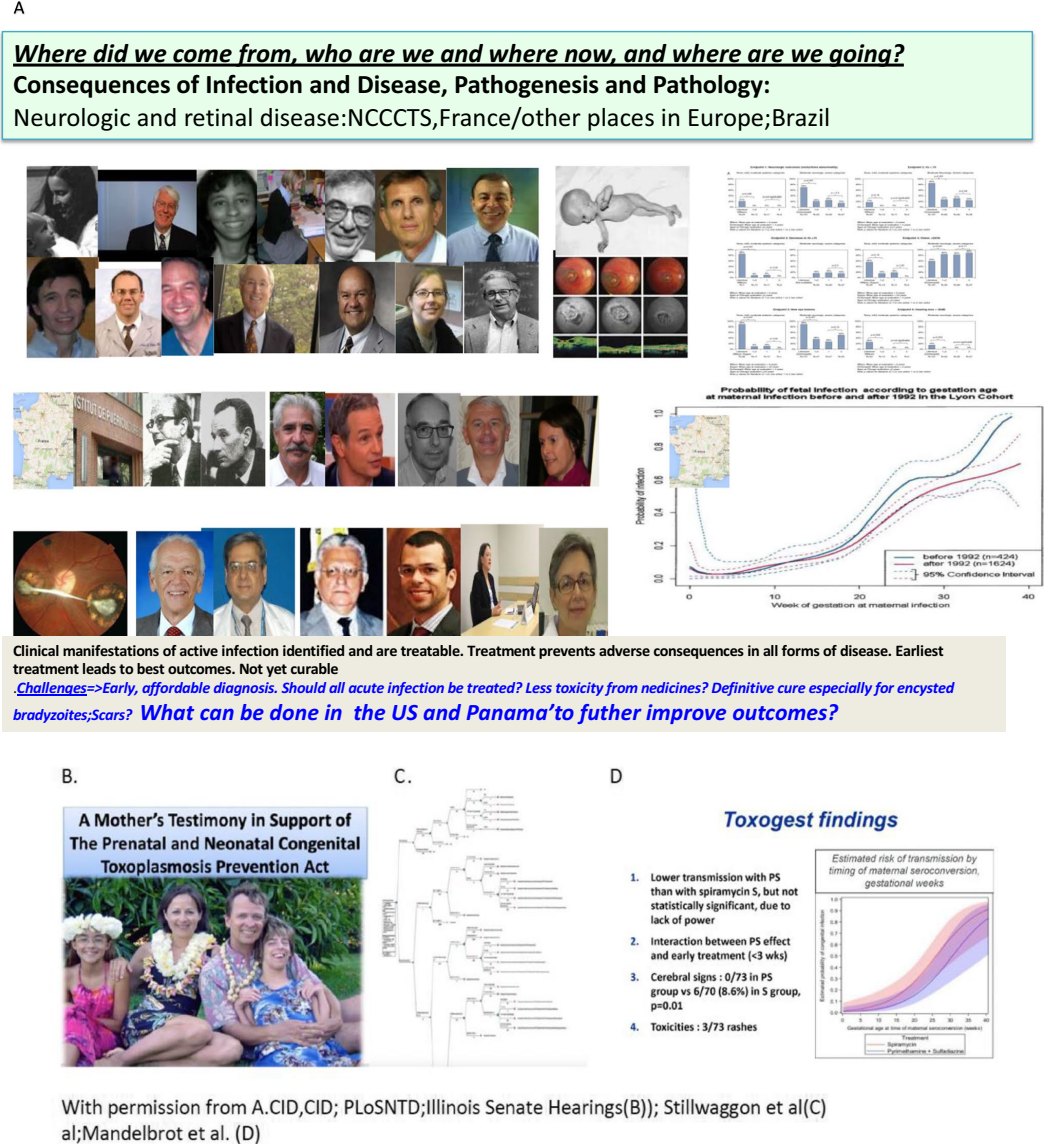
Early studies in France, the United States, and Brazil demonstrated efficacy of treatment and shaped understanding of this disease. The images from France, those in the NCCCTS in the USA, and in Brazil represent the early chronology of this work, and show some of the investigators who worked on some of these studies. Special accomplishments of colleagues are noteworthy and have not been mentioned specifically. Since a number of those working on this study in the early years have died in the past two years with some posthumous authors they are mentioned specifically as follows: Paul Meier designed the first Toxoplasma RCT for congenital toxoplasmosis with colleagues in the NCCCTS. Michael Gottlieb’s guidance as a Program officer at NIAID was instrumental in the establishment of the NCCCTS and many aspects of development of treatments and vaccines to prevent this disease along with others continuing in this in the NIAID DMID program. Jean Hickman worked with them within NIAID. Eileen Stillwaggon worked on cost benefit analyses demonstrating predicted cost savings when there was diagnosis and treatment of congenital toxoplasmosis in the United States. In Austria she found fourteen-fold cost savings occurred. In France, early treatment in gestation has been found to be superior to treatment started after delays (Wallon, Stillwaggon, Sawyer et al 2022, In submission). Charles N Swisher was one of the primary neurologists in the NCCCTS working with this study from 1981 until his passing in 2020. Jack S Remington and his laboratory performed the serologic tests for this study and included findings from the NCCCTS in co-authored book chapters, with the work of his laboratory continuing with the study. Lazlo Stein developed the hearing testing protocols. The contributions of others either as authors or acknowledged are substantial as well. Each of these scientists, physicians, and others had an important role in the understanding and improvement in outcomes for this disease.

The cost benefit analyses emphasize that treatment brings not only reduction in individual suffering but also brings substantial cost savings for countries (e.g., 14 fold in Austria [53••]). The major role France has had in developing this improved approach that saves life, sight, and cognition is represented also in Fig. 3. As this has been addressed in earlier publications [46•, 51•, 52•, 53••, 56••, 60••, 62••, 66••, 67••], it is not considered in more depth here. Many of the problems faced in the USA are avoided in France and Austria where prices for medicines are mandated by the government, social systems support medical care evenly for all those in the countries, and prenatal screening and reporting for toxoplasmosis are required by law.

### United States

In the United States, an ongoing longitudinal study called the National Collaborative Chicago-based Congenital Toxoplasmosis Study (NCCCTS) was initiated in 1981 and continues into the present time. Methodology has been presented in ongoing reports throughout that time. Methodology has included obtaining information about this infection, its epidemiology, diagnosis, treatment and outcomes (Tables 1, 2 and 3) [1••, 2, 3••, 4••, 5••, 6••, 7••, 8••, 9••, 10••, 11••, 12, 13, 14••, 15••, 16••, 17••, 18•, 19•, 20•, 21•, 22•, 23, 24••, 25, 26, 27•, 28•, 29, 30, 31•, 32•, 33••, 34•, 35•, 36, 37•, 38•, 39••, 40•, 41•, 42••, 43••, 44••, 45, 46•, 47••, 48••, 49, 50•, 51•, 52•, 53••, 54, 55••, 56••, 57•, 58••, 59•]. Results are compared with earlier studies [70, 71••]. Initially treatment dosing for infants were established in a phase 1 clinical trial, then with feasibility and safety and favorable outcomes identified, a randomized controlled trial compared higher and lower doses. In this context and with participants who missed treatment during the first year of life, all these families were then observed, carefully documenting outcomes across lifetimes. Methodology for treatment beginning in gestation also has been established with and extended by French colleagues making a model paradigm for care and prevention of sequalae by promptly treating the seroconverting pregnant woman and the fetus followed by treatment in the first year of life [33••].

**Table 1:** Chronology of Findings from the National Collaborative Chicago-based Congenital Toxoplasmosis Study, (NCCCTS) Program (1981–2022), with Mention of Precedent US Studies and Accomplishments of Others, Critical for Development of Approaches to Treating and Understanding this Disease

Study First author (date) [reference]	Details	Major Findings
Sabin, 1945 [considered in 4••, 16••, 19•, 20•, 33••]	Child from whom the RH type I strain was isolated.	*Toxoplasma* causes a severe disease in infants; RH strain of *Toxoplasma* was isolated.
Eichenwald, 1959 [considered in 4••, 16••, 19•, 20•, 33••;88]	Infants who presented with generalized or neurologic disease at birth had severe sequalae by four years of age when either untreated or treated for one month at birth.	Congenital toxoplasmosis presents with generalized or neurologic manifestations or both. This usually results in significant harmful effects on brain and eyes by the age of 4 years when untreated or treated only for one month.
Wilson, Remington, Stagno, Reynolds, 1980 [considered in 4••, 16••, 19•, 20•, 33••;90]	Infants who appeared to be asymptomatic or have mild involvement after birth, when untreated, almost all had significant retinal disease and fall in IQ by the time they were teenagers.	Late manifestations of congenital *Toxoplasma* infection occurred in the eye and brain by teenage years even in children who were asymptomatic at birth.
Mcleod R, 1990 [1••]	Infant with congenital toxoplasmosis had lymphocytes unresponsive to *Toxoplasma* antigens.	Selective unresponsiveness of a congenitally infected infant to *Toxoplasma* antigens.
McGee T, 1992 [2]	Absence of sensorineural hearing loss in treated infants and children with congenital toxoplasmosis.	Sensorineural hearing loss is very rare in the USA in humans with congenital toxoplasmosis.
Mcleod R, 1992 [3••]	Levels of pyrimethamine in sera and cerebrospinal and ventricular fluids from infants treated for congenital toxoplasmosis. It is a phase 1 clinical trial of treatment of congenital toxoplasmosis and finds therapeutic levels of pyrimethamine and safety with one year of treatment.	Congenital toxoplasmosis can be treated with pyrimethamine and sulfadiazine in infancy for one year safely, demonstrated in a phase 1 clinical trial. Two children received a higher dose than planned which formed the bases for a phase 2 clinical trial. Potentially therapeutic serum levels of pyrimethamine in the infants defined.
McAuley J, 1994 [4••]	Early and longitudinal evaluations of treated infants and children and untreated historical patients with congenital toxoplasmosis.	Favorable outcomes of treated congenital toxoplasmosis identified, manifestations and natural history described, Treated congenital toxoplasmosis can have favorable outcomes.
Swisher CN, 1994 [5••]	Describes neurologic findings of congenital toxoplasmosis in the USA.	This describes good outcomes even with significant neurologic disease and some prognostic factors associated with less favorable outcomes.
Roizen N, 1995 [6••]	Neurologic and developmental outcome in treated congenital toxoplasmosis. This demonstrates better neurologic outcomes and the importance of recognizing that what may be interpreted as developmental delays are really related to visual impairment. This emphasizes the importance of compensatory educational strategies.	Visual impairment can be misinterpreted as developmental delays, showing a necessity for compensatory educational strategies to overcome visual impairments.
Patel DV, 1996 [7••]	Resolution of intracranial calcifications in infants with treated congenital toxoplasmosis.	Calcifications often diminish in size or resolve during one year of treatment in infancy.
Vogel N, 1996 [8••]	Congenital toxoplasmosis transmitted from an immunologically competent mother infected before conception.	Very rarely a mother infected before conception can transmit to the fetus during gestation.
Mets MB, 1996 [9••]	Eye manifestations of congenital toxoplasmosis.	Large ocular scars can occur with normal or near normal vision. Treatment with pyrimethamine and sulfadiazine results in prompt resolution of active eye disease in infants and children. Eye lesions can occur later in treated children and more commonly in those who miss treatment *in utero* and in the first year of life.
Roberts F, 1999 [10••]	Pathogenesis of toxoplasmic retinochoroiditis secondary to toxoplasmosis. Pathology in fetal/infant eyes described.	Pathology involves destruction of retinal tissue and inflammation with some parasites present in the eye.
Mack DG, 1999 [11••]	HLA-class II genes modify outcome of *Toxoplasma gondii* infection.	HLADQ3 susceptibility, DQ1 protective allele in HLA transgenic mice. Pathology: cysts, perivascular cuffing, leptomeningeal inflammation and microglial nodules in brain parenchyma. In NCCCTS, HLADQ3 associated with development of hydrocephalus.
Brezin AP, 2003 [12]	Ophthalmic outcomes after prenatal and postnatal treatment of congenital toxoplasmosis. Description of retinal lesions in children in France by NCCCTS and french ophthalmologists.	Characterization of eye lesions and toxoplasmosis later in life.
Boyer KM, 2005 [13••]	Risk factors for *Toxoplasma gondii* infection in mothers of infants with congenital toxoplasmosis. Implications for prenatal management and screening.	There is some association with contact with cat excrement or eating meat that is not well cooked. But the majority of mothers had no recognized risk factors, suggesting unrecognized environmental contamination. Gestational screening for pregnant women and vaccines likely needed to eliminate the disease because much exposure is unrecognized.
McLeod R, 2006 [14••]	Severe sulfadiazine hypersensitivity in a child with reactivated congenital toxoplasmic chorioretinitis.	Dress syndrome (rash, eosinophilia, monocytosis, and a fever) occurred in a child in association with sulfadiazine. This can occur in treated children and is lethal 10% of the time.
Roizen N, 2006 [15••]	Visual impairment impacts measures of cognitive function in children with congenital toxoplasmosis, showing a need for compensatory intervention strategies.	Addressing visual impairment is critical for determining the meaning of cognitive test performance and designing compensatory strategies for visual impairments.
McLeod R, 2006 [16••]	Outcome of congenital toxoplasmosis treatment seen in the NCCCTS. Improved outcomes in a phase 1 and 2 clinical trial with treatment with five prespecified endpoints. Best outcomes with prenatal treatment.	Benefit of prenatal and postnatal treatment in randomized control trial with randomized doses of pyrimethamine with participants randomized to a lower or higher dose of pyrimethamine. Doses were chosen to have levels that were therapeutic for equipoise. Treatment can result in favorable outcomes with no substantial irreversible toxicity, transient lower neutrophil counts during treatment. Treatment is safe, long-term longitudinal follow-up is on-going, without later toxicities noted.
Arun V, 2007 [17••]	Treatment and types of cataracts in congenital toxoplasmosis.	Patterns of cataracts in infants with toxoplasmosis and successful surgical treatment.
Benevento JD, 2008 [18•]	Treatment with ranibizumab and antiparasitic therapy for toxoplasmosis-associated neovascular lesions.	CNVM due to toxoplasmosis are successfully treated by medicines plus antibody to VEGF.
Phan L, 2008 [19•]	Longitudinal study of new eye lesions in treated congenital toxoplasmosis.	There is some recurrence of disease. The recurrence rate is much less than in untreated children. It is a relapsing progressive disease. The peaks of age for recurrence are around 6-7 years, adolescence, and stressful times.
Phan L, 2008 [20•]	Longitudinal study of new eye lesions in children with toxoplasmosis who were not treated during the first year of life.	Occurrence of new eye lesion is much higher in the children who were not treated.
Jamieson SE, 2008 [21•]	Genetic and epigenetic factors at COL2A1 and ABCA4 influence development of congenital toxoplasmosis.	ABC4 and COL2A are susceptibility genes for toxoplasmosis. Parent of origin effect demonstrates effect of imprinting.
Jamieson SE, 2009 [22•]	Genetic and epigenetic factors and susceptibility genes in toxoplasmosis.	Overview of genetic susceptibility and epigenetic mechanisms for severity and end organ damage to brain and eye in congenital toxoplasmosis.
Tan T, 2010 [23••]	Identification of protective *T. gondii* epitopes, adjuvants, and host genetic factors in both mice and humans.	Part of a multistep program to create a vaccine to prevent toxoplasmosis in humans.
Lees MP, 2010 [24••]	P2X7 receptor-mediated killing of an intracellular parasite, *Toxoplasma gondii.*	P2X7R is a susceptibility gene for congenital toxoplasmosis and affects cytokine production. Demonstration with monocytes in culture and in mice.
Jamieson SE, 2010 [25]	Associations between the purinergic receptor P2X(7) (P2RX7) and toxoplasmosis.	P2X7R is a susceptibility gene for congenital toxoplasmosis and affects cytokine production. This is a study of two cohorts demonstrating susceptibility gene through Transmission Disequilibrium Testing (TDT) in the USA and a case control study in Europe.
Noble G, 2010 [26••]	Mothers of children with congenital toxoplasmosis that have chorioretinal lesions in the NCCCTS.	10% of mothers had eye lesions in the NCCCTS. Family members should be evaluated.
McLeod R, 2010 [27•]	Regarding: Prenatal and Neonatal Congenital Toxoplasmosis Prevention and Treatment Act, SB3667 in the context of the National Collaborative Chicago Based Congenital Toxoplasmosis Study.	Presentation to Illinois Senate emphasizing the importance of education and screening to prevent congenital toxoplasmosis in the USA.
Witola WH, 2011 [28•]	NALP1 and its influence on susceptibility, proinflammatory cytokine response, and fate of *Toxoplasma gondii*-infected monocytic cells.	NALP1 inflammasome encodes a susceptibility gene for *Toxoplasma*. Its effector function is through cytokines through cell death during infection with *Toxoplasma*.
Delair E 2011 [29]	Overview of important eye manifestations of toxoplasmosis in USA and France.	Eye manifestations of *Toxoplasma* in USA and France.
Hill D, 2011 [30]	Identification of antigen in oocysts that trigger antibody response in Toxoplasmosis.	Antibody to sporozoites identified in sera.
Stillwaggon E, 2011 [31•]	Maternal serologic screening to prevent congenital toxoplasmosis: a decision-analytic economic model.	Parameters for cost efficacy of pre-natal treatment defined. Applied to USA.
Boyer K, 2011 [32•]	Unrecognized ingestion of *Toxoplasma gondii* oocysts leads to congenital toxoplasmosis and causes epidemics in North America.	Epidemics of *Toxoplasma* in the USA due to oocysts identified. Most infections in mothers of infants with congenital toxoplasmosis in North America were due to unrecognized ingestion of oocysts, including in the Victoria epidemic.
Olario R, 2011	Severe congenital toxoplasmosis in the USA: clinical and serologic findings in untreated infants.	Severe toxoplasmosis in the USA including the NCCCTS patients and others in the Remington Serology laboratory.
McLeod R, 2012 [33••]	Prematurity and severity Associated with *T. gondii* alleles.	Describes genetic serotypes of infection in the USA and shows that certain serotypes are associated with less favorable initial manifestations. However, in treated infants later outcomes are not associated with serotype indicating that all serotypes of infections can be treated effectively.
Lai BS, 2012 [34•]	Molecular target validation, antimicrobial delivery, and potential treatment of *Toxoplasma gondii* infections.	Anti-sense treatment of validated molecular targets demonstrates a way toward a novel approach to treatment.
Burrowes D, 2012 [35•]	Spinal cord lesions in congenital toxoplasmosis demonstrated with neuroimaging, including their successful treatment in an adult.	Chronic *Toxoplasma* can lead to lesions that are symptomatic suggesting that there is chronic, active disease in people.
Bela SR, 2012 [36]	Impaired innate immunity in mice deficient in Interleukin-1 Receptor-Associated Kinase 4 leads to defective type 1 T cell responses, B cell expansion, and enhanced susceptibility to infection with *Toxoplasma gondii.*	IRAK4 is a susceptibility gene for toxoplasmosis.
Witola W, 2014 [37•]	Susceptibility genes and their mechanisms in congenital toxoplasmosis.	ALOX12 is a susceptibility gene for human toxoplasmosis.
Lago et al(2014) Comment by McLeod R, 2014 [38•]	Utility and limitations of *T. gondii*-specific IgM serum antibodies in the diagnosis of congenital toxoplasmosis in Porto Alegre.	Many infants with congenital toxoplasmosis have negative anti-*Toxoplasma* IgM at birth. When present in the neonate, IgM usually becomes negative very early.
McLeod R, 2014 [39••]	Management of congenital toxoplasmosis.	Guidelines for management of toxoplasmosis and its treatment.
Hutson SL, 2015 [40•]	Patterns of hydrocephalus caused by congenital *Toxoplasma gondii* infection and association with parasite strain.	Hydrocephalus in CT may be due to obstruction of aqueduct and/or foramen of monroe or ventricular wall abnormalities with normal pressure. Type II parasites are more often associated with aqueductal obstruction. This is not an all or none association, but suggests that certain parasite alleles may be responsible for these patterns.
Contopoulos- Ioannidis D, 2015 [41•]	Clustering of *Toxoplasma gondii* infections within families of congenitally infected infants.	*Toxoplasma* occurs in families and in those living in residential clusters.
McLeod R, 2016 [42••]	Genetic type of parasite in reference [40•] led to question whether change in outcomes could be due to something other than treatment, but appears to be due to treatment.	Genetic types of parasites associate with particular patterns of hydrocephalous. Type II with aqueductal obstruction, non II with obstruction of foramen of monroe and ventricular wall abnormalities with normal pressure, but does not explain favorable response to treatment.
Lykins J, 2016 [43••]	Understanding Toxoplasmosis in the USA through “Large Data” Analyses.	Based on millions of medical records regarding areas of the country where toxoplasmosis manifests and what medications are utilized. Associations with illness.
Begeman IJ, 2017 [44••]	Point-of-care testing for *Toxoplasma gondii* IgG/IgM using *Toxoplasma* ICT IgG-IgM test with sera from the USA and implications for developing countries.	This is a 100% sensitive and specific test for all types of parasites in the USA from the NCCCTS for every mother-baby dyad where congenital toxoplasmosis was present in the baby. If adapted in areas of high prevalence, and regular obstetrical care for other medical problems, there would be immense spillover benefit.
Peyron, 2017 [45]	Congenital toxoplasmosis in France and the USA: one parasite, two diverging approaches.	Emphasizes ongoing screening for all pregnant women by law in France and benefits for patients.
Ngo HM, 2017 [46•]	*Toxoplasma* modulates signature pathways of human epilepsy, neurodegeneration & cancer.	Influence of *Toxoplasma* on pathways for neurodegeneration, epilepsy, and cancer. This demonstrated biomarkers and suggests there may be significant effects on diseases that are considered chronic diseases of other causes. *Toxoplasma* may be a trigger and progressor of those diseases. Recent meta-analysis studies demonstrate that this may occur. Causal mechanisms are currently under study and biomarkers that overlap with neurodegenerative diseases have been identified.
Del Valle et al, 2021 [47••]	Congenital toxoplasmosis in dizygotic twins with late diagnosis.	Different manifestations in twins diagnosed late with one with suggestion of ongoing disease.
Gomez CA, 2018 [48••]	Evaluation of three point-of-care (POC) tests for detection of *Toxoplasma* immunoglobulin IgG and IgM in the USA: proof of concept and challenges.	The lateral immunochromatography IgG/IgM test LD BiO IgG/IgM Lateral Chromatography test was superior to an Irish and US test.
Lykins J, 2018 [49]	Rapid, inexpensive, fingerstick, whole-blood, sensitive, specific, point-of-care test for anti- *Toxoplasma* antibodies.	Lateral immunochromatographic test is 100% sensitive and specific when blood from fingerstick is used. It makes an inexpensive WHO ASSURED test to rapidly diagnose seroconversion during gestation to eliminate congenital toxoplasmosis with treatment of seroconverting women and in other clinical settings where establishing the diagnosis is critical.
El Bissati K, 2018 [50•]	Global initiative for congenital toxoplasmosis: an observational and international comparative clinical analysis.	Description of ongoing global initiative to eliminate congenital toxoplasmosis through prompt diagnosis and initiation of treatment. Differences between France, the USA, Colombia, and Morocco are emphasized. Example of an effected child with brain and retinal disease is taken from Figure 2 of this paper to emphasize the serious consequences of this infection across all demographics.
Aguirre AA, 2019 [51•]	The one health approach to toxoplasmosis: epidemiology, control, and prevention strategies.	Eliminating *Toxoplasma* will require a number of approaches including focusing on clinical care, on elimination of oocyst excretion from the cat vector, education about food preparation, and water purification.
McLone D, 2019 [52•]	Outcomes of hydrocephalus secondary to congenital toxoplasmosis.	Prompt correction of hydrocephalus by ventriculoperitoneal shunt results in best developmental cognitive and motor outcomes in congenital toxoplasmosis as demonstrated in NCCCTS participants.
El Mansouri B, 2021 [53••]	Performance of a novel point-of-care blood test for *Toxoplasma* infection in women from diverse regions of Morocco.	This demonstrates high functioning of point-of-care test and higher prevalence in a rural, agrarian area in Morocco in association with well water.
Grose A, 2022 [54]	Eliminating confounding false positive IgM tests by using a lateral immunochromatography test that meets WHO ASSURED criteria. In submission.	This improves ability to diagnose seroconversion, eliminating the possibility of false positive IgMs in those without *Toxoplasma* specific IgG.

*There are many additional important studies and findings from France, Austria, Colombia, Brazil, the USA, and elsewhere not listed in this table. These add considerable data to and complement the studies in this table concerning data from the NCCCTS. Especially important is the work of Kieffer, Wallon, Peyron, Desmonts, and Mandelbrot. All of these contribute to the guidelines that we propose that are in the supplement. They are in references 12, 51, 53, 54, and 60. Considerable prognostic, epidemiologic, and mechanistic studies and work contributing to understanding parasite genetic type are emphasized in these references. Newborn screening programs established in Massachusetts and New Hampshire demonstrate successful implementation of such programs at large scale and benefit conferred for children. Bullets indicate papers that have data that are particularly important for treatment..*

**Table 2:** Guidelines from Educational Book Chapters Based on NCCCTS, Toxoplasmosis Research Institute and Center First- Hand Experience and Review of Literature as an Educational Tool, Along with Presentations and Press Releases which Led to News Media Public Service Presentations by Others

	**Clinical Guidelines**	**Diagnosis**	**Medicines**	**Treatment Duration**	**Endpoint of treatment**
Pregnancy	Prompt diagnosis and initiate treatment.	Serologic screening monthly for seronegative individuals; ideally first test before conception; treat seroconversion.	PSL; PAL if hypersensitivity; Spiramycin before first fifteen weeks; amniocentesis at fifteen weeks post-amenorrhea.	Until term; treatment of infected baby to one year of age.	Infant is one year old or has received 12 months of treatment up to 14.5 months of age. Goal is to eliminate or successfully treat active infection in the fetus and newborn infant.
Infancy	Diagnose promptly at birth.	Clinical findings; serology in mother and infant; isolation PCR of placenta as indicated.	PSL per guidelines.	One year.	Elimination of all manifestations if possible or substantially reduce active manifestations.
Recurrent Eye Disease	Diagnose and treat promptly.	Clinical status and serology	PSL per guidelines; PAL if hypersensitivity; for CNVM anti-VEGF is given intraocularly.	Beyond that of active findings; suppression with Azithromycin.	Resolution of activity of eye disease.
Severe Manifestations in Primary Infection	Diagnose and treat promptly.	Clinical status and serology.	PSL per guidelines; PAL if hypersensitivity.	Beyond that of active findings; suppression with Azithromycin.	Resolution of activity and several months beyond.
Immunocompromised Person	Diagnose and treat promptly.	Clinical status and serology.	PSL per guidelines; PAL if hypersensitivity.	Beyond that of active findings; suppression with Azithromycin.	Treatment may be needed to continue until resolution of immunocompromise.

NCCCTS=National Collaborative Congenital Toxoplasmosis Study, P=Pyrimethamine, S=Sulfadiazine, L=Leucovorin, A=Azithromycin, CNVM=Choroidal Neovascular Membrane, VegF=Vascular endothelial growth factor

**Table 3:**
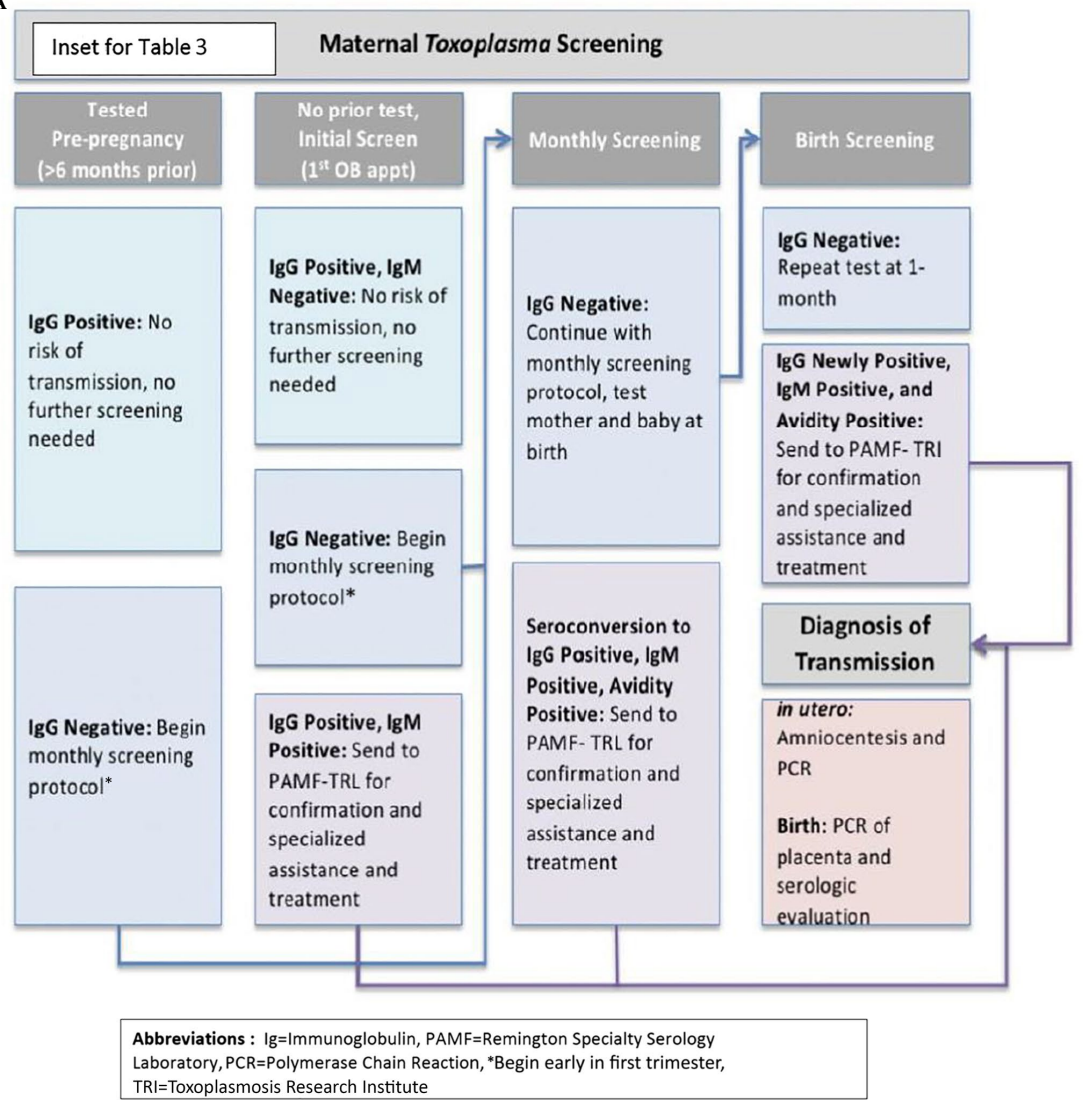

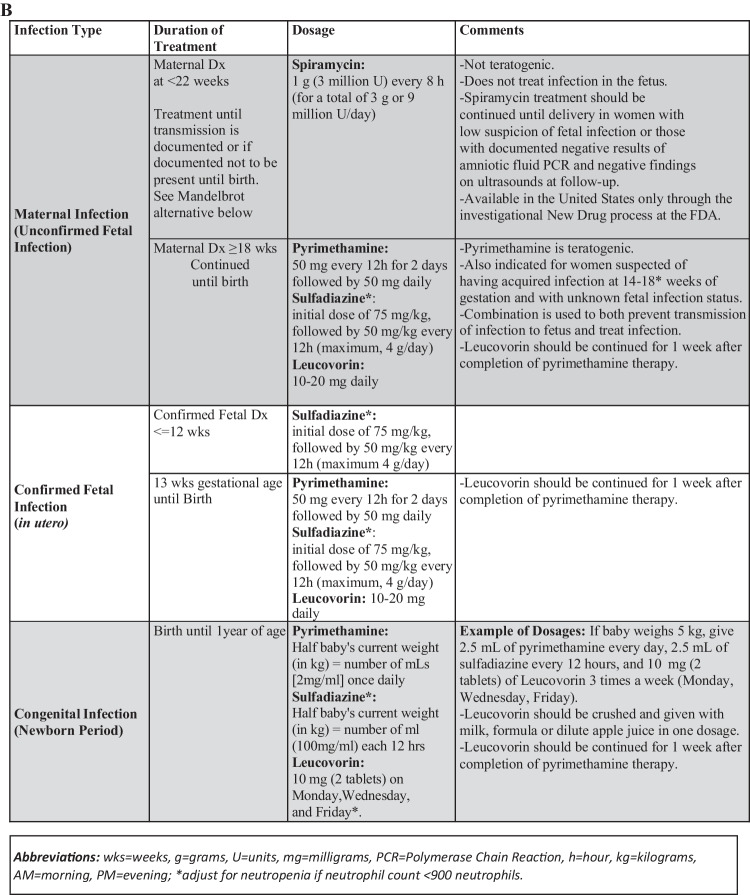
Treatment Guidelines

Y=Yes, N=No, COVID refers to SARS CoVi 2 pandemic

**Fig. 4:**
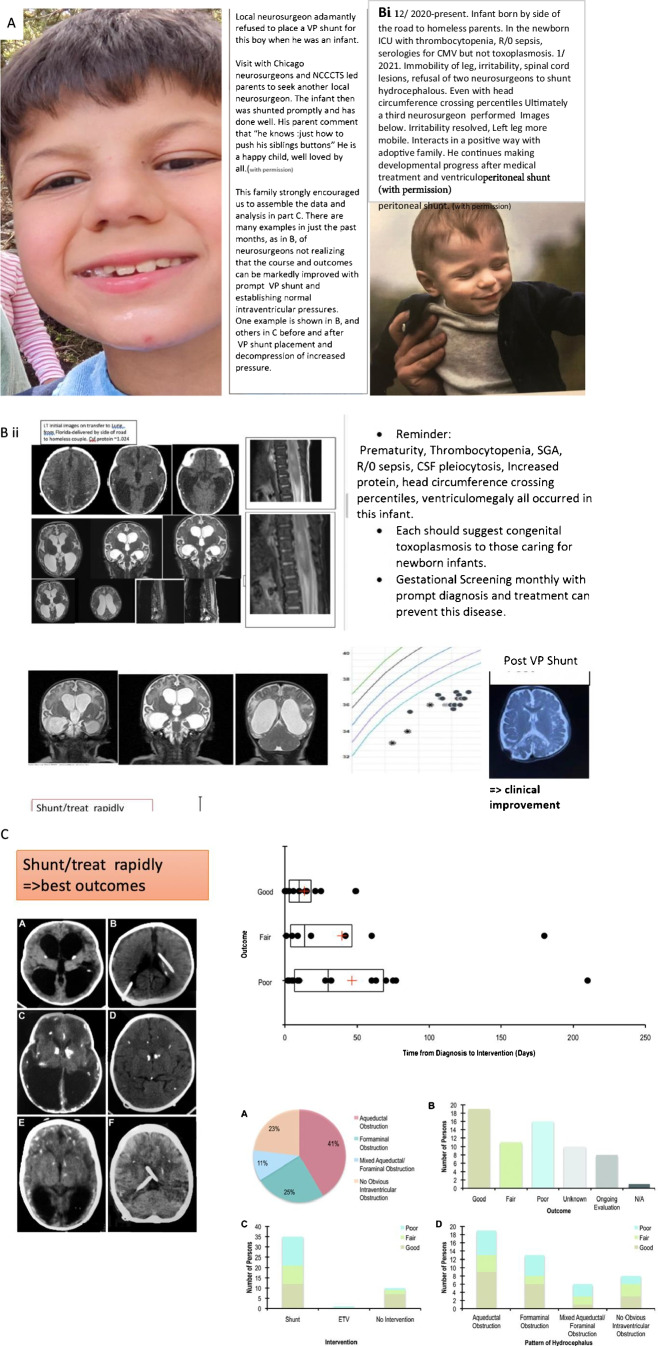
Restoration of anatomy and clinical improvement can occur with prompt treatment and placement of VP shunt. Third ventriculostomies often fail in this infection. A. Shows a recent photo (with permission) of a child whose first neurosurgeon was adamant about not shunting this child when he was an infant. The family sought care elsewhere had a ventriculoperitoneal shunt placed quickly and the child has done well. In just the first months of 2021 this scenario has repeated itself 5 times. Each time, except one infant in Guam, the children were shunted and all improved. B. Another example of an infant with a delay in shunting with a good response shown when an entricular peritoneal shunt was placed. His spinal cord lesions and associated signs are also resolving slowly although with developmental delays. C Figures from McLone et al reproduced with permission show the restoration of anatomy and better outcomes when needed shunts are placed without delay. Each aspect of care for this disease is urgent and emergent and should be treated as expectant for favorable outcome, recognizing this does not always occur but can and does on many occasions. Images reproduced with permission.

**Fig. 5:**
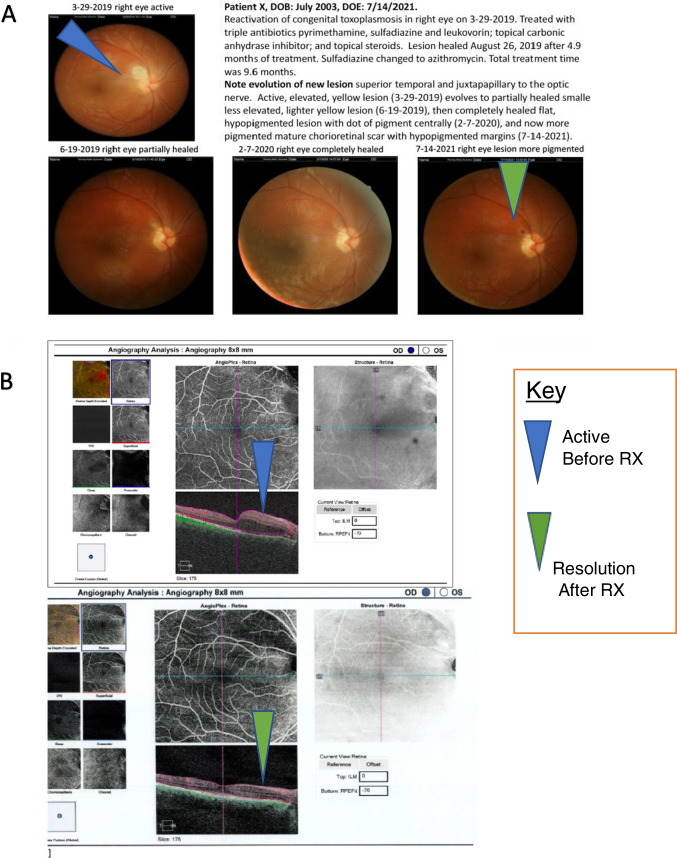
Retinal photographs and Optical Coherence Tomographic studies showing large active lesion with substantial edema (top) and then gradual near complete resolution with treatment with pyrimethamine, sulfadiazine and leucovorin and initially prednisone eye drops. This demonstrates that treatment should be initiated promptly, treatment should continue beyond resolution of the lesion, even large lesions can resolve almost entirely leaving only a small area of pigment, and no permanent detrimental change in visual acuity. An Atlas of retinal lesions is in the Supplemental, and also at toxoplasmosis.org, included herein with permission. The examples in the Atlas show the variability in appearance of lesions of toxoplasmic chorioretinitis.

Results from work in the United States have been published in individual manuscripts [1••, 2, 3••, 4••, 5••, 6••, 7••, 8••, 9••, 10••, 11••, 12, 13, 14••, 15••, 16••, 17••, 18•, 19•, 20•, 21•, 22•, 23, 24••, 25, 26, 27•, 28•, 29, 30, 31•, 32•, 33••, 34•, 35•, 36, 37•, 38•, 39••, 40•, 41•, 42••, 43••, 44••, 45, 46•, 47••, 48••, 49, 50•, 52•, 55••, 56••] but not with a composite overview in some time. This data set is included herein in a not previously published composite form in a table with comments that summarize some of the major earlier and more recently updated findings in the context of others’ work (Tables 1, 2 and 3; Figs. 3**-**5, Part I Supplement) [1••, 2, 3••, 4••, 5••, 6••, 7••, 8••, 9••, 10••, 11••, 12, 13, 14••, 15••, 16••, 17••, 18•, 19•, 20•, 21•, 22•, 23, 24••, 25, 26, 27•, 28•, 29, 30, 31•, 32•, 33••, 34•, 35•, 36, 37•, 38•, 39••, 40•, 41•, 42••, 43••, 44••, 45, 46•, 47••, 48••, 49, 50•, 51•]. Pyrimethamine, sulfadiazine, leucovorin, and spiramycin are essential for the treatment and prevention of congenital toxoplasmosis (CT) [17••, 33••, 46•, 48••, 53••, 60••, 65]. Through ongoing programs for care, the personal experience of the authors informed observations of availability of medicines in Panama and the United States. These observations were made in the context of building programs in each of these countries.

These studies demonstrate that in the United States most diagnoses are made at birth, or later, when significant clinical manifestations have already occurred (e.g., reference 33). Nonetheless, findings which should be sentinel markers of this disease often go unattributed to this infection resulting in unnecessary delays in diagnosis and treatment. As in the child in part B of Fig. 5, and others, prematurity, thrombocytopenia, small for gestational age, rule out sepsis, hepatocellular abnormalities, and meningitis often go undiagnosed as being caused by toxoplasmosis and the diagnosis is missed. This results in needed treatment not being given for months. This has resulted in irreparable damage that could have been prevented.

Prompt prenatal treatment in the United States, as in France and Austria, results in the best outcomes [17••, 33••, 46•, 48••, 53••, 60••, 65]. Outcomes with treatment postnatally also results in markedly better outcomes than earlier without treatment. Those data and analyses include an early phase 1 clinical trial (1981–1990), and later randomized trial (1990–present). Kaplan Meier analyses of the randomized trial with pre-specified endpoints in which a lower and a higher dosage of pyrimethamine were compared. Both treatments were predicted/selected to likely be efficacious based on the Phase 1 clinical study. Doses expected to be effective were utilized for equipoise, with the assumption that if differences from all past experience were found, efficacy would be proven, and perhaps a small dose response effect might be evident. There was not a significant difference in those outcomes for the higher and lower dose, but pre-specified outcomes for both were considerably better than prior studies where children were untreated or treated only for a month [88–90].

In infants who had not been treated *in utero*. there was greater severity at birth and more prematurity in those infected with non II parasite serotypes. In contrast, for infants who were treated *in utero* those differences associated with parasite strain (genetics) were not present at birth [33••]. Later outcomes were not different for those treated children with differing serotypes of parasites reflecting infection with different strain in these treated children [33••]. A comment prepared for a grant application by one of the NCCCTS neurologists and R. McLeod is included as follows, as it describes, in a different format, observations in the NCCCTS during these past 4 decades:

“*My goal as a contributor to this study is to understand the consequences of Toxoplasma infection across life, particularly as this infection affects the brain. I am an expert in Pediatrics, Child Neurology and Developmental Pediatrics. I have served as [one of] the primary neurologist [s] in this study for the evaluation of the neurologic status of the study patients since the inception of this study in 1981. I have evaluated most of the families involved in this study and I have enjoyed this work immensely. I have watched this symptomatic newborn disease transition from one that was assumed to always have a bleak prognosis at birth to one that many times is associated with good outcomes. It has also been fascinating to watch the development of understanding better management and pathogenesis of the infection. The many contributions our work has made to this understanding, and to define the next frontiers for both treatment and deciphering pathogenesis of this disease has been gratifying. It has been extremely rewarding to observe such children move on to University, to productive work and to beginning their own families, all unexpected outcomes at the outset of this work in 1981. (CNS)”*

Examples of physician resistance to considering possible benefit from shunt placement and potential good outcomes are in Fig. 4 and in the Box). In some cases, the need for a ventriculoperitoneal shunt is clear with anatomy indicative of obstruction of the Aqueduct of Sylvius with a dilated third ventricle and lateral ventricles, head circumference crossing percentiles and a full anterior fontanelle, and even then there sometimes is resistance to shunt placement. Drainage made through creating a small orifice at lower surface of the third entricle so it drains in the basilar cistern called a third ventriculostomy work less well with risk from inflammatory cerebrospinal fluid after anti-parasitic medicines are discontinued. In some cases where there is less obvious, clear potential benefit, and anatomy associated with an inflammatory, fibrotic pathogenesis without aqueductal obstruction, the approach we have used is summarized in this paraphrased comment by one of the NCCCTS neurosurgeons. This experience is also presented in the manuscript by McLone et al [50•] and in Fig. 4 [50•] with permission:

“*The shunt may help your child and one can only know by placing it and seeing if it does. No one can predict that and there are no guarantees but it is a hope that it will help him/her and we will all be fortunate if that happens. There are always risks but these are not uniquely worse for your child and not inevitable. Without the shunt your child will not have that chance. (DMc. DF, RP, RMc and more recently P. Das and J Ruge*)”

**Table:** 

**Box Update: Some strengths, benefits, and problems in access to, implementation, and understanding of the importance of various treatment options identified and exemplified in the following recent patient experiences/cases below:** **Case 1**: Infants who received treatment from large bottles of pyrimethamine and sulfadiazine stored for longer than one week developed severe and prolonged neutropenia. These are suspensions rather than solutions. They sediment and congeal resulting in under and overdosing. Medicines should be in 25ml bottles and made fresh each week adjusting dosages for the child’s weight. **Case 2**: Patient in USA was exposed to Toxoplasma *in utero.* The patient was treated in the first year of life and has done well. At the age of 16 years she developed a peripapillary lesion medial and superior to the optic disc with considerable fluid, cells and loss of visual acuity. Treatment with pyrimethamine and sulfadiazine with leucovorin led to resolution. Duration of treatment was for many months. Treatment is continued until lesions become quiet and resolve and then for several more weeks. It is not a disease where duration can be specified as a consistent, uniform length of time but rather depends on the patient’s clinical status. See Figure 5e and also Supplemental Figure 5. Azithromycin is used for suppression after complete resolution of active disease when frequent recurrences are in areas that are vision threatening, especially for patients whose vision is significantly impaired in one or both eyes. Azithromycin obviates the problem of hypersensitivity to sulfonamides that occurs from less effective trimethoprim/sulfamethoxazole. Failures of treatment of active disease with trimethoprim/sulfamethoxazole are not infrequent. Trimethoprim/sulfamethoxazole plus clindamycin has been used a few times when pyrimethamine was unavailable. Sulfadiazine has now been made available in Canada after a prolonged time when it could not be obtained there. **Case 3**: Patient from US developed congenital toxoplasmosis in utero. The disease advanced and resulted in significant cerebral edema. Neurosurgery was consulted for placement of a shunt but did not intervene until the disease was advanced. This case highlights that the problems in toxoplasmosis treatment are solvable. Greater interdisciplinary collaboration is needed across pediatricians, infectious disease providers, and neurosurgeons in order to improve patient care outcomes. A photo of the patient years after successful surgical intervention can be seen in Fig. 5. **Case 4**: 40-year-old body builder, took corticosteroids, presented with extensive encephalitis. He had steroid treatment and was found to have very low or no IgG, IgM, or IgA with this immunodeficiency a contributing cause. He developed sulfadiazine hypersensitivity, He initially responded to de-sensitization. But then developed a marked increase in liver function tests, some biliary sludge on ERCP, liver functions returned to near normal but then became elevated again. He was experiencing a slow neurologic rehabilitation when he developed inanition, and seizures and a cardiac arrest with unsuccessful attempts at resuscitation... Some severe illness due to *Toxoplasma* is associated with not previously recognized immune deficiency, genetic mutations or allelic variations in genes which confer susceptibility [47••, 117, 123••, 124••, 125••].	

Examples of resolution of a large symptomatic retinal lesion in a teenager in the United States are shown in **Figure** **5****.** Treatment was continued beyond the time the lesion was fully resolved. The approach to treatment of recurrences is to promptly utilize pyrimethamine, sulfadiazine and leucovorin (PSL) (or with azithromycin instead of sulfadiazine if there is hypersensitivity to sulfonamides). These medicines (PSL) have demonstrated better efficacy in animal models, a correct ratio of constituents for synergy and a higher trough level of the anti-Dihydrofolate reductase component of the combination than trimethoprim sulfamethoxazole which has resulted in treatment failures.

If there is monocular vision and lesions in the better seeing eye near to the fovea, or with multiple recurrences, after complete resolution of activity, suppression with azithromycin has been used without further recurrences. Duration for such suppression is not established.

Azithromycin is used only when QT interval is normal, i.e., not prolonged. Trimethoprim sulfamethoxazole is not used for suppression because of the high frequency of hypersensitivity to sulfonamides which then precludes further use of the better sulfadiazine with pyrimethamine combination if there is another recurrence (please see above). A school aged child responded to clindamycin TMP/SMx but an older patient relapsed quickly [McLeod et al unpublished observations, 2022]. These medicines were used because of difficulty in obtaining pyrimethamine initially and are not the treatment of first choice.

Additional examples of ocular manifestations of *Toxoplasmosis* are presented in Supplement to Part 1 (Fatima Clouser Atlas, Part I, Supplemental) and at Toxoplasmosis.org. Others were presented earlier in other manuscripts and Atlases and book chapters [1••, 2, 3••, 4••, 5••, 6••, 7••, 8••, 9••, 10••, 11••, 12, 13, 14••, 15••, 16••, 17••, 18•, 19•, 20•, 21•, 22•, 23, 24••, 25, 26, 27•, 28•, 29, 30, 31•, 32•, 33••, 34•, 35•, 36, 37•, 38•, 39••, 40•, 41•, 42••, 43••, 44••, 45, 46•, 47••, 48••, 49, 50•].

### Brazil and Colombia

The extensive well-organized work in Brazil and Colombia has been summarized recently [57•, 74••] and follows the same pattern of markedly improved outcomes in those who are treated. There have been comprehensive scholarly summaries of the programs for Brazil, that were completed earlier and recently [62••, 74••, 77, 151]. Brazil has played an important role both in clinical care on a large scale and in understanding parasite genetics as well as in basic and translational studies. Education programs in Brazil have included physician/scientists from Brazil, Europe, the United States, and Colombia with government sponsorship of programs for care. Collaborative work particularly in genetics and immunology/vaccines is included among the references herein and addressed in more depth in Part IV.

### Morocco

Morocco has utilized gestational screening for decades according to the French model but not with monthly testing. A robust program to eliminate congenital toxoplasmosis has also been initiated with more frequent serologic screening [57•]. A thousand women have now been tested. Educational programs have been initiated. Epidemiologic studies like those described for Panama and Colombia have also been performed recently. There is an ongoing study of screening of thousands of pregnant women in two practice settings and a program to screen twenty thousand women in the poorer, high prevalence agricultural areas are under consideration as part of a government initiative.

## Anti-parasitic treatments using medicines currently available in clinical practice: What is working and where are there problems?

As mentioned above, in France, Austria, and Brazil medicines are easily available and price is not an issue because of the countries’ policies on pricing and availability of medicines. In all the United States currently accessing medicines presents challenges. The recent history concerning the availability of medicines has influenced care for toxoplasmosis in the United States. In the United States in 2015, Turing Pharmaceuticals (now rebranded as Vyera Pharmaceuticals) acquired Daraprim (pyrimethamine), a medicine used to treat toxoplasmosis, and hiked the price by over 5000%, from $13.50 a pill to $750 for commercially insured patients. This caused substantial concern with broad criticism from the press and in senate hearings. The company defended the increase by stating that only a small percentage of patients had commercial insurance and for those uninsured medicine would have no charge (“penny pricing”), and that the profits gained from the price hike would be used to develop better treatments for toxoplasmosis and that patient co-pay would not be affected. Six years later, the cost of Daraprim has remained at $750 per pill with no public news of Vyera’s alternative toxoplasmosis medicine(s) since its approval to initiate a phase 1 study of a DHFR inhibitor in 2018. There are two candidate molecules with earlier phase studies but no public news as yet of their advancing for clinical use. The current pace of progressing medicines into clinical use for SARS-CoV-2 provides a stark contrast.

The history of pyrimethamine as a medicine is relevant: Pyrimethamine was discovered in the 1950s and has remained a major component of the treatment for toxoplasmosis throughout the subsequent decades. However, with only about 2000–8000 Americans recognized to be in need of this treatment annually, there was little incentive for other pharmaceutical companies to develop generic versions. As such, despite costing pennies to manufacture, companies like Turing/Vyera were able to increase the price by also enacting anti-competitive practices including tightly controlled distribution. As of October 2020, there are two additional generic versions of pyrimethamine, but the pricing has not been lowered significantly. Without insurance, this medicine is provided without charge. Only a relatively small percentage of the pyrimethamine is paid for by indemnity insurance and pre-approval can now involve substantial delays. Initially insurance coverage seemed to occur with relatively little difficulty and there was a robust assistance program and prompt distribution of medicines was possible. More recently, insurance company delays and refusals have made obtaining this medicine challenging for physicians, this has presented considerable difficulties in promptly providing emergent medicines for those who do not have 340B pharmacies. Such 340B pharmacies that can provide medicine at nominal or no cost while waiting for insurance coverage to be clarified (bridging the provision of medicines while waiting for medicines to be delivered) and for uninsured patients with no delays. These delays and difficulties can be harmful for patients. Bridging programs may help to remedy this in some instances. Hospitals with pharmacies that can provide medicines at low costs to patients who are medically indigent (340B pharmacies) can help with bridging when patients are registered there and some of these have obtained approvals to ship to patients in other states. For example, The University of Chicago is authorized to ship to California, New York, Georgia and Florida for patients registered at the University which can be accomplished following a telehealth appointment. Thus, they can assist patients in those locations. There are also pharmacy assistance phone numbers physicians can contact for assistance with obtaining medicines for the different companies with affiliated pharmacies. There is a charity that can assist patients also (phone numbers are available from Vyera “Daraprim Direct”). Unfortunately, as the charity provides support for many medicines, it is often depleted and runs out of resources early in each year. Therefore, it does not fulfill the desired goal throughout the year. Thus, obtaining medicines is feasible, even if substantial extra work for physicians, but has elements that currently do not function well. This information can allow patients and their physicians to know that proven treatments can be available to them, but it can be cumbersome, time consuming and result in delays or denials with insurance pre-approvals. There has been a recent anti-monopoly settlement with the FCC for multiple states and a patient class action lawsuit is pending.

While Vyera Pharmaceuticals has introduced programs, called “Daraprim Direct,” to help patients with financing their treatment plans, the price hike has clearly inflicted delays in obtaining medicines in urgent circumstances and suffering for Americans with toxoplasmosis. As described above, Daraprim Direct currently offers free medication to those uninsured and under 500 percent of the federal poverty level. Company coupons providing $10 monthly treatment regimens are also available for commercially insured patients whose insurance continues to deny coverage for this medicine when an appeal for a pre-authorization is submitted, as long as physicians continue the time-consuming appeal process. The limited distribution of Daraprim due to its cost creates other problems for patients, namely the lengthy wait times as physicians negotiate with insurance providers. Where treatment of emergent infection requires immediate attention, these delays can cause irremediable damage, such as loss of sight and death due to difficulty from diminished access. 340B pharmacies have provided medicines in emergencies, to be replenished when insurance allows so no patient suffers.

Finally, those under Medicare Part D face 31% copay due to Daraprim’s placement as a Tier V drug based on cost. Such patients, often under standard triple therapies such as prescriptions of Daraprim, Leucovorin, and sulfadiazine, face monthly fees of over $4000 for multi-month regimens that must often be repeated for reemergence of toxoplasmosis symptoms. Without the price hike, monthly fees would be merely $80 due to the comparatively lower prices of Leucovorin and sulfadiazine. As a result of these issues facing Daraprim prescription, some physicians choose to fall back to cheaper and less effective alternatives such as trimethoprim sulfamethoxazole or clindamycin, with two physicians recently trying the two together without any data to support this.

More recently, two additional companies gained approval for generic pyrimethamine but for a longwhile had chosen to continue to list the drug at prices nearly as high as the initial high price by Vyera Pharmaceuticals, rather than offering an affordable alternative. This has made access more complex with prior approvals, taking time and substantial delays.

As above at the University of Chicago, a system that permits prompt access for 340B pharmacies while providing bridge medicines, obviates some of these problems but it remains cumbersome and difficult to deal with pharmaceutical company preapprovals. Further problems with access and delays have become more pronounced with limitations in access to care during the SARS-CoV-2 pandemic (see representative cases, tabulated data in Tab 4, Box 1).

**Table 4:** Representative Illustrative Cases During COVID-19 Pandemic Presenting to Toxoplasmosis Research Institute and Center for Patient Care

**Patient Location**	**Unusual Exposures/Demographics**	**Diagnosis (Y/N)**	**Systemic Symptoms**	**Eye Disease**	**Brain Disease**	**Other Considerations Concerning Treatment**
**Pregnancy**						
Singapore	No known exposures in screening and pregnancy	Y; Serum antibodies in mother	Rule-out sepsis; chorioamnionitis	None	None	Prompt treatment; negative amniocentesis at 15 weeks of amenorrhea; antibody load*; treatment initiated promptly
Upstate New York	Kittens	Y; Seroconverted	Mother seroconverted	None	None	Mother treated promptly
New York City, New York	None	Y; Acute serologies	Mother screened	None	None	Spiramycin; negative amniocentesis; awaiting baby's birth
**Newborn**						
Guam	None	Y; Serologies and clinical	Less systemically ill	Yes	Hydrocephalus; severe brain disease	Difficulty in obtaining pyrimethamine; refusal of Neurosurgeons to shunt
Cincinnati, Ohio	None	Y; Serologies	On ventilator	Yes	Severe brain disease	Refusal of neurosurgeons to perform shunt procedure; medical treatment initiated
Tallahassee, Florida	Homeless, lived in swamp; feral cats in geographic area; born by side of road	Y; Delayed	Low platelets; rule-out sepsis	Bilateral chorioretinal scars	Paralysis of leg; spinal cord lesion; hydrocephalus (Fig. 4)	Substantial delays in shunting and starting medicines
Houston, Texas	Mother, homeless	Y; Delayed	Yes	Yes	Yes	Treatment missed
Lancaster, Pennsylvania (Female twin)	None	Y; Delayed	[47••]	None	None	Approach to late diagnosis
Lancaster, Pennsylvania (Male twin)	None	Y; Delayed	[47••]	Large retinal scar	Ventricular dilatation	Approach to late diagnosis
Hawaii	High referral site	Y	In some cases	Yes	Yes	Treated
Canada	High referral site	Y	Yes	Yes	None	Treated
Mississippi	High referral site	Y	Yes	None	Few calcifications	Medication issues
Mexico/Miami, Florida	High referral site	Y	Yes	New active lesions	None	Hypersensitivity to sulfadiazine, difficulty obtaining medicines; late diagnosis
Cleveland/Sandusky, Ohio	High referral site	Y	Yes	Yes	Yes	Preventable severe disease; family cluster of infection
Akron, Ohio	High referral site	Y	Yes	Yes	Yes	Need for ventriculoperitoneal shunt
**Later Eye Disease**						
Coastal Valley, California	High referral site; family from Central America	Y	Yes	Reactivated eye disease	Yes	Delay in obtaining medicine
Fort Myer, Florida	COVID positive	Y; Serologies and clinical	None	Recurrent eye lesions	None	Difficulty confirming resolution of lesions
Miami, Florida	None	Y; Serologies and clinical	None	Recurrent eye lesions	None	Recurrence treated without difficulty; suppression
Bloomington, Illinois	Middle aged	Y; Serologies and exam	None during recurrence	Yes	None	Response to treatment
Chicago, Illinois	Late teenager	Y; Exam	None during recurrence	See: photos (Fig. 5)	None	Recurrence resolved
Chicago, Illinois	Late teenager	Y; Exam	None; only eye symptoms	See: photos	None	No activity
Chicago, Illinois	Late teenager	Y; Exam	None; only eye symptoms	See: photos	None	No activity
Boston, Massachusetts	Young adult	Y; Exam	None during recurrence; GI symptoms	Unilateral loss of sight	None	Delay in obtaining medicine harmed patient outcome
Near Lancaster, Pennsylvania	Late teenager	Y; Exam	None during recurrence	None	None	Recurrence resolved
India	Middle aged	Y; Serologies	None during recurrence	Permanent eye damage	Seizures	Delay in obtaining medicine resulted in permanent damage
Mexico	None	Y; Serologies and clinical	None	Recurrent eye disease	None	Recurrence with difficulties in obtaining medicines
New Jersey	Born in Guatemala; international adoption	Y; Serologies and clinical	None	Yes; bilateral macular chorioretinitis	None	Concerns regarding improving vision
Chicago/North- brook, Illinois	None	Y; Serologies and clinical	Present at birth	Yes	Yes	Illness at birth demonstrated importance of diagnosis of Toxoplasmosis with low platelets, rule-out sepsis; hydrocephalus; complicated illness (hypothermia, UTI's, need for shunt setting adjustment)
**Adults/Older Children Postnatal**						
Grass Valley, California (grandmother)	Consumed deer heart	Y; Serologies	Fever; malaise	None	Severe neuro-psychiatric issues; lucent areas in brain MRI of uncertain significance	No improvement after months; treated
Grass Valley, California (mother)	Consumed deer heart	Y; Serologies	Fever; malaise	None	None	Improved gradually within months with treatment
Grass Valley, California (child)	Consumed deer heart	Y; Serologies	Fever; malaise	None	None	Improved in weeks; not treated
Near Atlanta, Georgia	Adult male	Y; Serologies and clinical	Heart disease bradycardia; malaise	Significant	None	Difficulty in obtaining medicines†, however, no delays; responded to treatment
Rochester, New York	None	Y; Serologies and clinical	Present at birth	severe bilateral retinal disease	Manifestations of spinal cord/brain lesions later in life	Ongoing illness later in life
Latvia	None	Y; Serologies	Yes	None	None	Persistent symptoms; unexplained, possible amyloidosis, being evaluated
Seattle, Washington	None	Y; Serologies and clinical	Fatigue; eye disease	Yes	Seizures	Prolonged illness
Kentucky	None	Y; Serologies and clinical	Malignancy	None	Yes; brain lesions	Required treatment involving slow resolution after transplant
New York City, New York	None	Y; Birth	Yes	Yes	Yes	Recurrent eye disease responds to treatment
South Carolina	Laboratory accident in first trimester of pregnancy	Infection prevented	No	None	None	Use of azithromycin in emergency in first trimester

Y=Yes, N=No, COVID refers to SARS CoVi 2 pandemic

Very recently a pharmaceutical company called Oakrum began to sell pyrimethamine at ~$0.30 per 25 mg tablet which could mean that this resolves the problems associated with cost in the United States. It has not been possible at present to learn where this is manufactured or if there is any problem with quality or safety, or where the starting material (API) originates. If this is really a low-price tablet of high quality from a reliable source it could mean the treatment of this disease with optimal medicines will be feasible, straightforward and convenient again. It could lead competitor company prices to become comparable, and easily affordable

In Tables 4, 5, Box and Figs. 4, 5, we present approaches that have worked effectively where problems have arisen. These problems include loss of sight, inadequate access to neurosurgical shunt procedures, lack of recognition of well-known presenting manifestations of CT in neonatal Intensive Care Units, complications with laboratory access, and limited availability of prenatal care during the COVID-19 pandemic further complicating healthcare.

**Table 5:** Why Should We Care and Should All Toxoplasma Infection be Diagnosed, Treated Promptly and Eliminated when possible or manifestation reduced, and If So, What Steps Should be Taken to Do So

	**Setting**	**Consequences in** **Terms of Suffering**	**Cost to Society**	**Benefit to** **Society**
**Utero**	Gestational testing	A Mother's Testimony: A Presentation to the Illinois Legislature in Support of Education and Screening [61]	US, Austrian, Analyses [53••,^56^••] France prenatal/postnatal versus only postnatal(in submission)	14-fold cost saving [53••], Mother's testimony [61], Spillover benefit [56••], Concurrent screening Toxoplasma G and M, hepatitis B, HIV, CMV, Syphilis, Rubella [81••] to decrease maternal-child mortality and morbidity
**Children**	Illness at birth and neonatal screening	NCCCTS [Table 1]	[56••]	Not perfect but better than no treatment
**Adults**	Acute and chronic infections	Contribution to loss of cognitive function, epilepsy, malignancy, neurodegeneration [47••]	Large losses and acute and chronic infections	Elimination of disease and suffering, better functioning, and costs for care saved
**Older Adults**	Acute and chronic infections	Contribution to loss of cognitive function, epilepsy, malignancy, neurodegeneration [47••]	Large losses for acute and chronic infections	Elimination of chronic diseases where *Toxoplasma* is an initiator and a progressor of chronic diseases
**Immunocompromised**	Acute and chronic reactivated infection	Heart transplant patient from donor [94], patient with bone marrow transplant, AIDS patient in the Midwest [95]	Suffering, loss of life, healthcare costs	Saving lives, sight, cognition, quality of life
**Eye Disease**	Most common infection of retina worldwide	Loss of sight and function	Large costs to society	Saving sight and quality of life

Spiramycin, used in the first trimester to prevent infections of the fetus, has been provided since the 1990s at no charge in the United States when it was cleared by Dr. McLeod, or the Remington Specialty Laboratory, and then the FDA. IRB approval is needed after the first emergency use for a practitioner. Benefit from this medicine particularly in the first 14 weeks of gestation recently was noted in a meta-analysis [68••]. This medicine has been available in Panama and Colombia commercially, as it is in Europe, Brazil, Argentina, and Guyana.

The National Collaborative Toxoplasmosis Study (NCCCTS) and other published materials provide guidelines for treatment which also are outlined in the manuscripts and book chapters on Management of congenital toxoplasmosis [60••, 65, 70, 71••, 72••, 73••] and updated and summarized herein (Fig. 1). The work of Mandelbrot et al [60••] indicates that prompt use of pyrimethamine and sulfadiazine can result in better outcomes after 15-weeks’ gestation for a small subset of infected fetuses [60••].

There is an important distinction that is often overlooked in comparing data concerning prevention of CT in the literature in studies from France and work in the United States. In the United States, conception is dated from two weeks after the last menstrual period. Whereas in France, gestational week is counted from the onset of amenorrhea. Thus, in the literature from France, gestational age at which amniocentesis is performed is written as 17 weeks amenorrhea, this would be 15-weeks gestation in the United States. When dating of gestational age is considered the date of conception would be 2 weeks later than the onset of amenorrhea.

## Parasite and Host Genetics

Serotyping assays have previously allowed us to distinguish infections caused by Type 2 strains from all other strains, collectively referred to as NE-II within the NCCCTS cohort [33••]. A new assay utilizing additional polymorphic peptides expands the utility of the earlier assay, and now resolves infection caused by Type 1, 2, 3, haplogroup 12 and non-archetypal parasites [148, 149]. This expands the utility of the earlier methodology used in the NCCCTS cohort and makes it possible to determine the strains causing infections in regions of the world where Toxoplasma genotypes are more heterogenous, as found in Colombia and Panama. Furthermore, limited genotyping studies performed on a few clinical samples within the USA by Pomares et al [150] suggest that type 1, 2, 3, and haplogroup 12 parasites dominate and cause illness in patients in the United States, providing the impetus to re-serotype the NCCCTS cohort with the new assay. Additional host susceptibility genes have also been identified (Tables 1, 2 and 3) [47••, 123••, 124••].

## Conclusions

As reviewed herein, a great deal has been learned about optimal diagnosis and management from work in France, Austria and the USA Work in Brazil [152, 153••, 154], Colombia, and Morocco added substantially to this in recent decades (as also reviewed elsewhere comprehensively recently [57•,^65^, ^74^••]. In some cases, the need for a ventriculoperitoneal shunt is clear with anatomy indicative of obstruction of the Aqueduct of Sylvius with a dilated third ventricle and lateral ventricles, head circumference crossing percentiles and a full anterior fontanelle, and even then there sometimes is resistance to shunt placement. However, benefits of ventriculoperitoneal shunt to treat ventricular dilatation are considerable, as are benefits from using available medicines promptly to treat active disease for pregnant women, those immunocompromised, and for those with ocular disease.

Recent data and updated clinical experiences demonstrate that treatment for this disease when it is active should be considered to be emergent with outcomes expectant to be favorable at the outset. Recent progress to make screening easier and less expensive in France, Austria, and the United States, to detect *Toxoplasma* infection acquired in gestation and to facilitate prompt emergent treatment, is considered in depth in part IV of this series of manuscripts. Some solutions concerning costs and current conditions concerning medicines are also considered. Parts II, III, and IV of this series address individual topics in building these programs, the role of contamination of water sources and the environment. They provide detail concerning educational interventions (II), risk factors (III), and public health (IV).

## Supplementary Information

ESM 1 (PDF 9974 kb)
