# Supplementary material for: Building Programs to Eradicate Toxoplasmosis Part I: Introduction and Overview
Source: Curr Pediatr Rep. 2022 Aug 22;10(3):57–92. doi: 10.1007/s40124-022-00269-w (PMC9395898; doi:10.1007/s40124-022-00269-w)
Supplement: Supplementary file 1 — (PDF 9974 kb) [file 40124_2022_269_MOESM1_ESM.pdf]

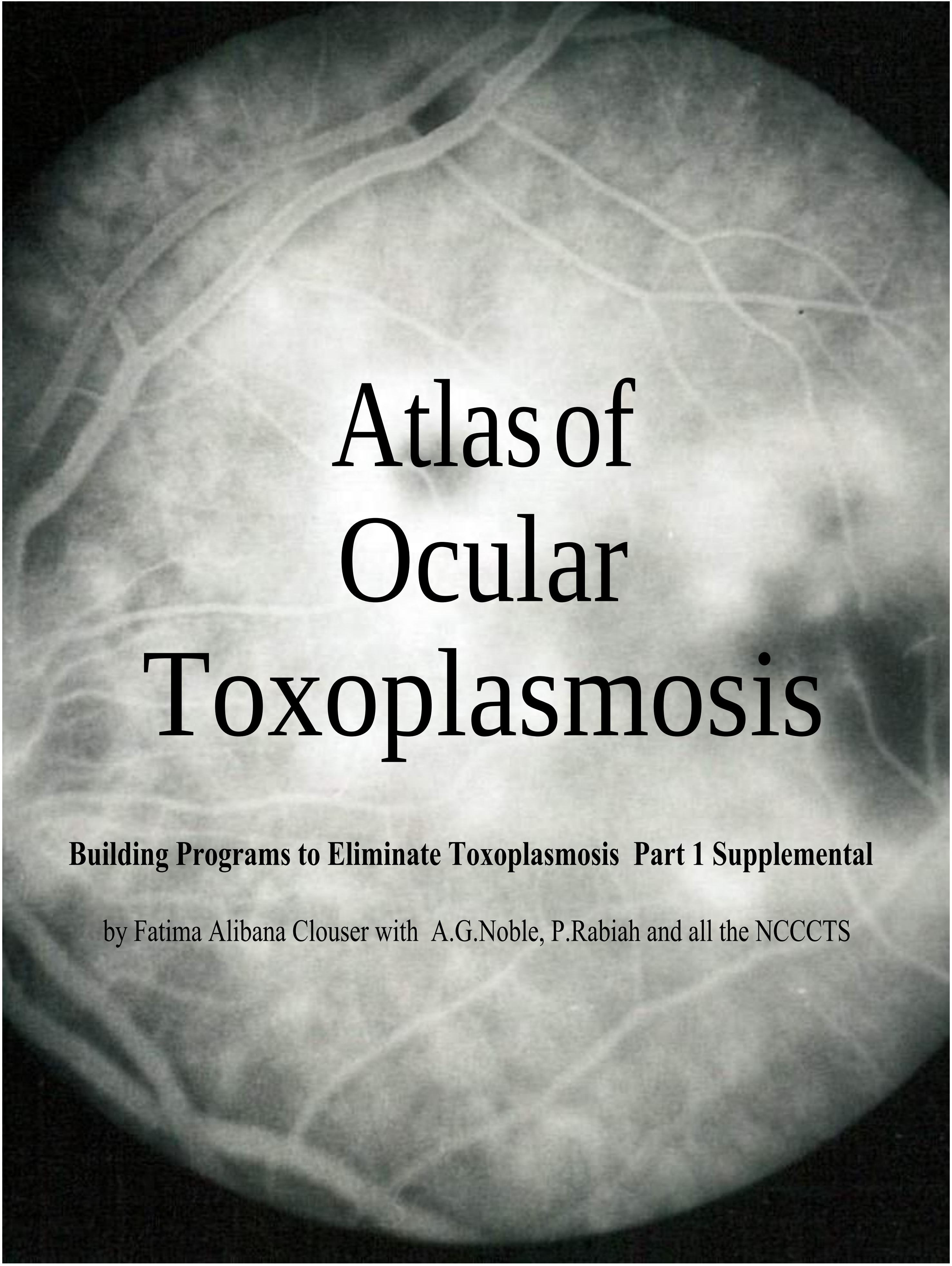

Atlas of Ocular Toxoplasmosis

Building Programs to Eliminate Toxoplasmosis Part 1 Supplemental

by Fatima Alibana Clouser with A.G.Noble, P.Rabiah and all the NCCCTS

Table of Contents

I. Introduction

- a. Normal Fundus-Plate Nº 1

II. Types of Toxoplasmosis

- a. Congenital
  - a. How to diagnose
  - Clinical Manifestations
    - New lesion
    - Active lesion
    - Quiet lesion
    - Lesion Progression with treatment
    - Phases through life
    - Newborn
    - Children
      - Other considerations: Amblyopia in Children from Toxoplasmosis
      - Twins
    - Teens
    - Adulthood
    - Late Adulthood
- b. Acquired
  - a. Mothers
- c. Other considerations
  - a. Visual Field defects
  - b. Low Vision
  - c. Amblyopia
  - d. Retinal Detachments

III. Imaging Modalities

- a. Past
  - a. Slide Film
  - b. 35mm film
- b. Present
- c. Fundus photography
- d. Wide field photography
- e. Future directions
- f. Supplemental testing-OCT, FANG,

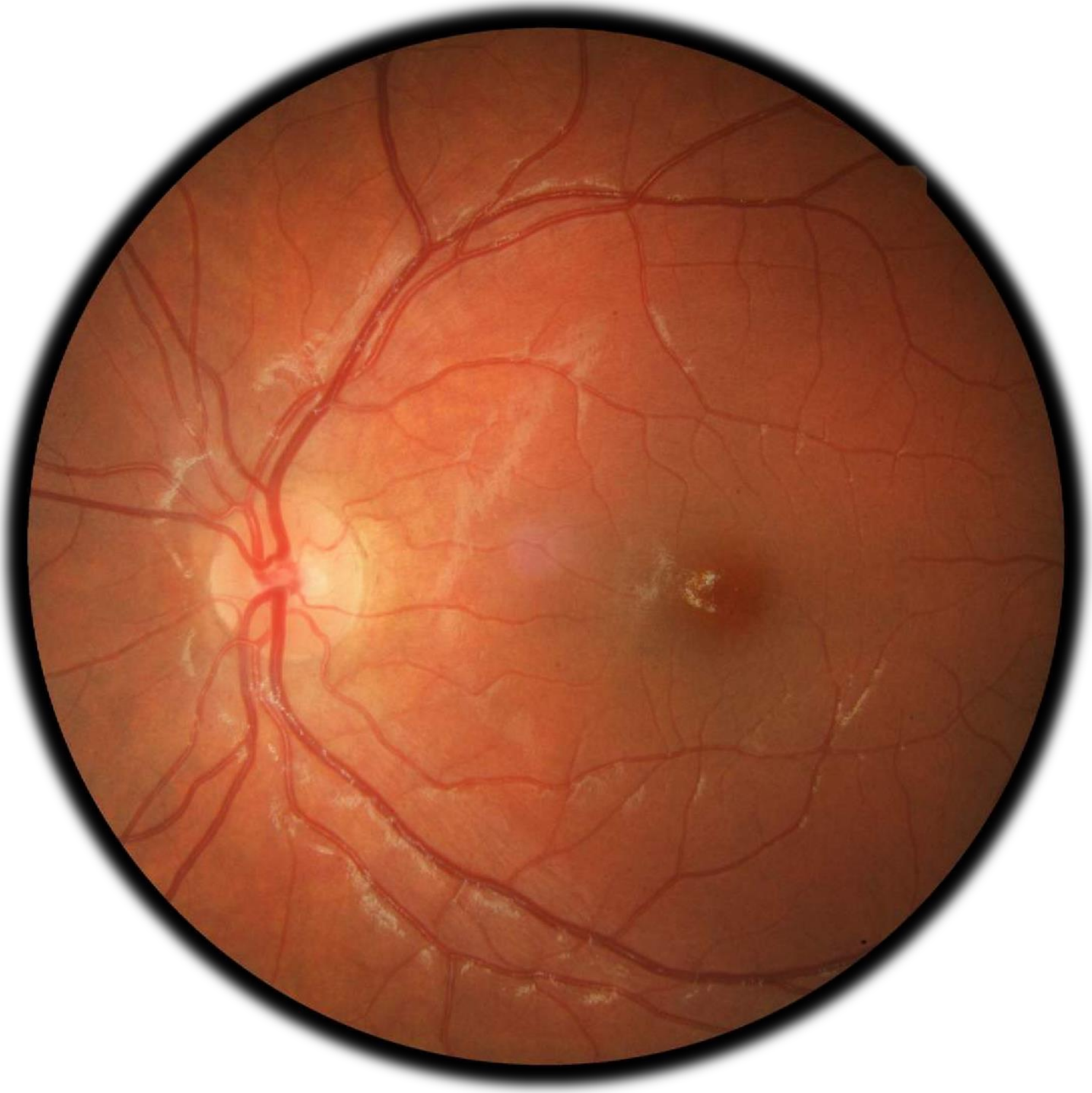

Plate No. 1- Normal Fundus

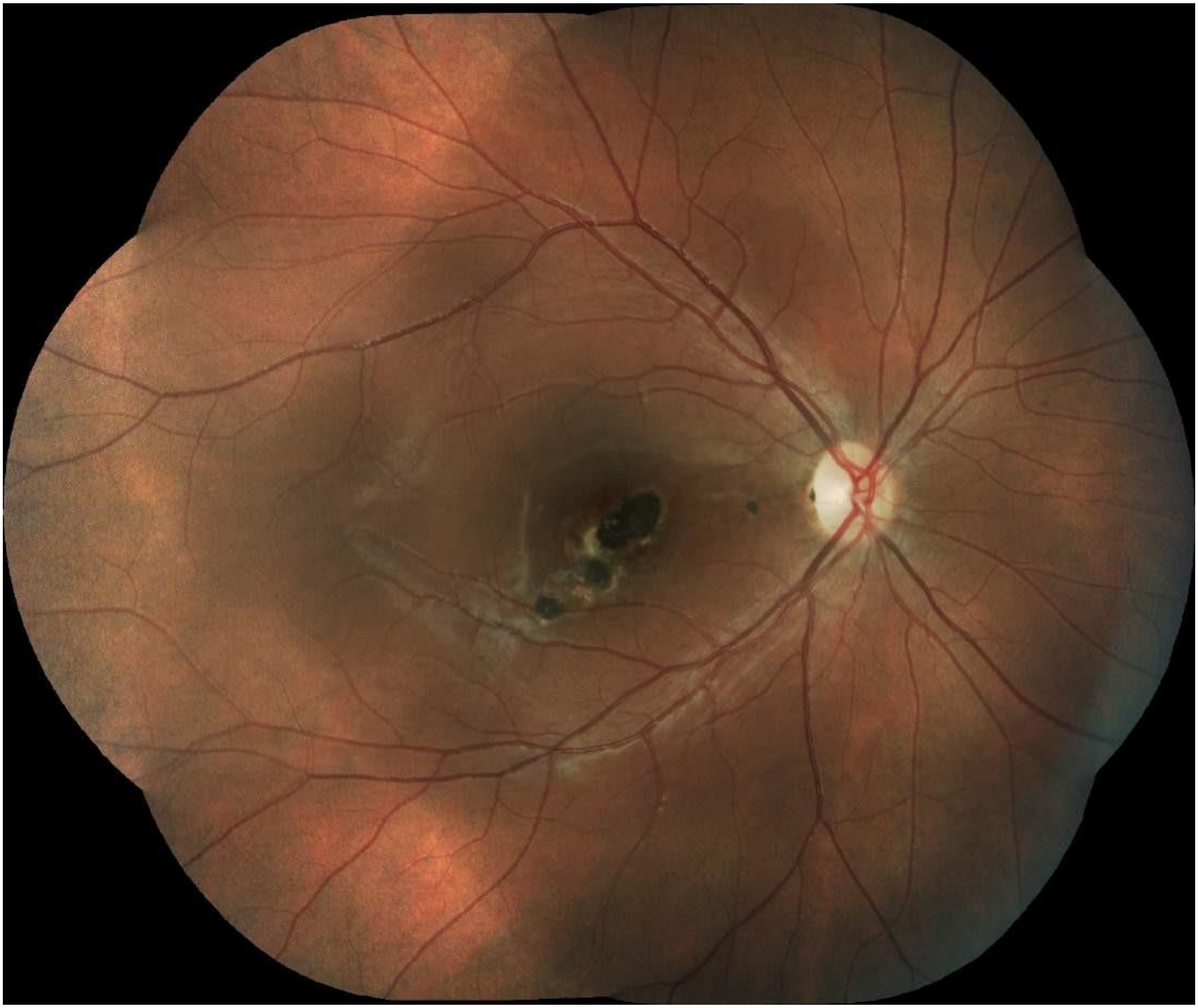

Plate No. 2- Patient A: 4 hyperpigmented chorioretinal lesions in the macula. Largest lesion is oval with slightly irregular margins which are sharp and is surrounded by a thin, hypopigmented margin. This lesion involves the nasal portion of the fovea only and much of the fovea is not affected by this lesion followed by a trail of satellite lesions. Small pinpoint of dark pigment on the temporal margin at 9 o'clock of optic nerve is not a lesion.

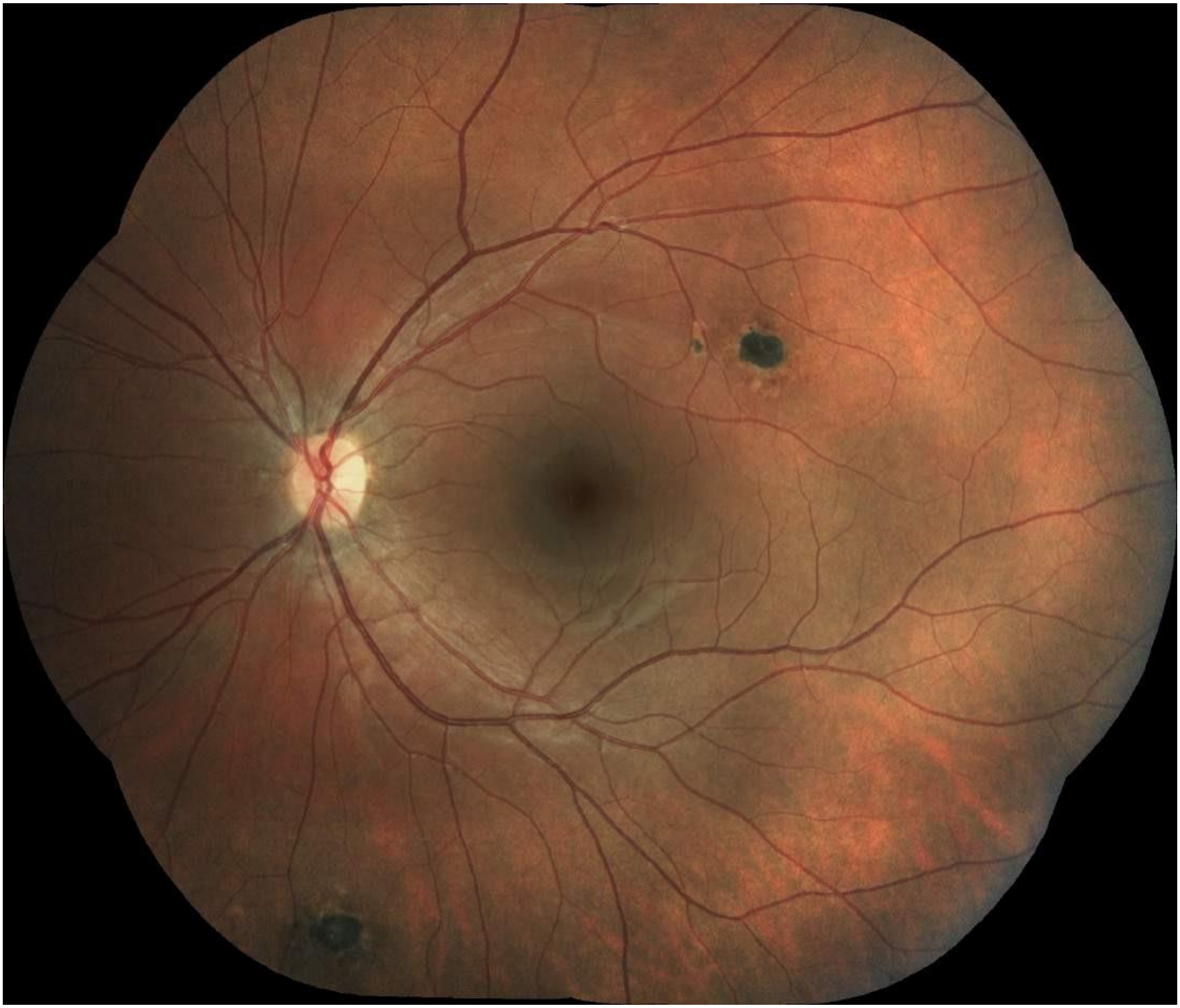

Plate No. 3- Patient A: Oval, well demarcated hyperpigmented lesion circumscribed by a thin hypopigmented rim which lies superior temporal to the fovea with a small, round satellite lesion in proximity. In the periphery (add in relation to optic nerve position) there is similar hyperpigmented lesion with irregular borders with a hypopigmented rim.

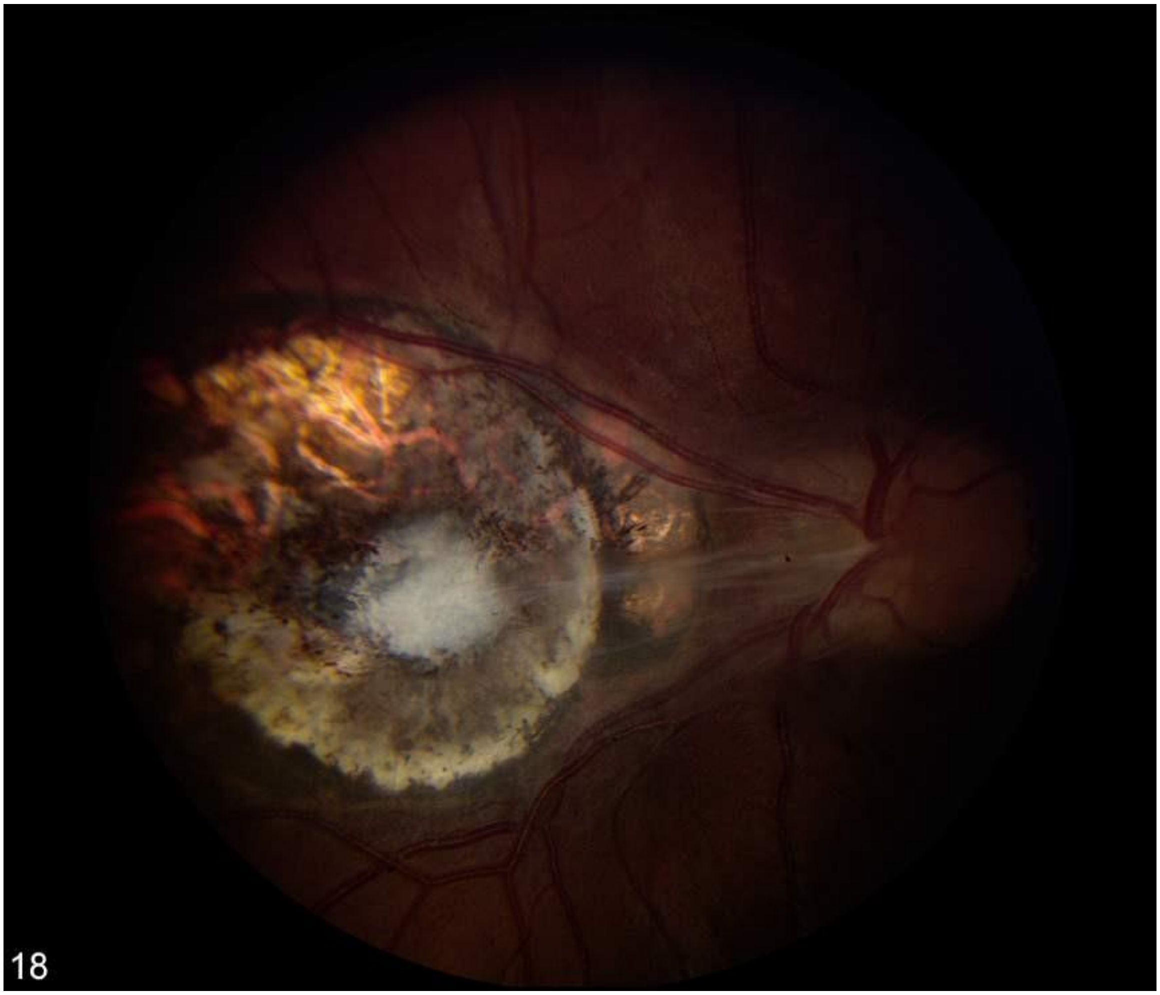

Plate No. 4 and 5-Patient B: The optic nerve and vessels of the optic nerve are both dragged temporally by the very large macular chorioretinal scar. The superior and inferior temporal vascular arcades are straightened over the large chorioretinal scar. In the macula there is a very large chorioretinal scar involving the entire macula from the optic nerve to the end of the temporal vascular arcades including the fovea. The central scar is white, deeply excavated down to bare sclera, exposing choroid layers and vessels. There are several bands of white fibrosis which extend from the central vessel in the optic nerve to the deep white center of the scar. This central deep white scar is surrounded by a mixture of hyperpigmented scar temporally and a mixture of grey and white scar superiorly, nasally, and inferiorly. Both the crescent shaped nasal part of the scar and the larger central scar are both circumscribed by a thick hyperpigmented margin with an adjacent halo. There is no normal macular tissue detected within the temporal vascular arcades.

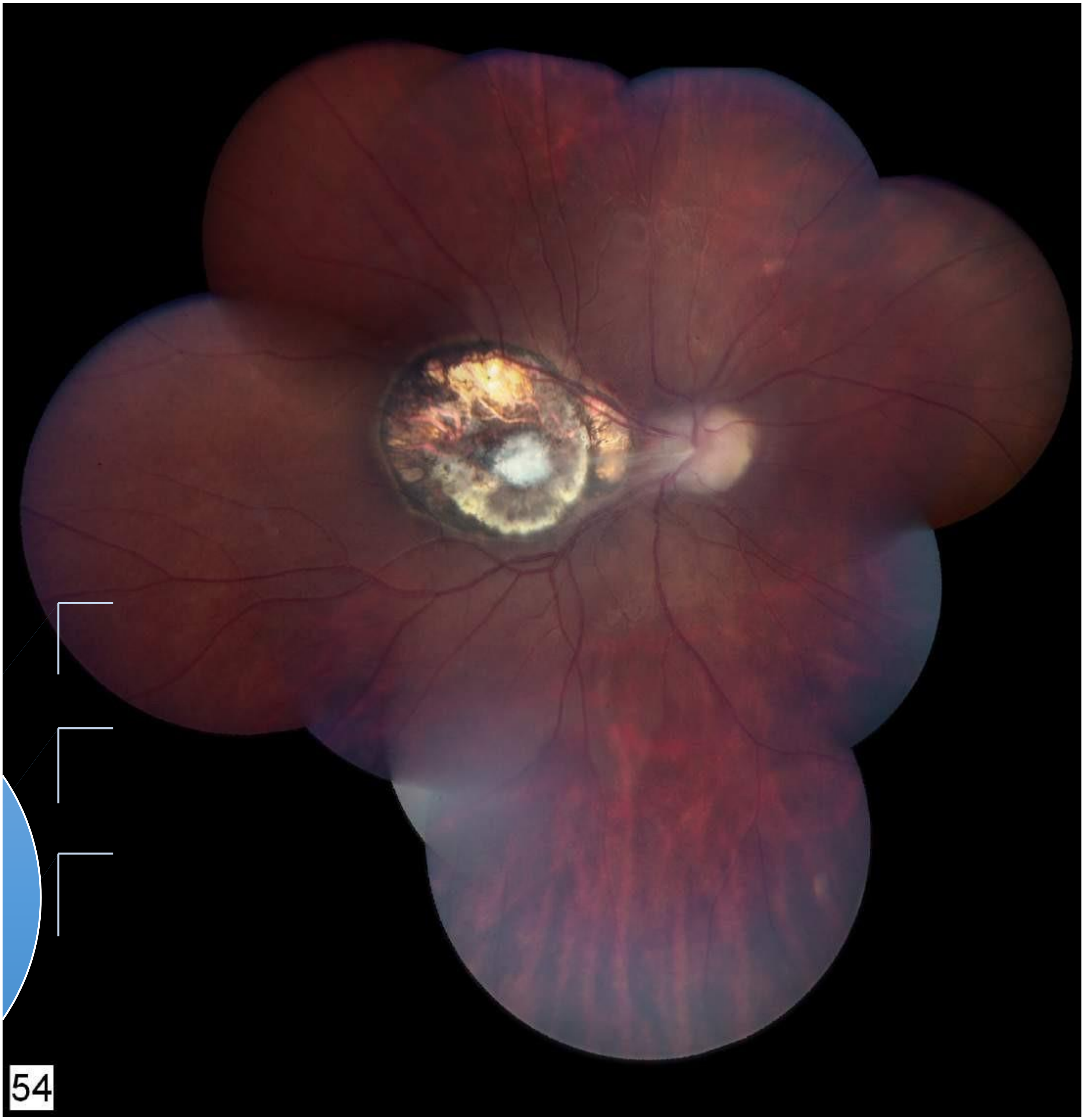

Plate No. 5

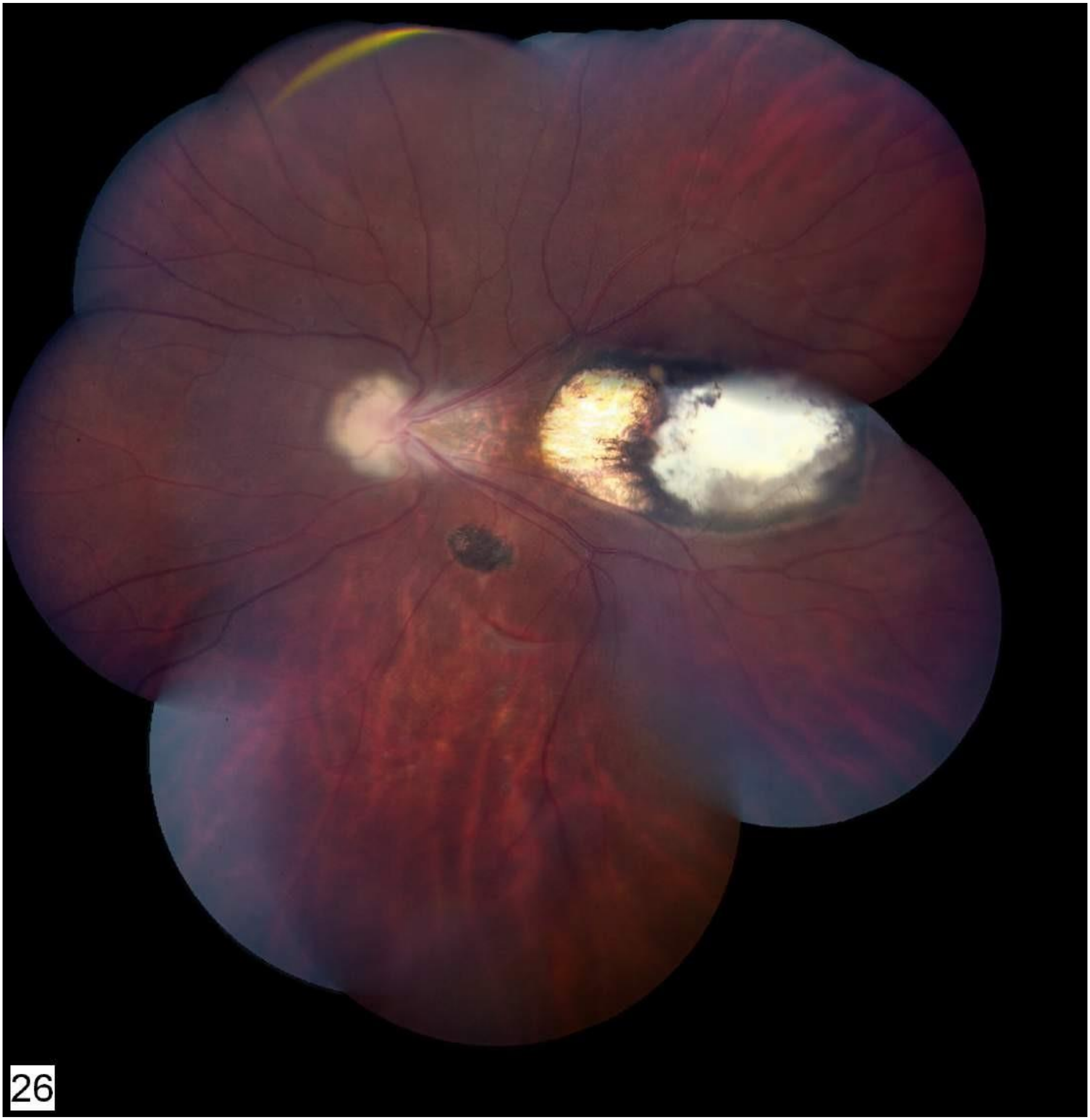

Plate No. 6-Patient B: The optic nerve is abnormal with little color, the nasal margin is mildly blurred with a small area of peripapillary pigment from 10 o'clock to 12:00 o'clock, and a scleral crescent nasally. The temporal part of the optic nerve is obscured by the chorioretinal scar which begins at the central optic nerve vessels. The optic nerve and vessels of the optic nerve are both dragged temporally by the very large macular chorioretinal scar. The superior and inferior temporal vascular arcades are straightened over the large chorioretinal scar. In the macula there is a very large chorioretinal scar involving the entire macula from the optic nerve to the end of the temporal vascular arcades including the fovea. The temporal part of this scar is white, deeply excavated down to bare sclera. The large nasal crescent of the scar is yellow choroid with hyperpigmented streaks and with the vessels of the choroids visualized. The central deeply excavated scar and the nasal crescent are both circumscribed by moderately thick hyperpigmented margin with an adjacent halo. The scar also involves the temporal part of the optic nerve and the juxtapapillary area; here, the triangular scar is grayish with the choroidal vessels showing through. Between this peripapillary scar and the larger circumscribed macular scar is an isthmus of macular tissue which is mildly abnormal.

Approximately three-quarters of a disc diameter inferior temporal to the optic nerve outside the inferior temporal vascular arcade there is a small, densely hyperpigmented chorioretinal scar with a small lacuna nasally; it is approximately one-half of a disc diameter in size. (Note: This lesion was previously reported as superior temporal to the optic nerve; however, review of photographs verified the inferior temporal location of the chorioretinal scar). A good view of the periphery was obtained and there were no other chorioretinal scars detected peripherally.

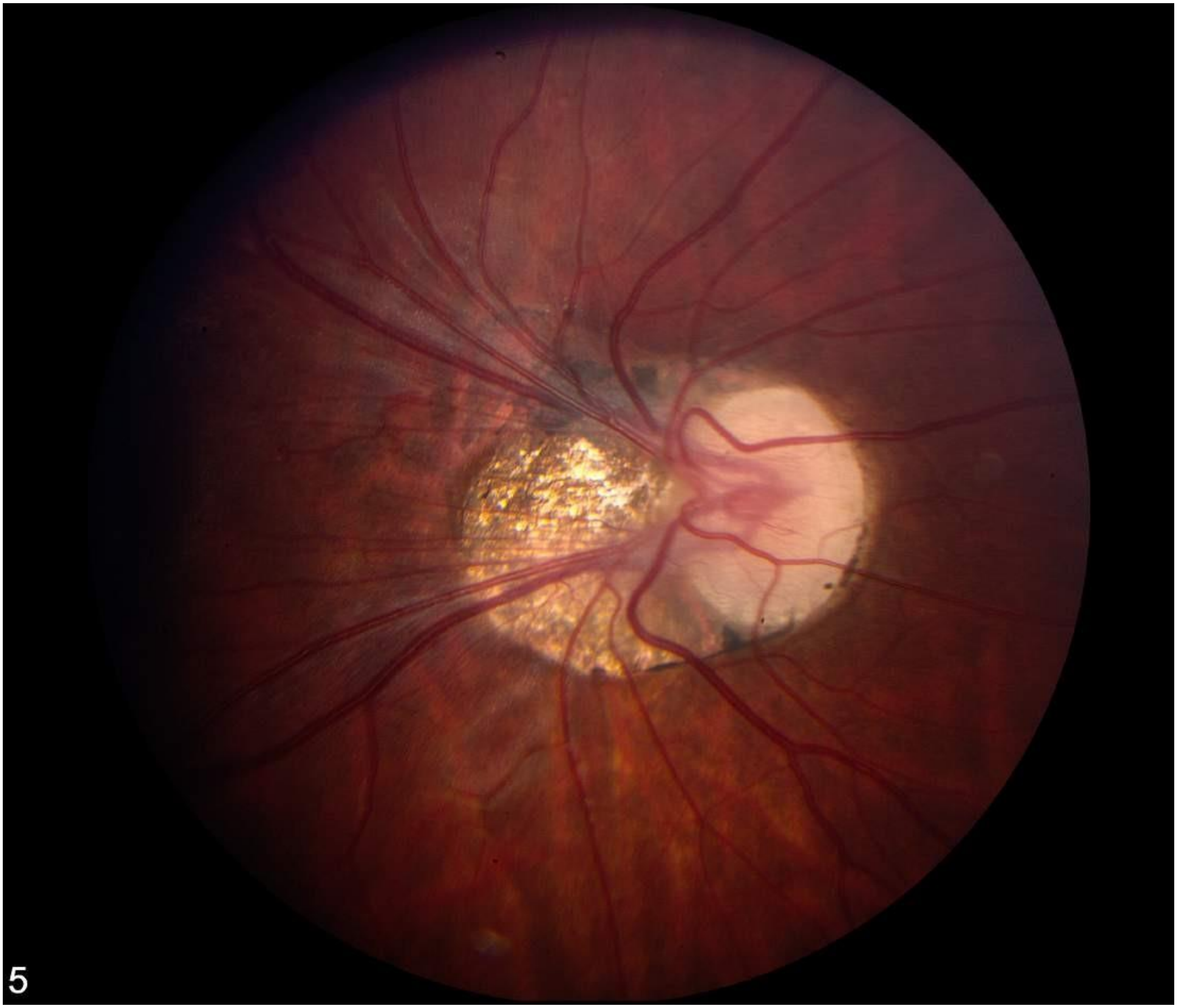

Plate No. 7- Patient C: Juxtapapillary (or peripapillary?) chorioretinal scar contiguous to the temporal side of the optic nerve that is nonactive with perivascular sheathing causing straightening of the vessels.

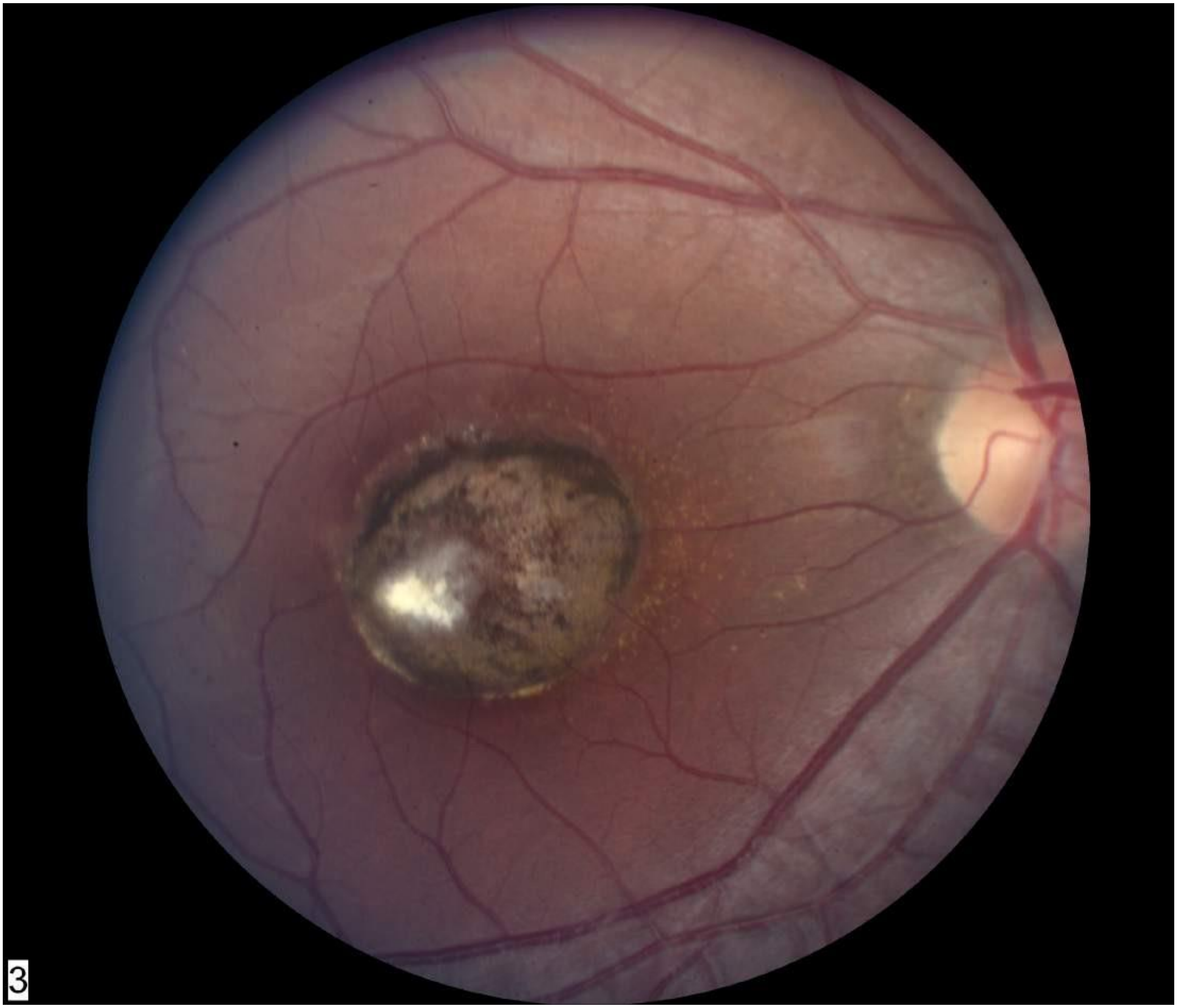

Plate No. 8 – Patient D: Moderately large, characteristic toxoplasmosis chorioretinal scar with sharply demarcated margins, encompassing the fovea in the central macula.

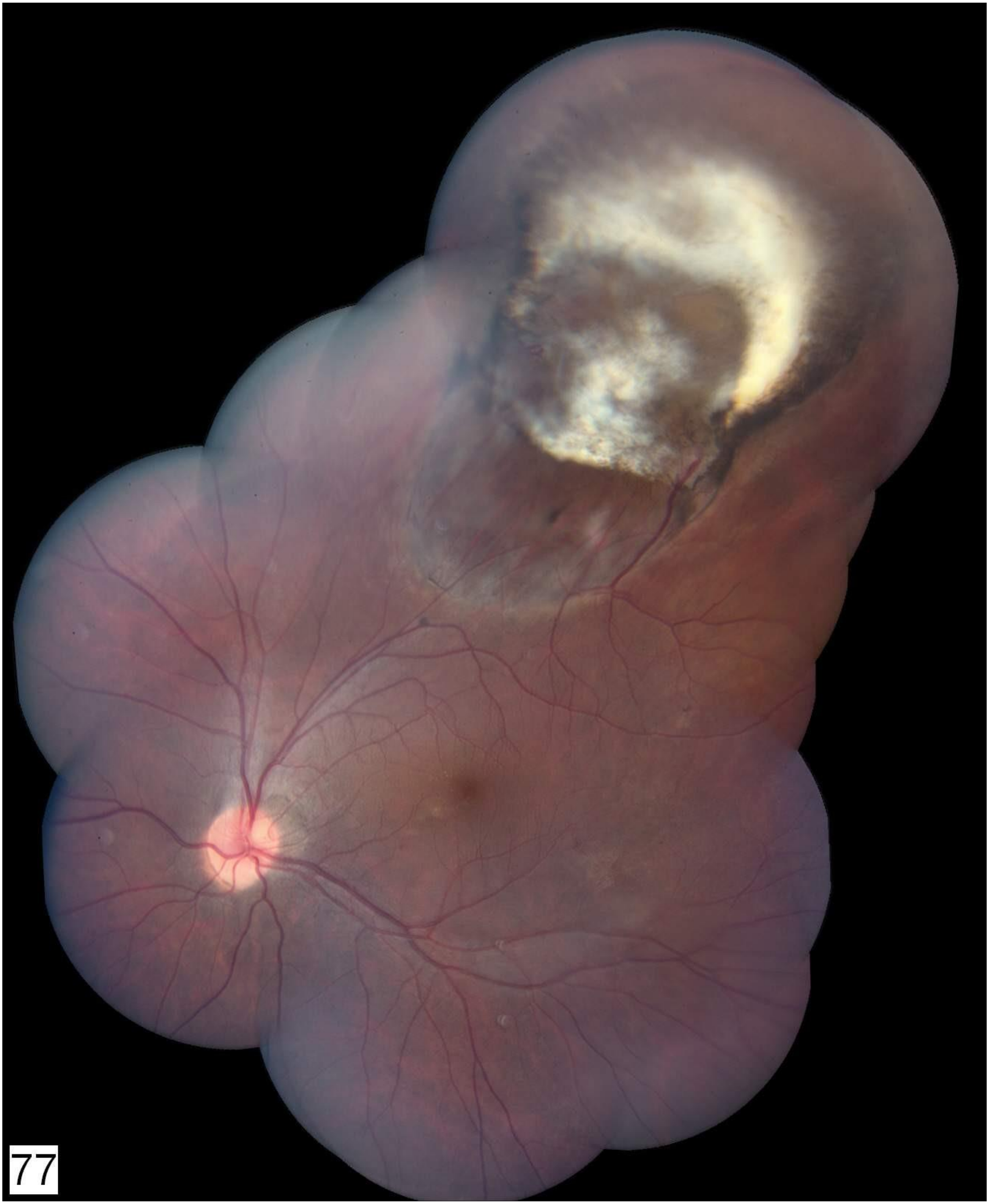

Plate No. 8 – Patient D: Superior temporal vascular arcade is pulled straight by the larger mid-peripheral chorioretinal lesion.

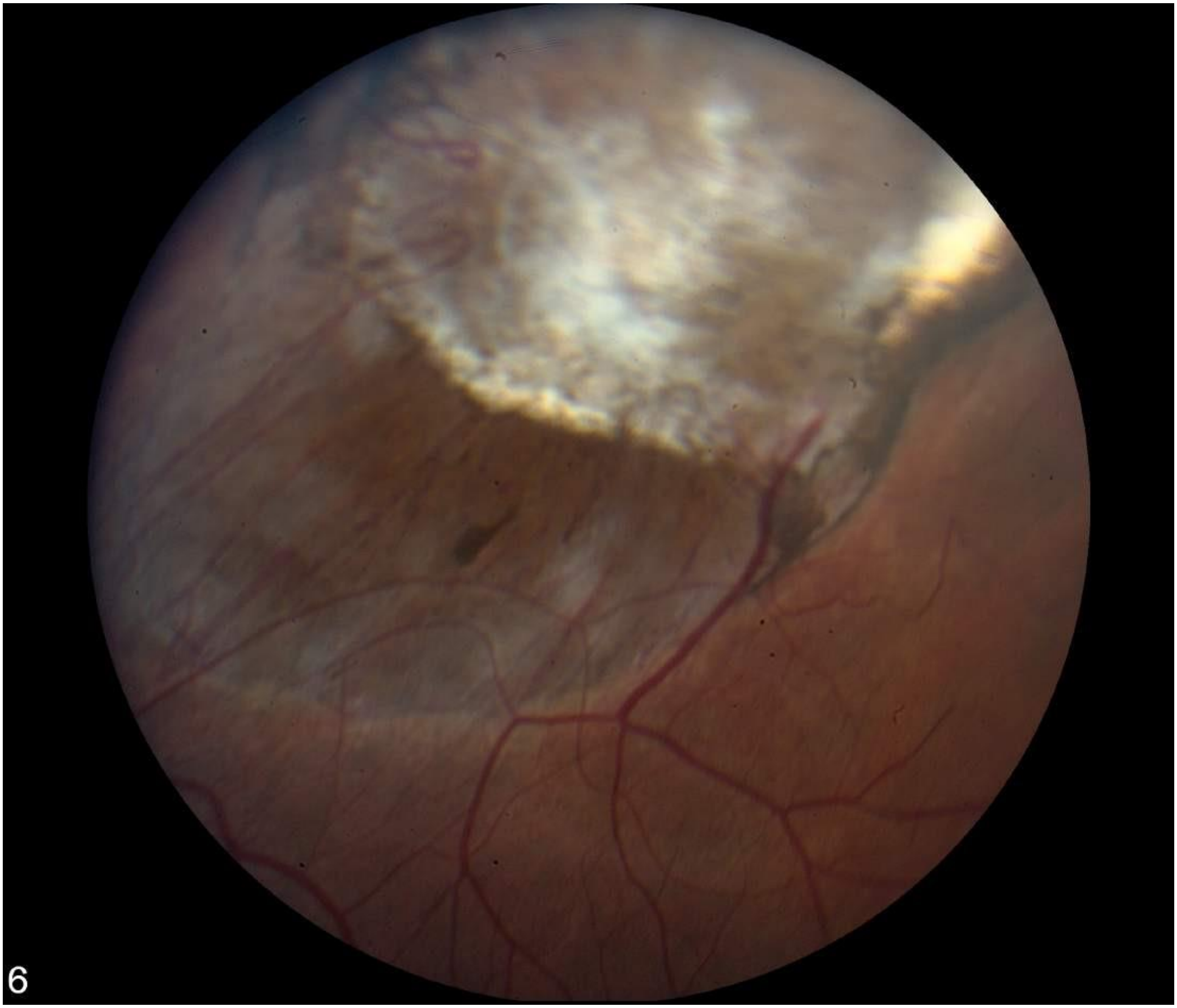

Plate No. 9 – Patient D: Enlargement of peripheral lesion

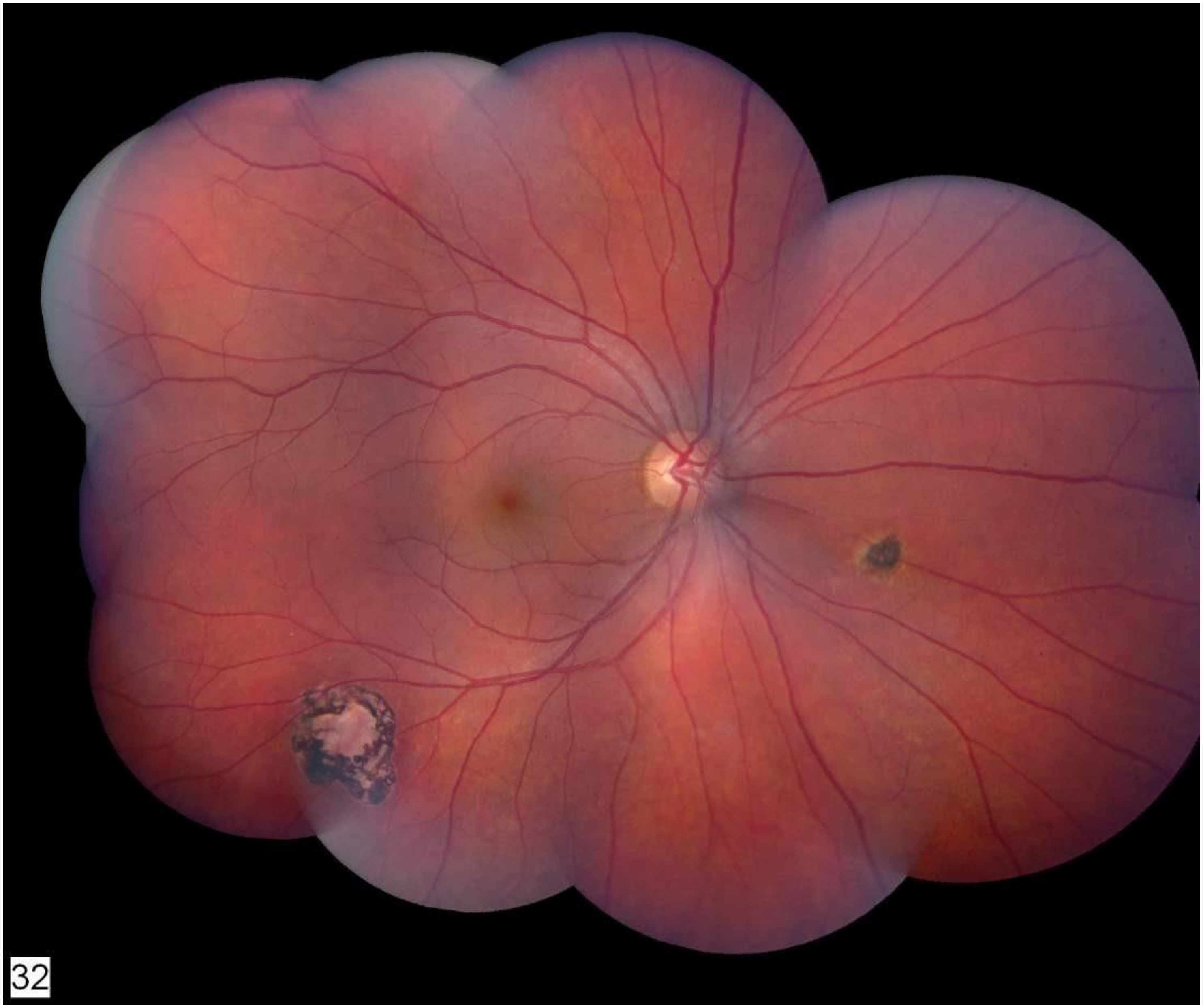

Plate No. 10 – Patient E: Peripheral lesions following retinal vessels, first lesion is small, round, hyperpigmented. Second lesion is moderately small with irregular border, hypopigmented center.

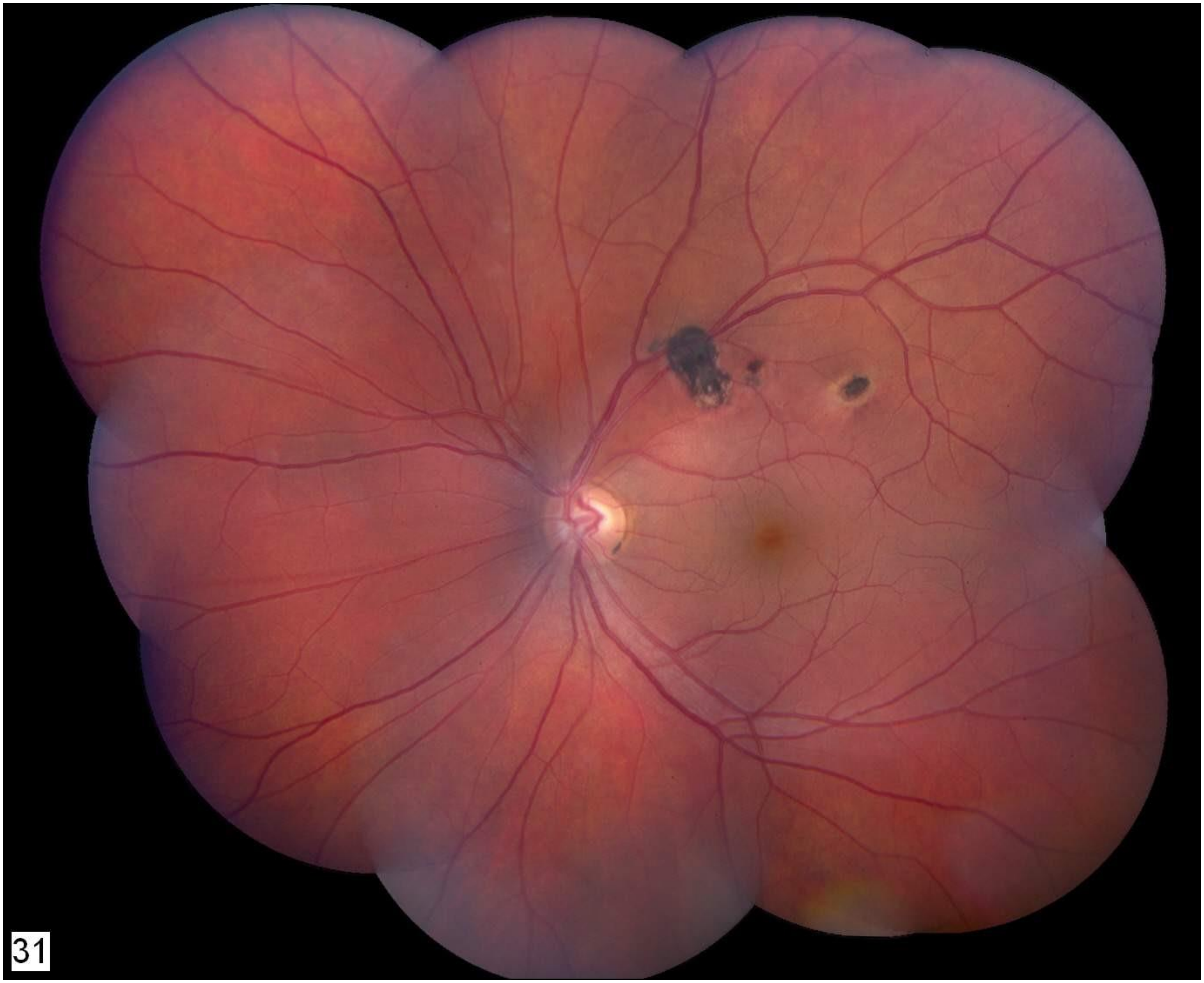

Plate No. 11 – Patient E: Moderately large, characteristic toxoplasmosis chorioretinal scar with sharply demarcated margins, encompassing the fovea in the central macula.

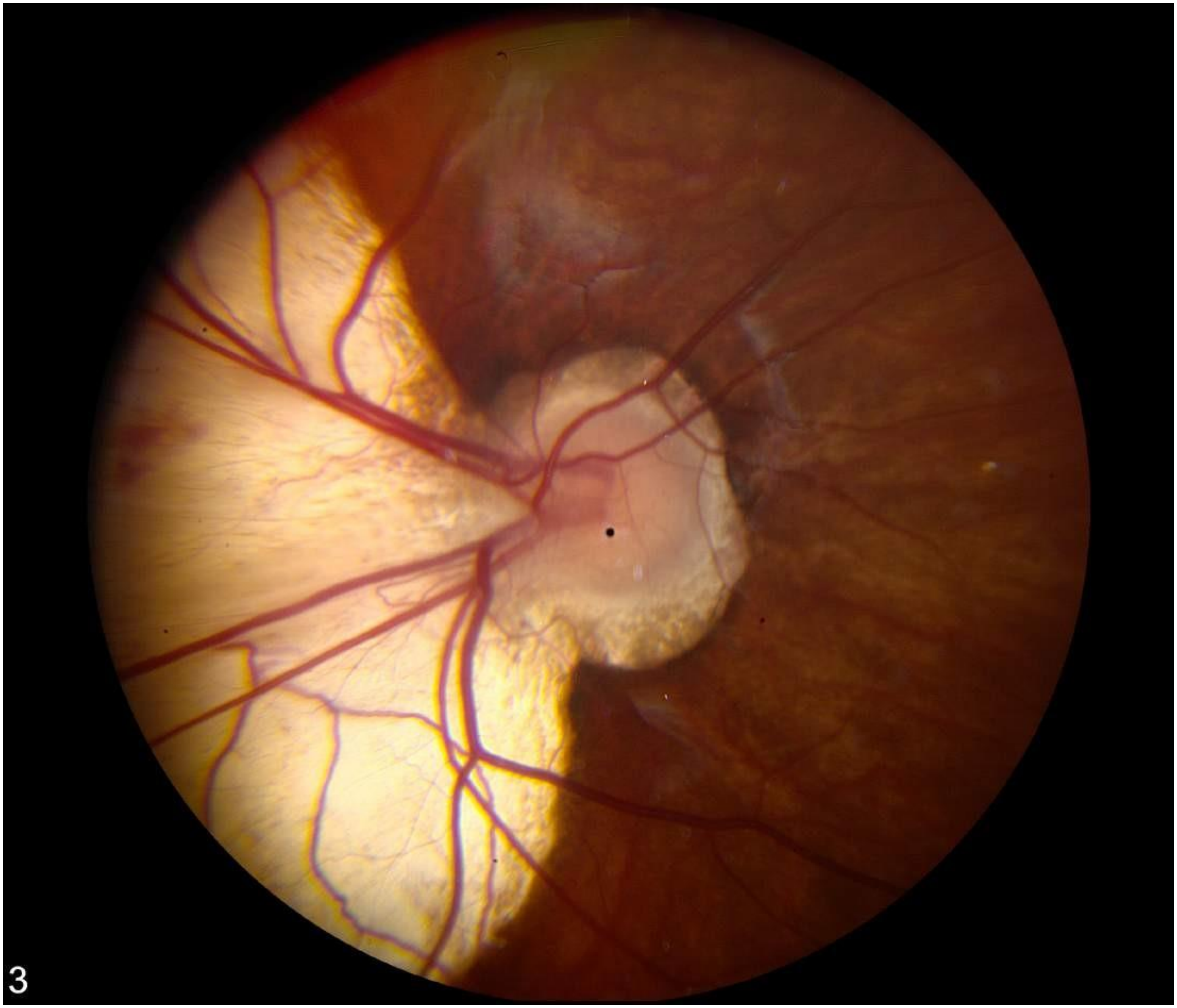

Plate No. 12- Patient F- Abnormal optic nerve due to large peripapillary chorioretinal lesion. Optic nerve is tilted, elongated, and there is no identifiable central cup or neural rim. Lesion extends from nasal side of the optic nerve temporally through the entire macula to approximately mid periphery.

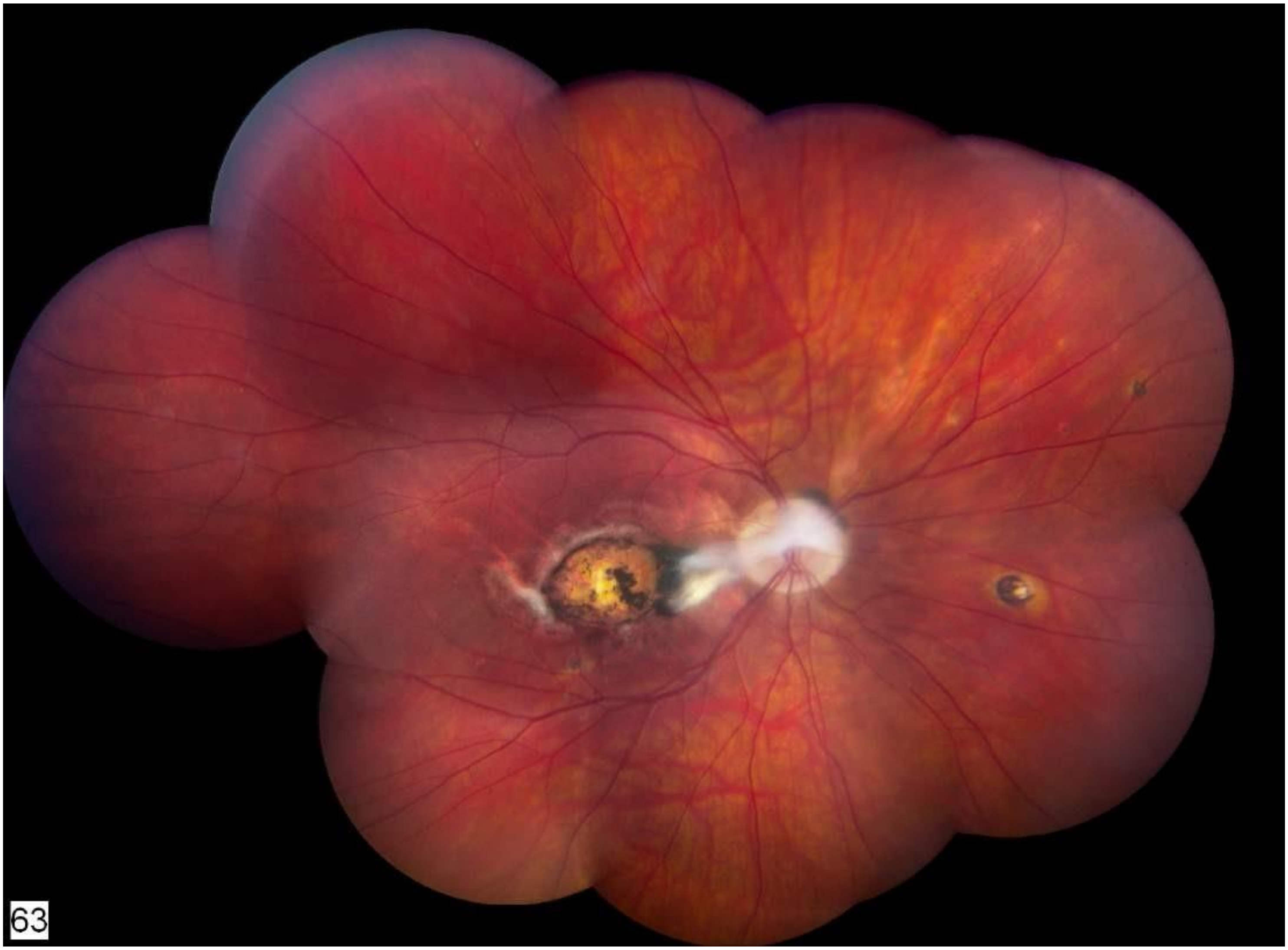

Plate No. 13- Patient G- Fibrovascular proliferation band over optic nerve. The vessels are normal. In the macula there is a large, deep chorioretinal scar, approximately one disc diameter, with mixed hypopigment and hyperpigment centrally, surrounded by hyperpigment peripherally, with a hypopigmented sharp margin superiorly. This chorioretinal lesion encompasses the fovea. There is a small, elliptical-shaped area of white fibrosis extending superior temporally from the lateral margin of the central chorioretinal lesion.

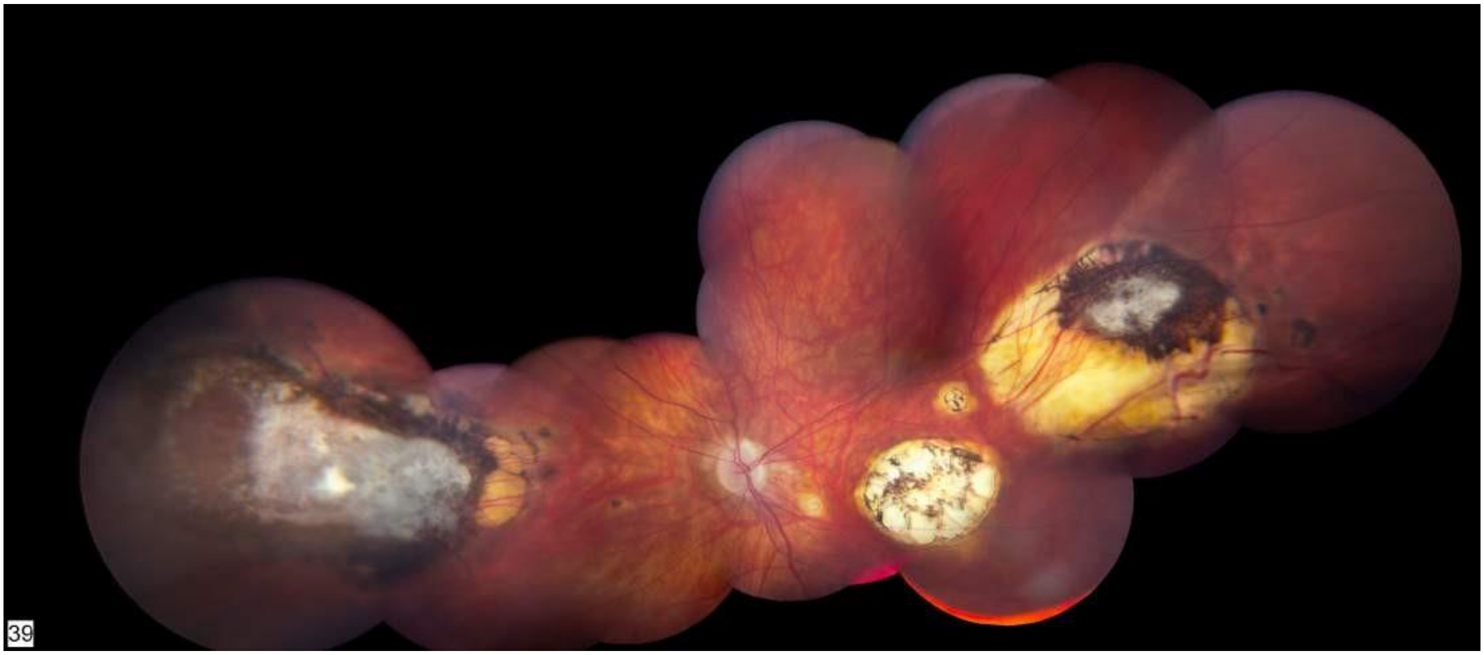

Plate No. 14- Patient G- The optic nerve has mild pallor (+1); the temporal neural rim has more pallor than the nasal rim. The margins are sharp and there is mildly pigmented peripapillary ring. The superior and inferior temporal vascular arcades are straightened over the large chorioretinal scar in the macula. The superior temporal vascular arcade runs through the large peripheral chorioretinal scar superior temporally.

In the macula, there is a small, 0.25 disc diameter, hypopigmented lesion inferior temporal to the optic nerve between the optic nerve and the large central macular lesion. The margins of this lesion are less distinct than the other lesions. There is a large, round chorioretinal scar in the center of the macula which encompasses the fovea. It is approximately 2.5 disc diameters in size and it has scattered hyperpigment outlining lacunae; the lacunae are deeply excavated and are mostly white. This central large lesion is surrounded by a thin, sharp hypopigmented margin. There is a third small chorioretinal lesion in the macula located superior to the large central lesion, just inferior to the superior temporal vascular arcade. This third lesion is moderately small, 0.25 disc diameter in size, round, mostly hypopigmented with scattered hyperpigment surrounded by a sharp margin. These lesions are similar to the previous examinations. Superior temporal to the macula, in the periphery of the 2 o'clock meridian, there is a very large, approximately 4 disc diameters, chorioretinal lesion which is hypopigmented with the choroidal vasculature visible anteriorly. The superior temporal peripheral part of this lesion has an area approximately 2 disc diameter in size which is deeply excavated, fibrosed, white centrally surrounded by a thick margin of deep hyperpigment. This lesion is similar to the previous examination. There are 3 hyperpigmented, small chorioretinal lesions peripheral to this very large superior temporal lesion. Superior nasal to the optic nerve in the far periphery of the 10 o'clock meridian there is another very large chorioretinal lesion, approximately 5 disc diameters horizontally by 3 disc diameters vertically. The majority of the lesion is deeply excavated, fibrosed and white surrounded by a thick, deeply hyperpigmented rim. The anterior leading edge of this lesion is hypopigmented with the choroidal vasculature visible; there is scattered hyperpigment along the margin. There is a group of 3 chorioretinal lesions just anterior to the very large superior nasal lesion in the periphery of the nasal quadrant. These chorioretinal lesions are small, 0.1 disc diameter and hyperpigmented. The most superior lesion is round and is surrounded by hypopigment. The lesion closest to the larger chorioretinal lesion is curvilinear and is composed of several (approximately 7) contiguous, smaller round lesions. The third lesion is linear, is perpendicular to the larger lesion.

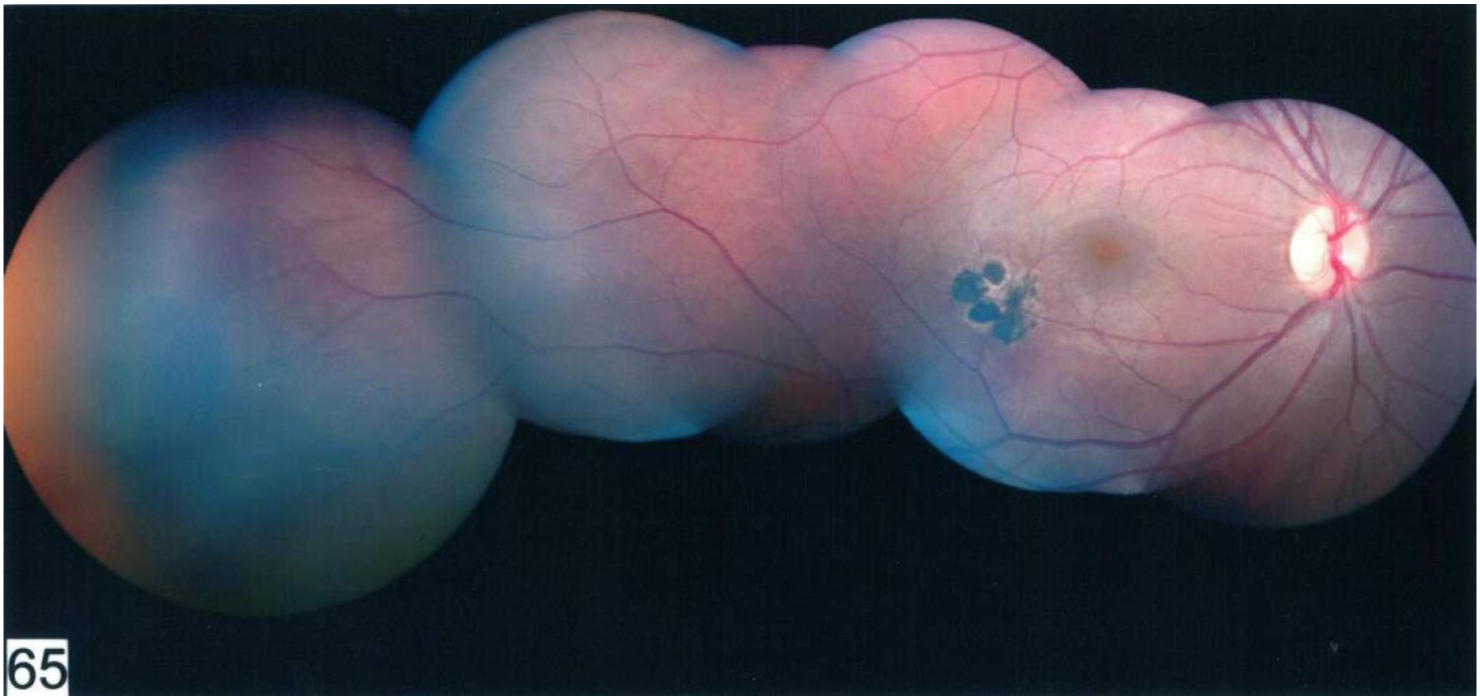

Plate No. 15– Patient H

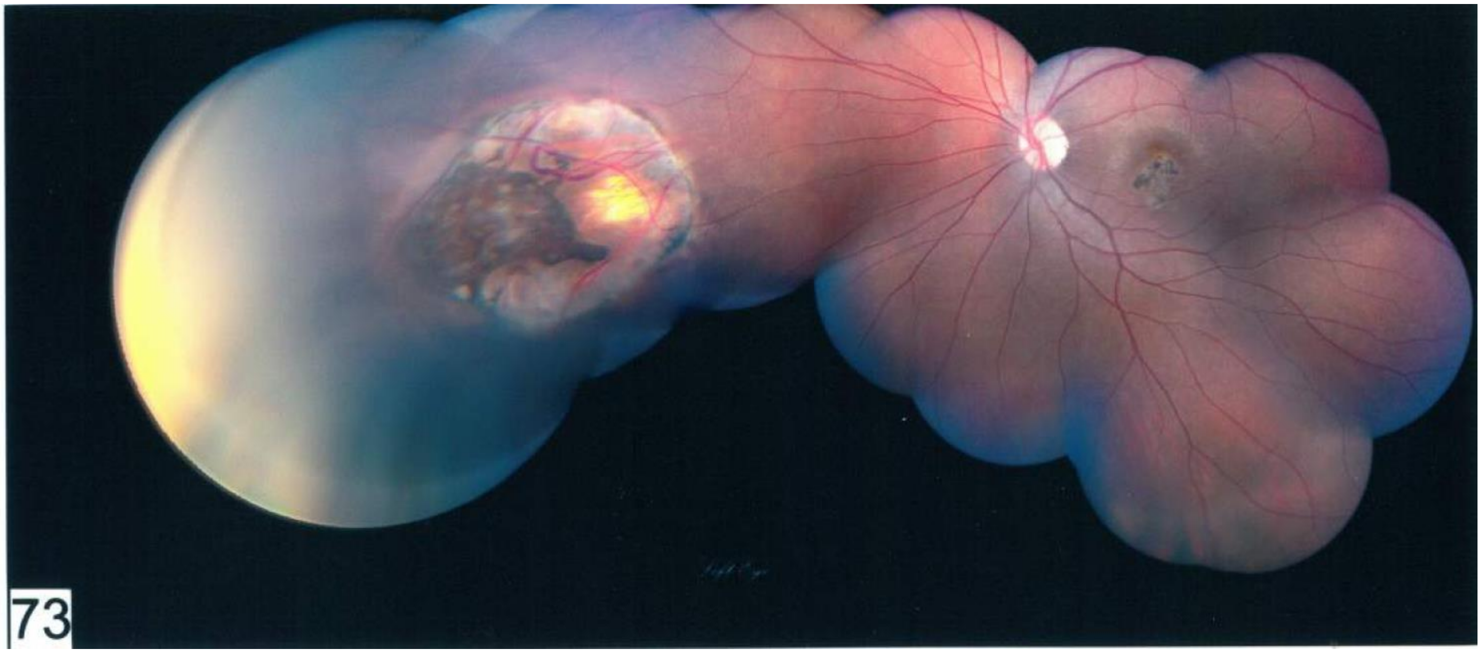

Plate No. 16-Patient H

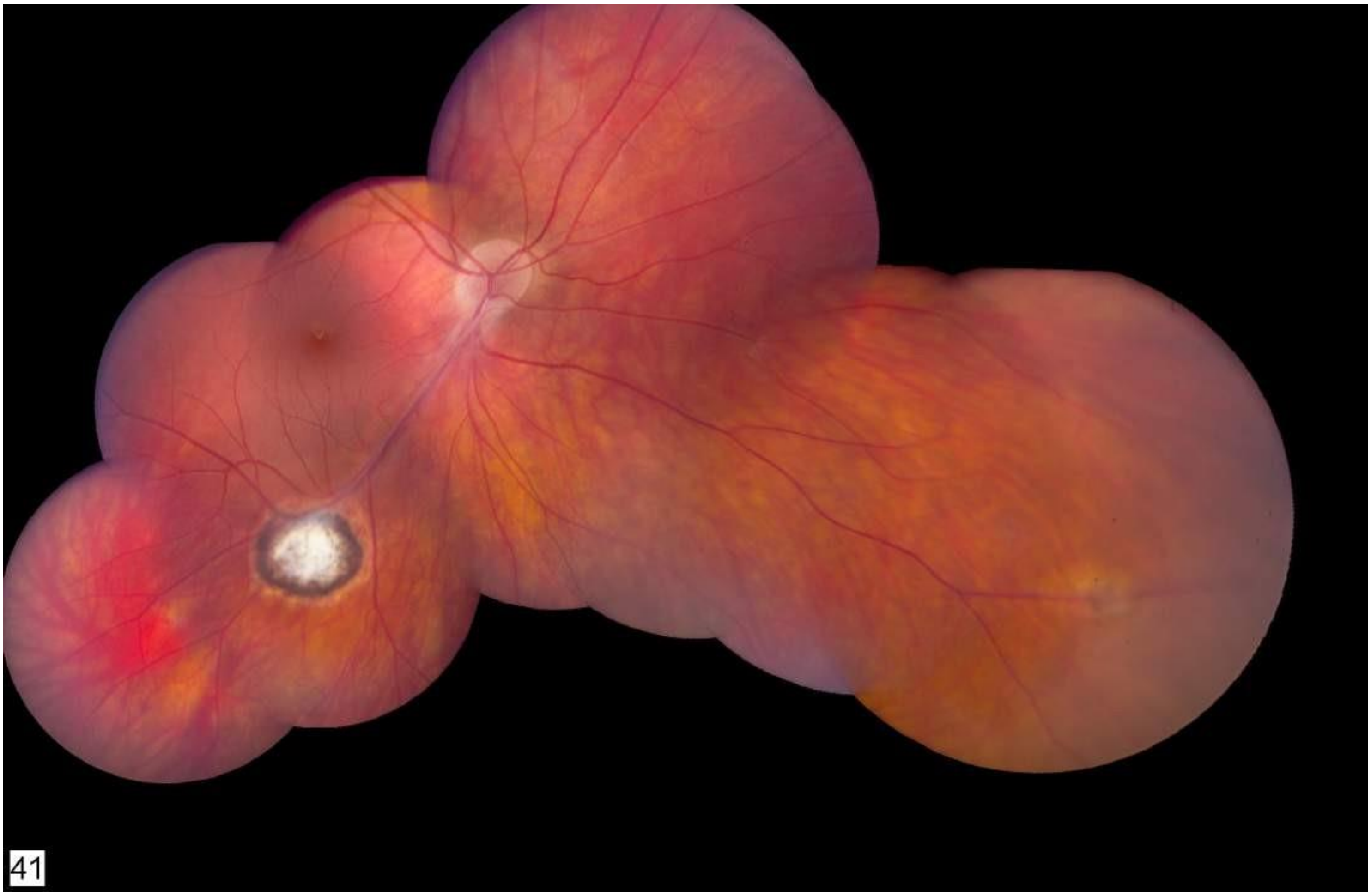

Plate No. 17 – Patient I - Fibrovascular proliferation?

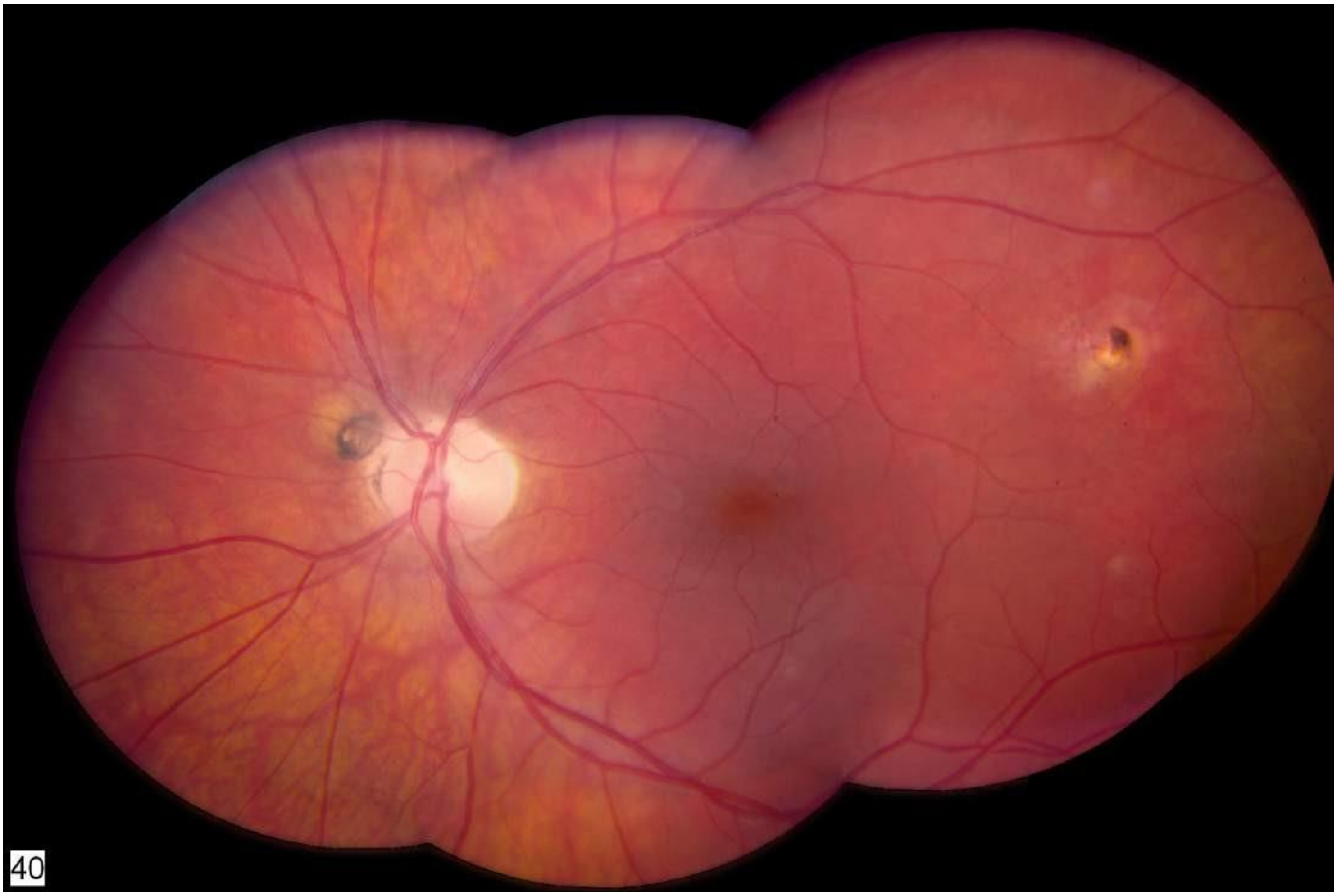

Plate No. 18 – Patient I

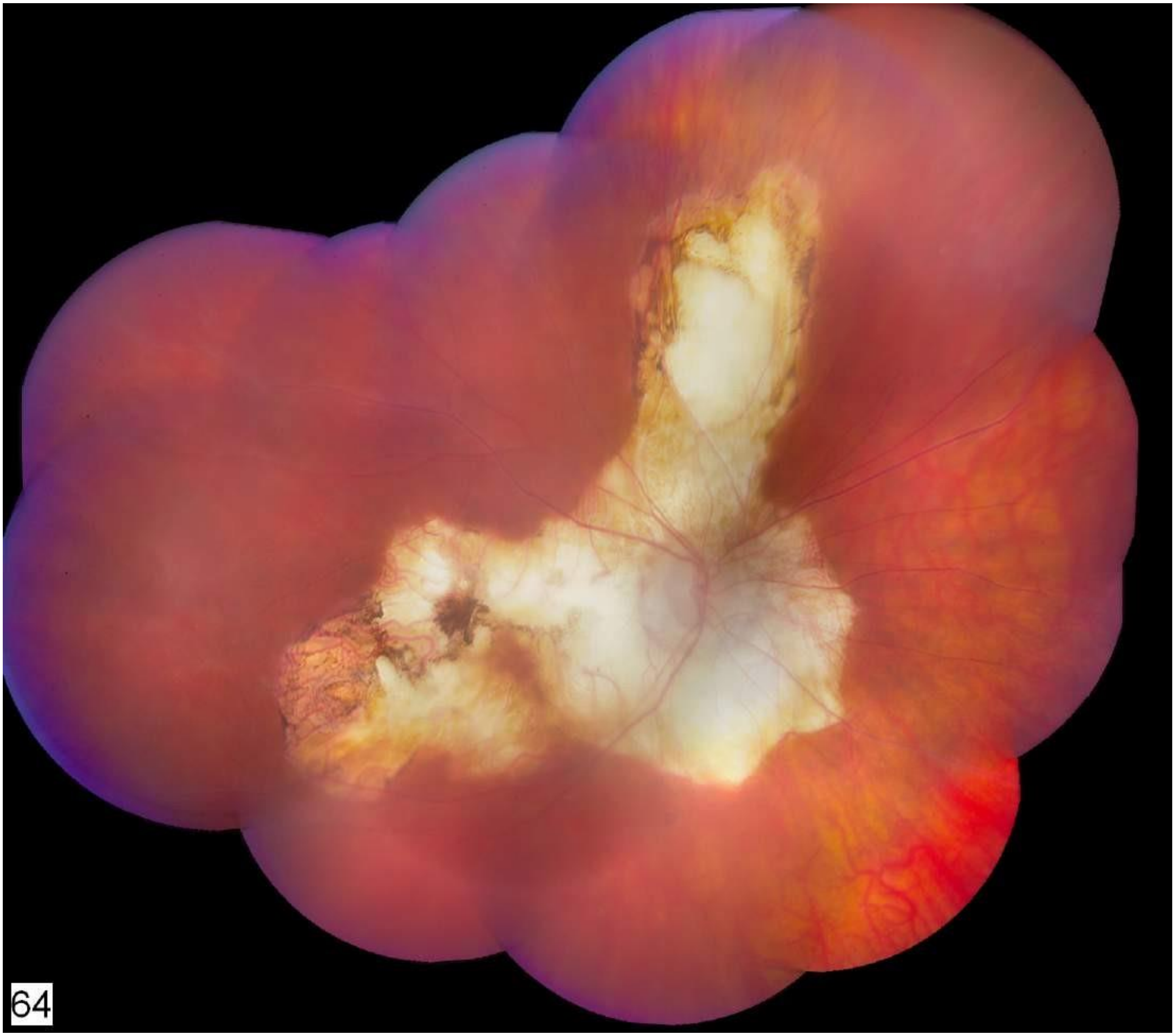

Plate No. 19 – Patient J

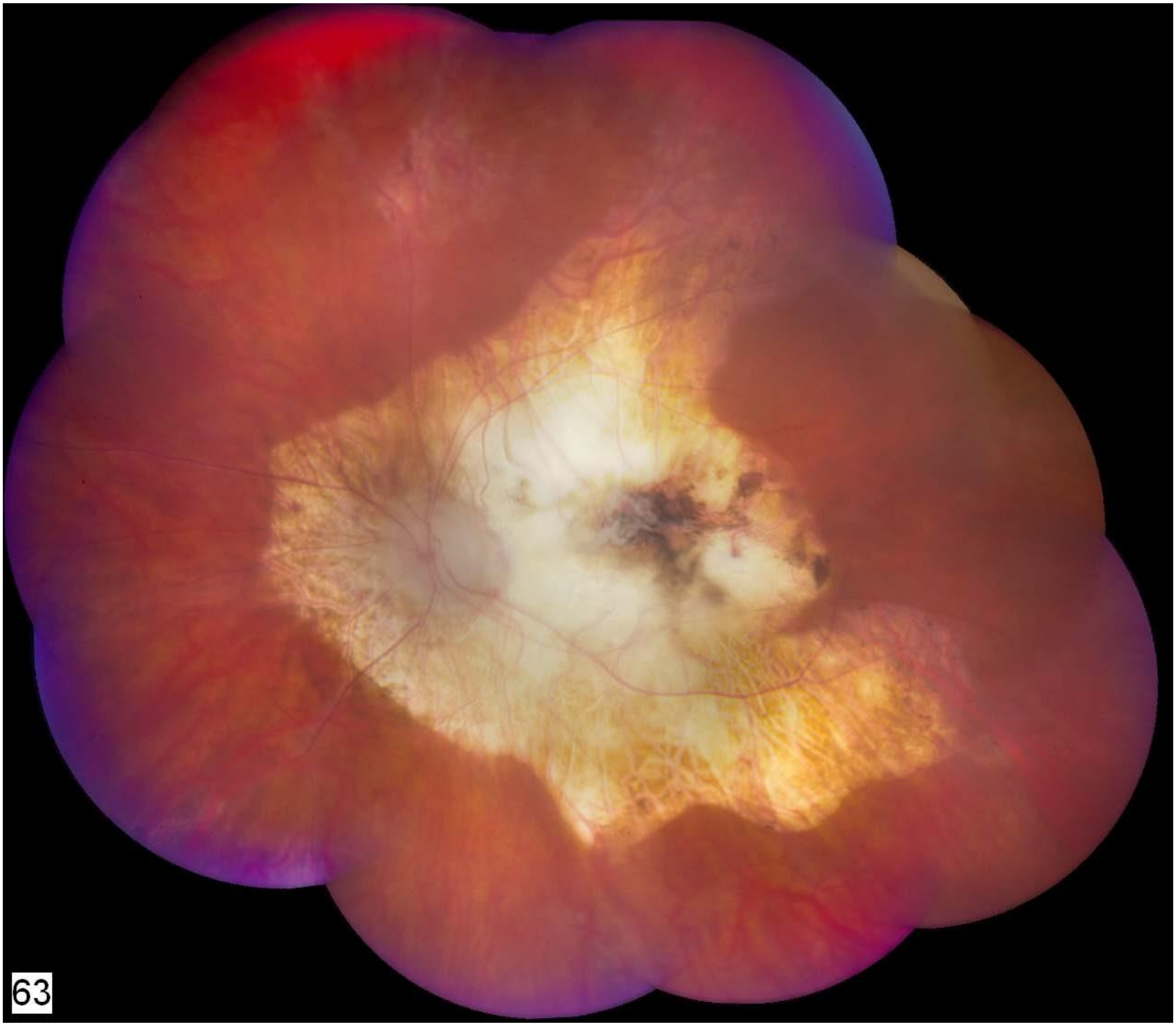

Plate No. 20 – Patient J

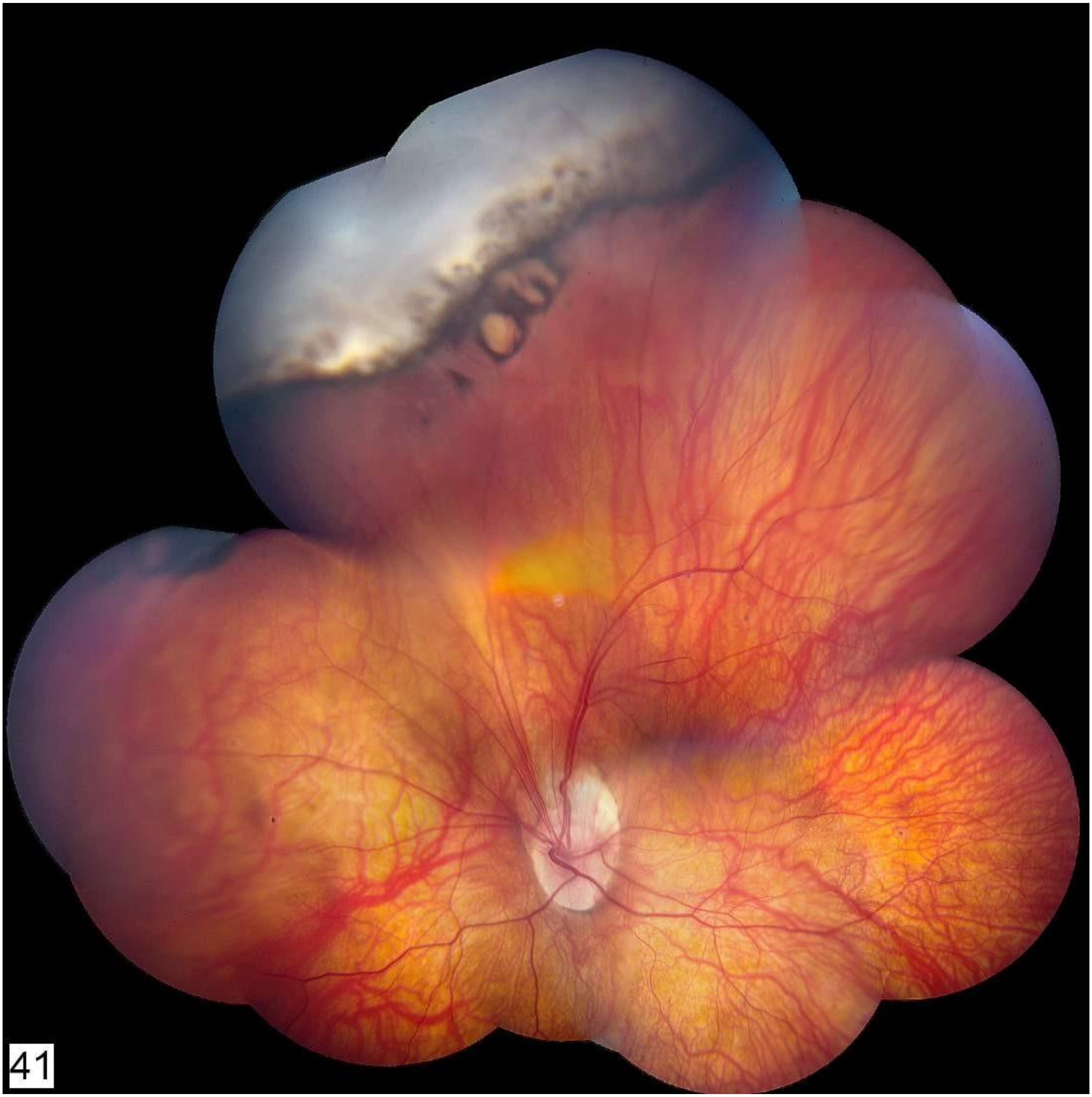

Plate No. 21 – Patient K

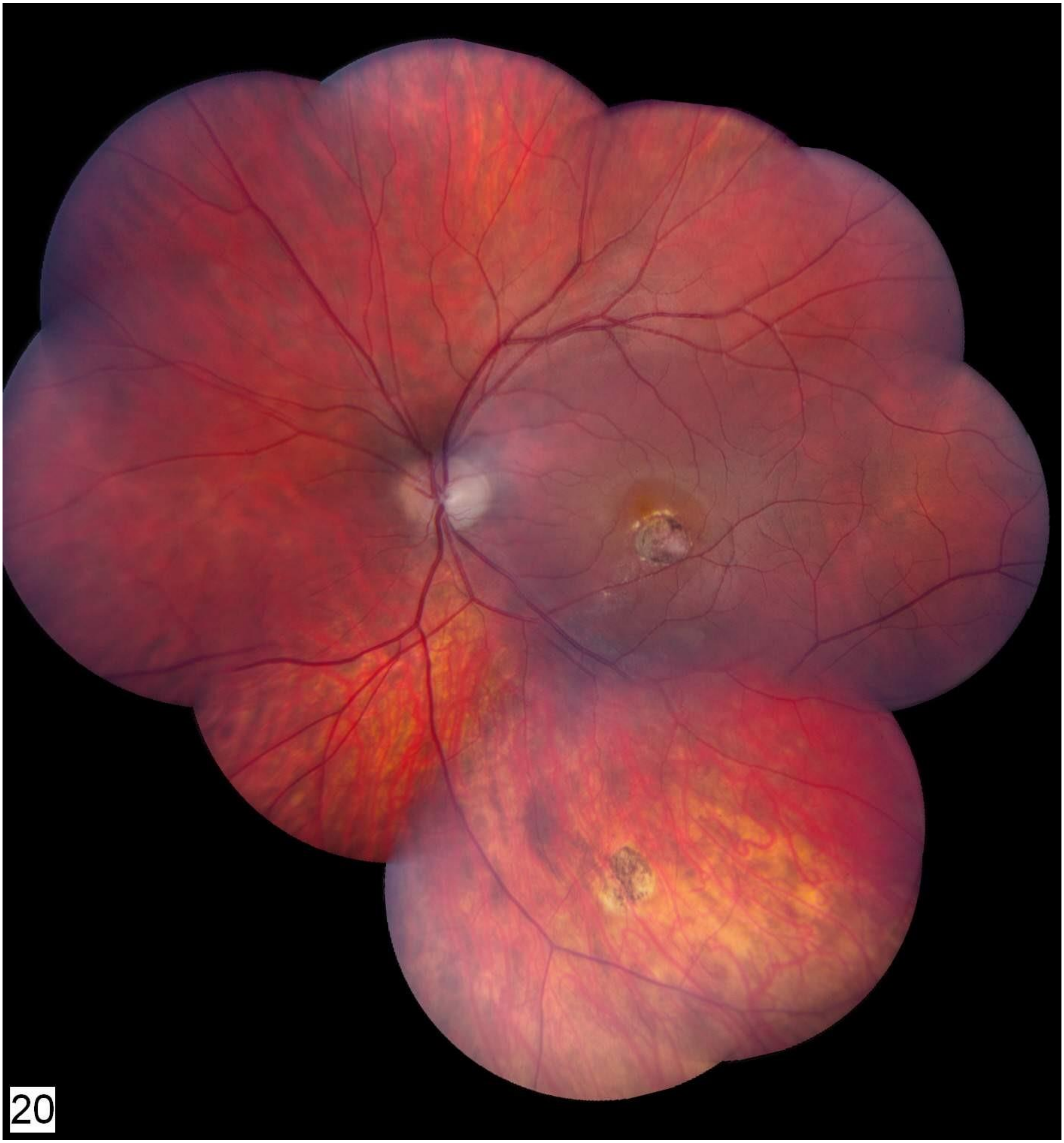
20

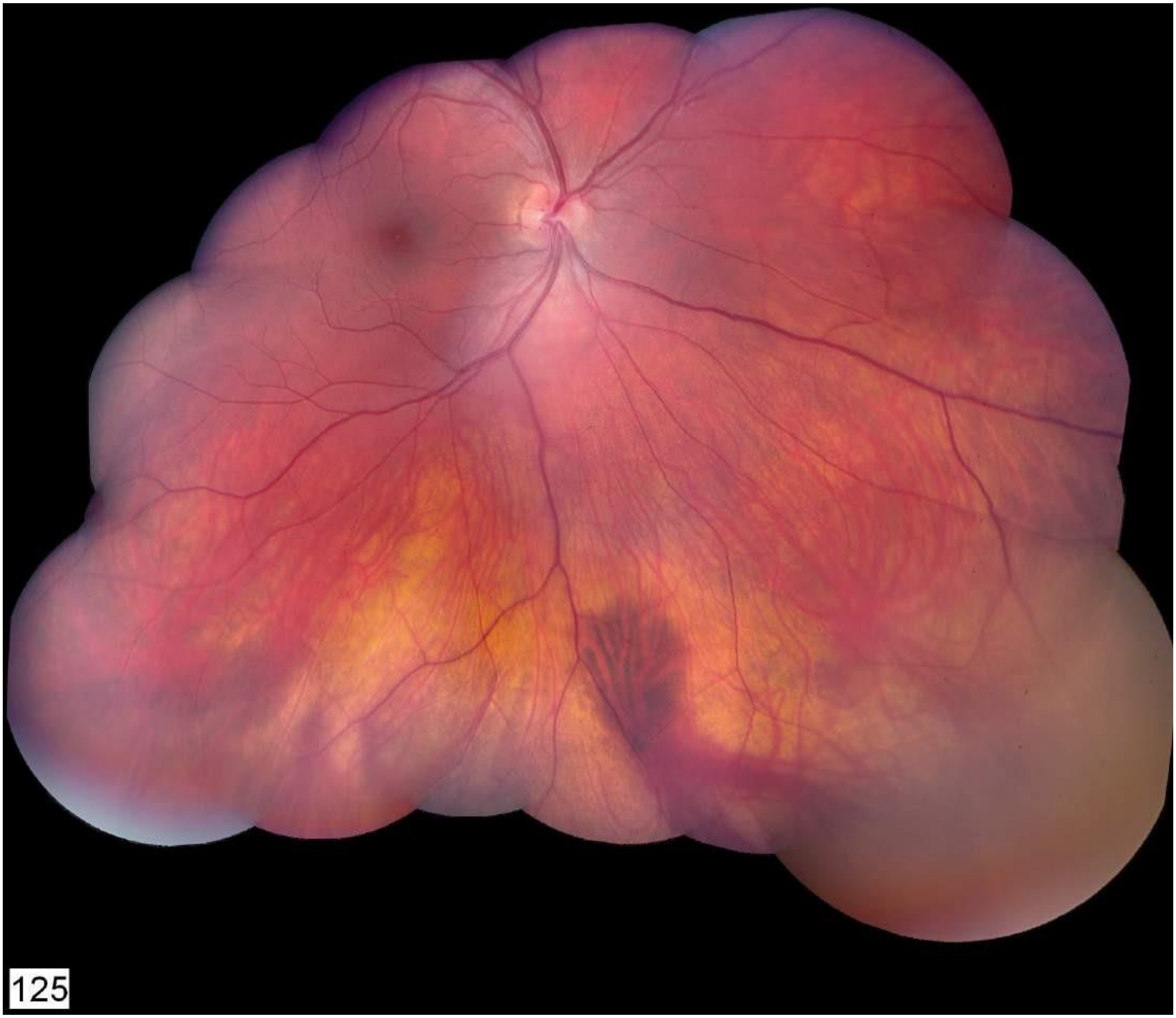

Patient L

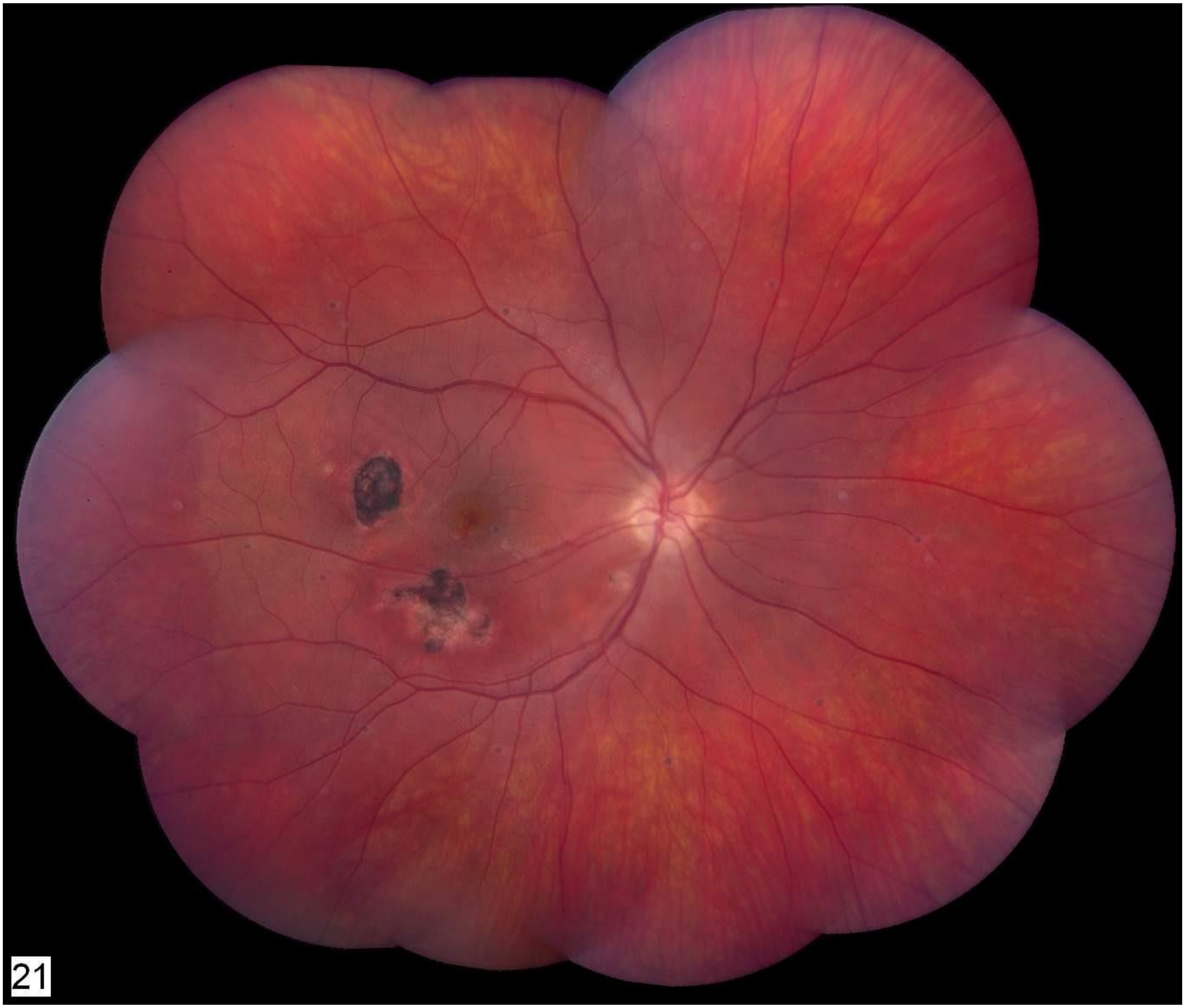

Patient M

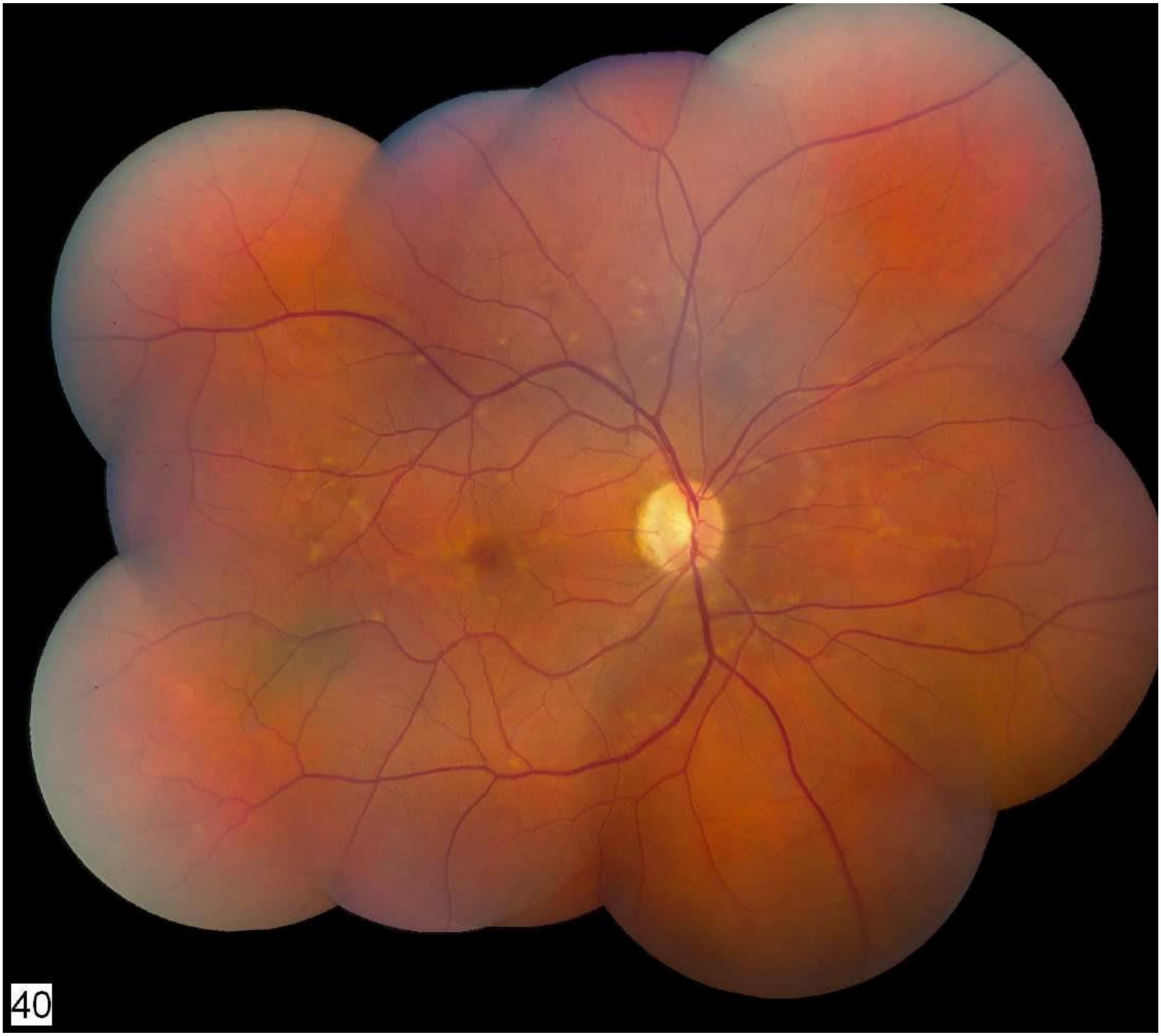

Patient N

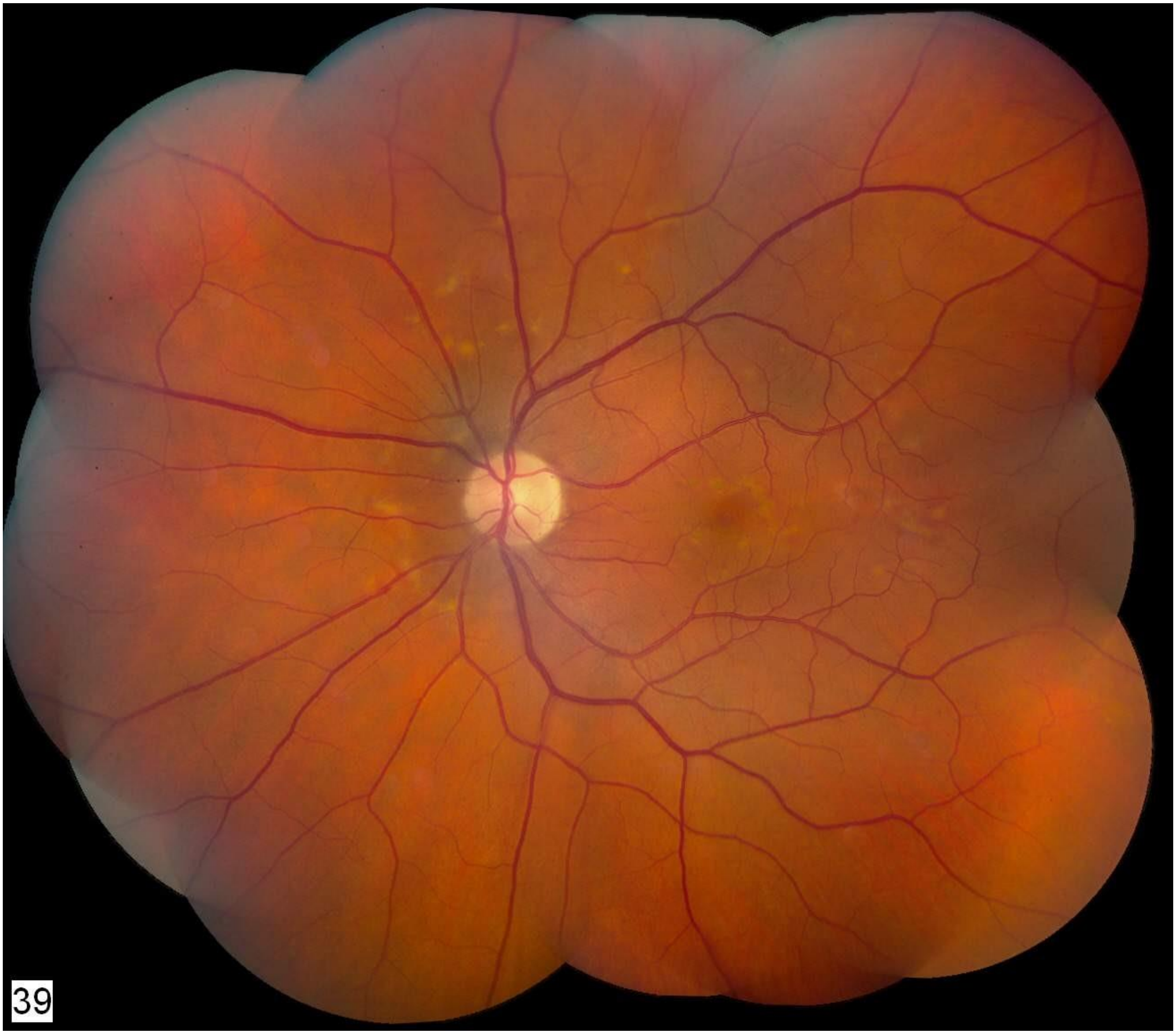

Patient N

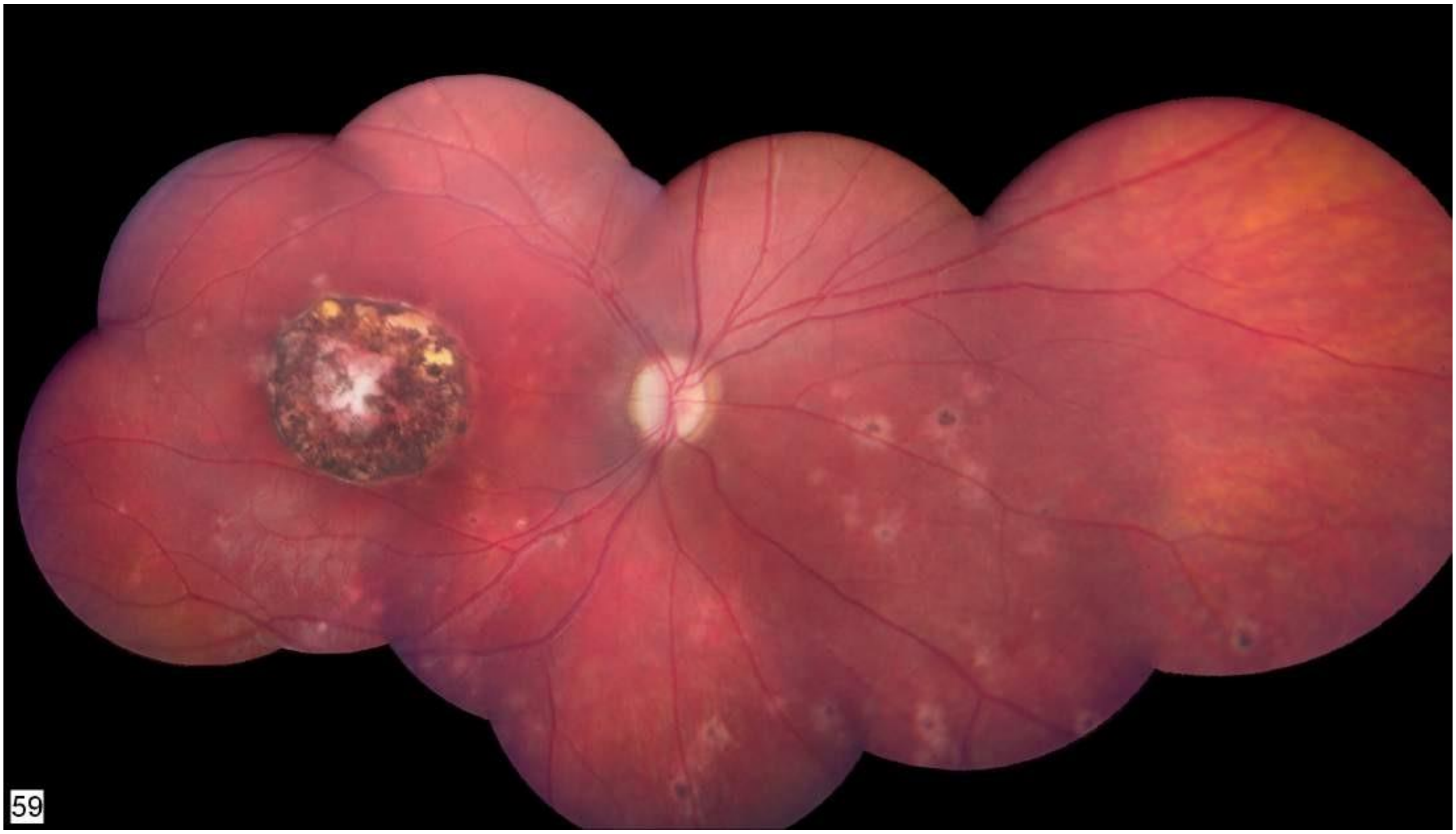

Patient O

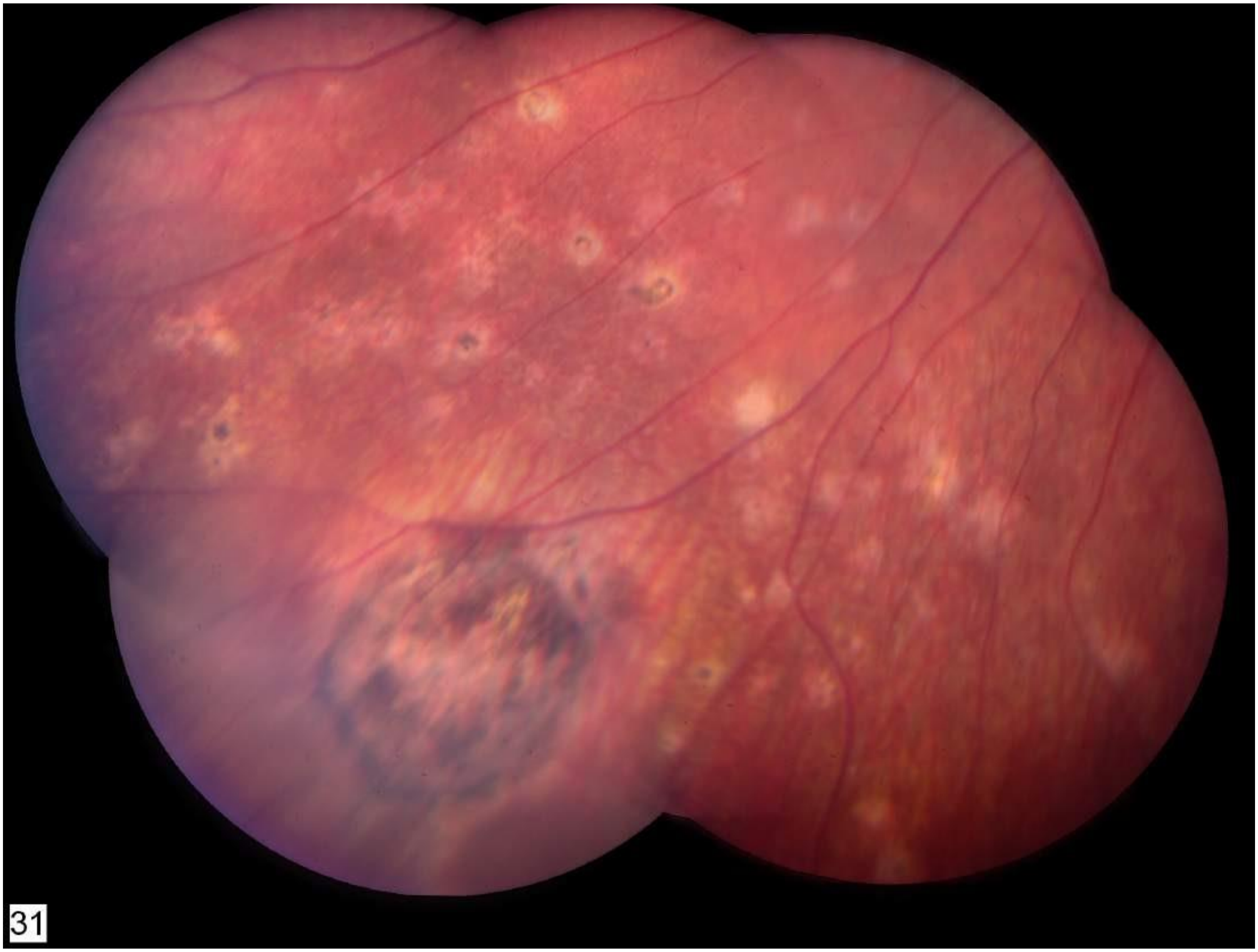

Patient O

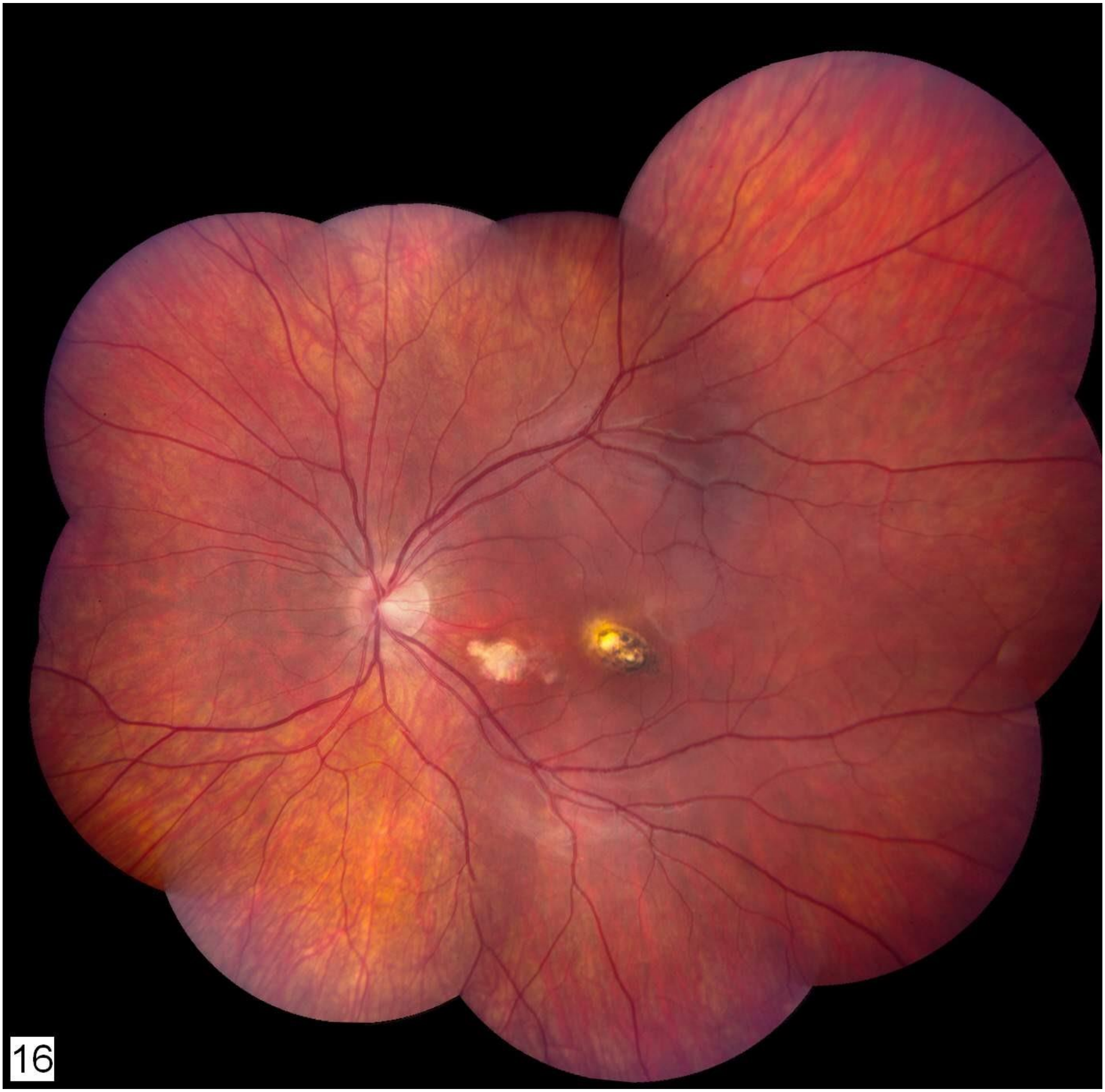

Patient P

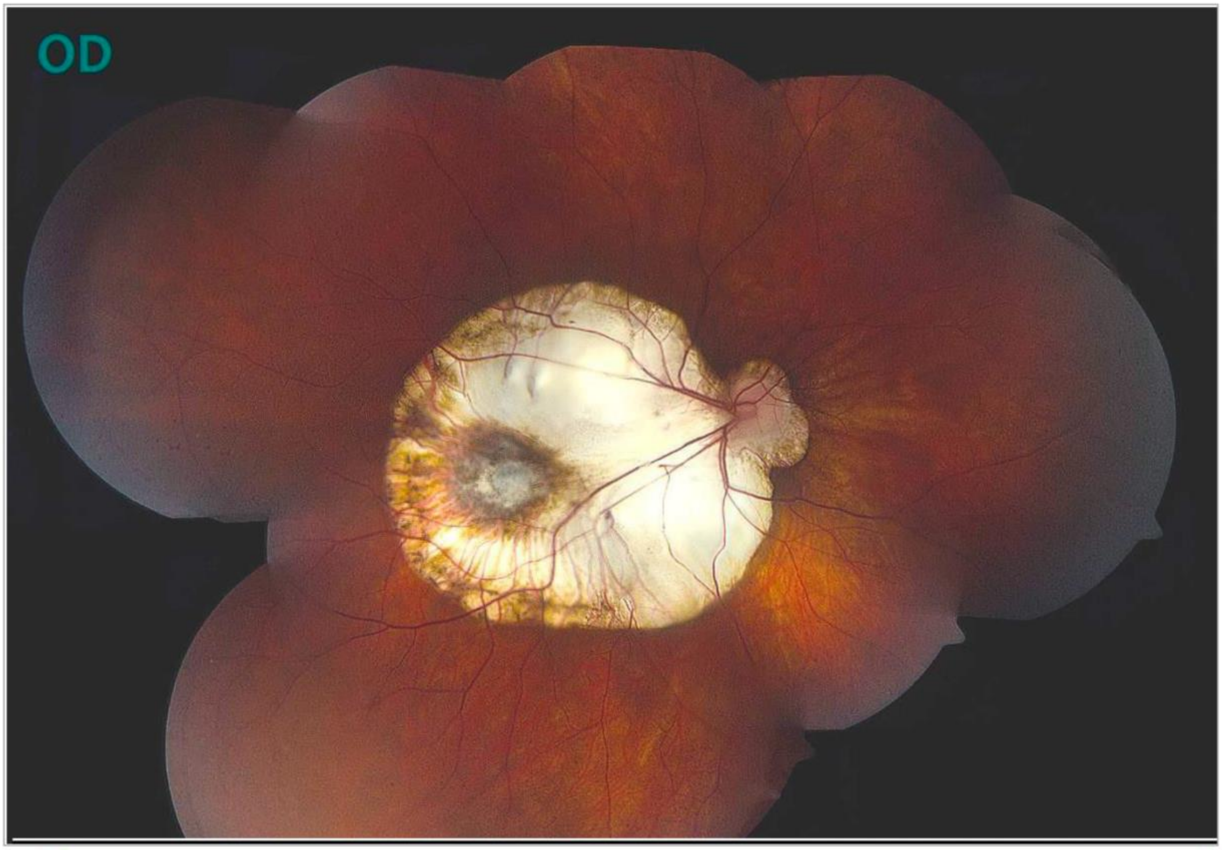
OD

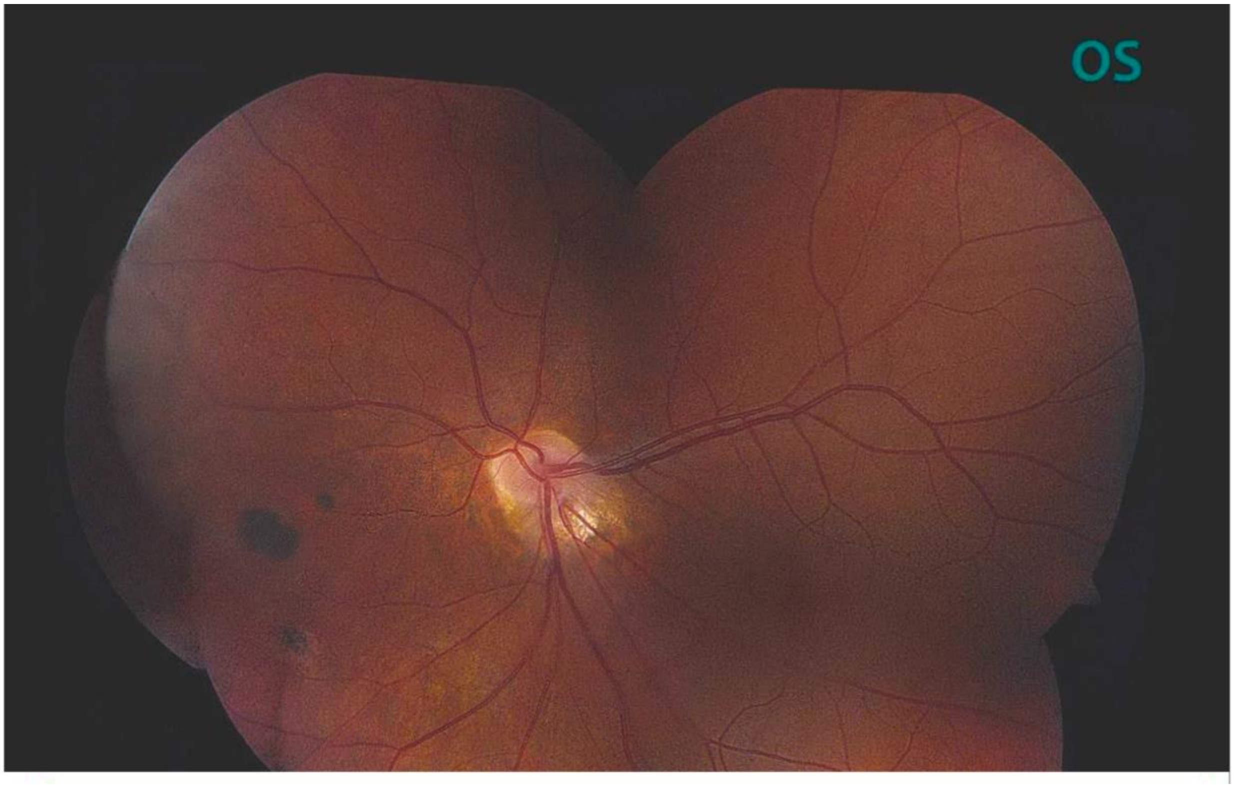
OS

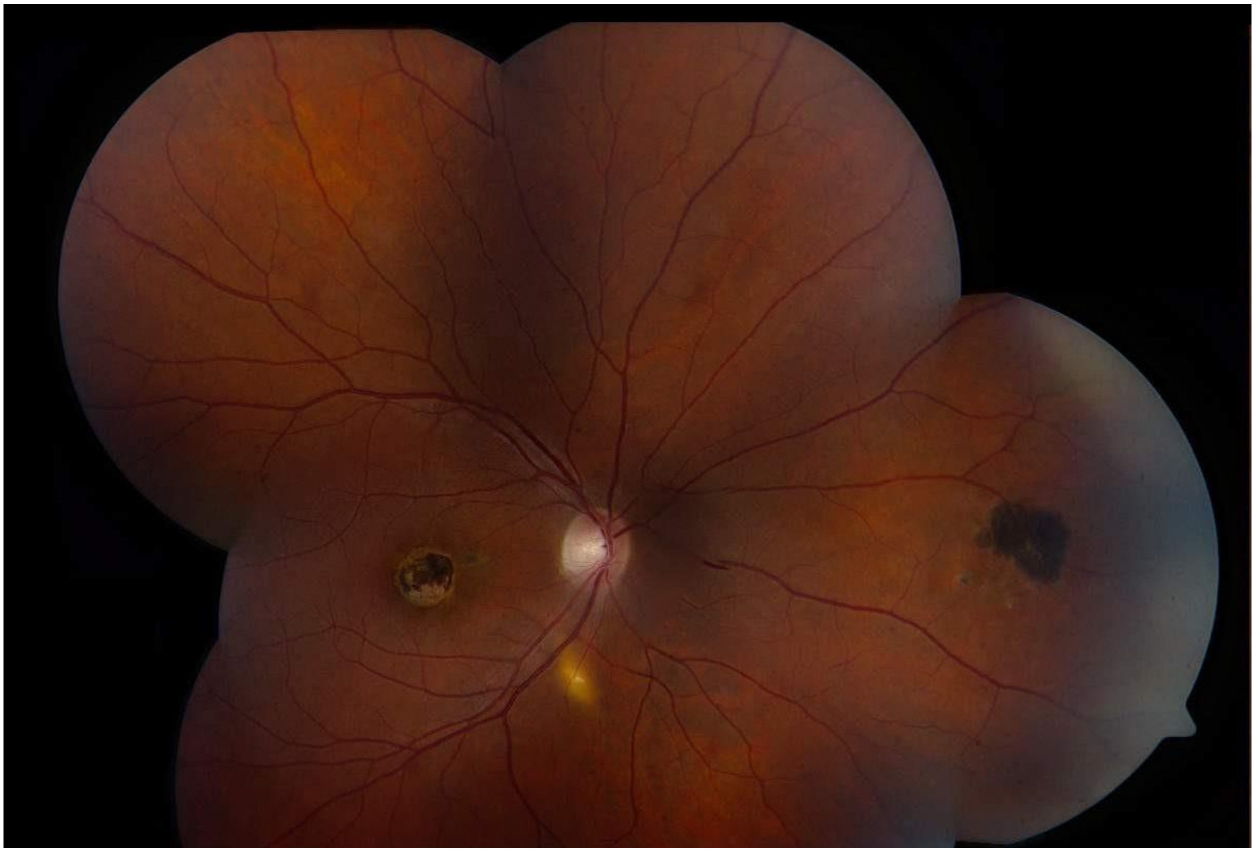

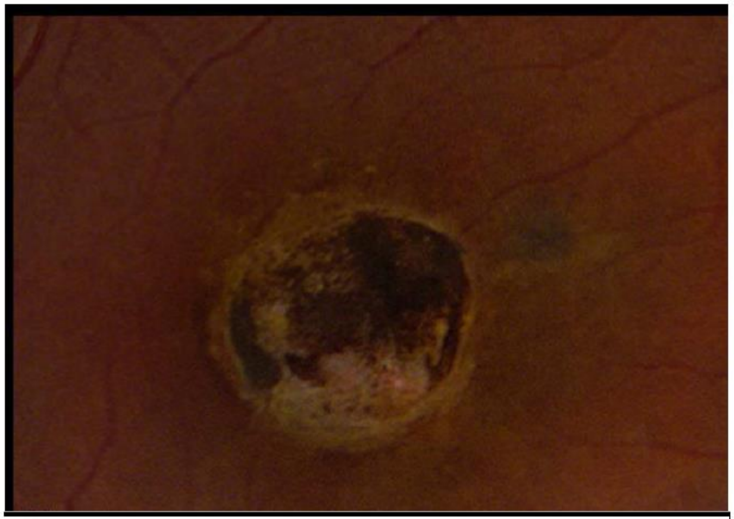

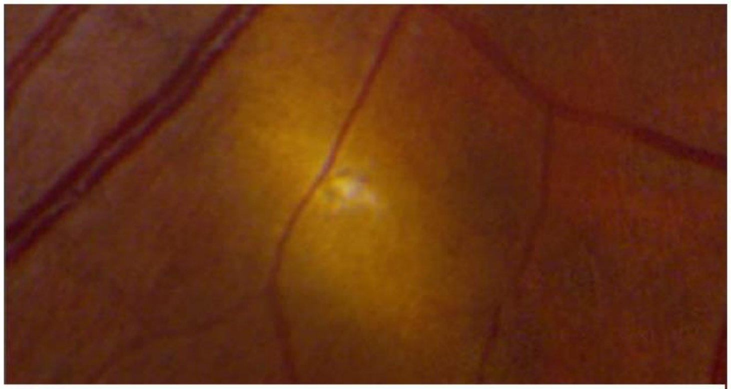

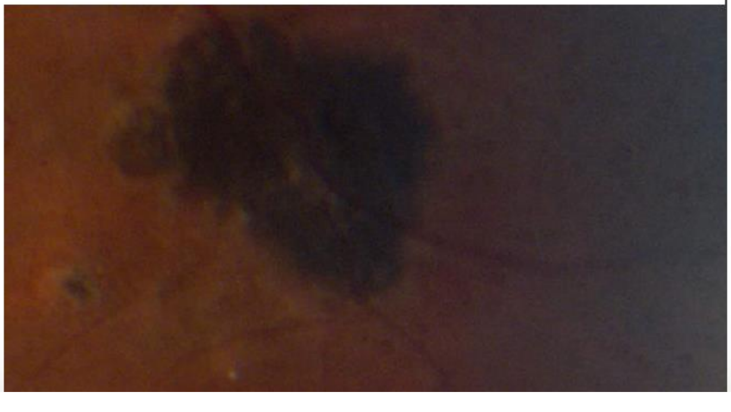

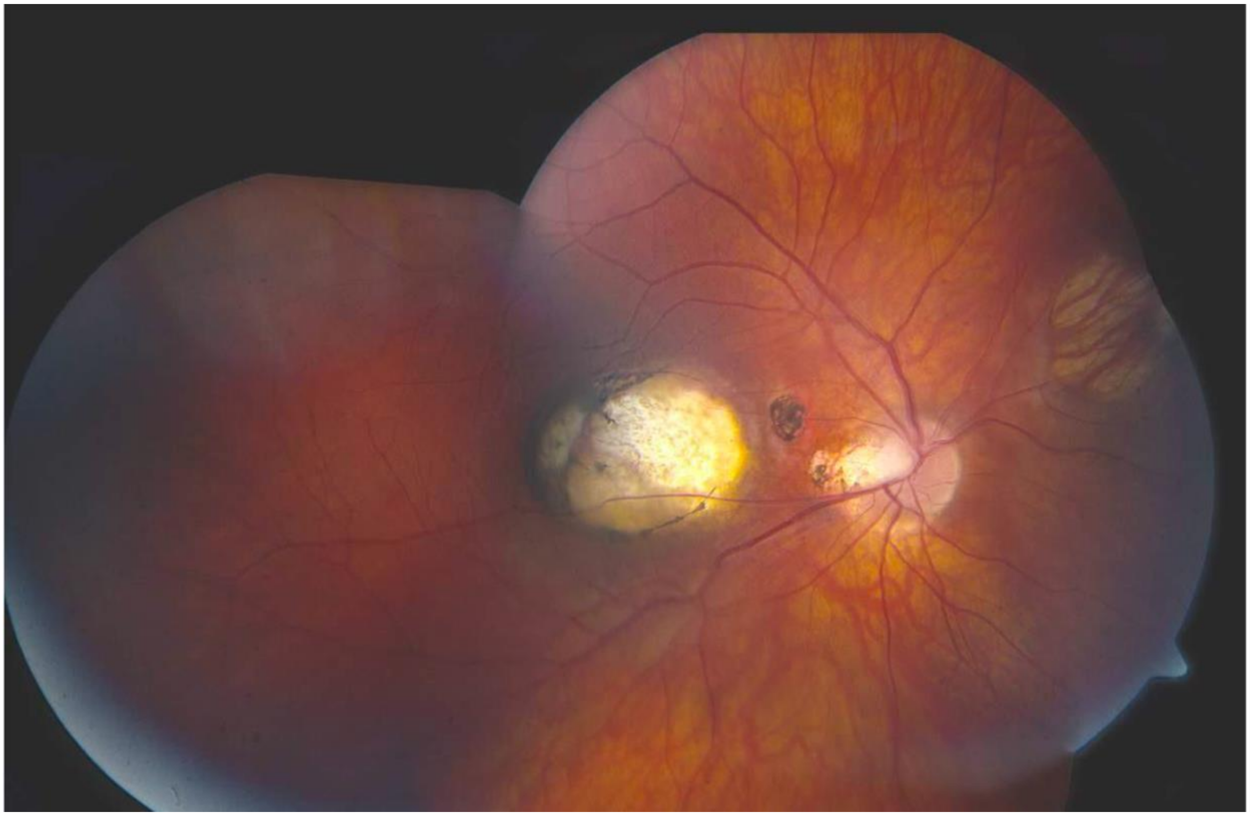

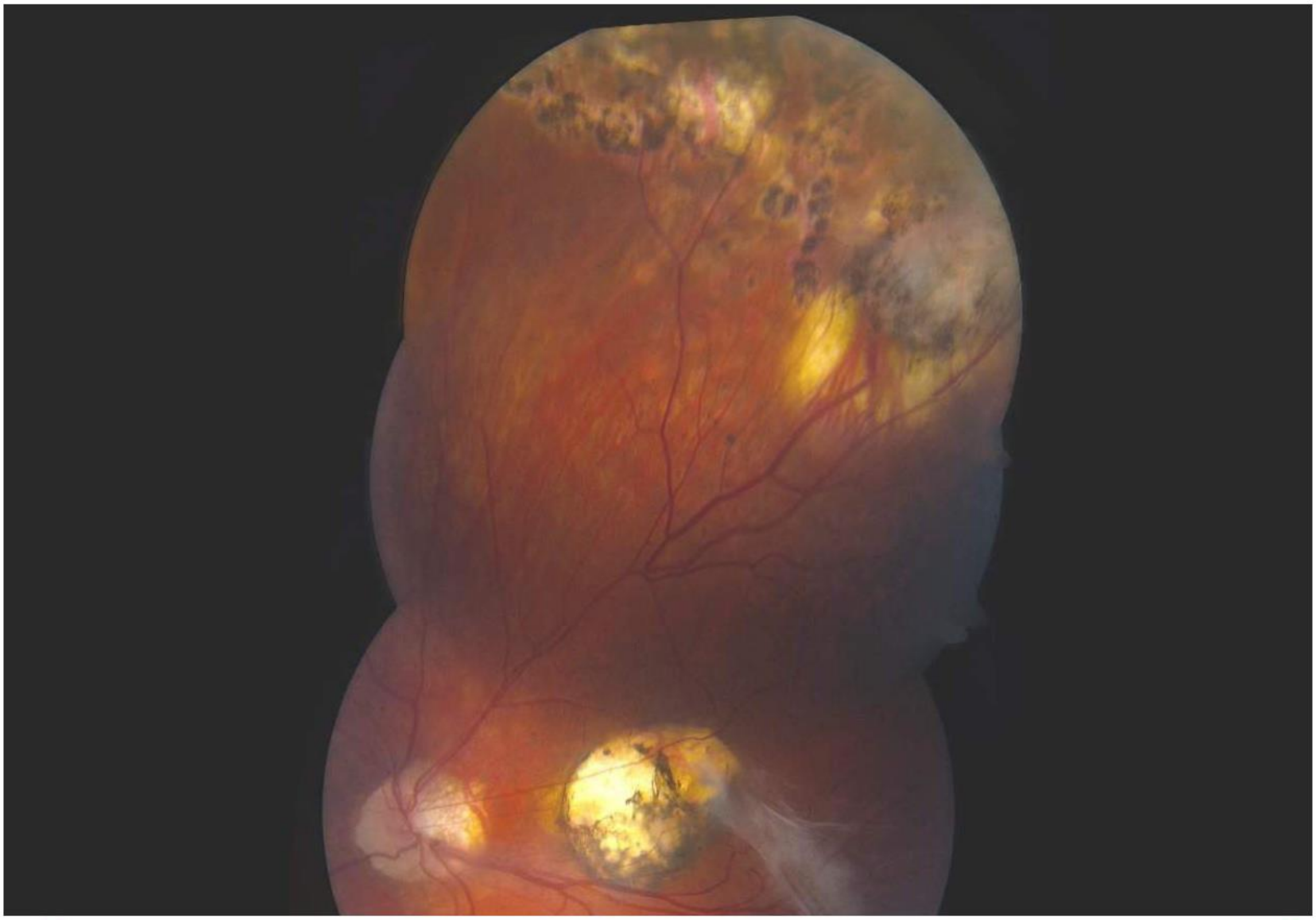

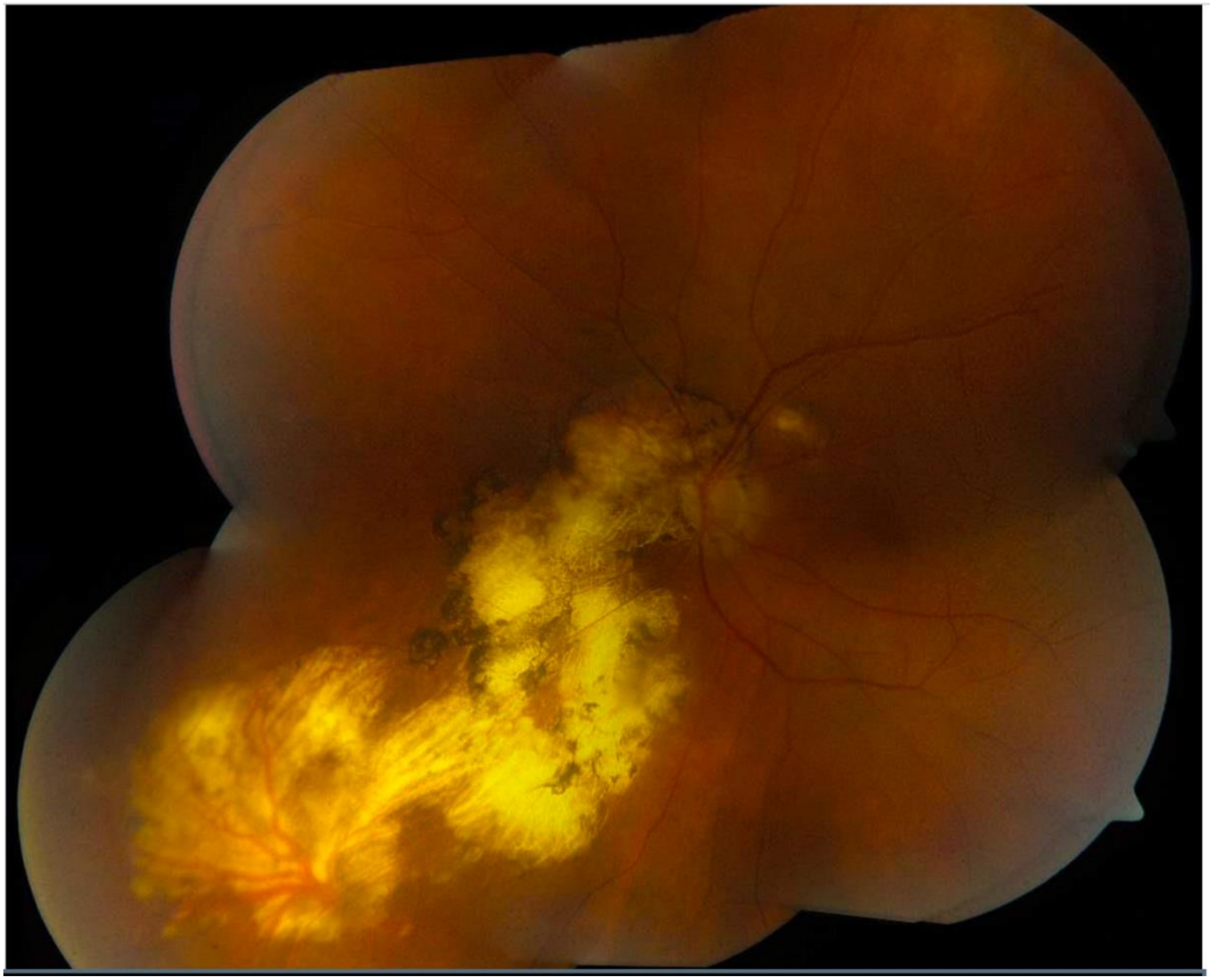

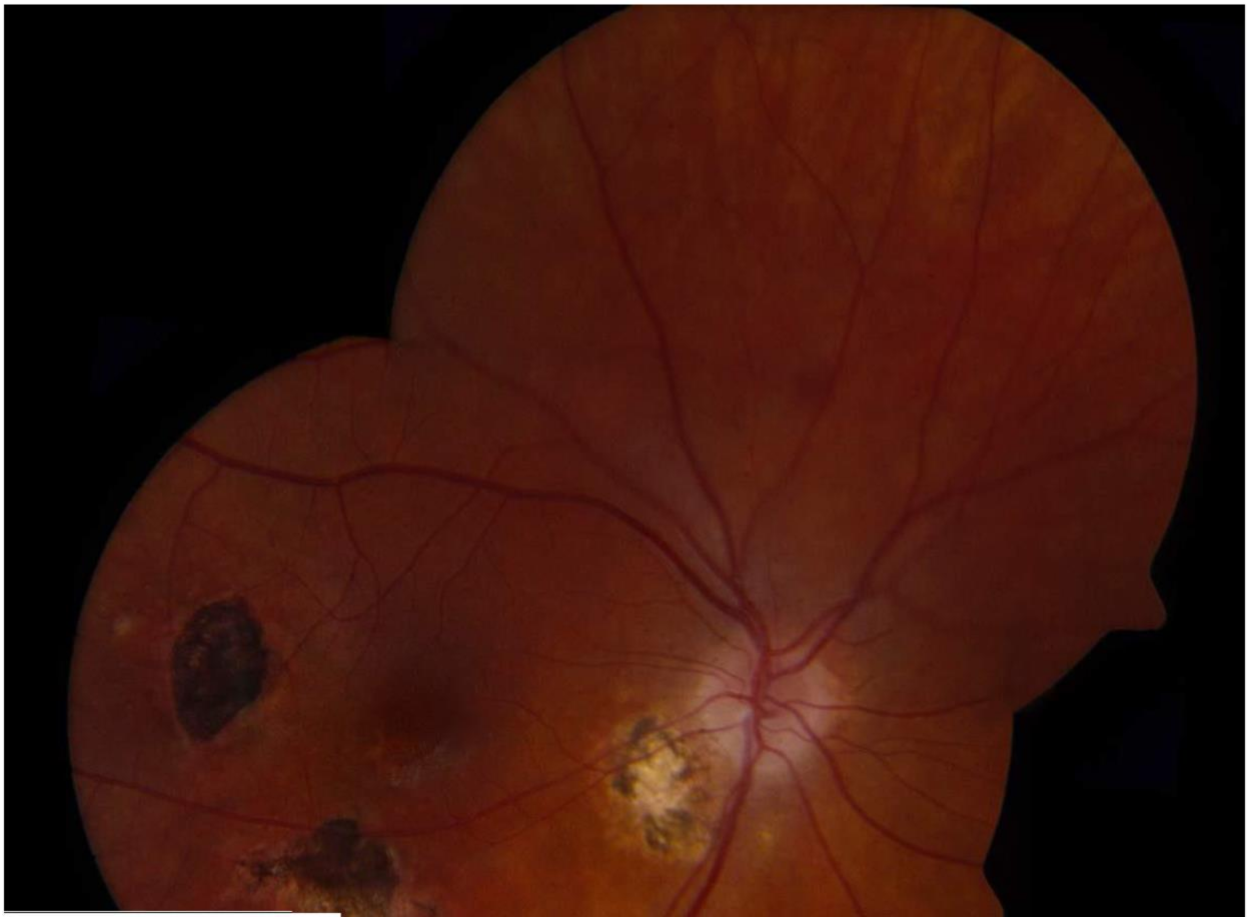

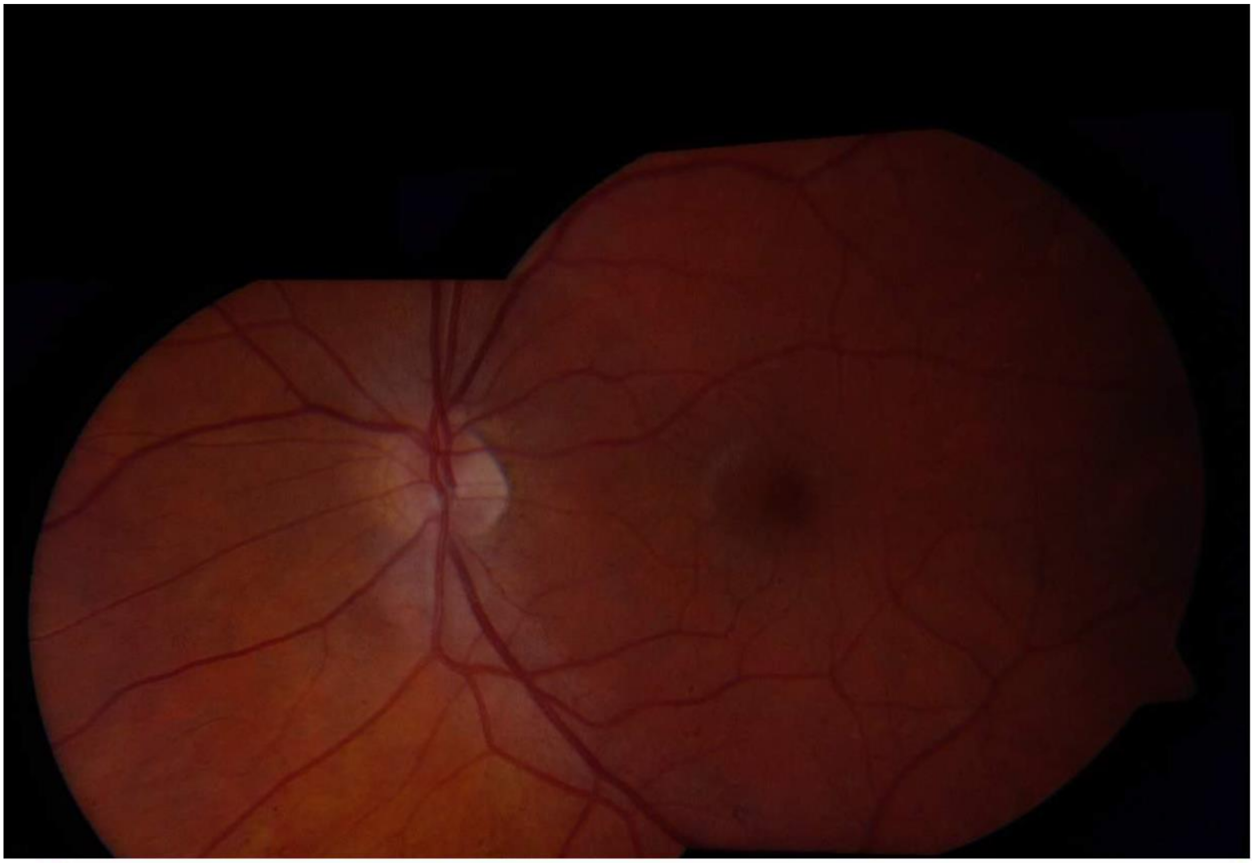

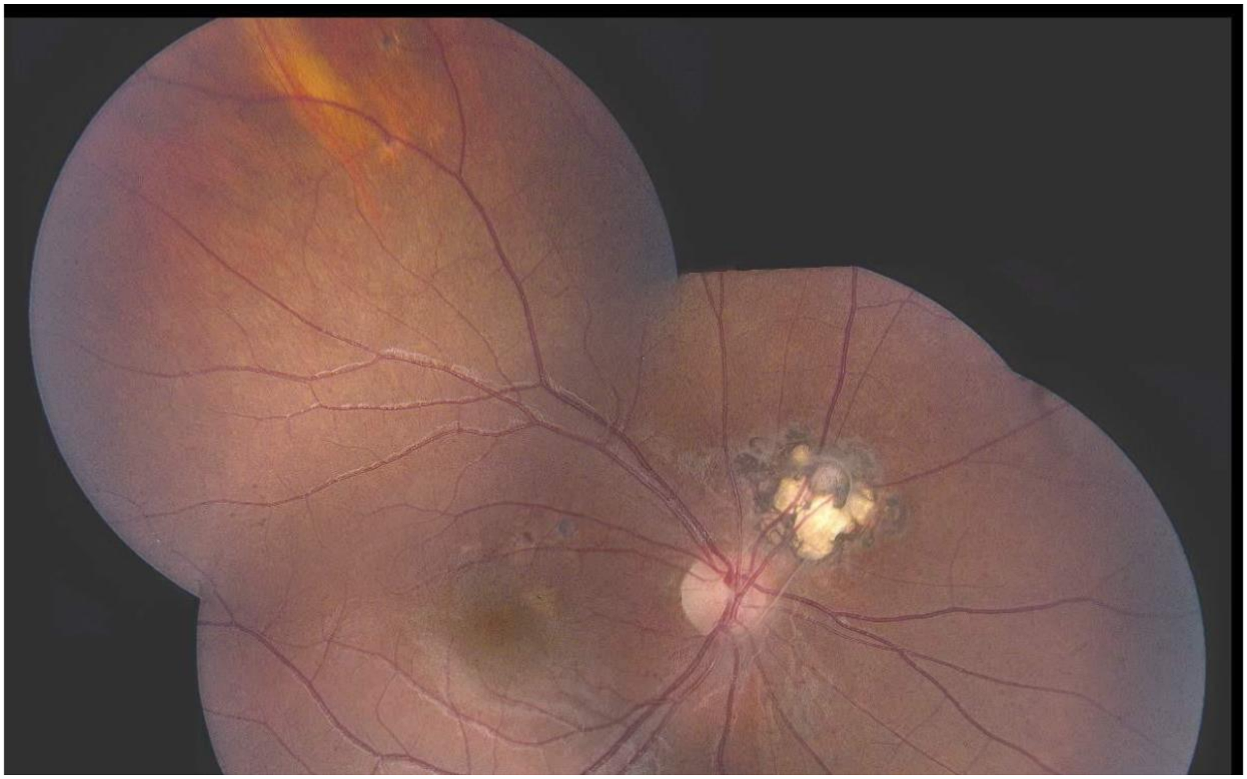

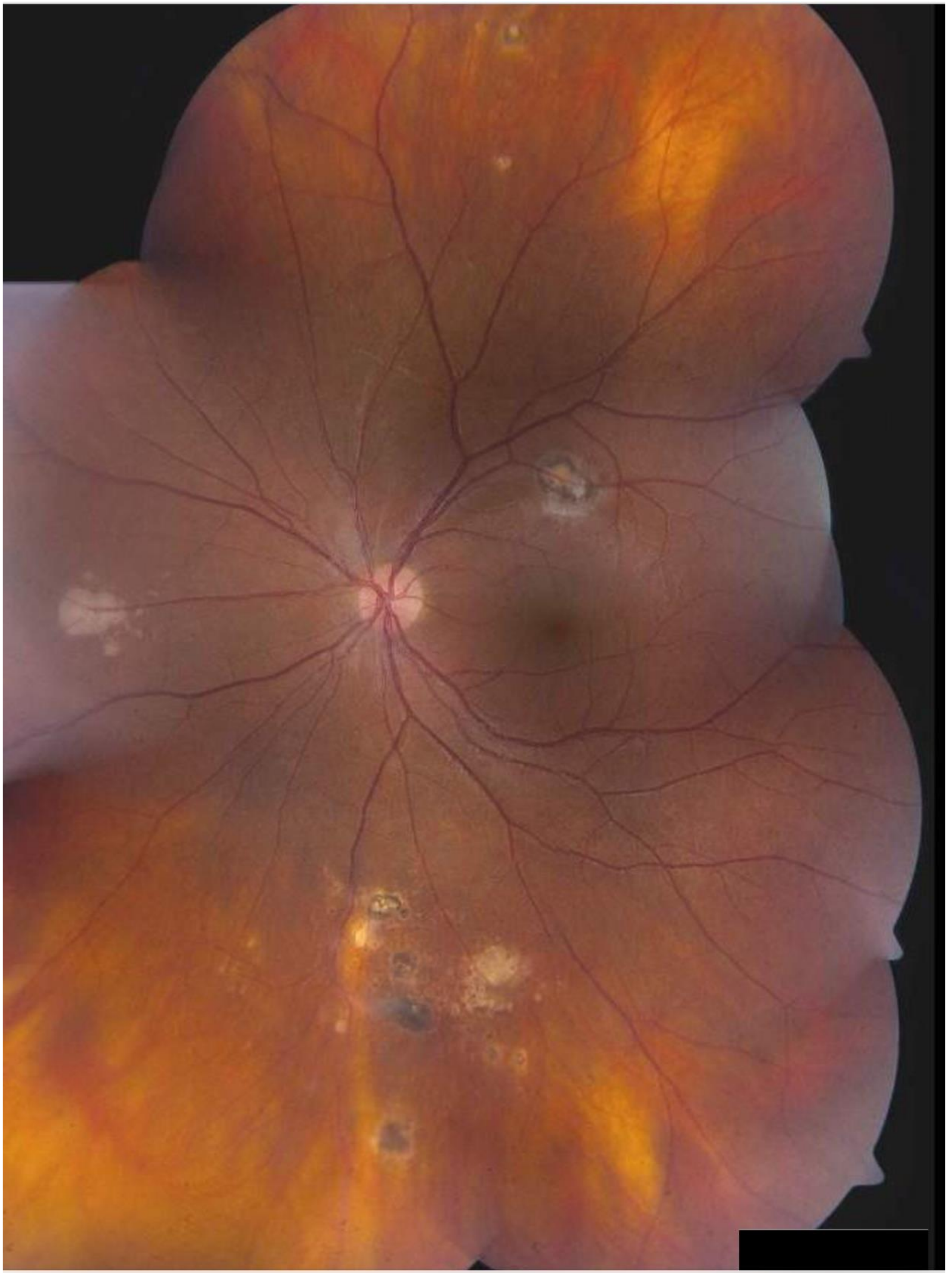

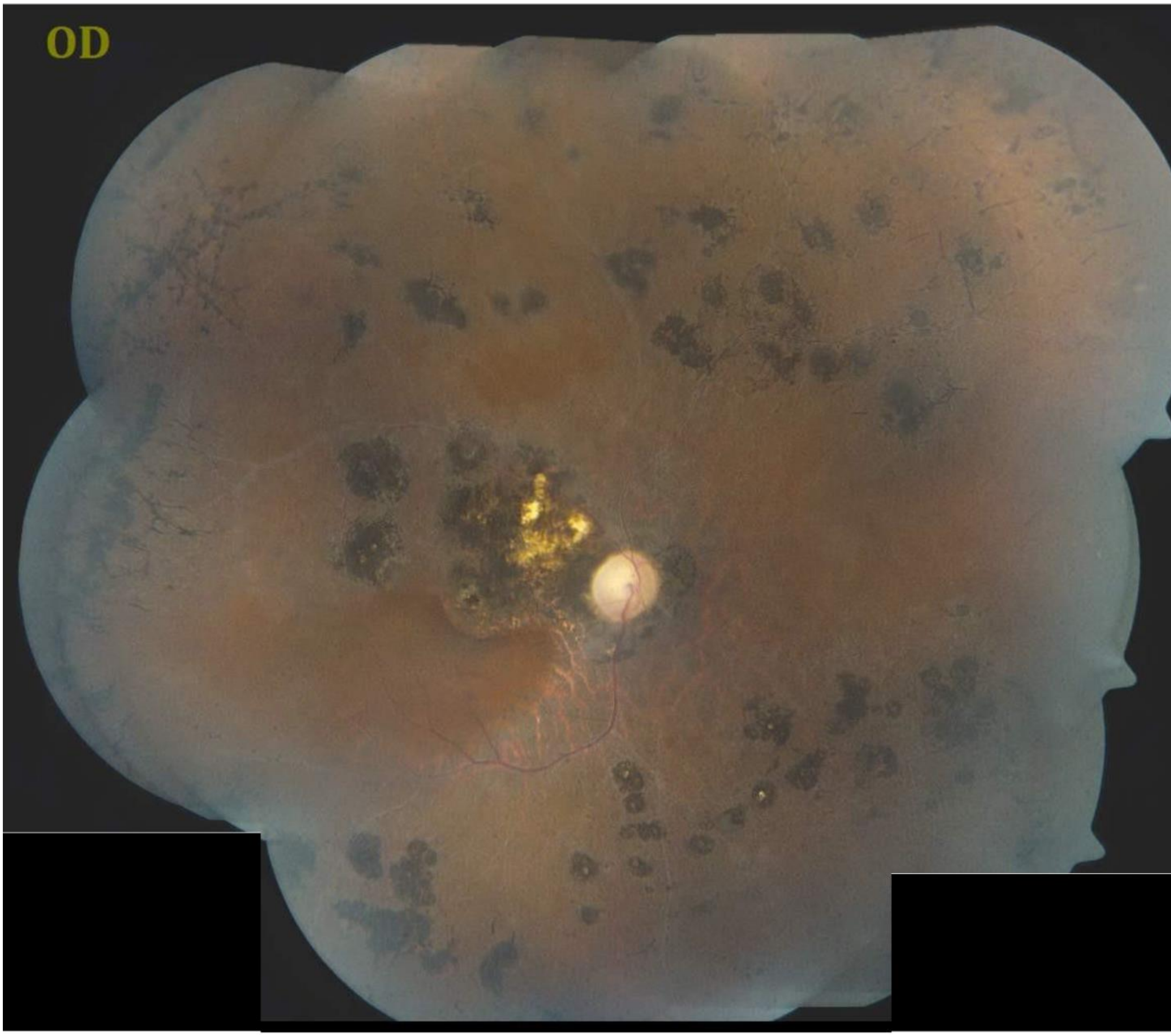

Patient Q- right eye: 2+ optic nerve diffuse pallor. Peripappillary choriortetinal atrophy scar pigmented scar extending to the macula from the superior direction. It ha[illegible] engulfed the fovea. numerous pigmented chorioretinal scars scattered throughout the periphery in right eye.

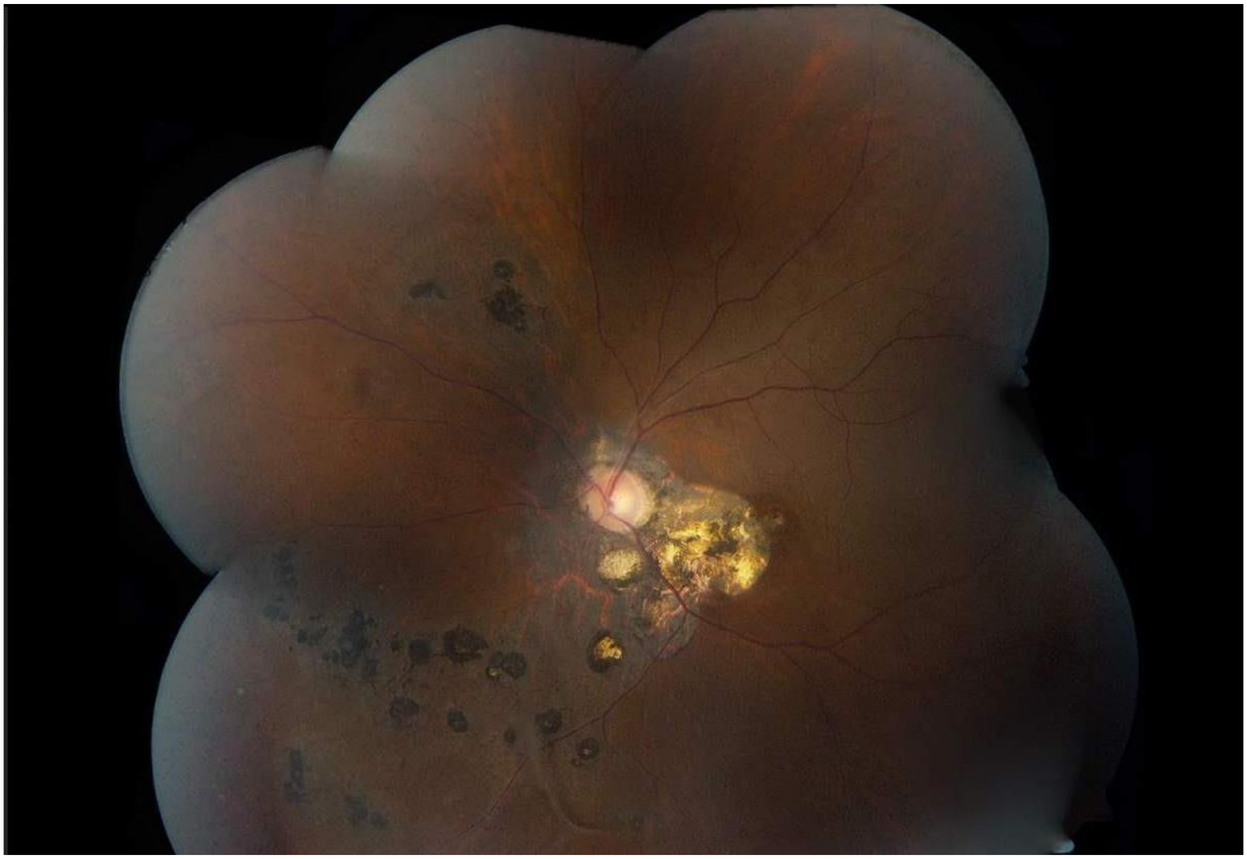

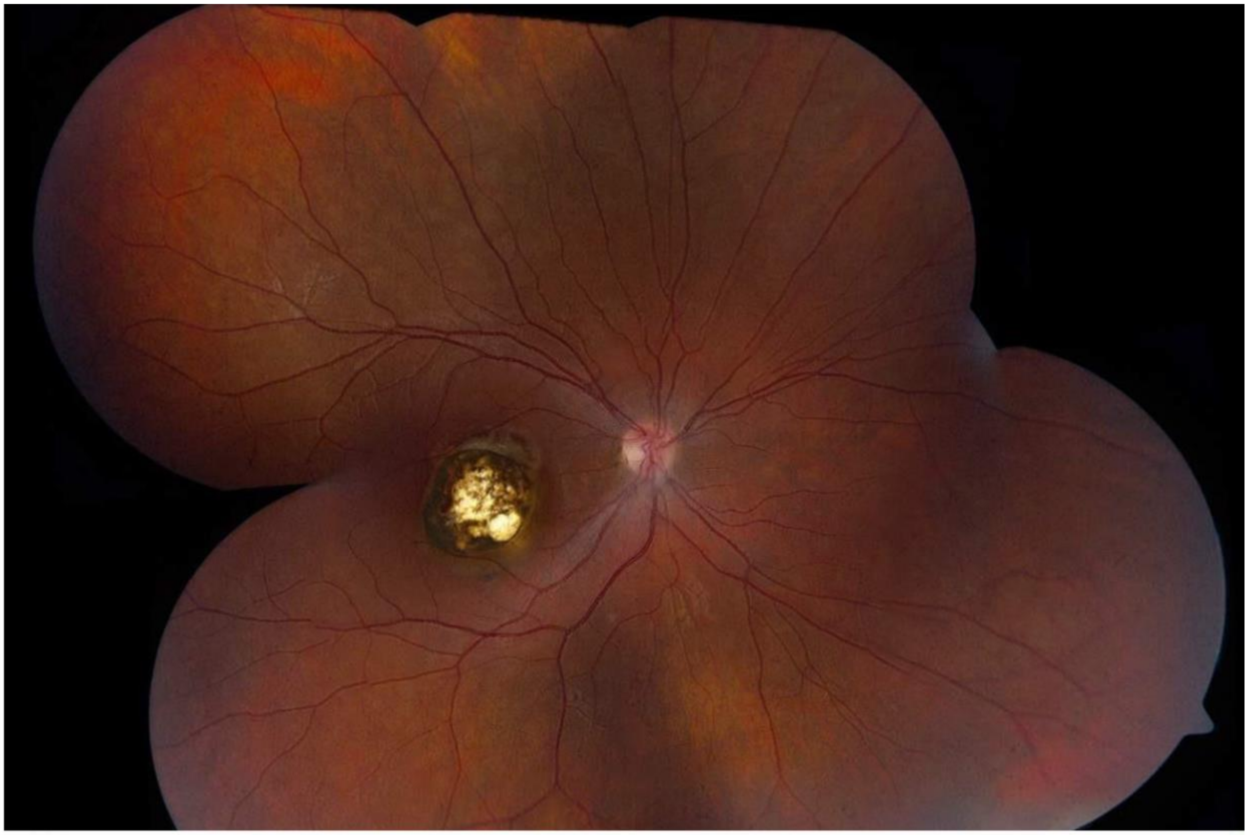

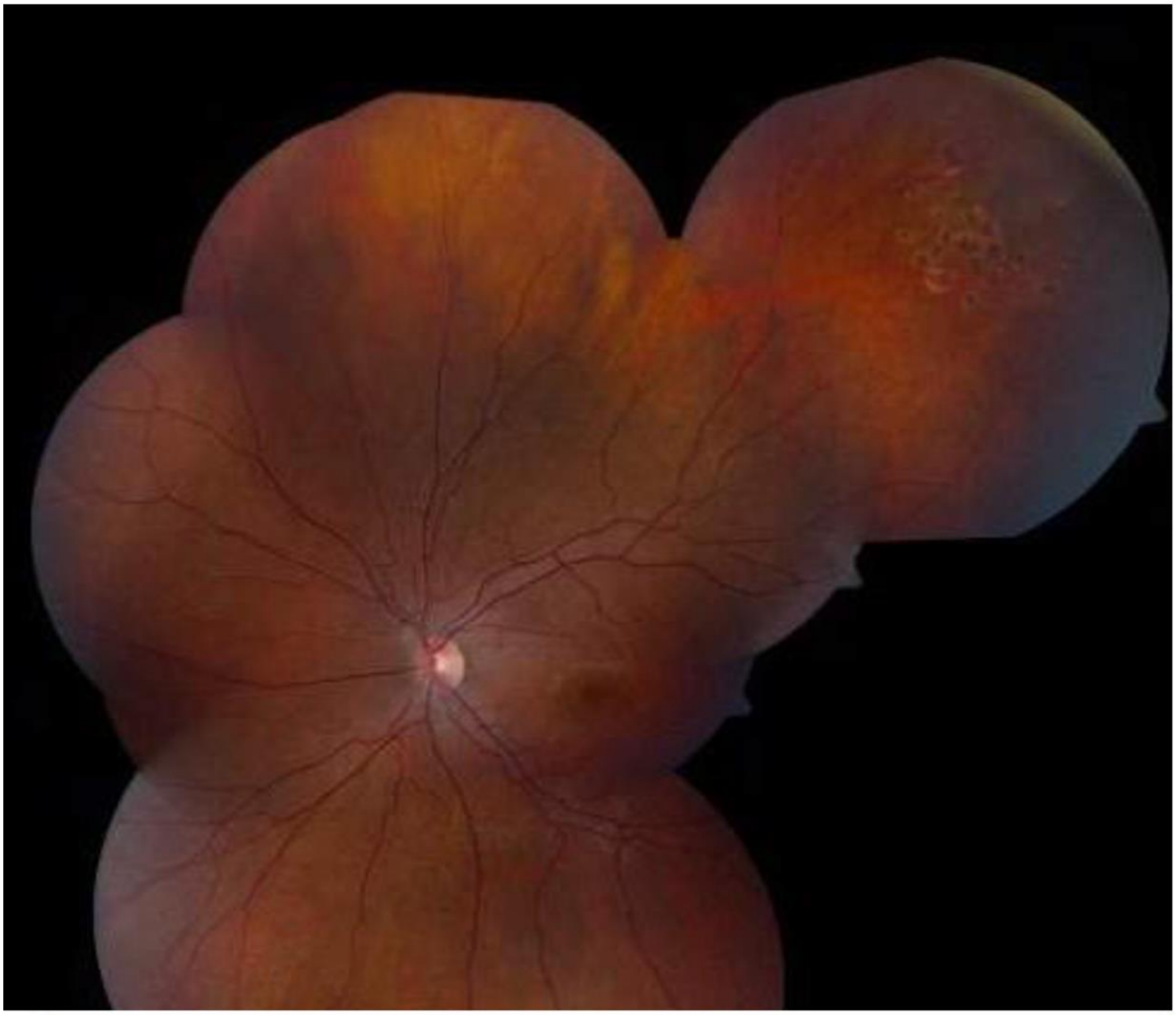

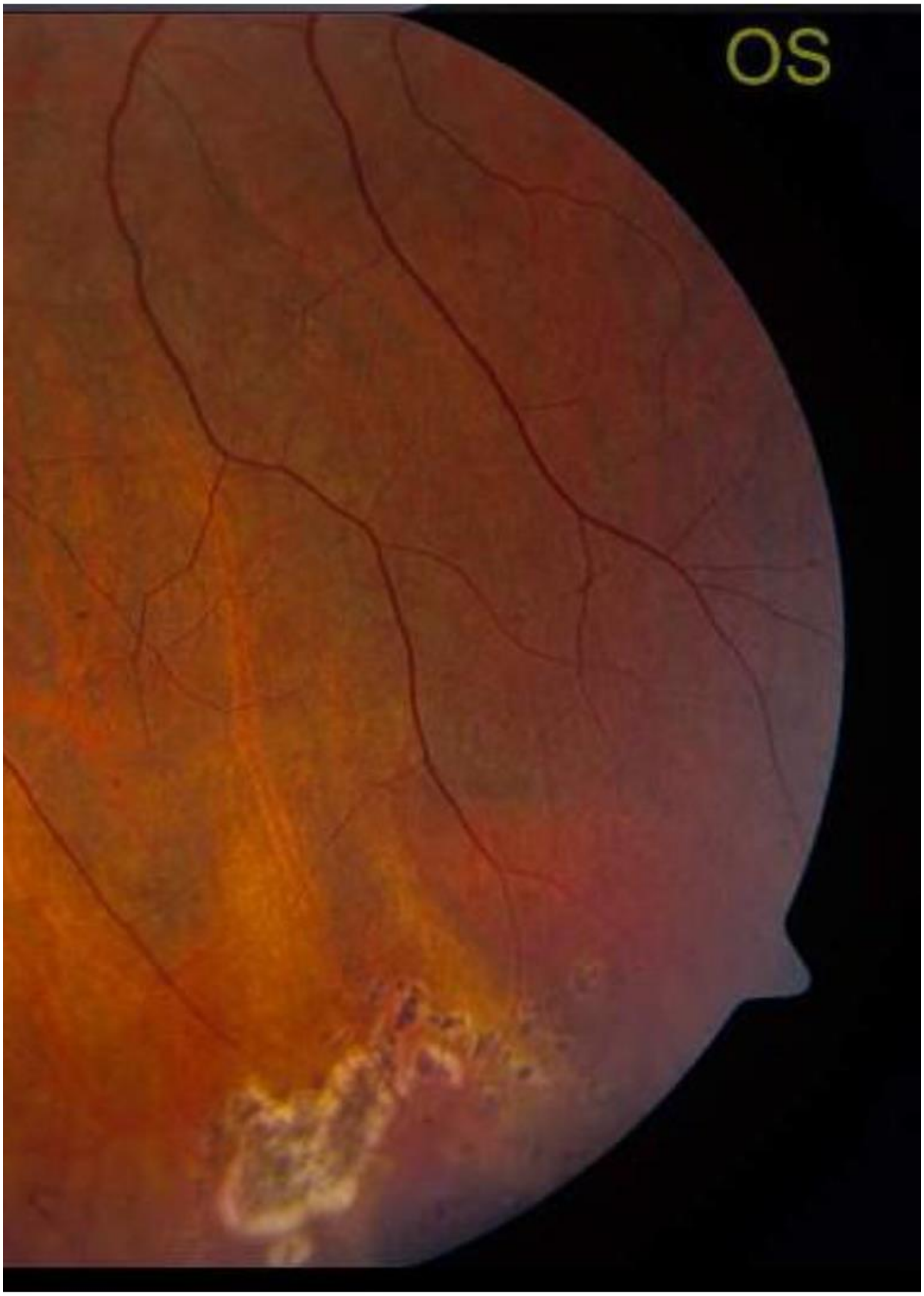
OS

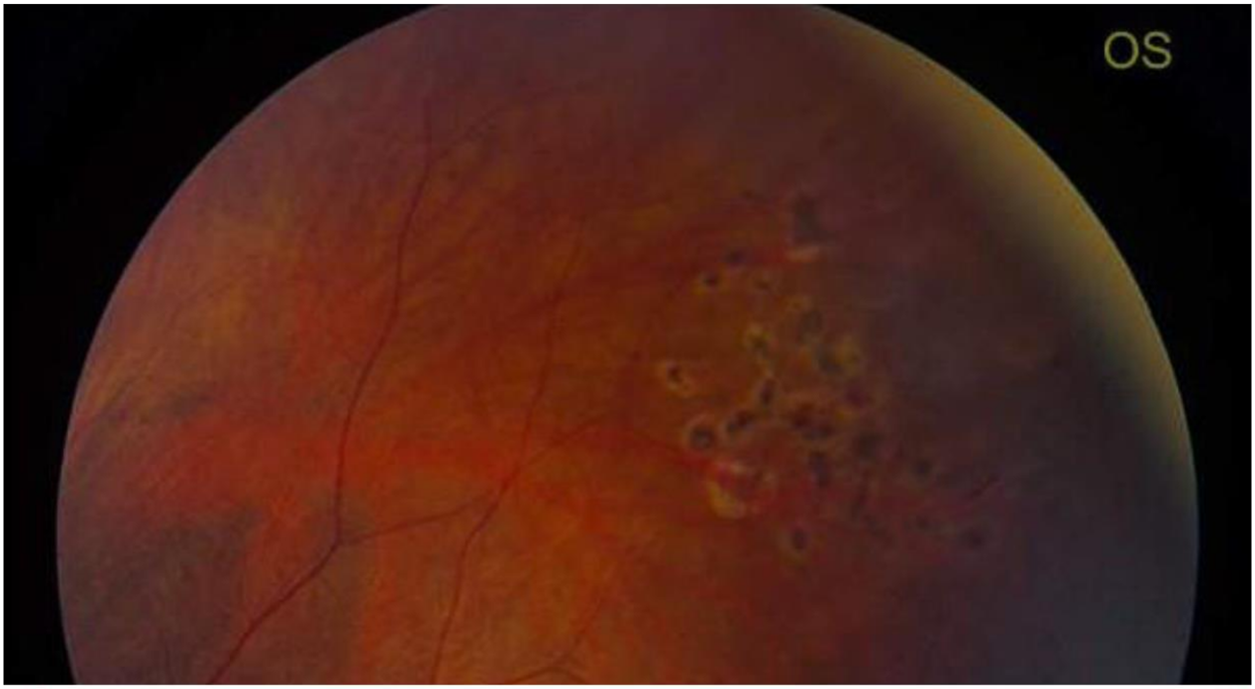
OS

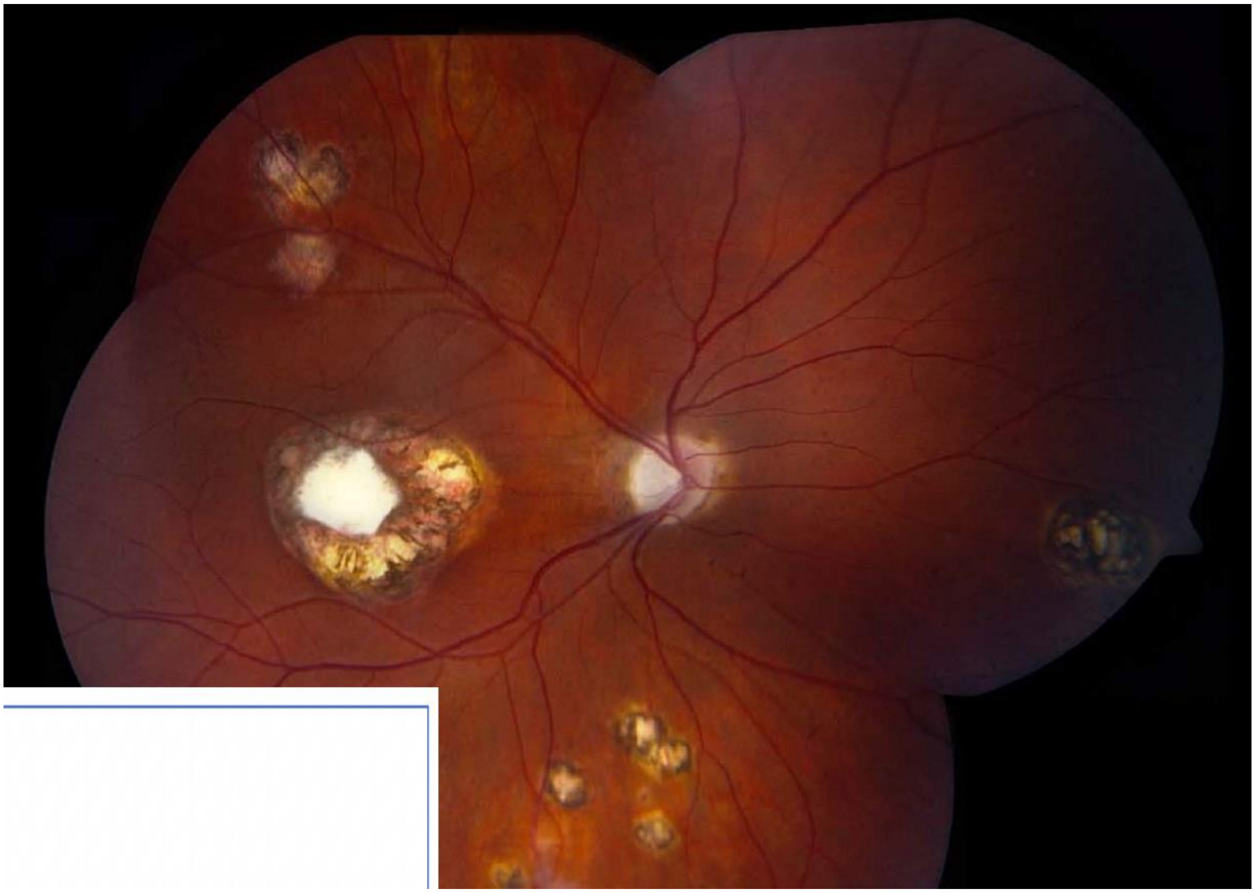

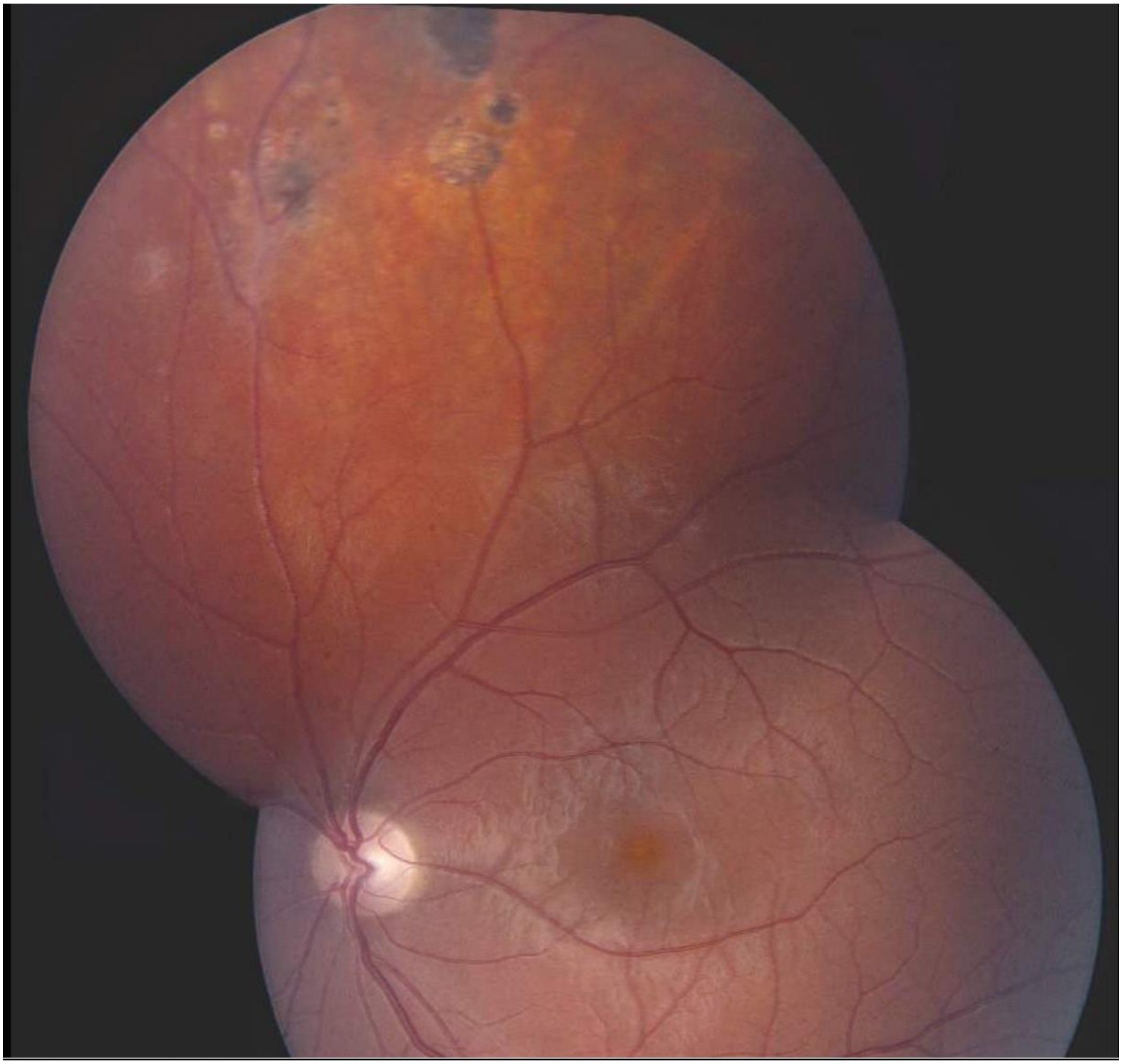

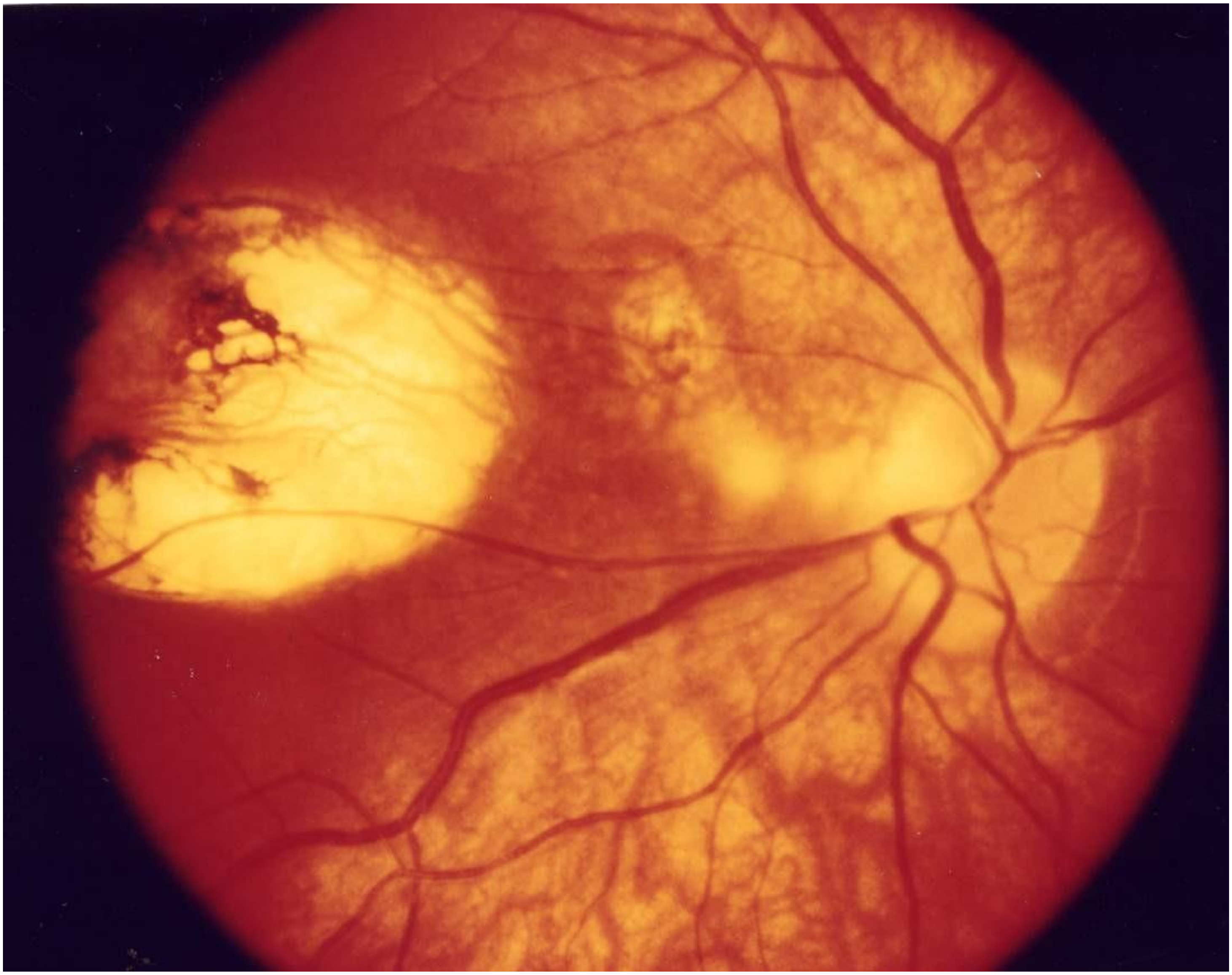

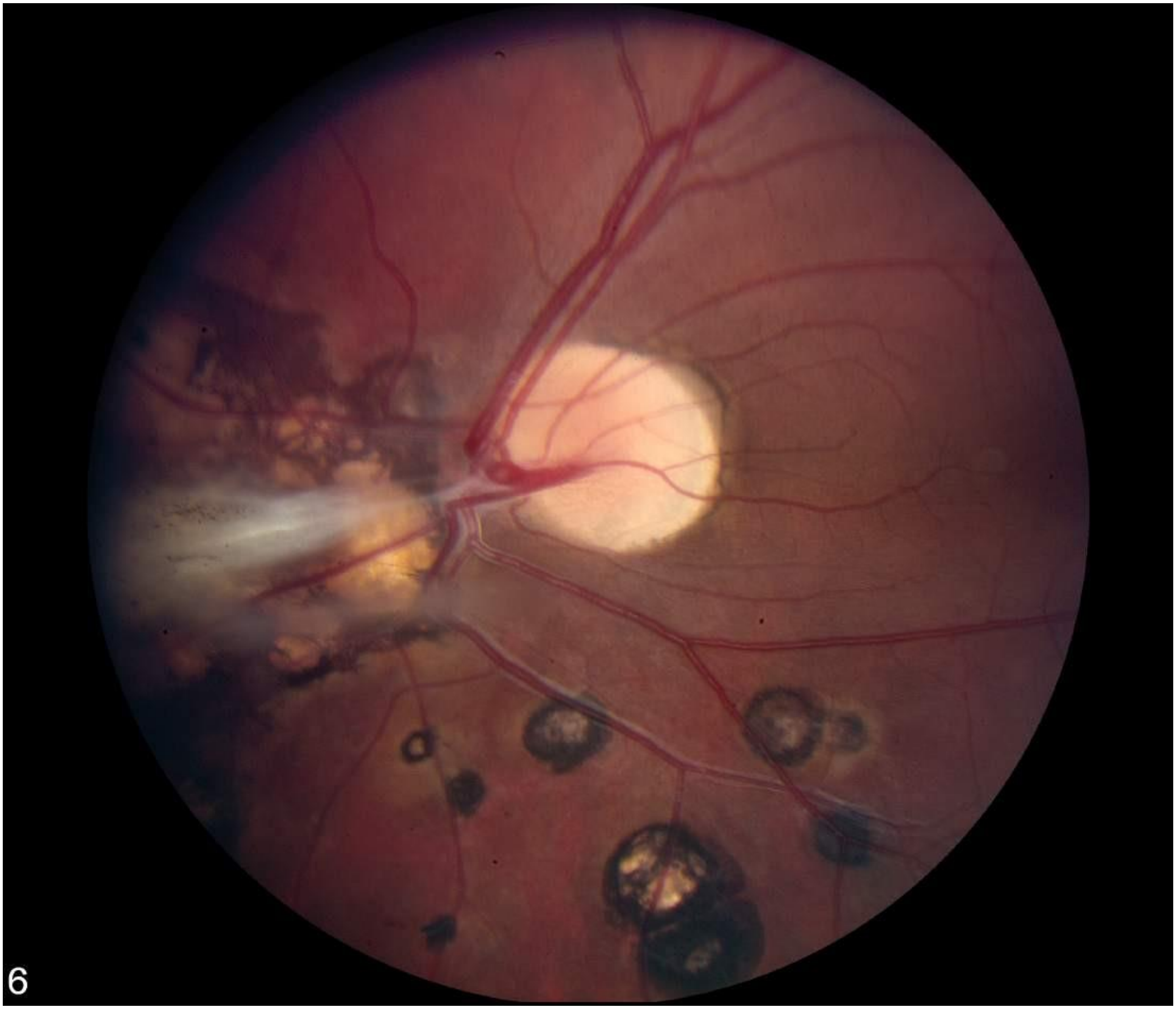

Gliotic lesion extending from the optic nerve to the large macular lesion. Note dragging of the optic nerve and vessels, as well as multiple scattered satellite lesions inferior to the optic nerve.

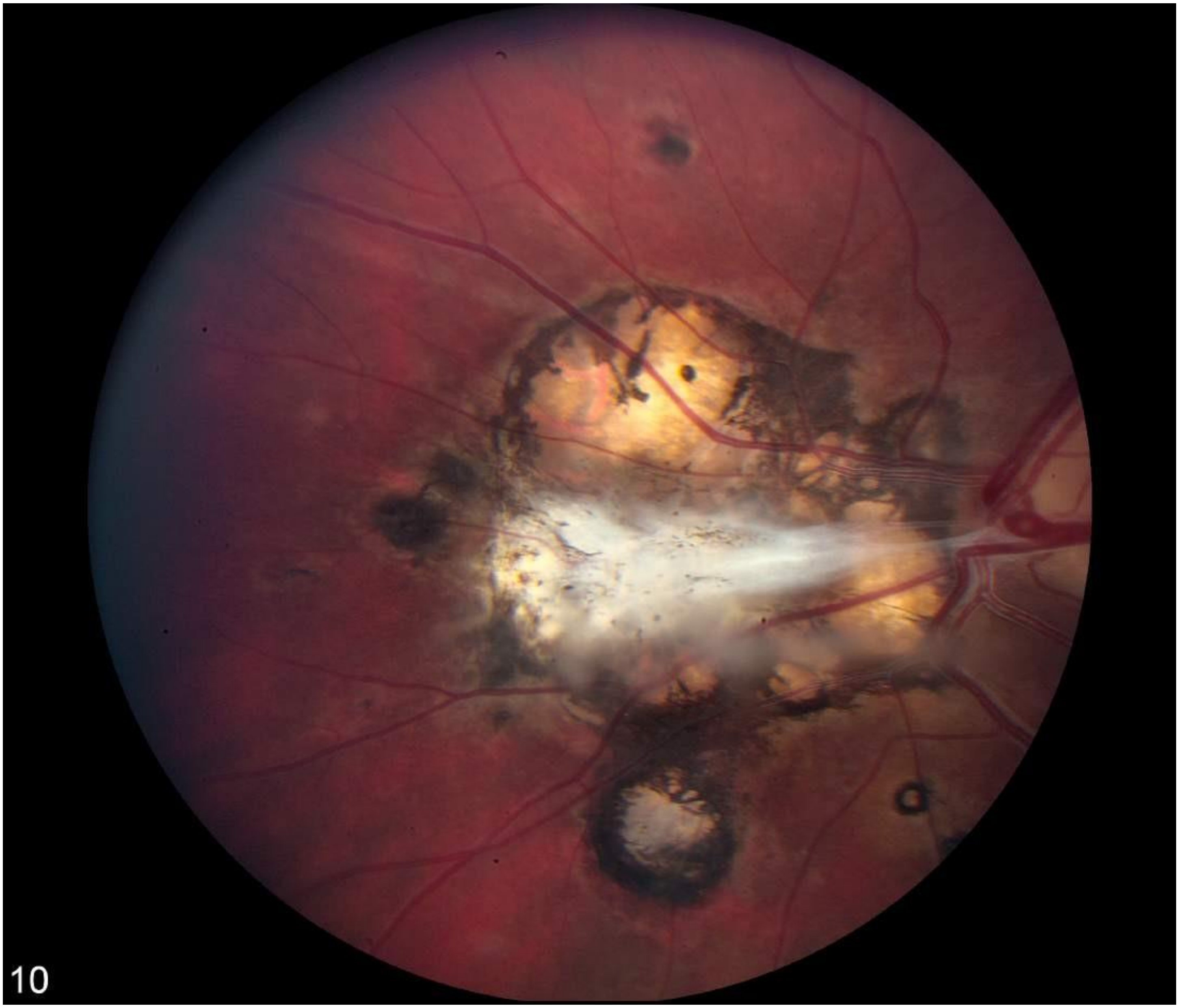
10

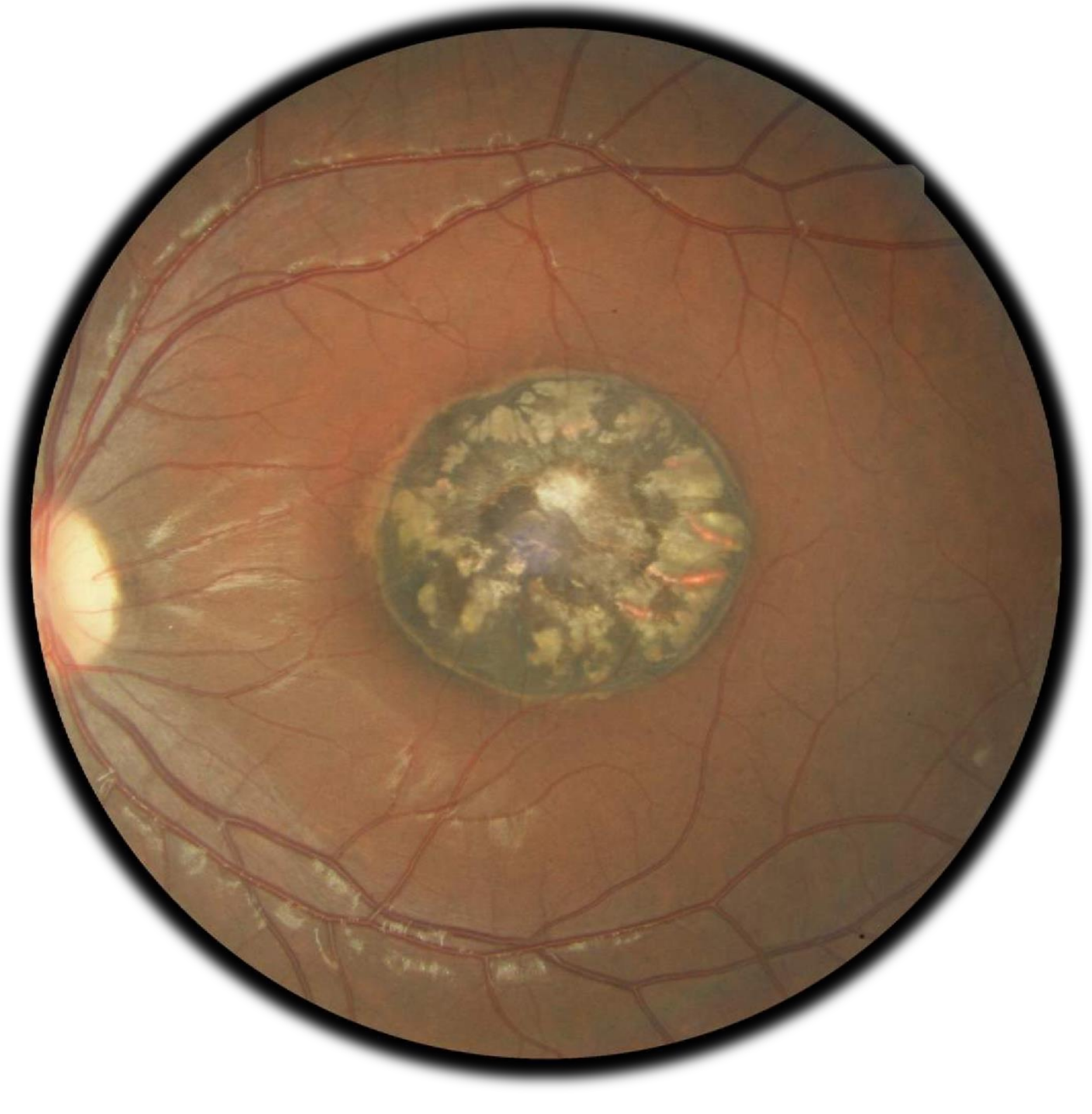

Patient R- January 2014, Classic Congenital lesion, well circumscribed with hyperpigmented edges, macular lesion covering the fovea.

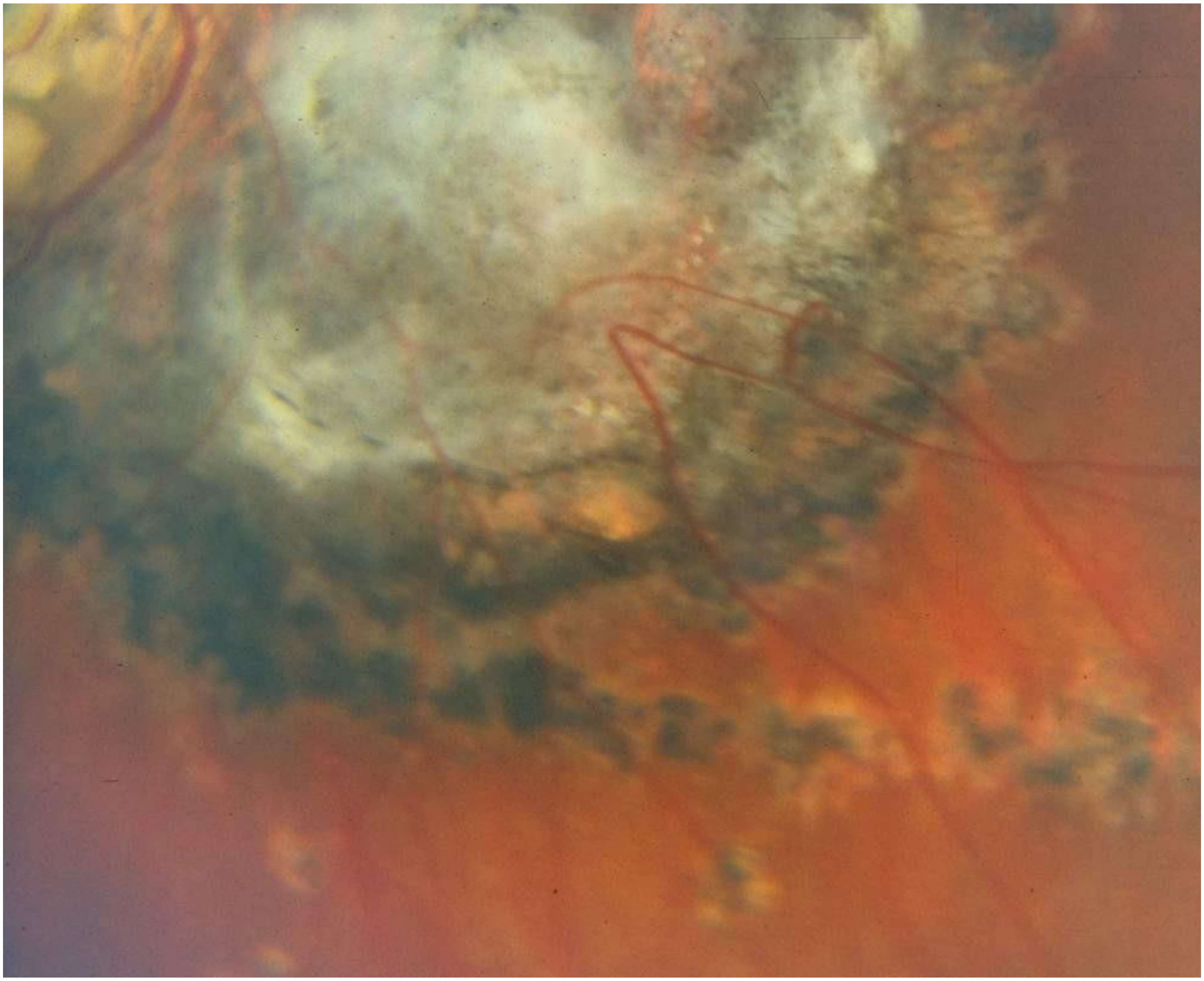

Large scar with irregular, hyper-pigmented borders, deep, excavated center, and trail of satellite lesions.

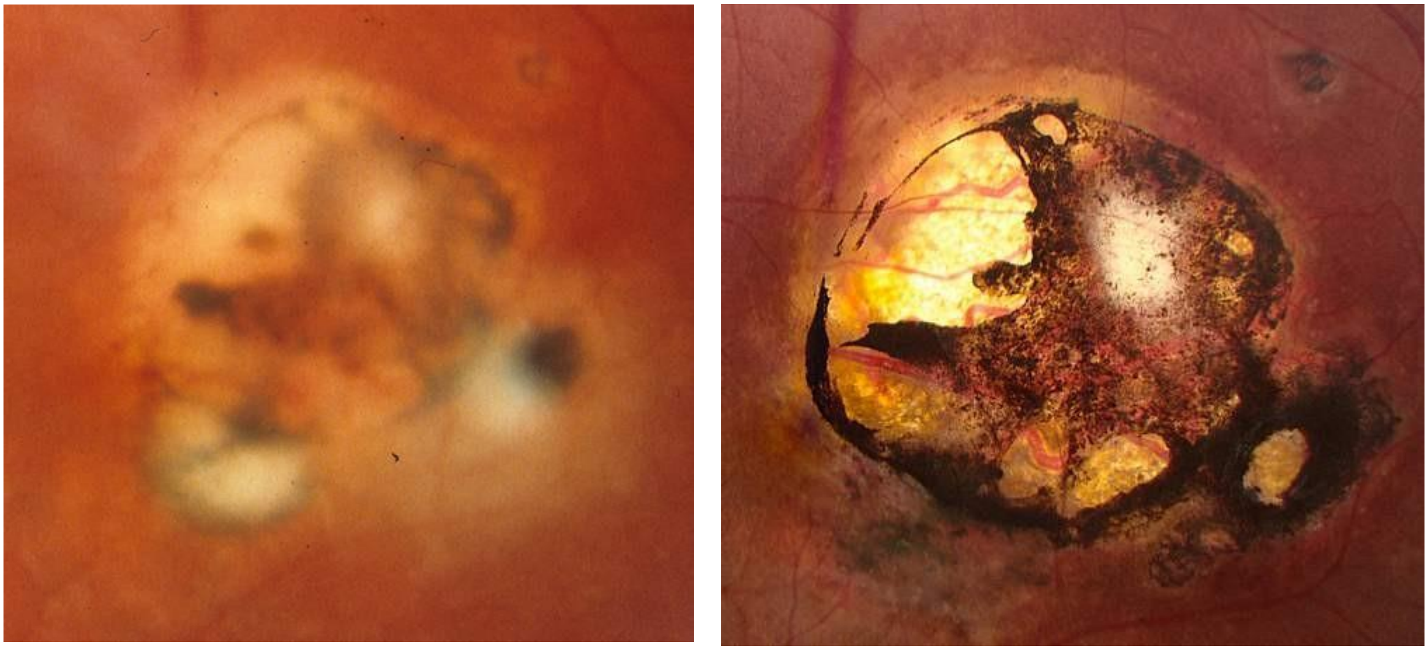

Evolution of large, circular macular lesion. Lesion was active, and severe inflammation makes the image blurry and appearing out of focus. In the right image, the lesion on the becomes more hyperpigmented overtime.

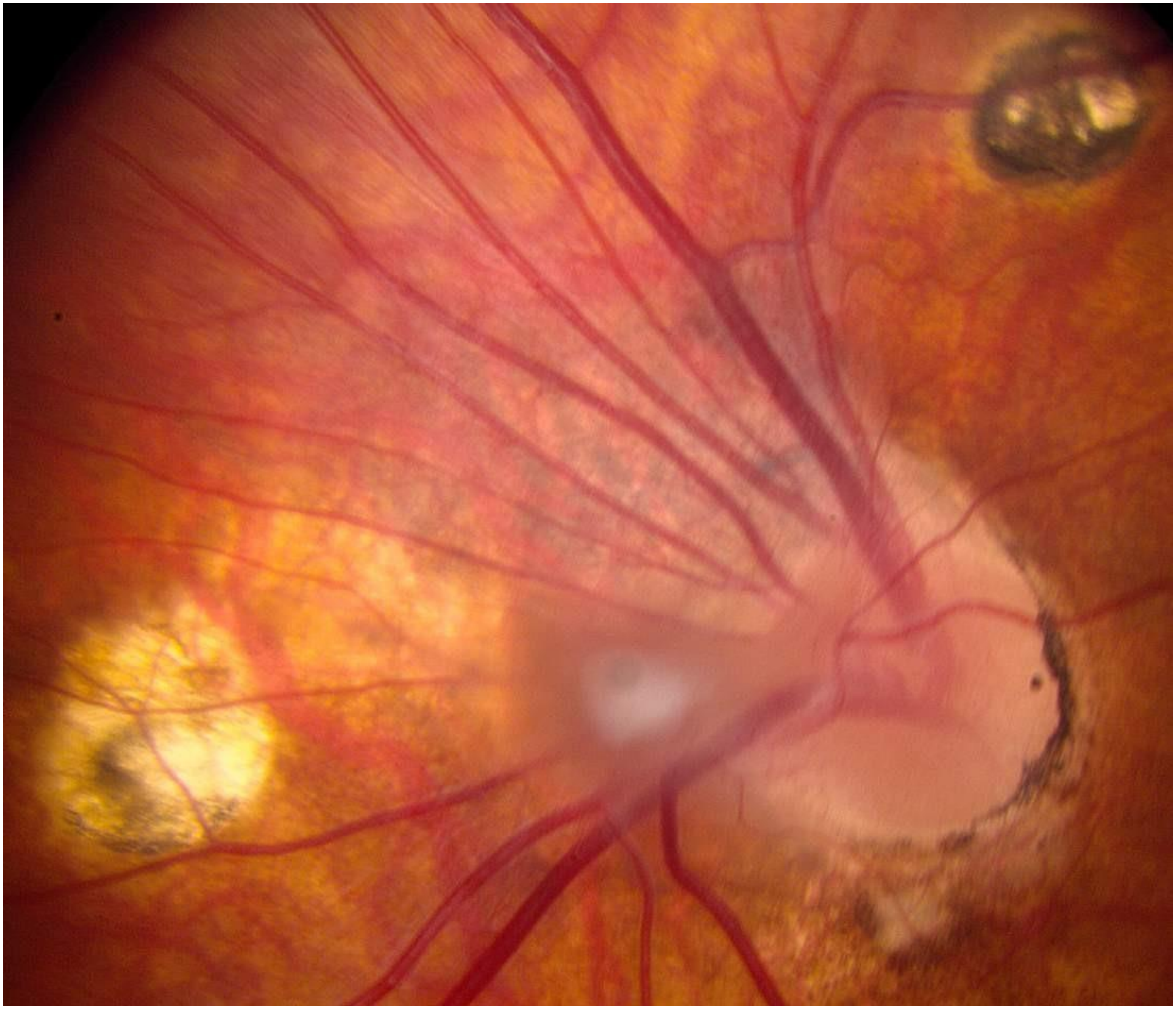

Lesions surrounding the optic nerve that is surrounded by fibrotic tissues. There is also extensive straightening of the vessels.

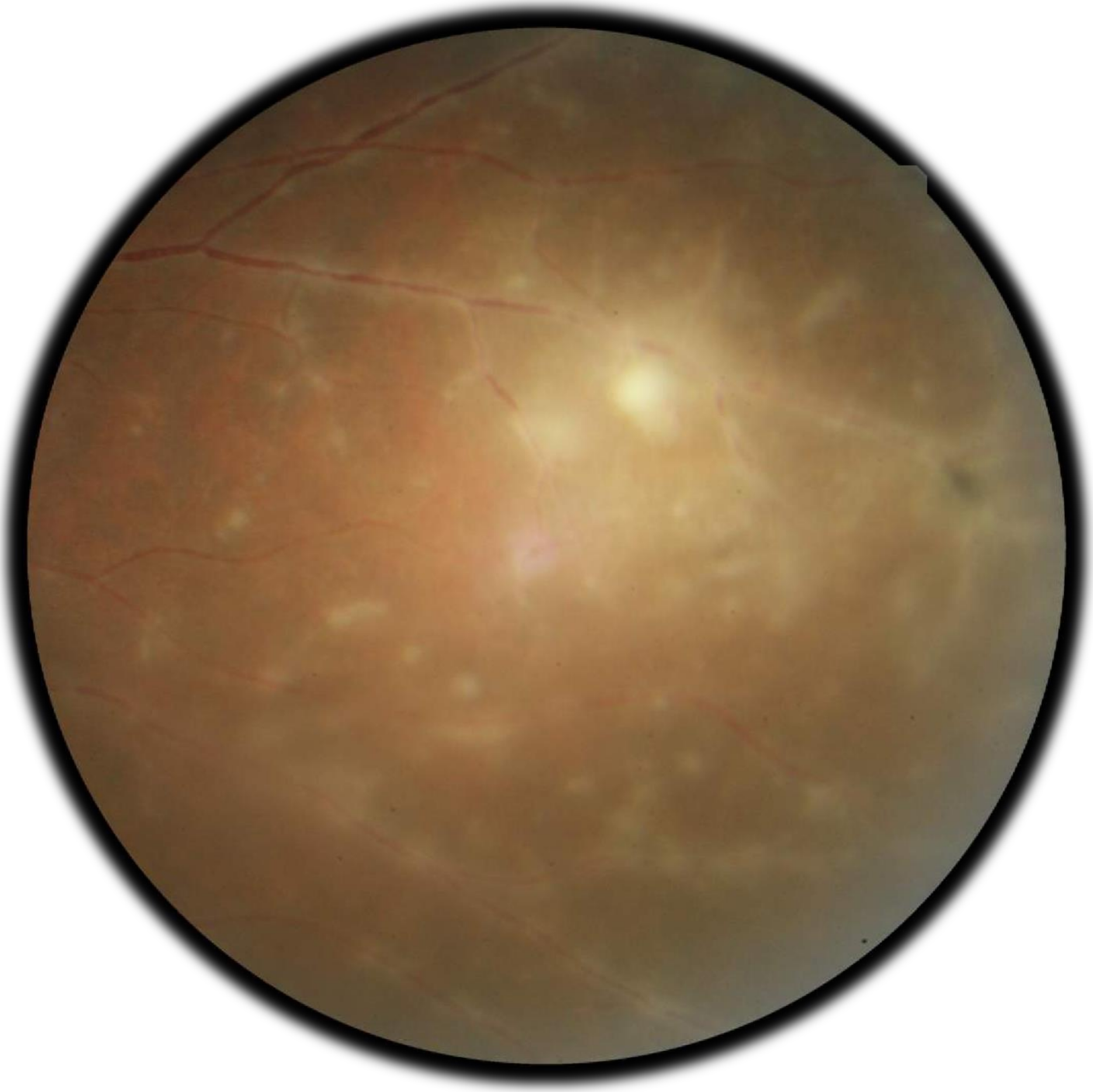

Patient R- January 2014, Acute Toxoplasmosis lesion with Kyrieleis (trail of scattered satellite lesions)

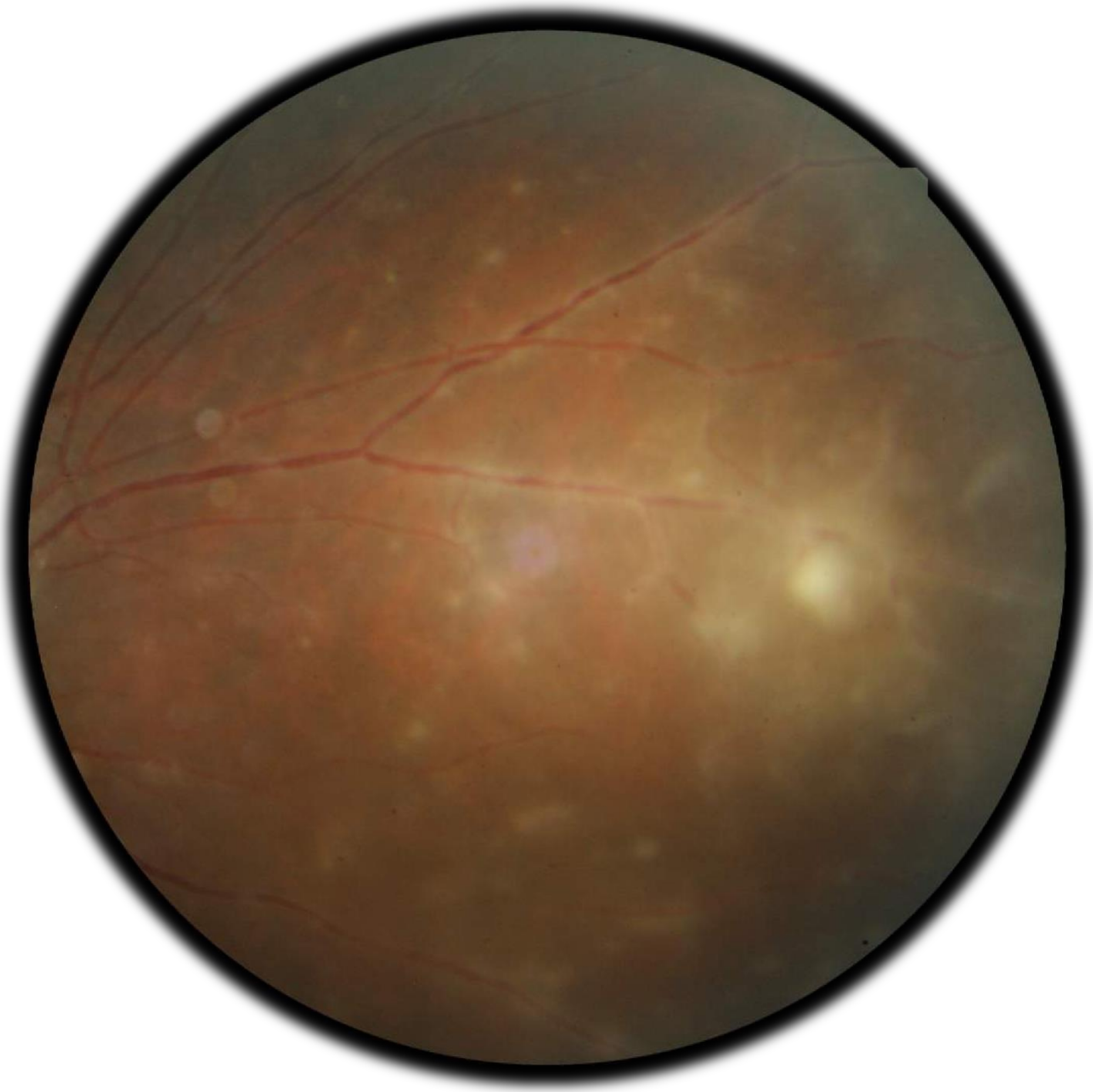

Patient R-January 2014, Acute Toxoplasmosis lesion with Kyrieleis (trail of scattered satellite lesions) or
Miliary toxoplasmosis

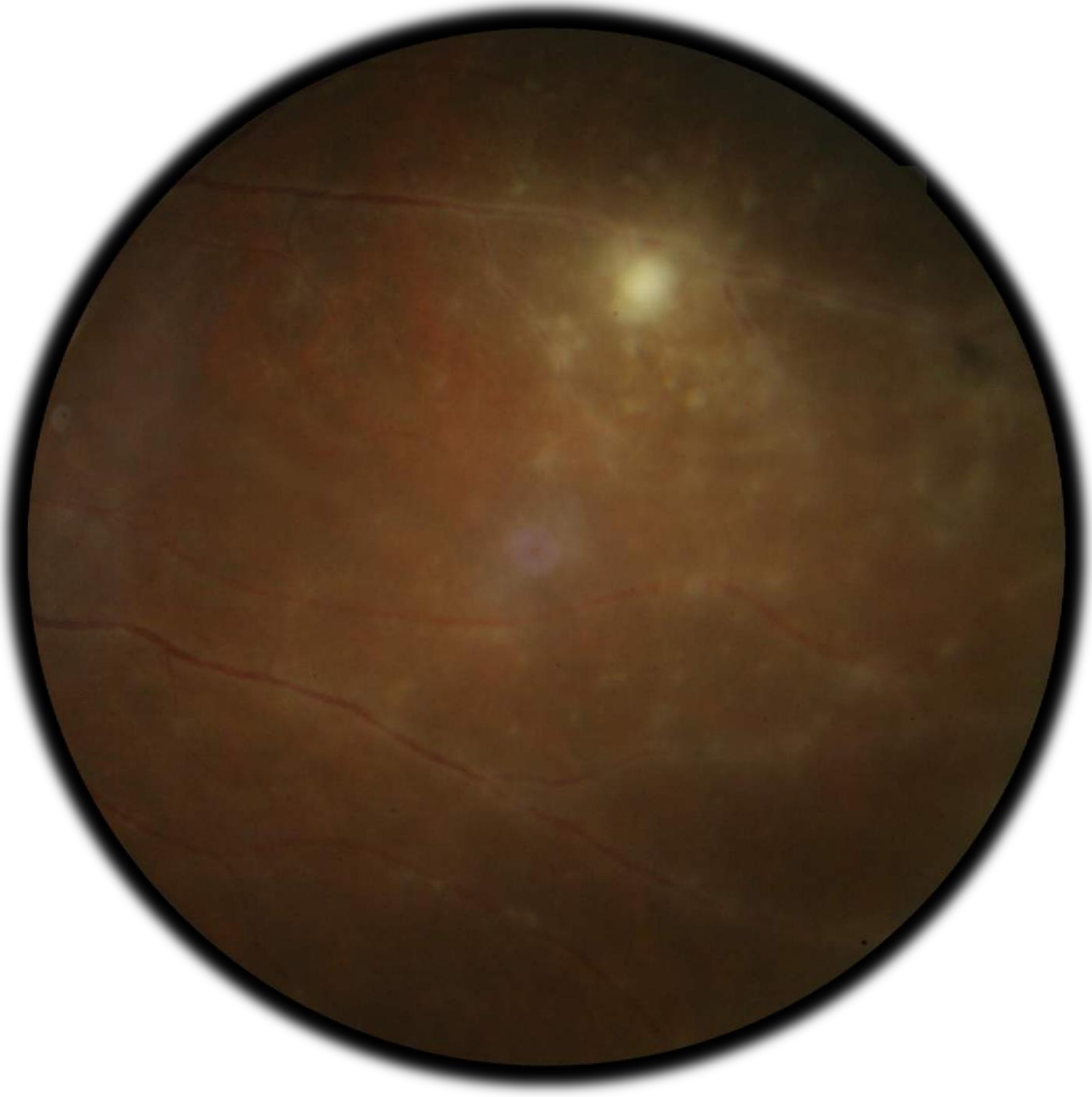

Patient R- February 2014, Fluffy new active lesion, creamy, not well defined, indistinctive borders

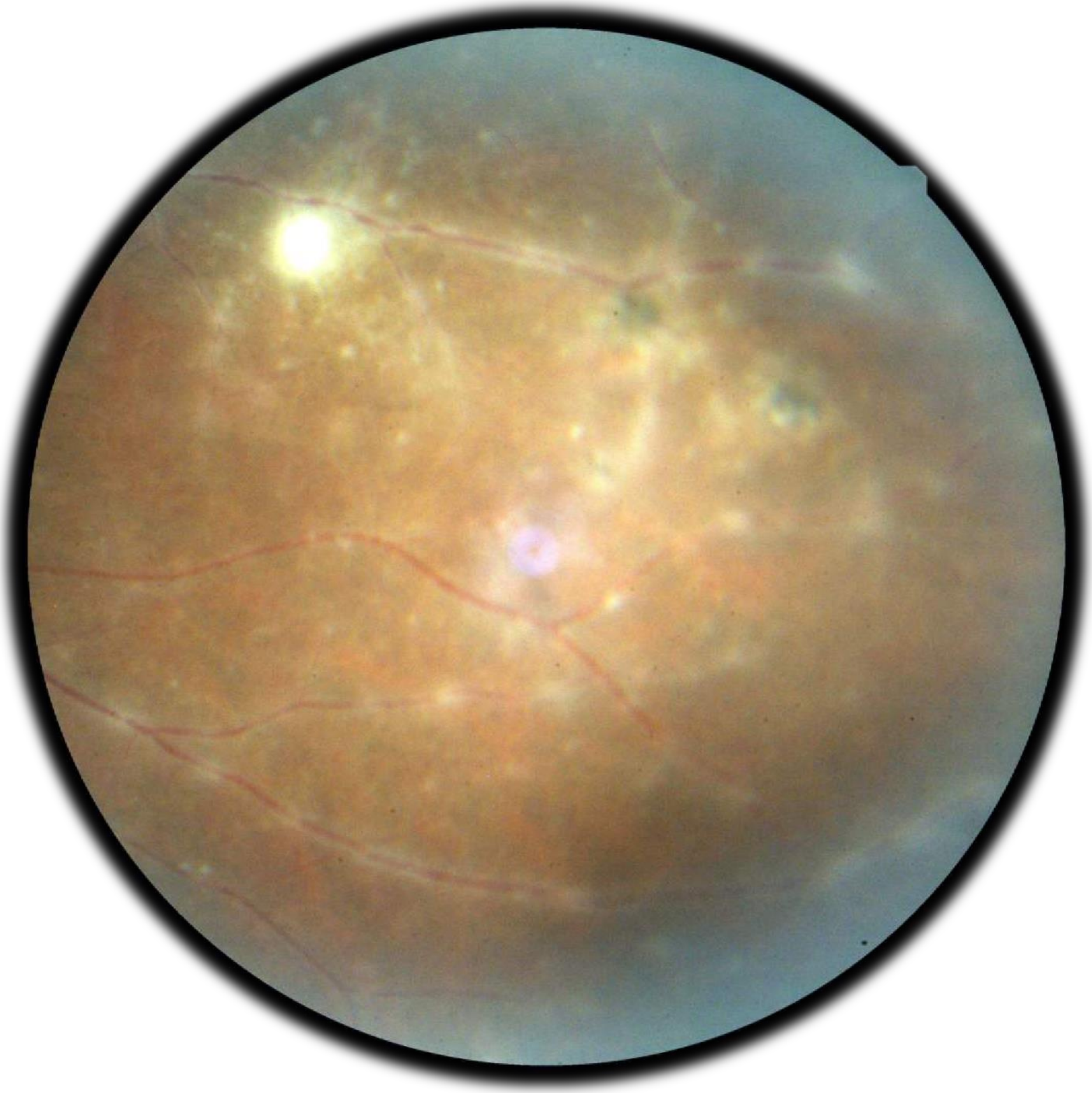

Patient R - February 2014, Trail of satellite lesions overlying retinal vessels

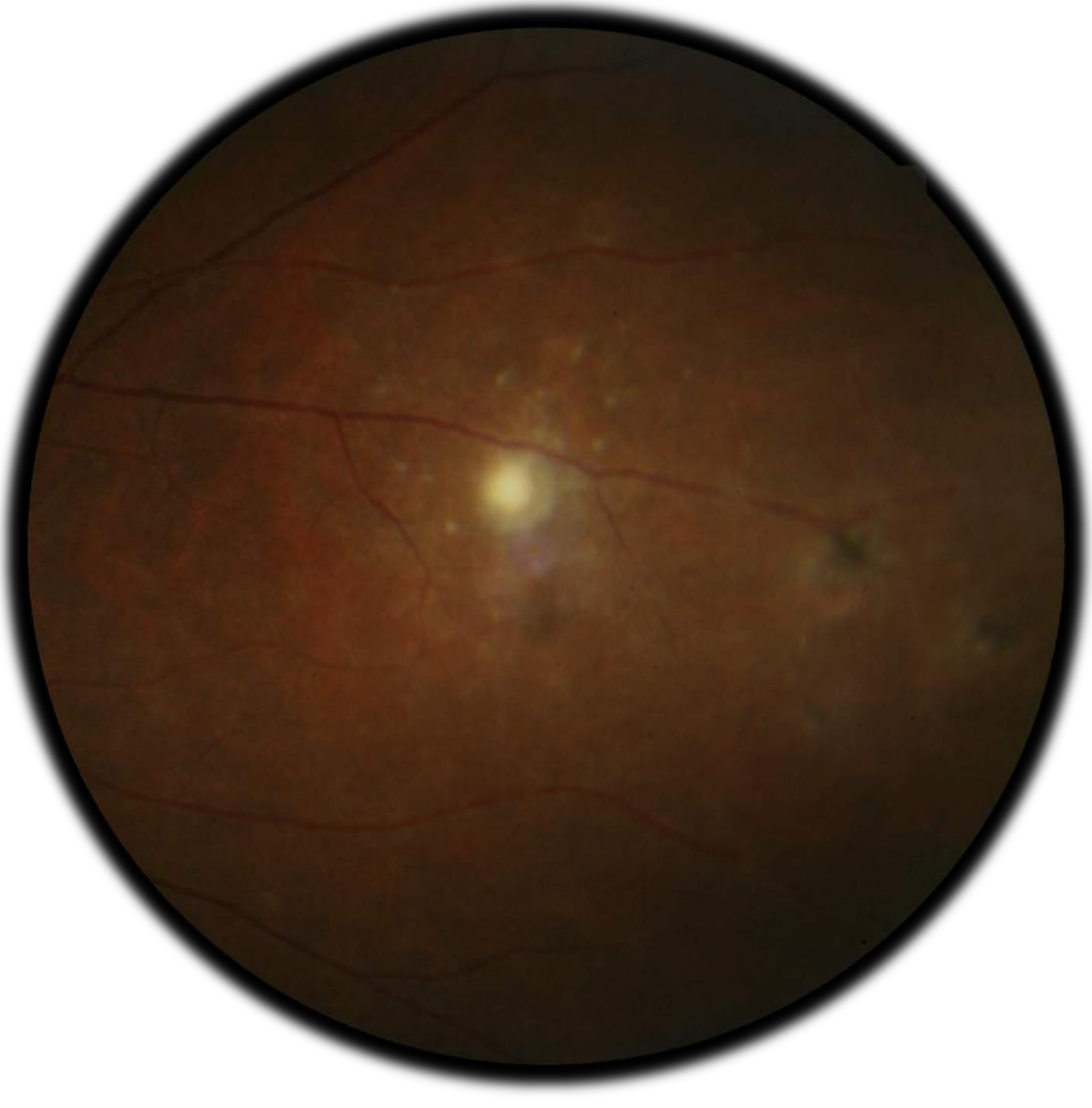

Patient R-March 2014- Lesion is becoming more defined and less hazy, 2 months post treatment

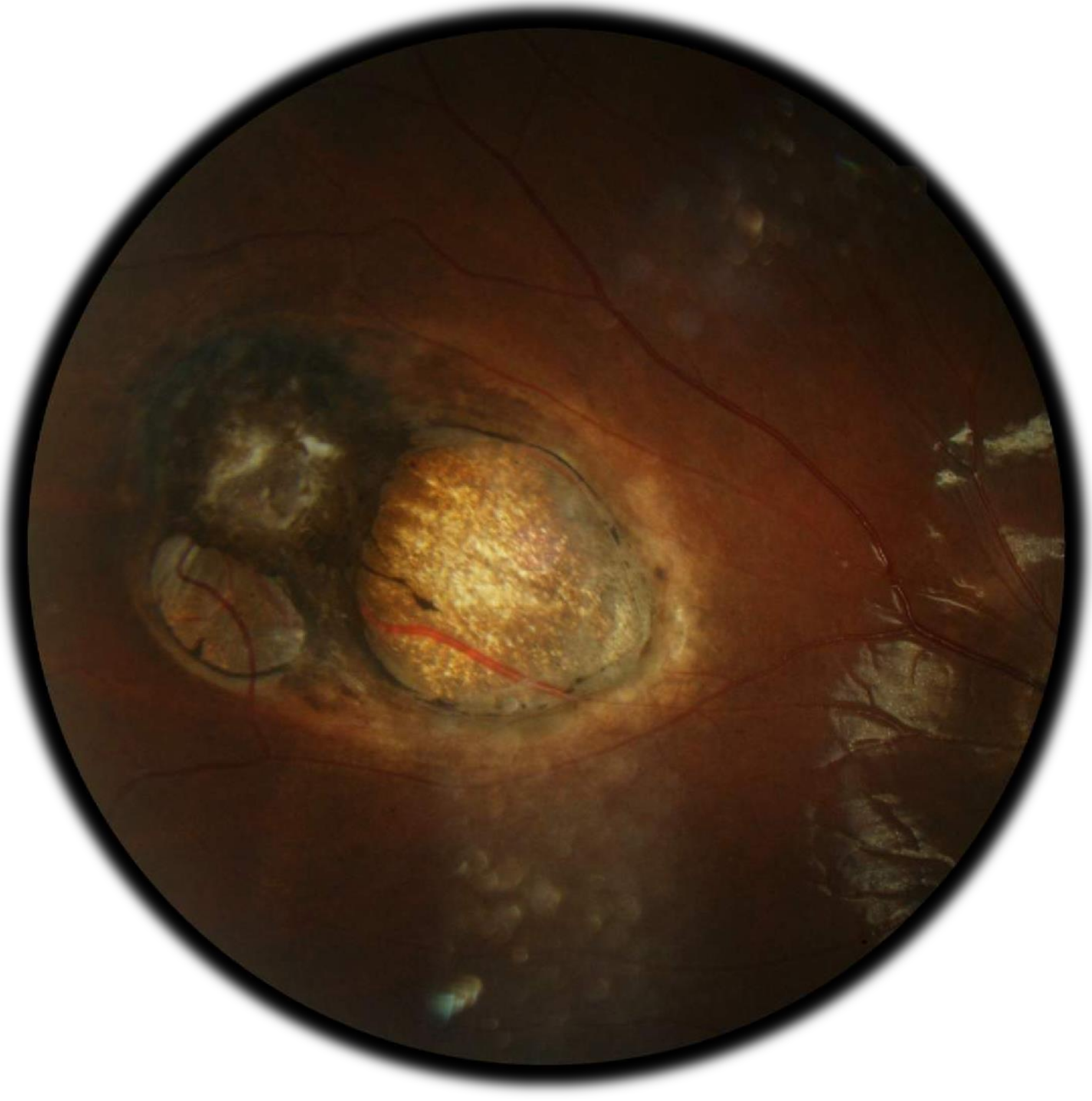

Patient S- February 2014, Left Large, excavated peripheral hypopigmented lesion with hyperpigmented borders, trilobed,

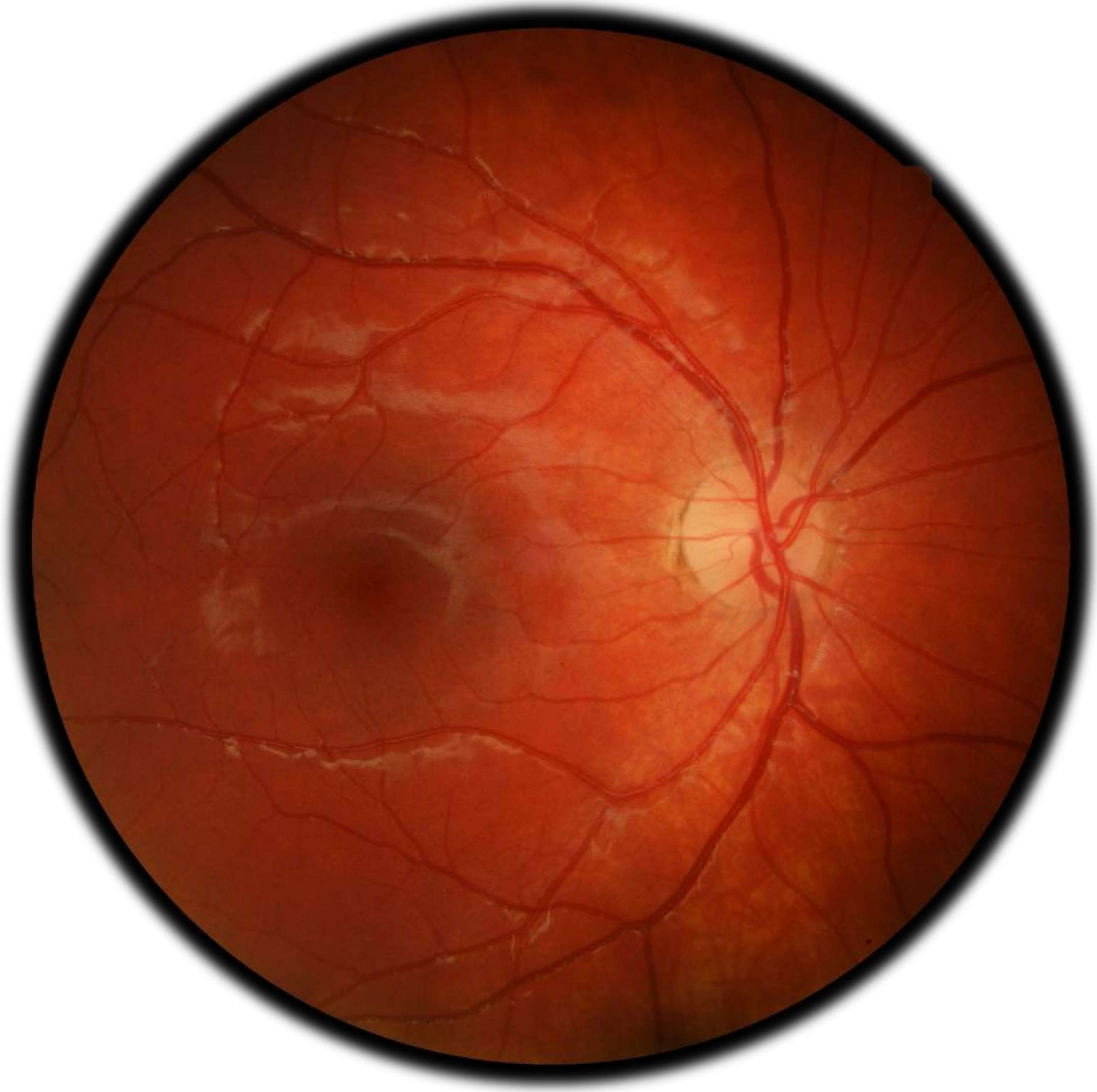

Patient T-November 2012- Normal Fundus in right eye, treated the first year of life

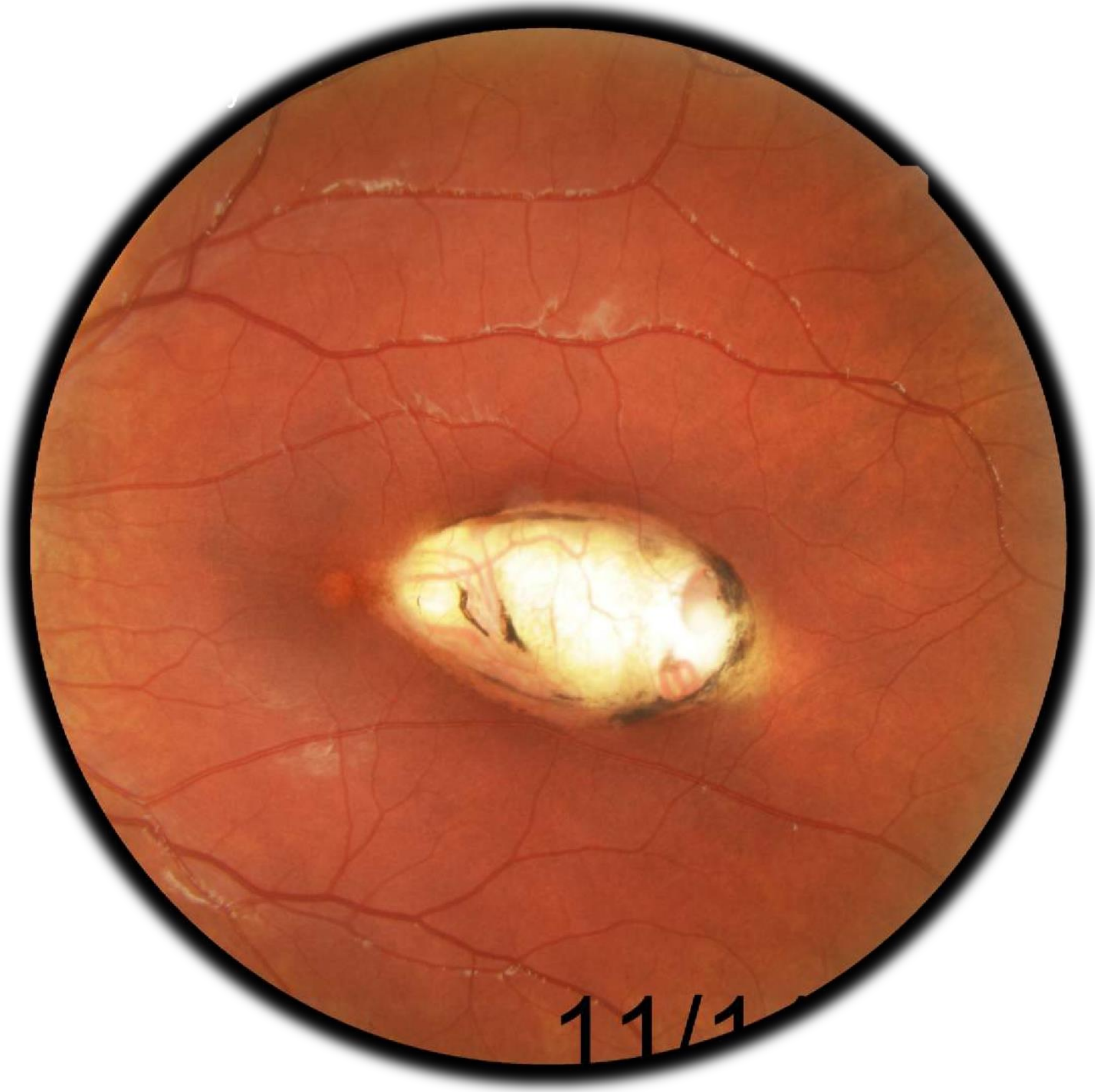

Patient U - November 2013, Left peripheral hypopigmented lesion with irregular borders with color change in underlying retina/choroid

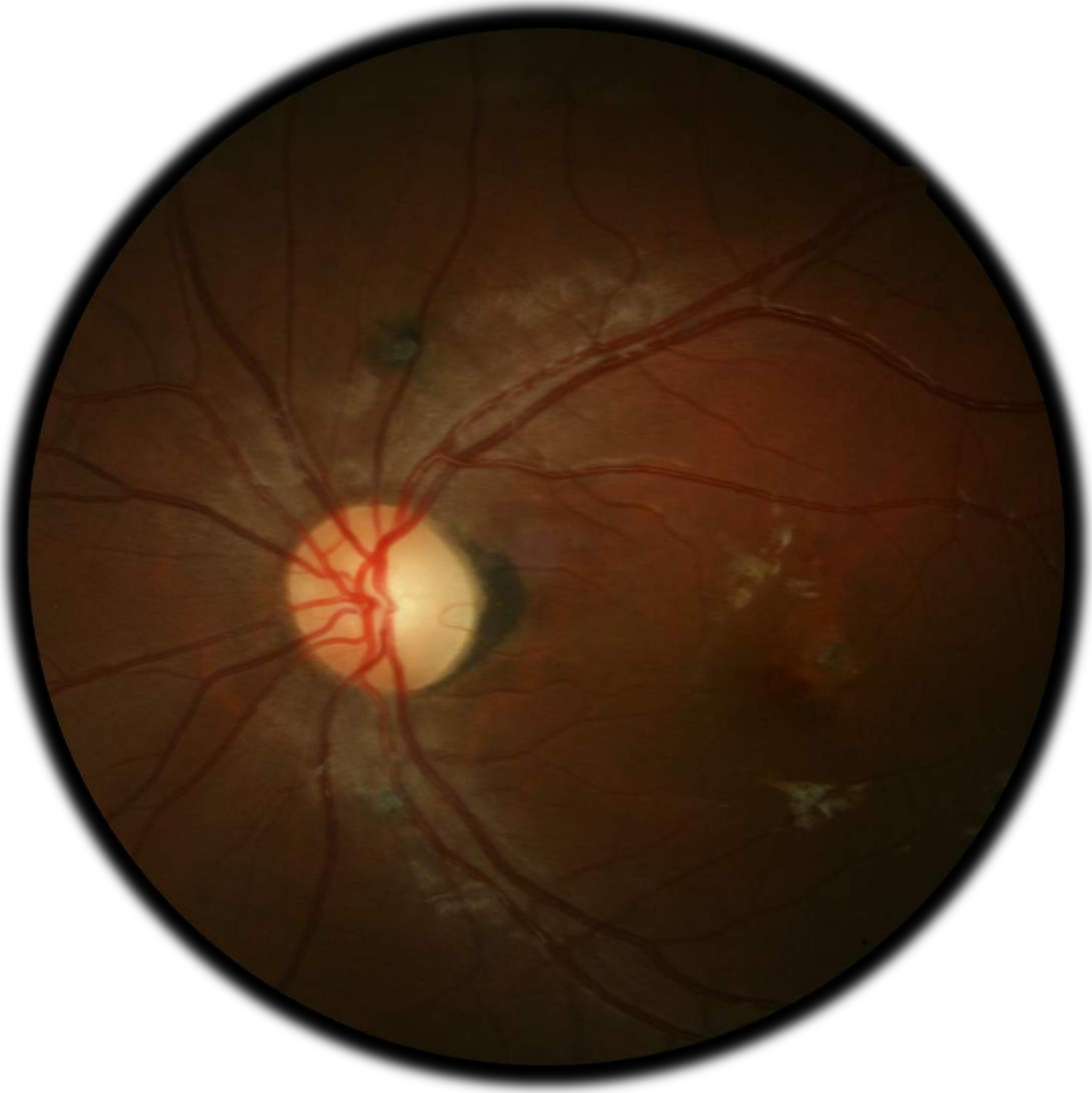

Patient U- Pigmented target, juxtapapillary lesion

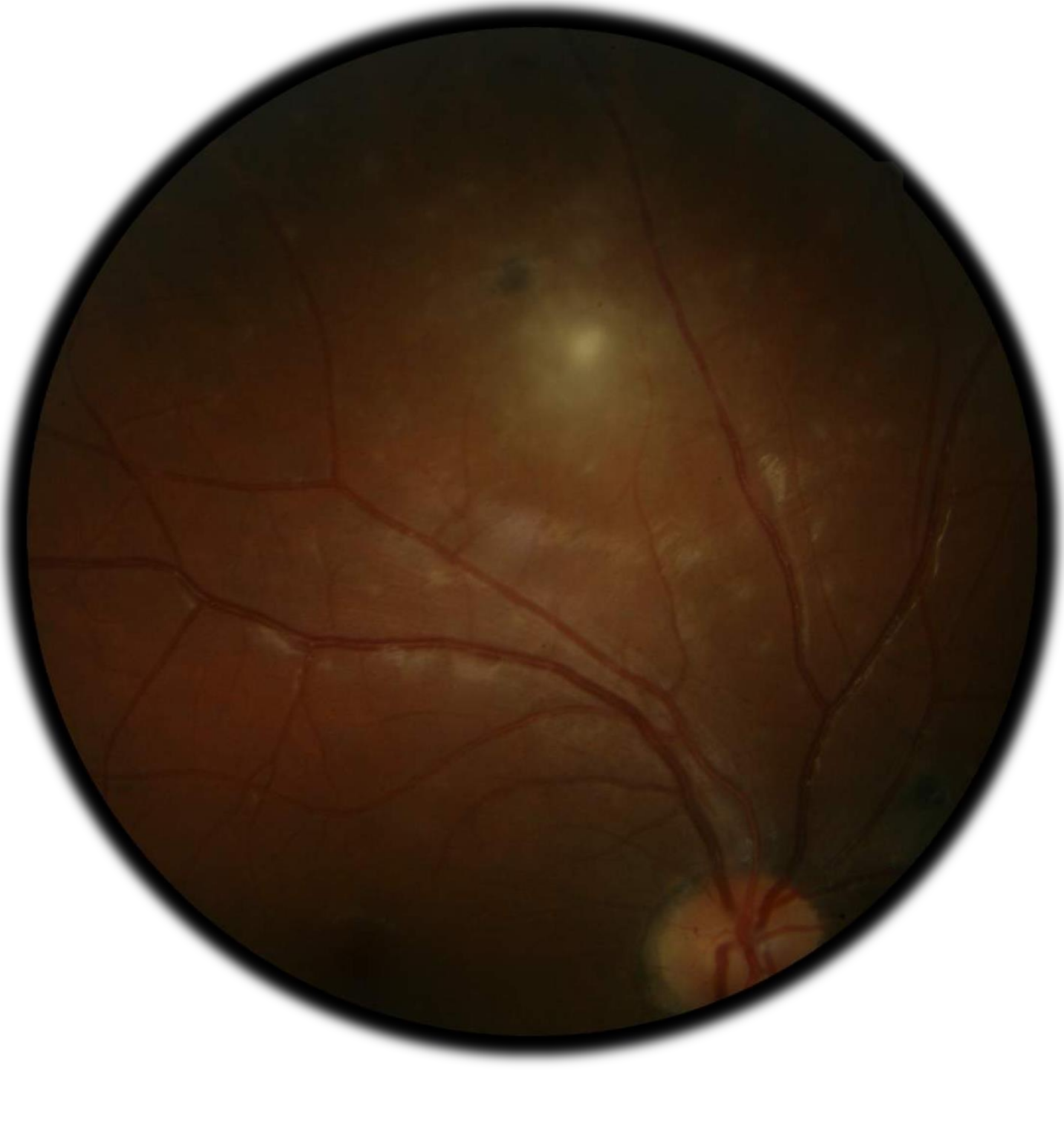

Patient U- Acute fluffy lesion contiguous to an old scar

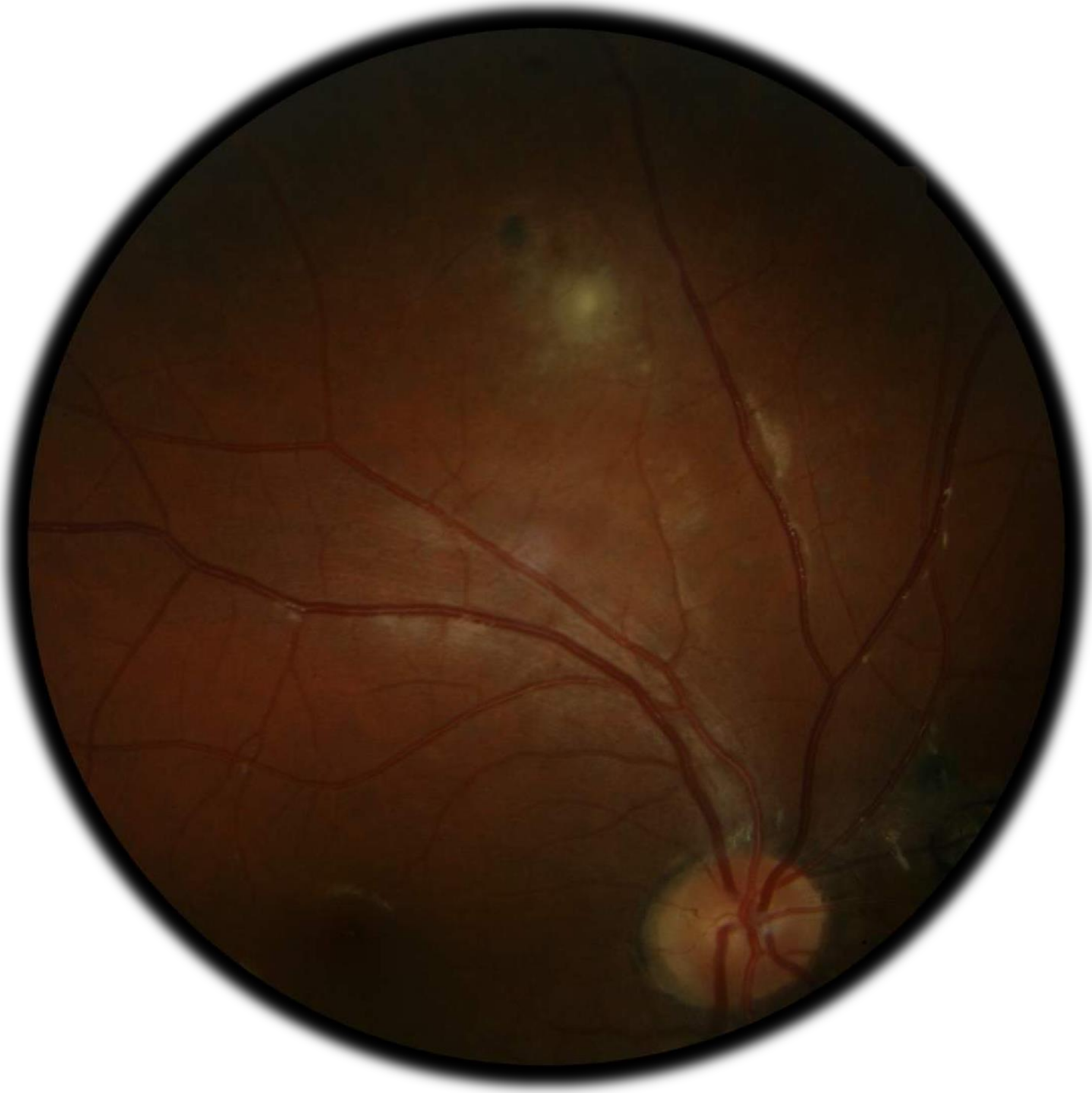

Patient U- Acute, peripheral, fluffy lesion contiguous to an old scar

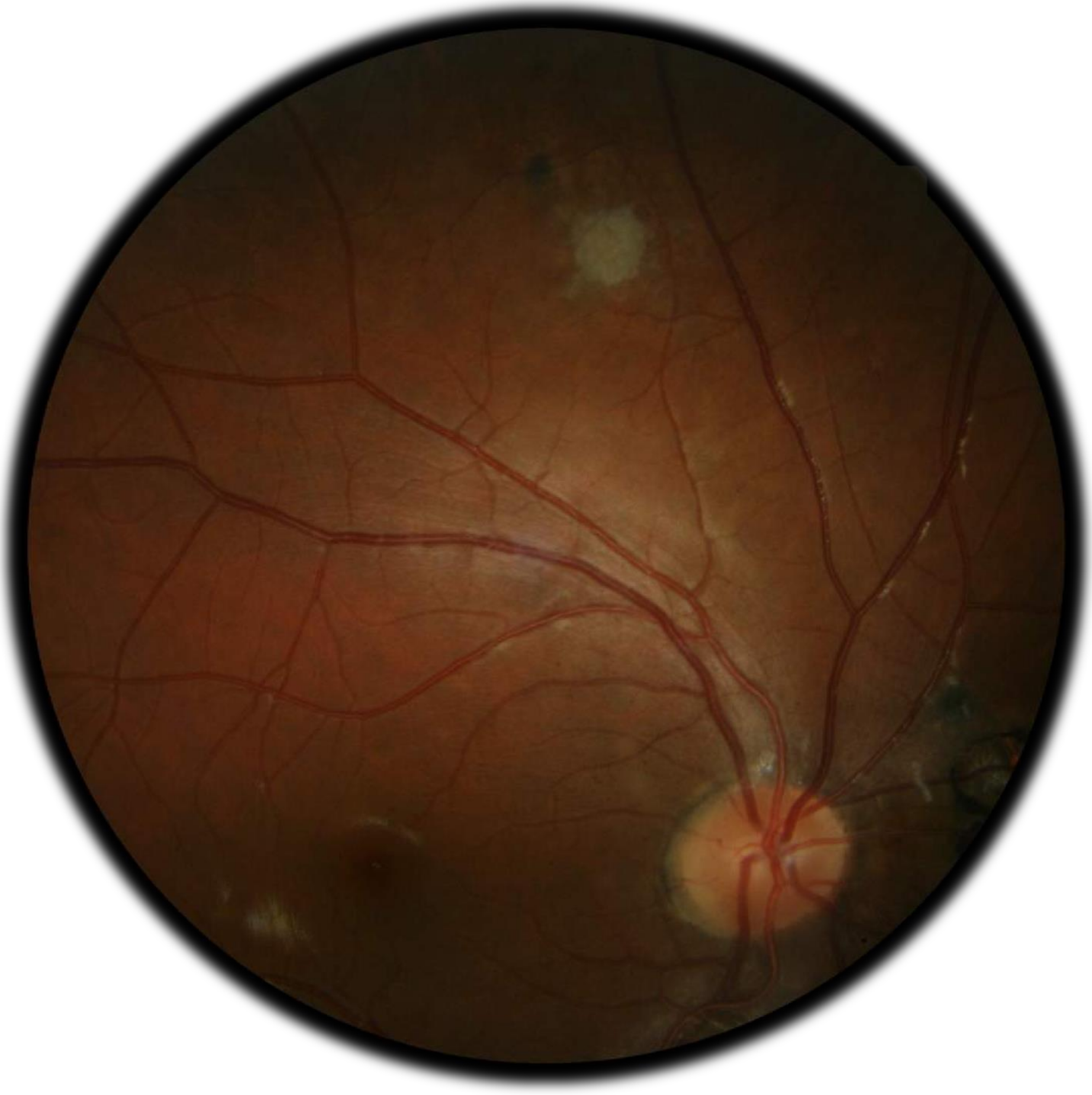

Patient U- Active peripheral lesion post treatment, has distinct borders.

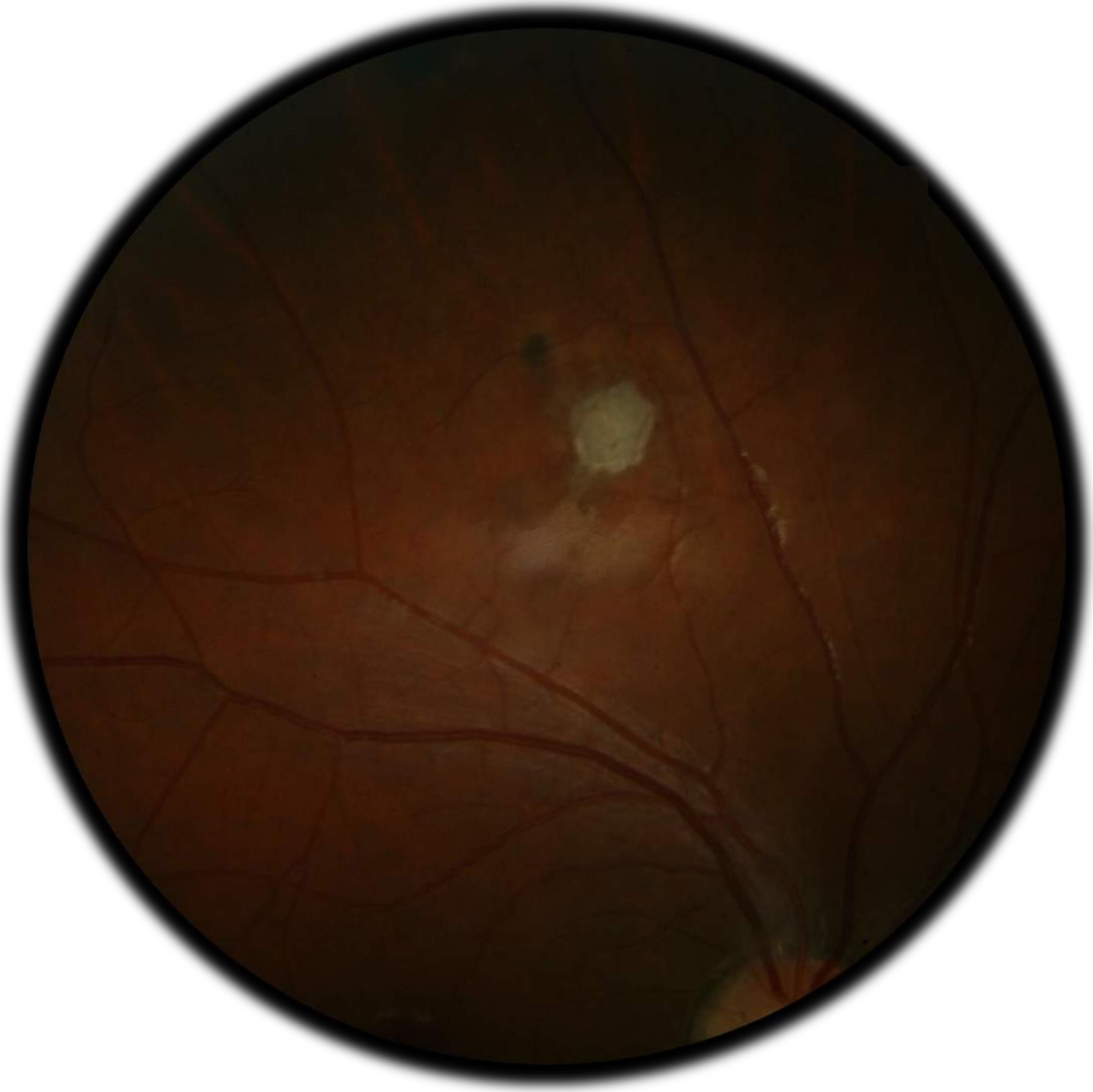

Patient U - Active peripheral lesion post treatment, has distinct borders.

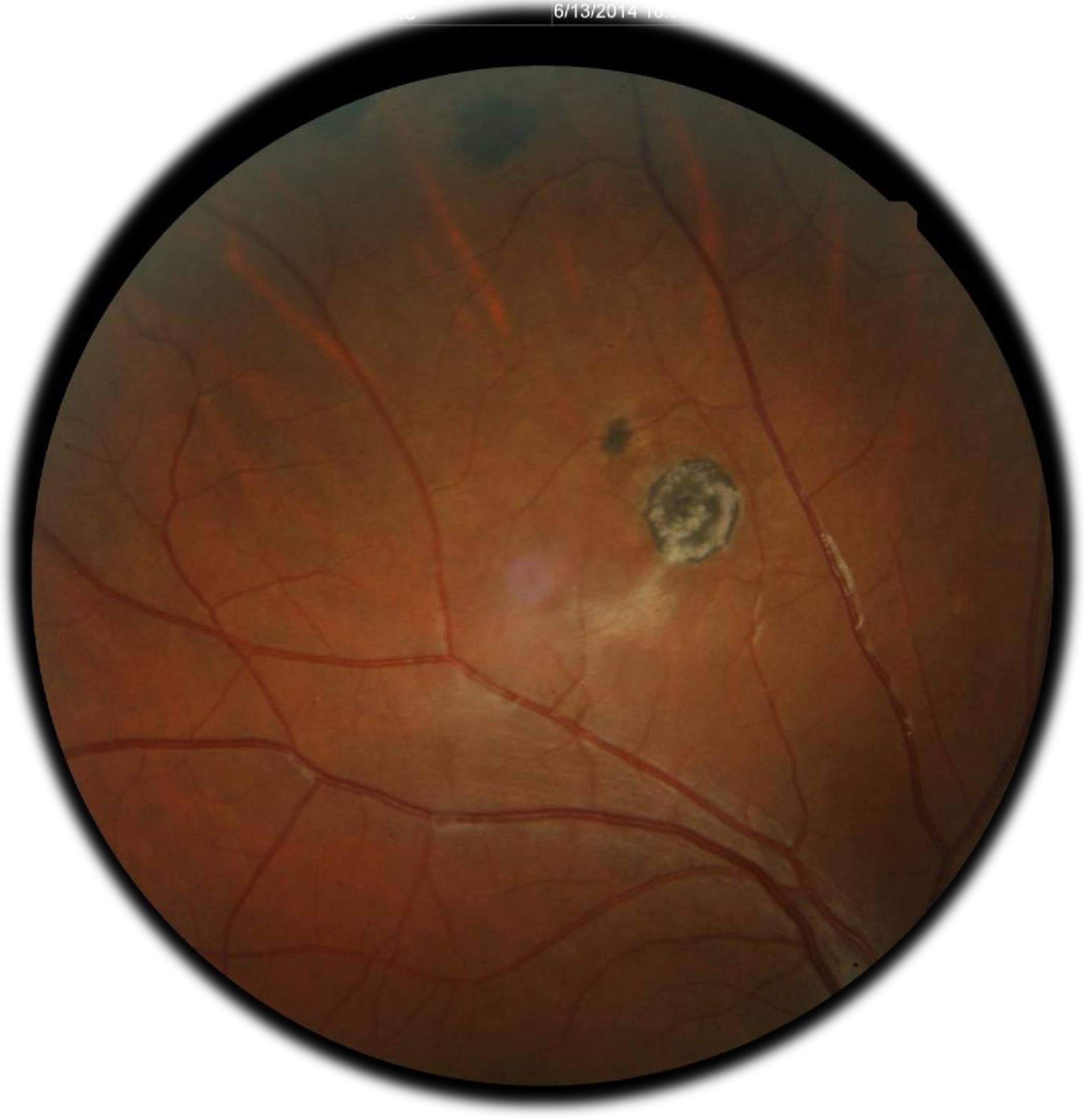

Patient U – June 2014, 1 year post treatment, scar has become pigmented, with some fibrotic tissue

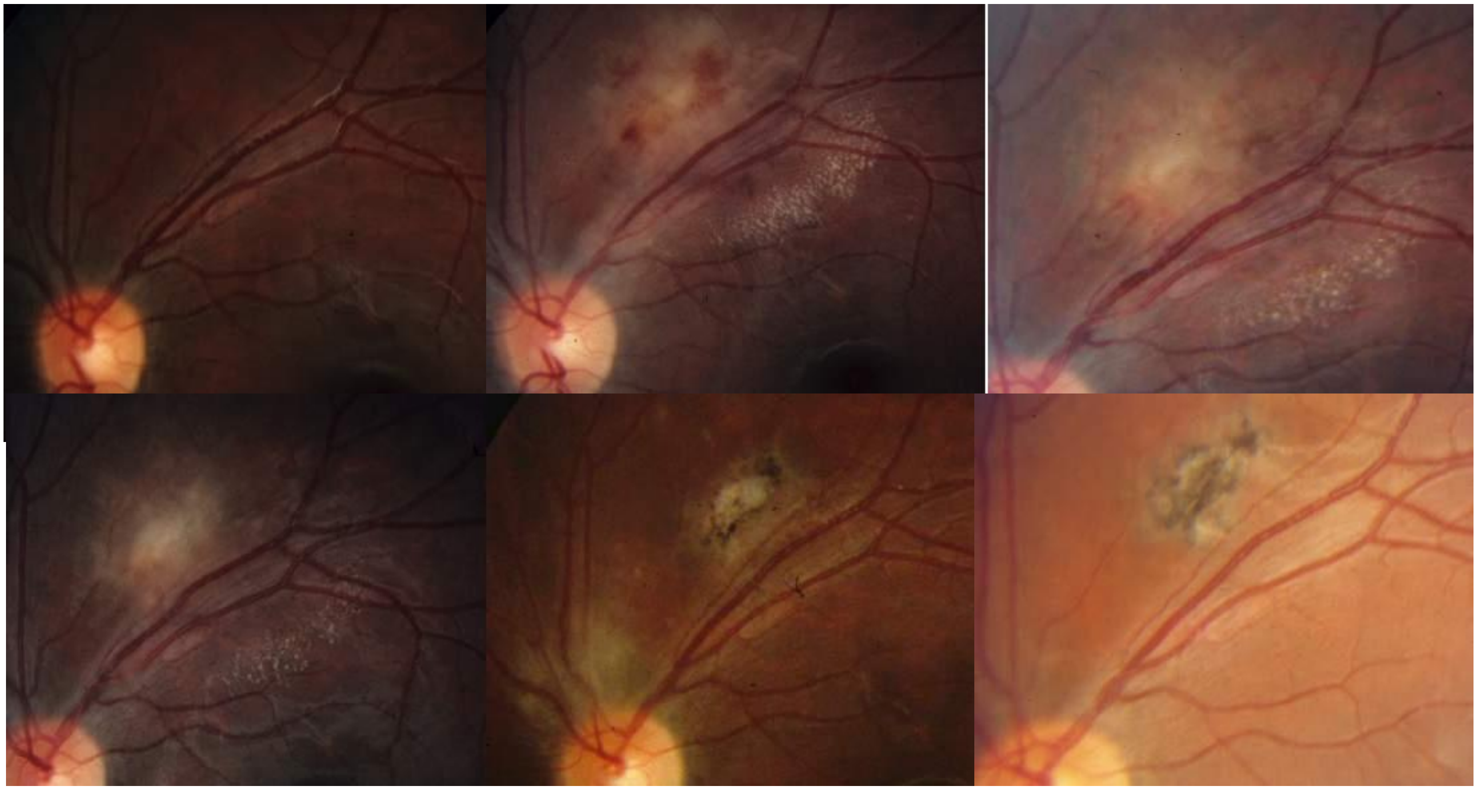

Evolution of a new lesion over time
Top Left (10/19/94): No sign of lesion.
Top center (8/26/96): Some patches of hemorrhage and white infiltrate above the major temporal arteries, and a trial of small dotted, yellow lesions in between the macula and major temporal arteries.
Top right (9/10/96) and Bottom left (10/4/96): The white infiltrates as seen previously is likely to be an active lesion with fluffy appearance and indistinctive borders.
Bottom Center (6/8/98) and Bottom right (5/21/02): Inflammation resolved and lesion became more hyperpigmented overtime.

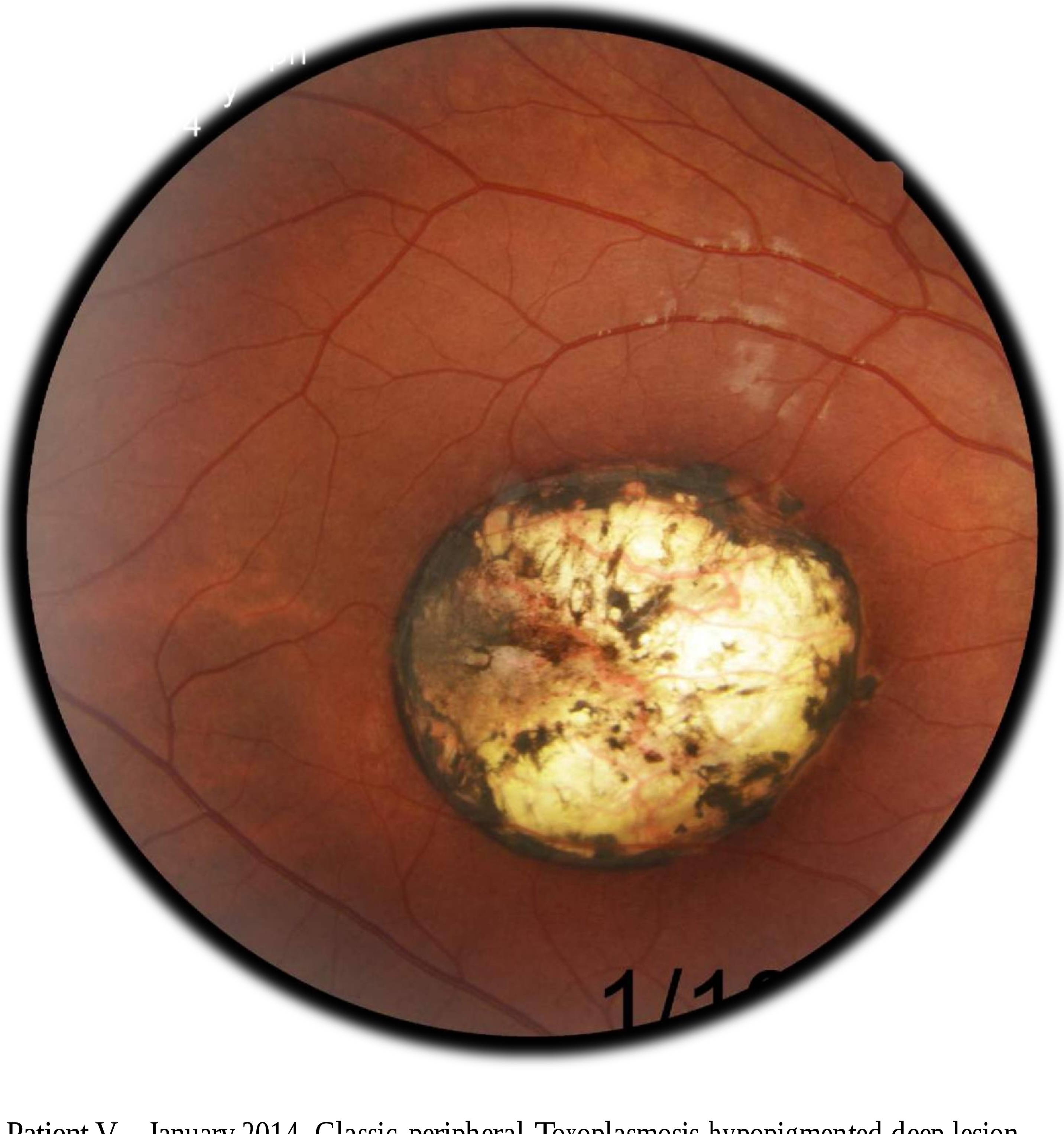

Patient V – January 2014- Classic, peripheral Toxoplasmosis hypopigmented deep lesion

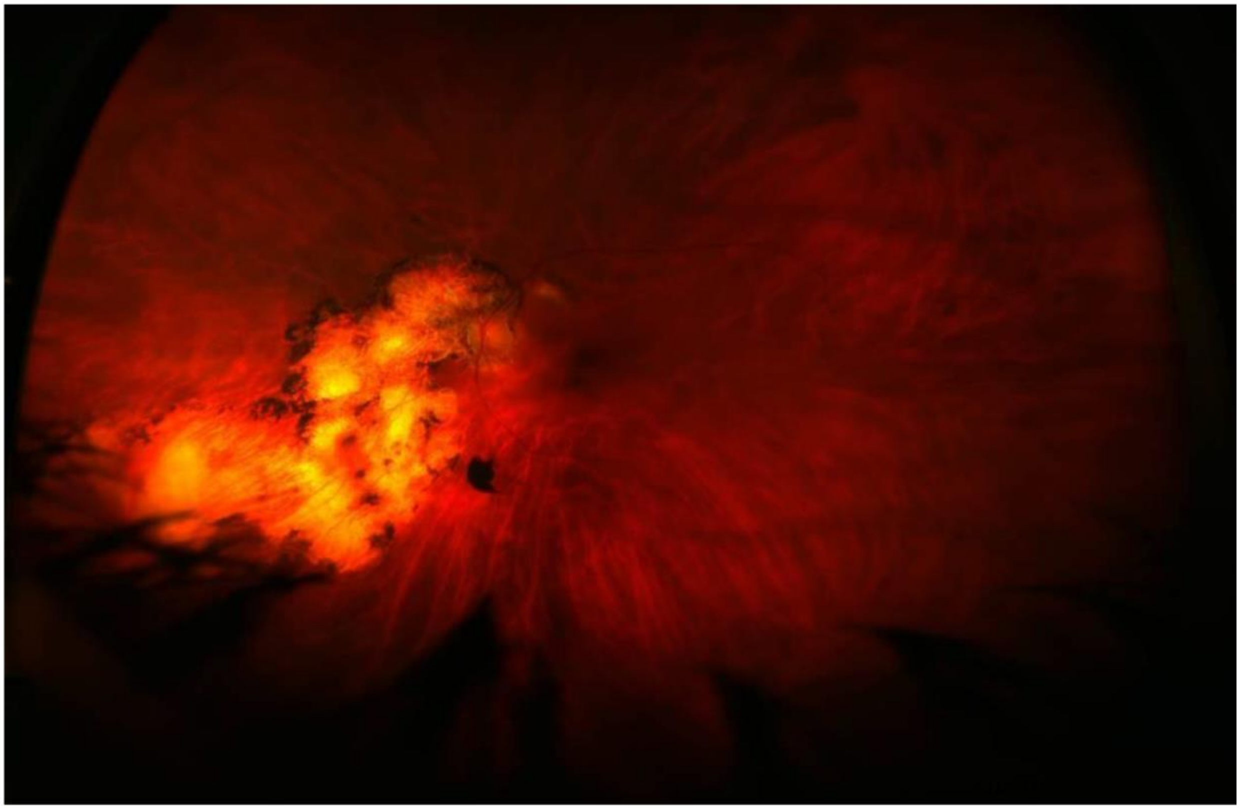

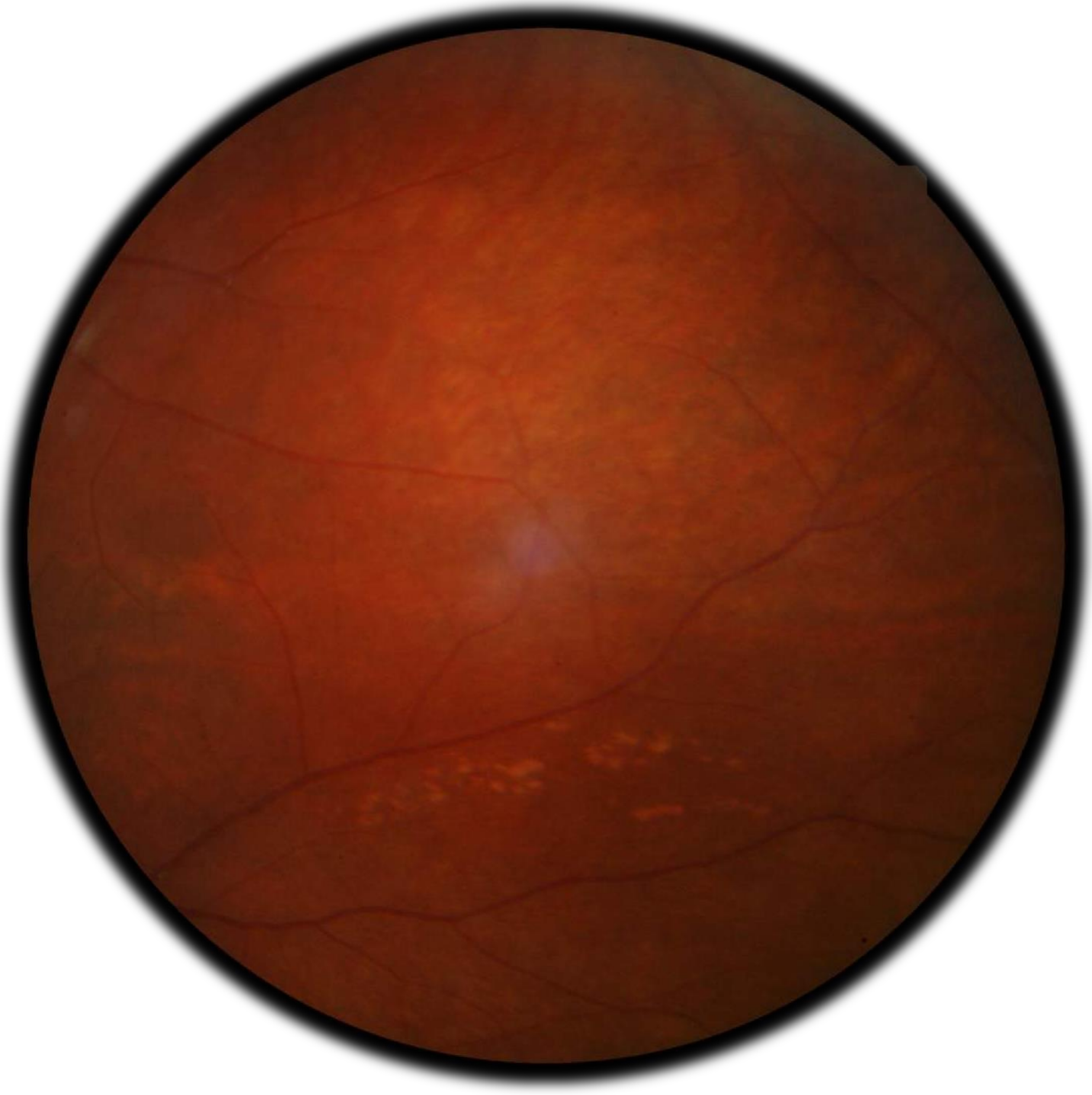

Patient V – February 2014, Left periphery

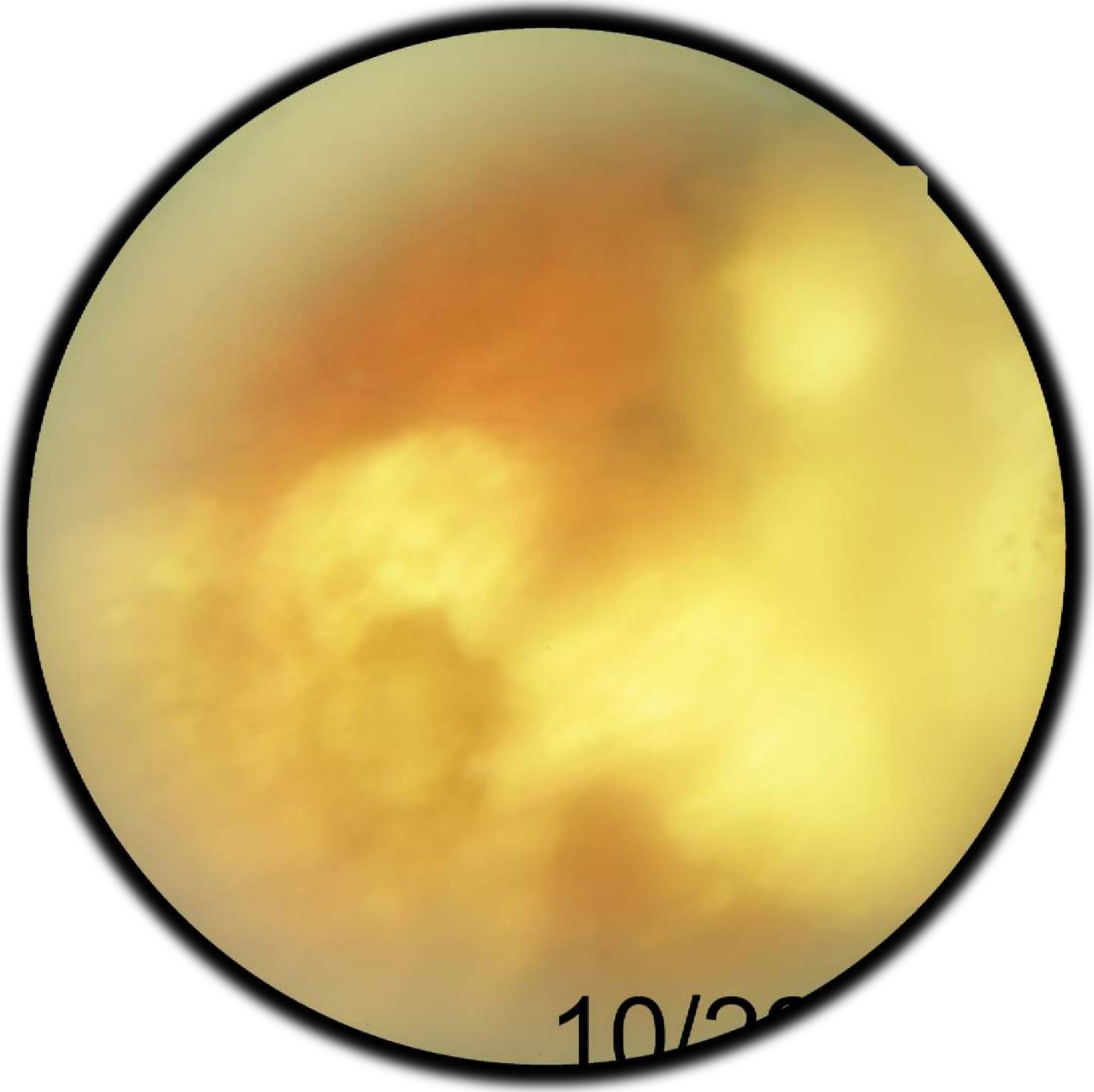

Patient W - active, acute reactivation with haze, Waxing and waning reactivation of chorioretinitis in left eye for 12 months with significant vitritis, vitreous condensation, debris, fibrils, and vitreous veils and macular edema associated with chronic inflammation. 4 active peripapillary chorioretinal lesions- creamy, elevated, edematous, and hypopigmented. Severe inflammatin makes the image blurry.

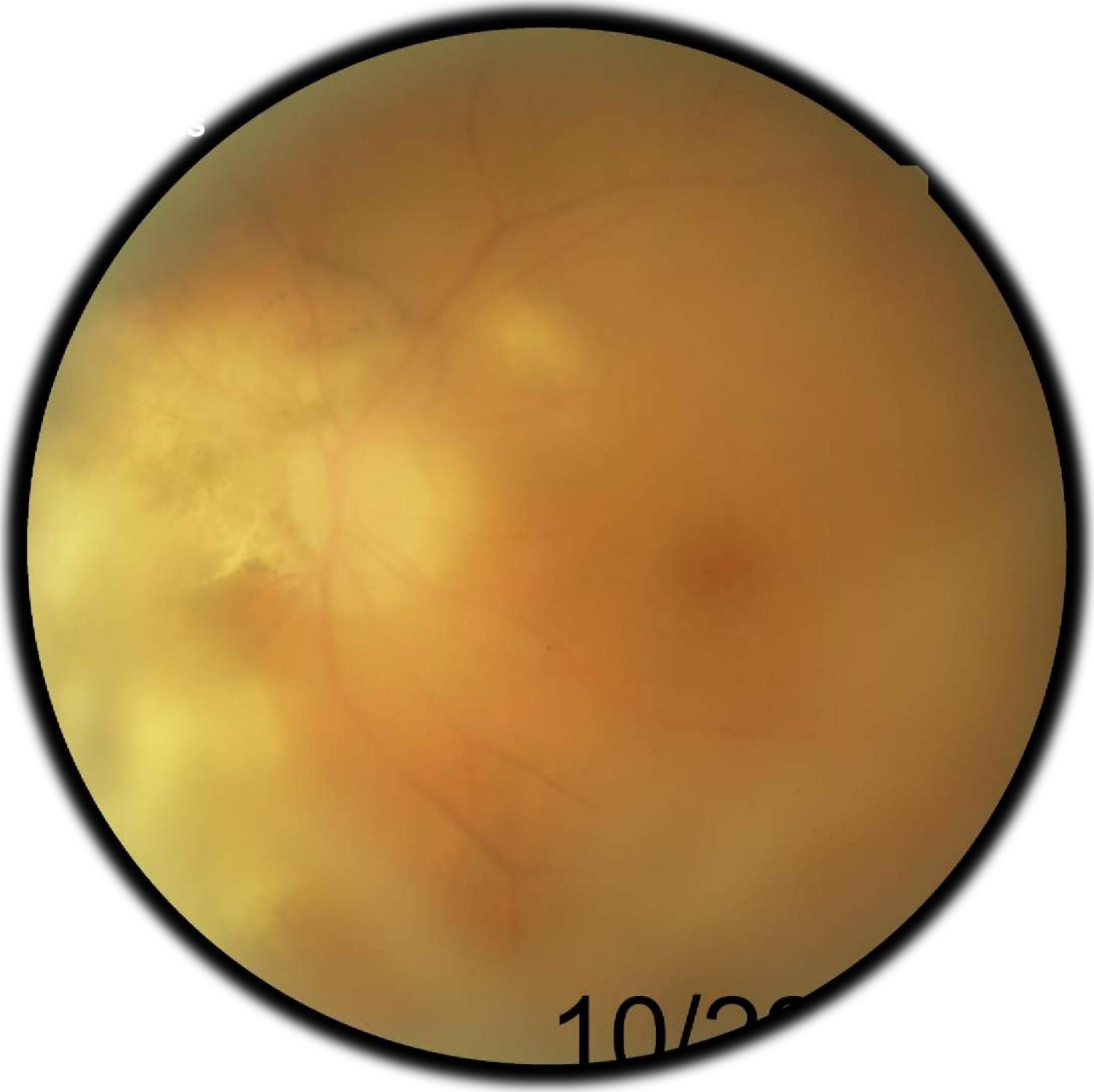

Very active, hazy, inflamed

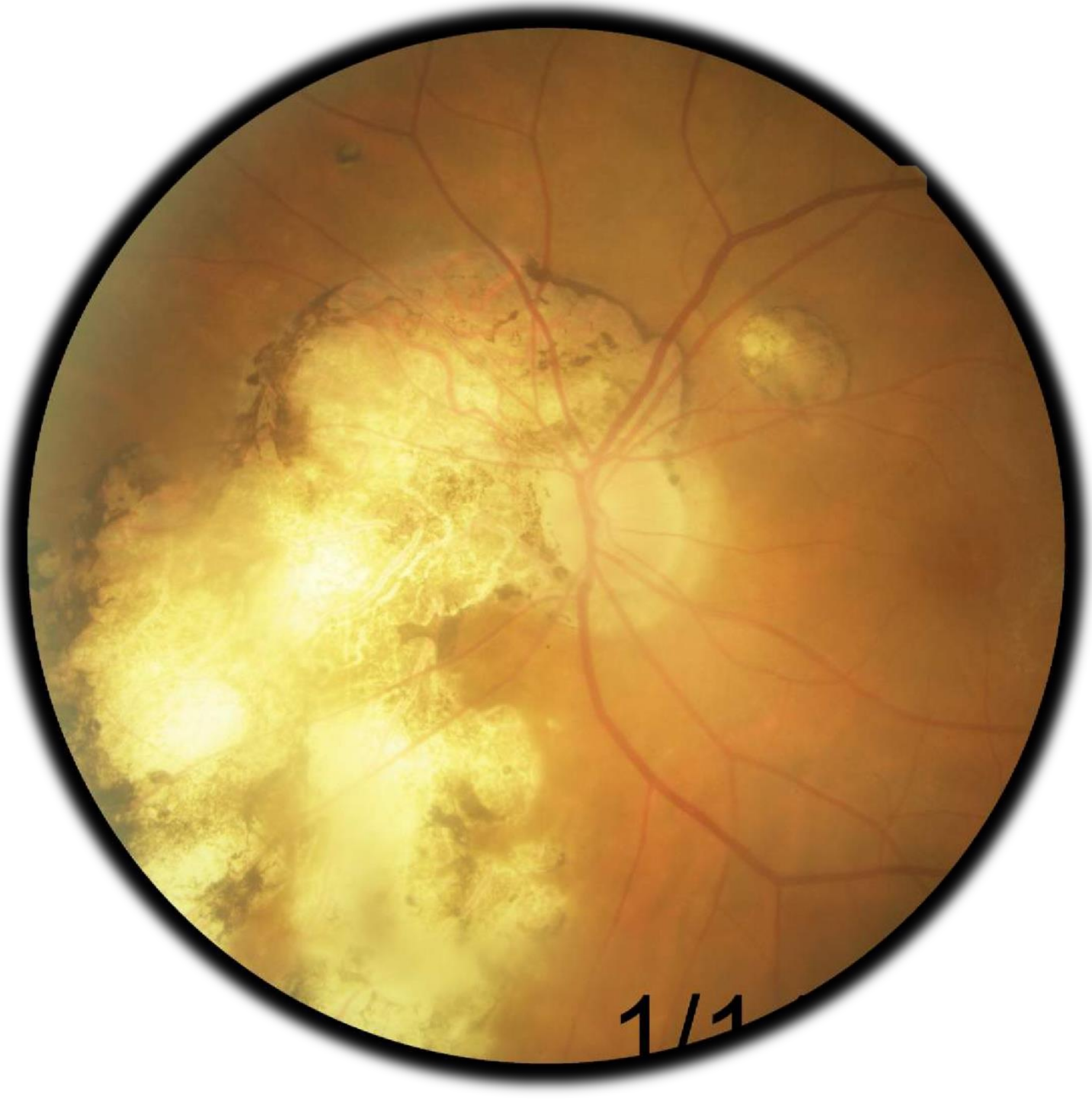

Peripapillary, reactivation, active, inflamed

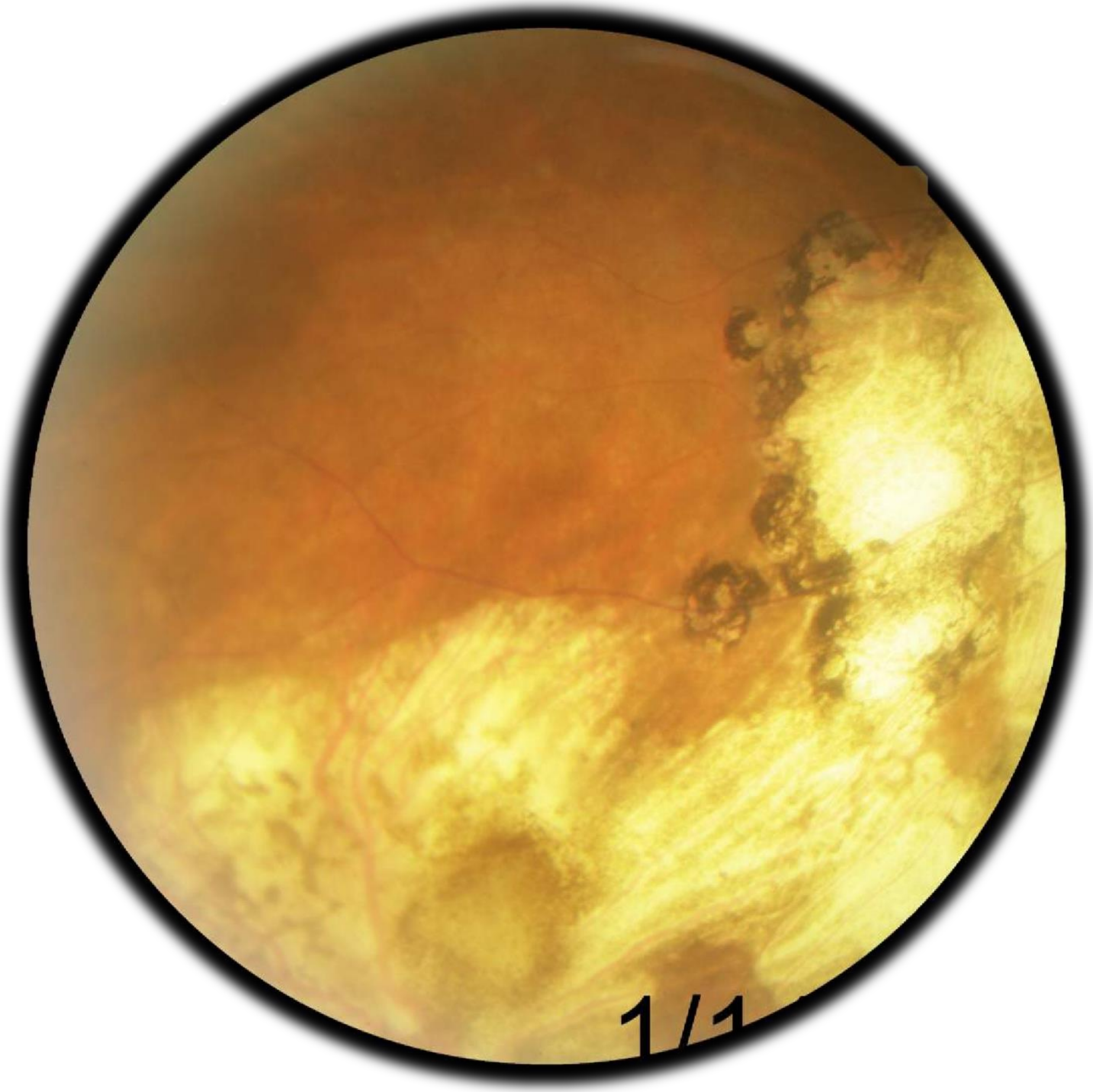

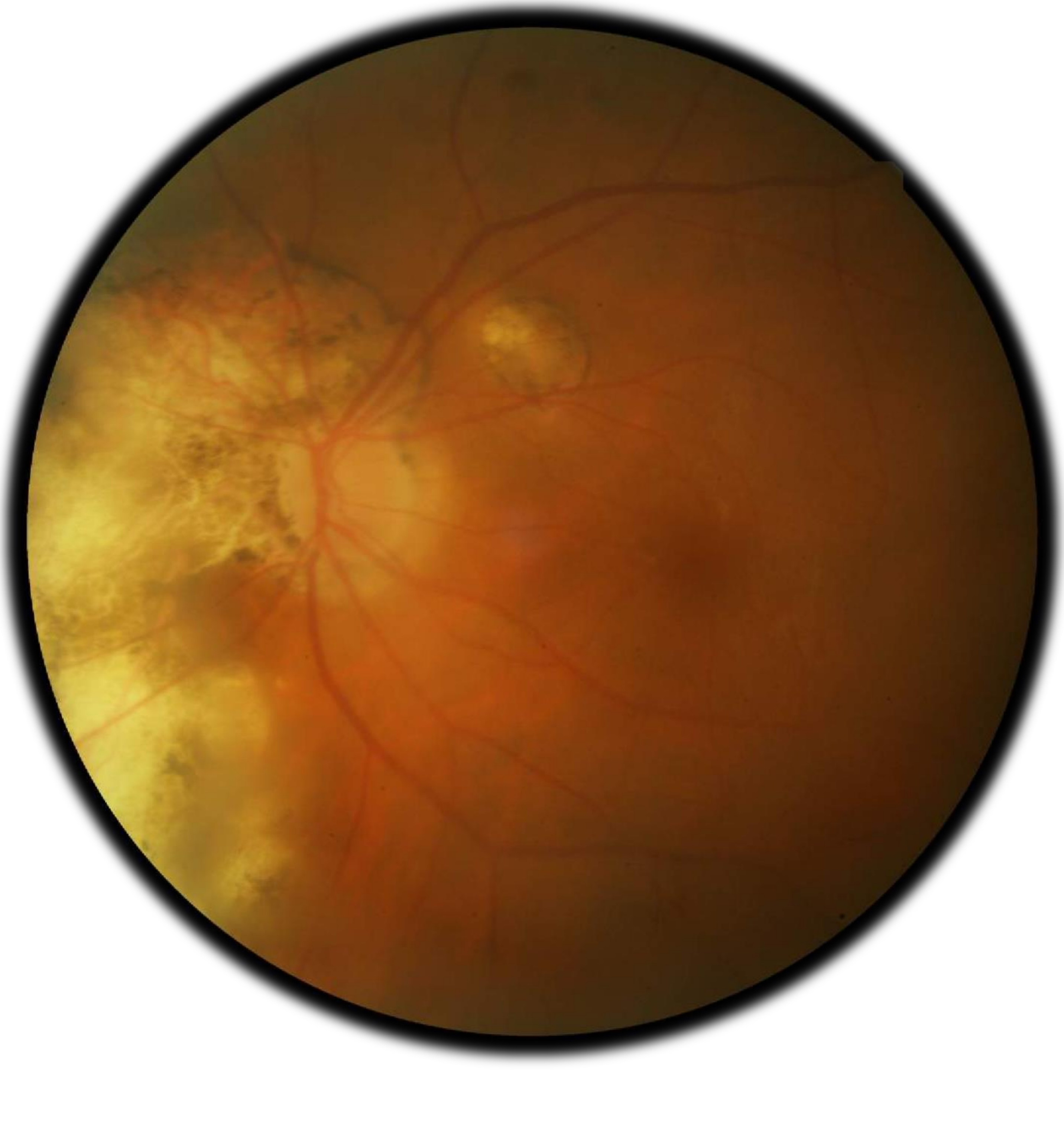

Patient W December 2012

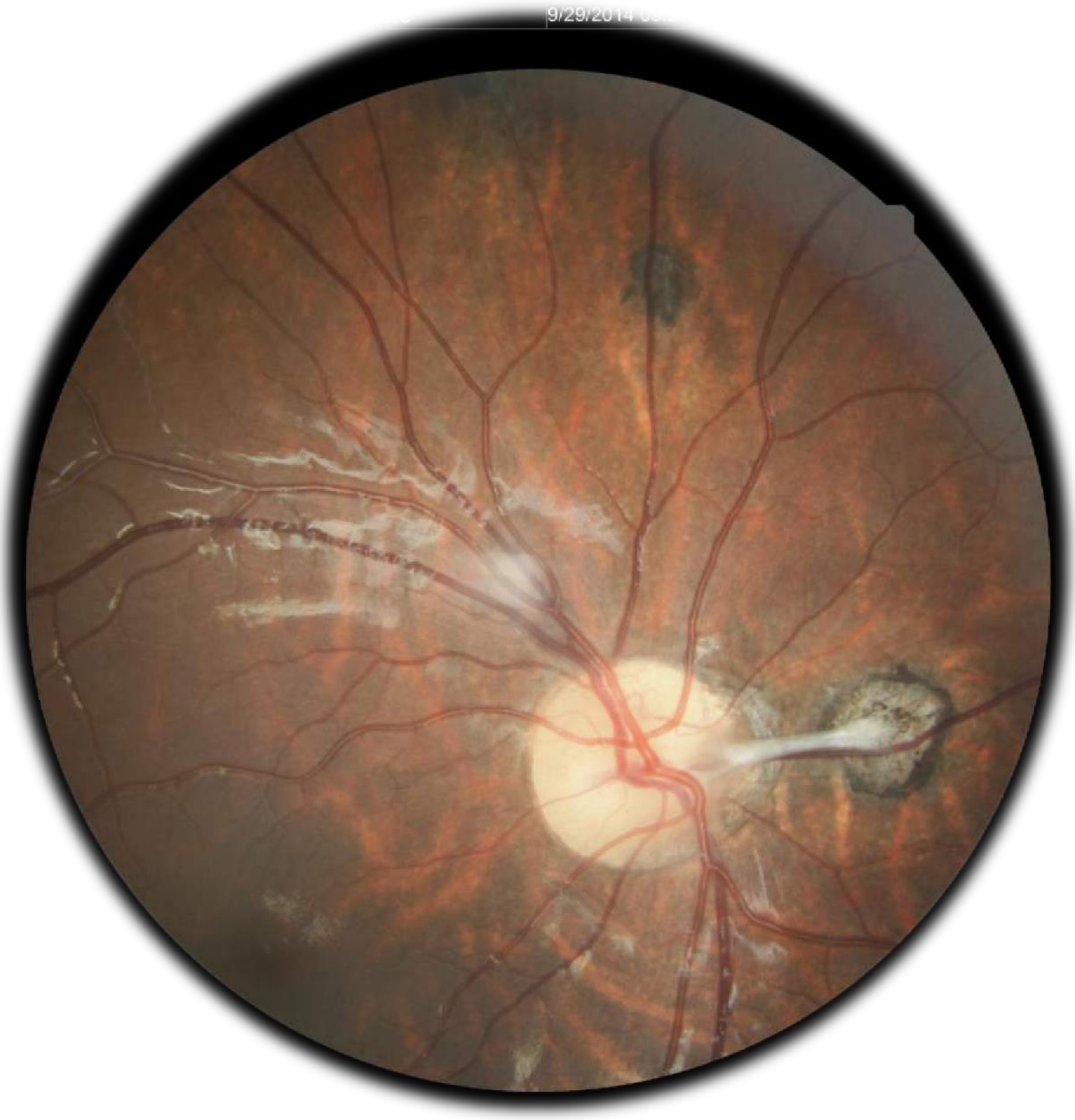

Patient X- Congenital toxoplasmosis, treated in utero for 7 weeks. Born with two inactive lesions, had a reactivation at 8 weeks of age. Was then treated for the first year of life. On three and a half year old exam:

□Right eye: Thin juxtapapillary, mildly pigmented chorioretinal lesion along the nasal margin of the optic nerve extending from approximately 1 o'clock to approximately 5'oclock.

□Moderately sized, chorioretinal lesion in the nasal quadrant slightly inferior to the horizontal raphe along a retinal vessel which comes off the optic nerve at 4'oclock and runs slightly inferior and nasal. The lesion has five sides with sharp, pigmented margins surrounding a lighter, grey central area. It is very close to the optic nerve; however it is not contiguous to the juxtapapillary chorioretinal lesion along the nasal margin of the optic nerve. There is a long, thin strand of white fibrosis extending from the neural rim of the nasal part of the optic nerve and splaying out in the center of this chorioretinal lesion superior to the retinal vessel which is overlying the lesion. The strand of fibrosis appears to be in the vitreous and is not subretinal.

□One moderately small darkly pigmented oval chorioretinal lesion located in the near periphery in the 1'o clock meridian- superior to the superior nasal vascular arcade.

□A very small pigmented, round chorioretinal lesion in the near periphery. Approximately 1 or more disc diameters superior and more temporal to the optic nerve in the 12 o clock meridian. Able to detect this lesion because child was looking at a toy in up gaze.

□Fibrovascular proliferation

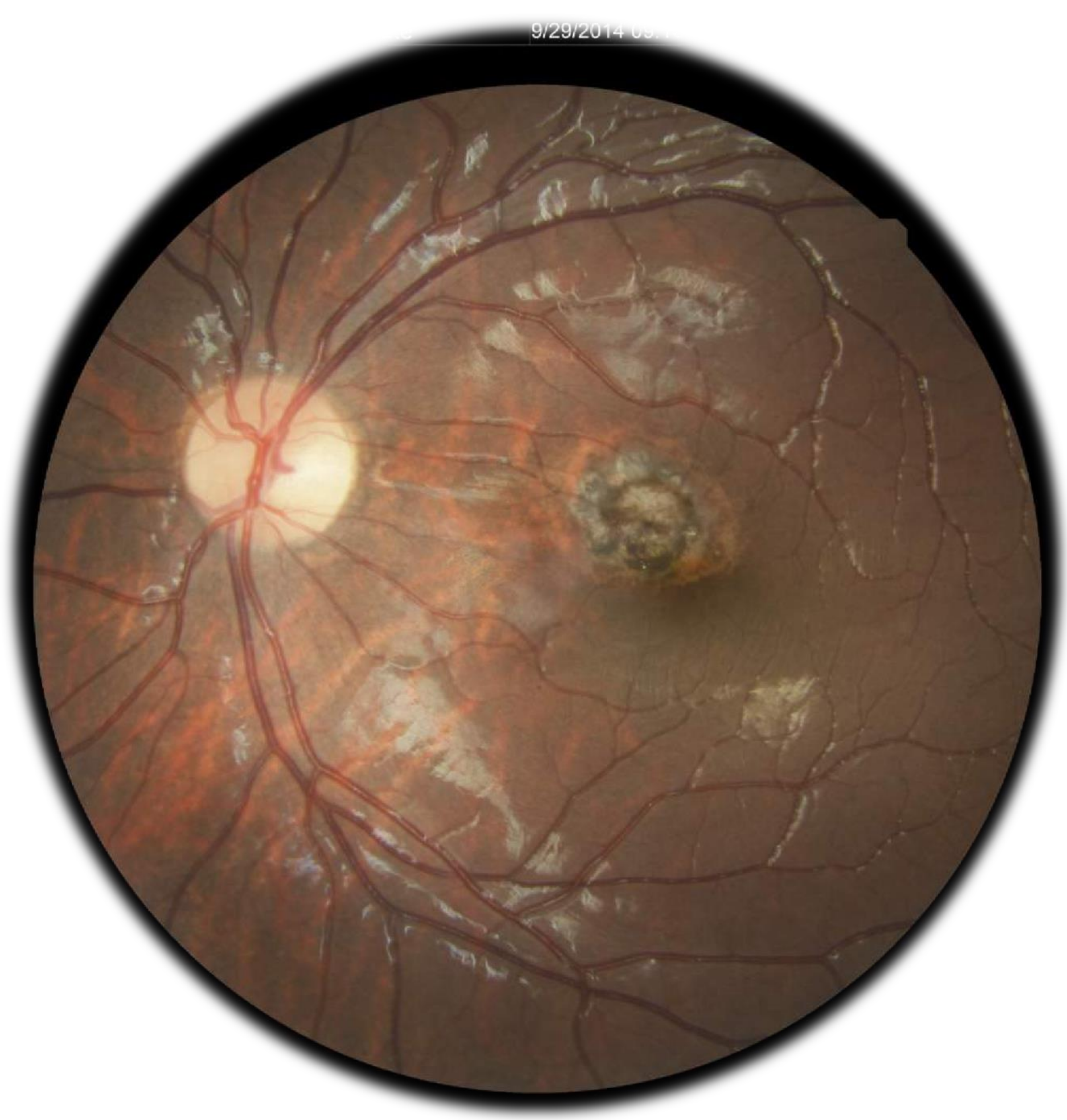

□Left eye: a moderately sized, oval chorioretinal lesion in the macula which is lighter centrally and surrounded circumferentially by a thin area of hyperpigment. There is a less pigmented and thicker margin surround the lesion in two areas, nasally and
Superiorly. Clinically it is difficult to determine if the lesion spares part of the fovea inferiorly. But on the fundus picture on nidek camera, there is a small portion (approx. 0.20 disc diameters) of the fovea spared inferiorly. The lesion is inactive, it is flat, well circumscribed, no edema and no elevation. This central macular lesion is consistent with congenital toxoplasmosis □

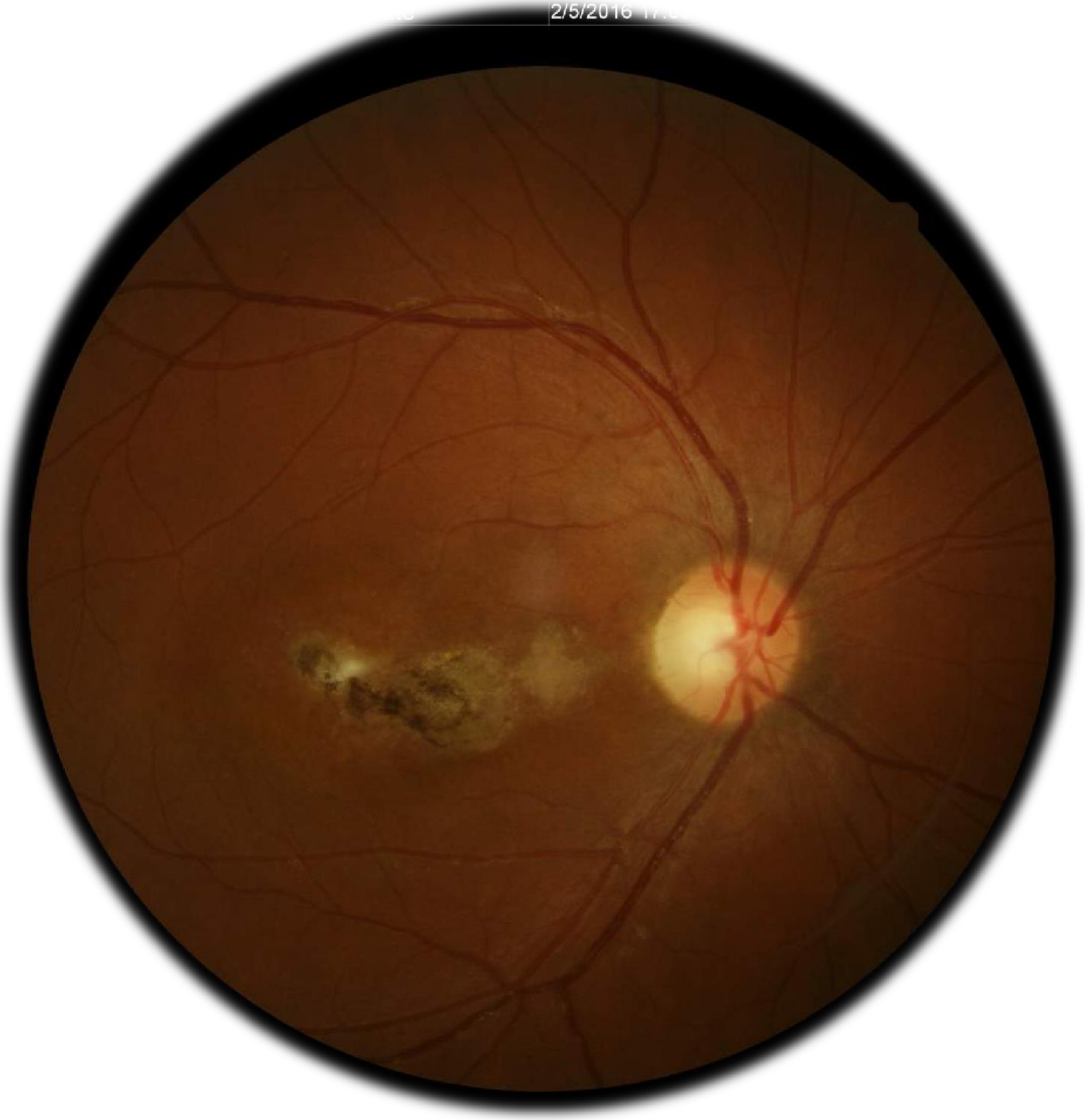

Patient Y, Small, acquired toxoplasmosis lesions with Choroidal Neovascularization

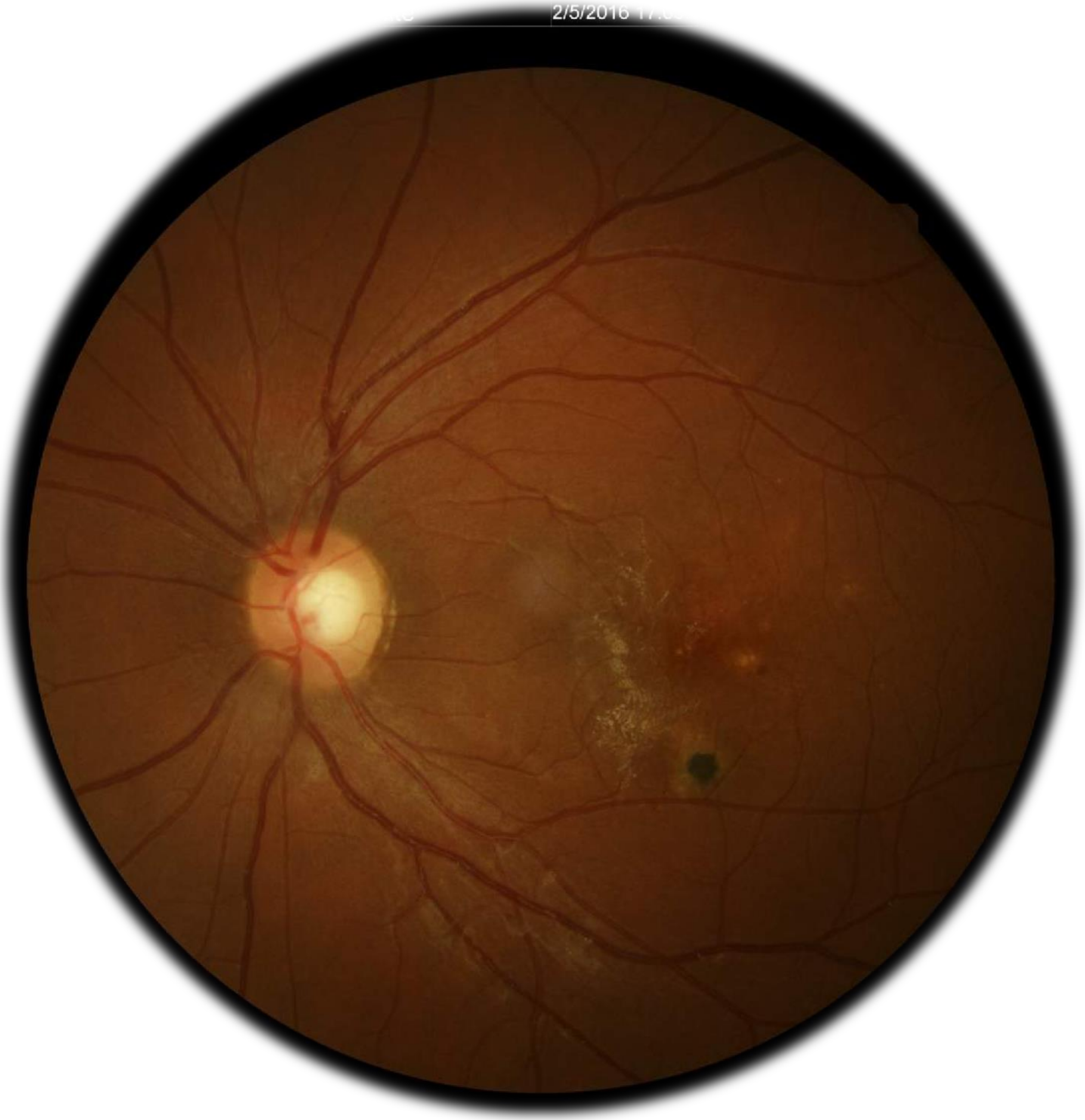

Patient Y

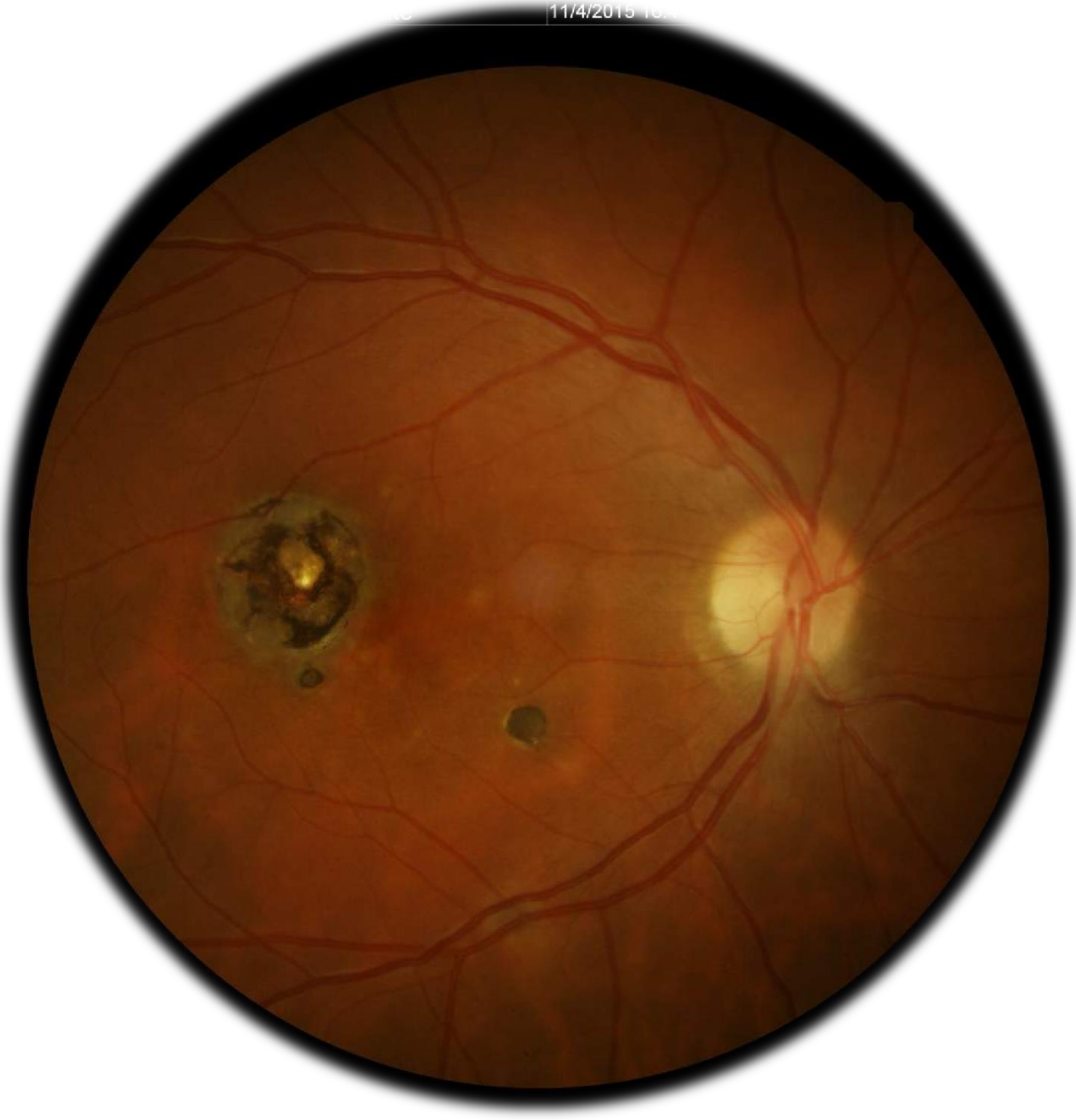

Patient Z

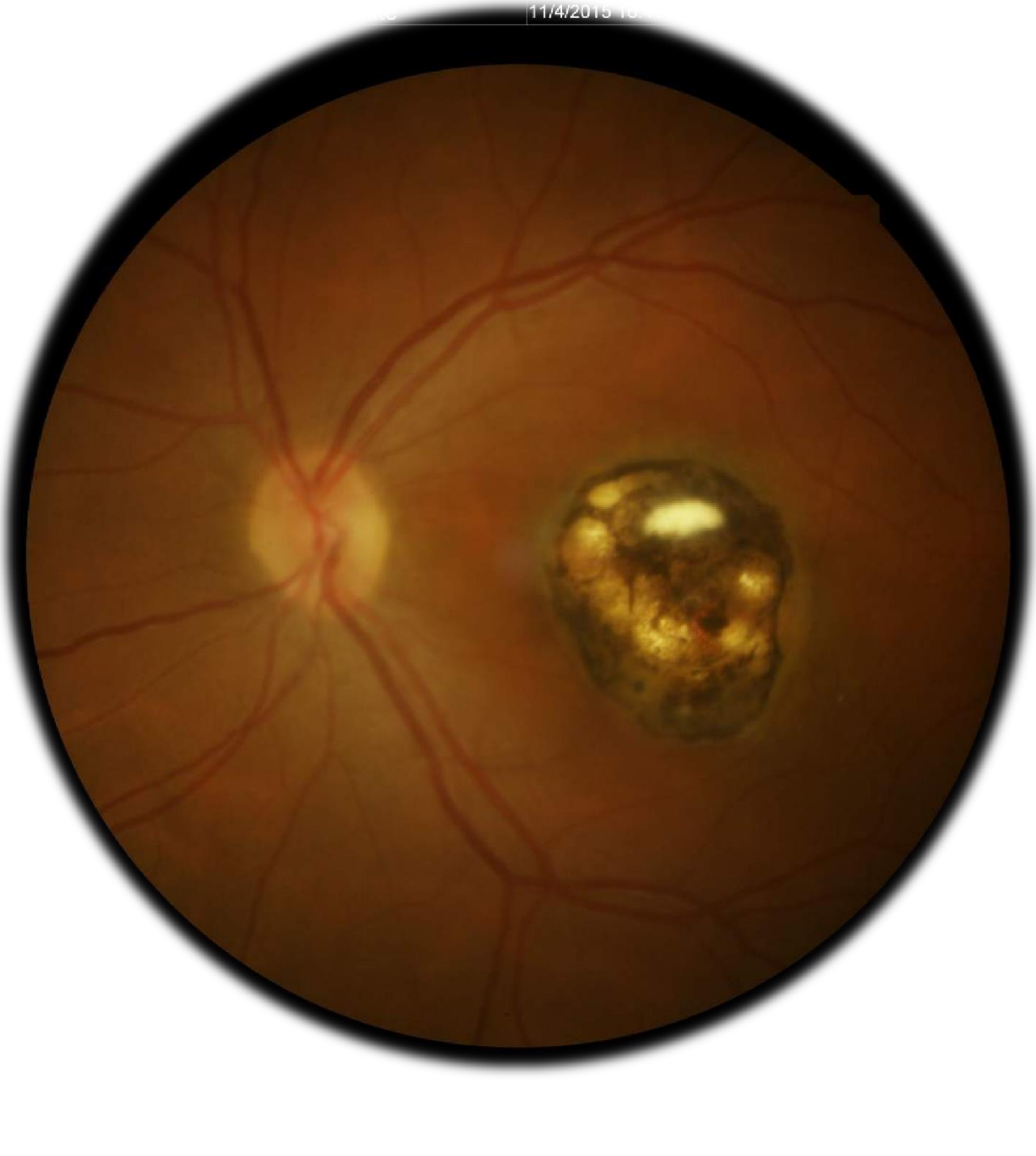

Patient Z

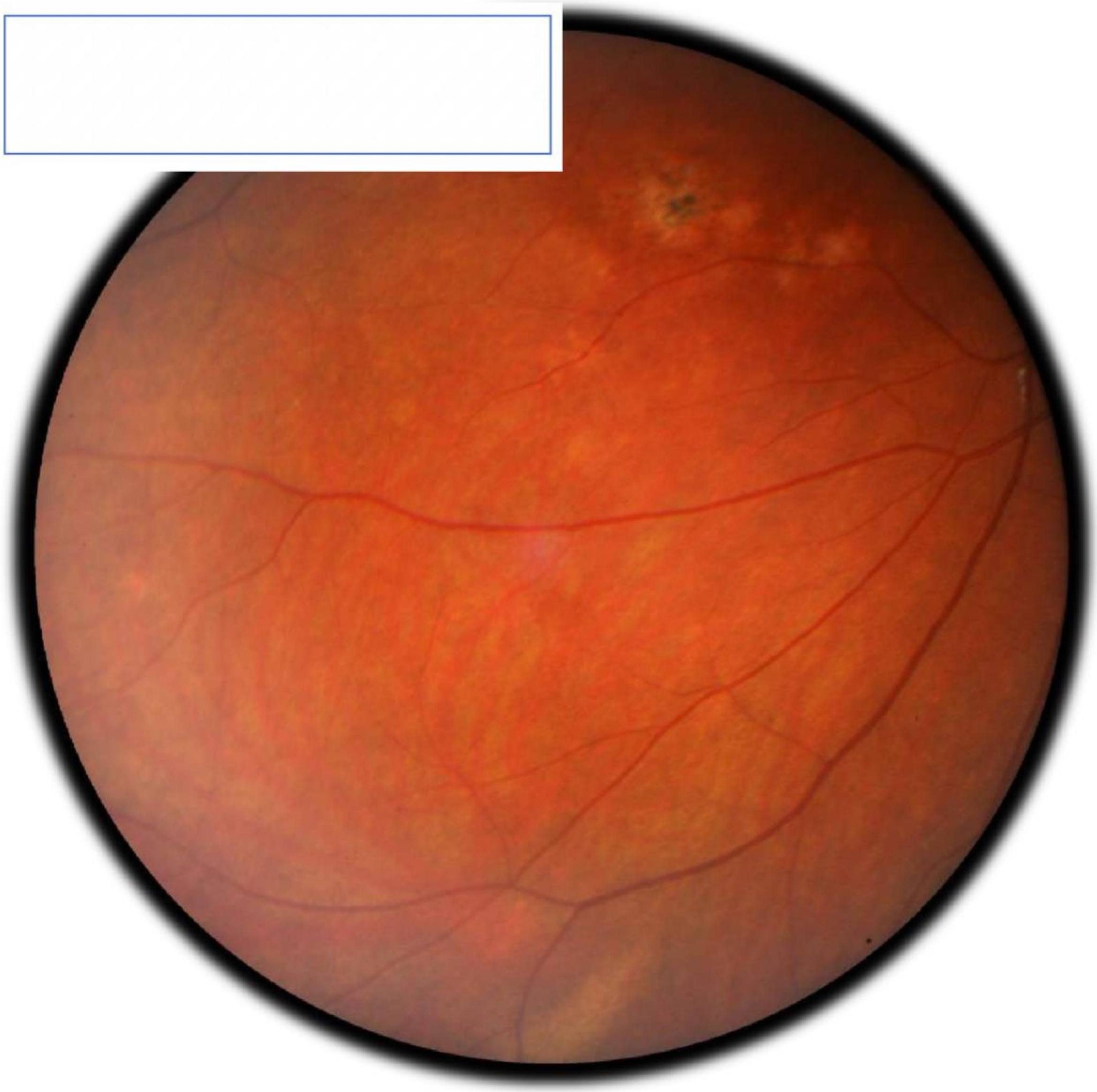

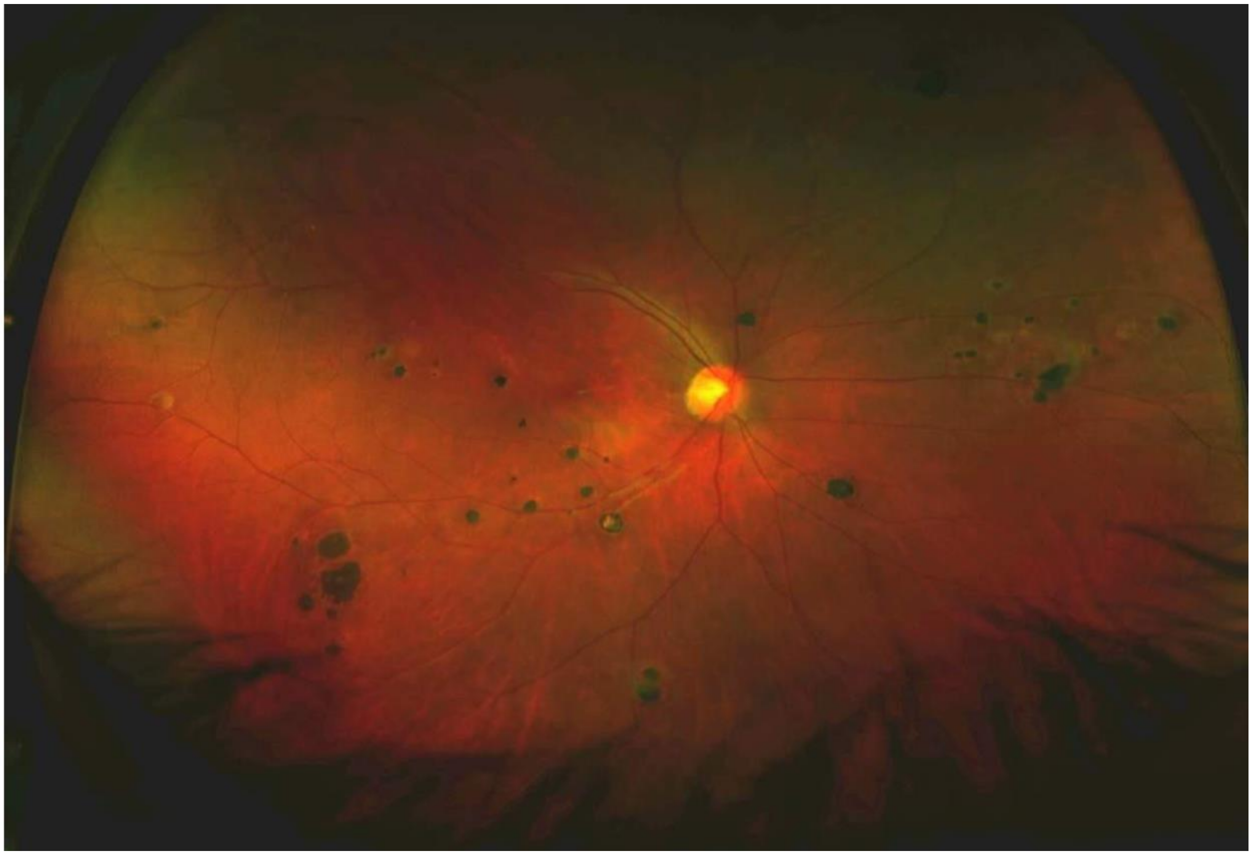

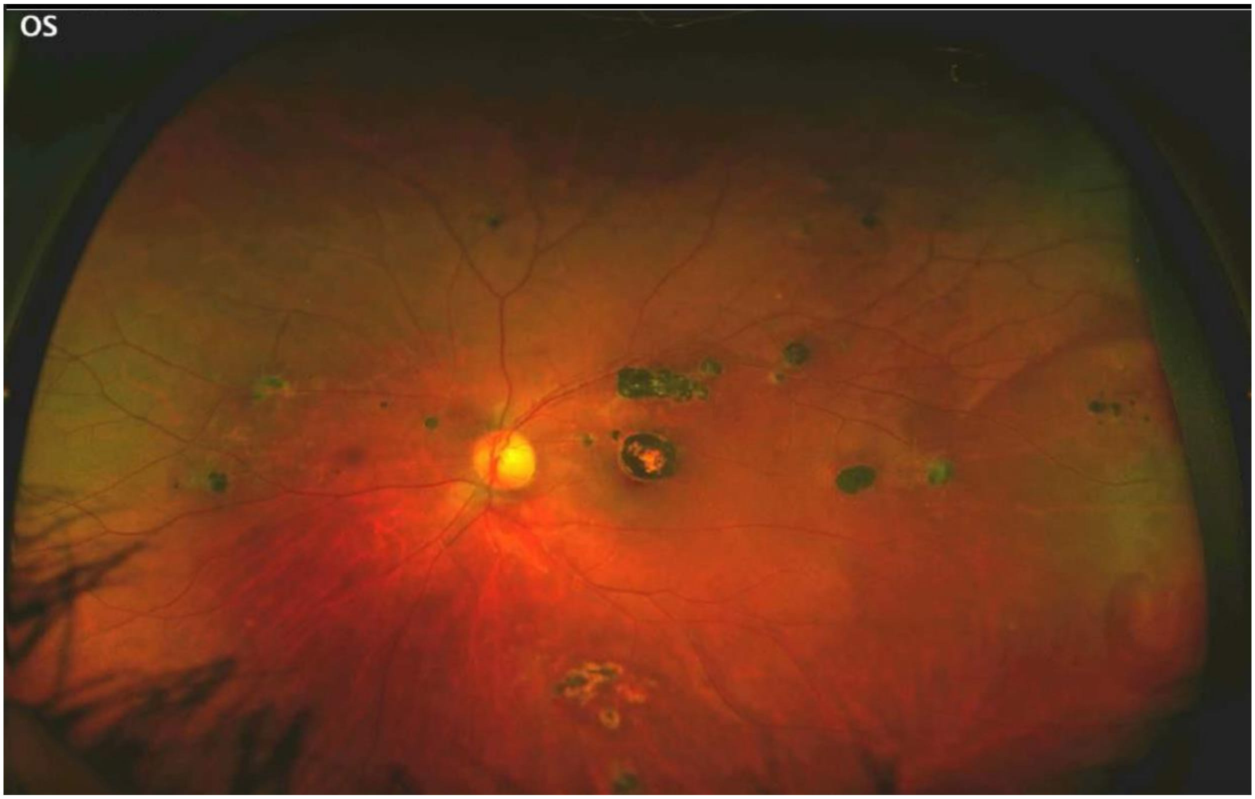
OS

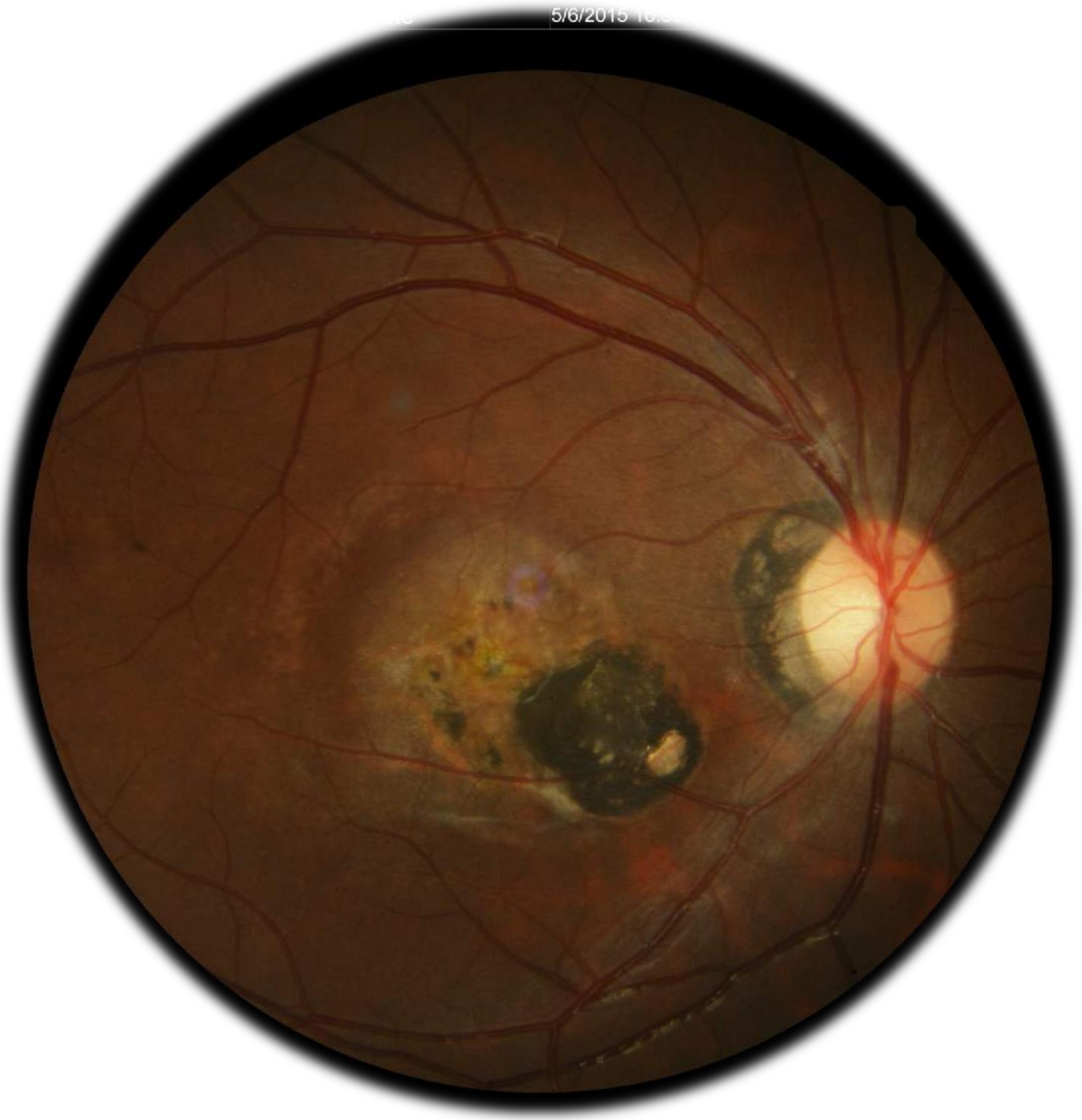

Patient AA: Peripapillary old lesion

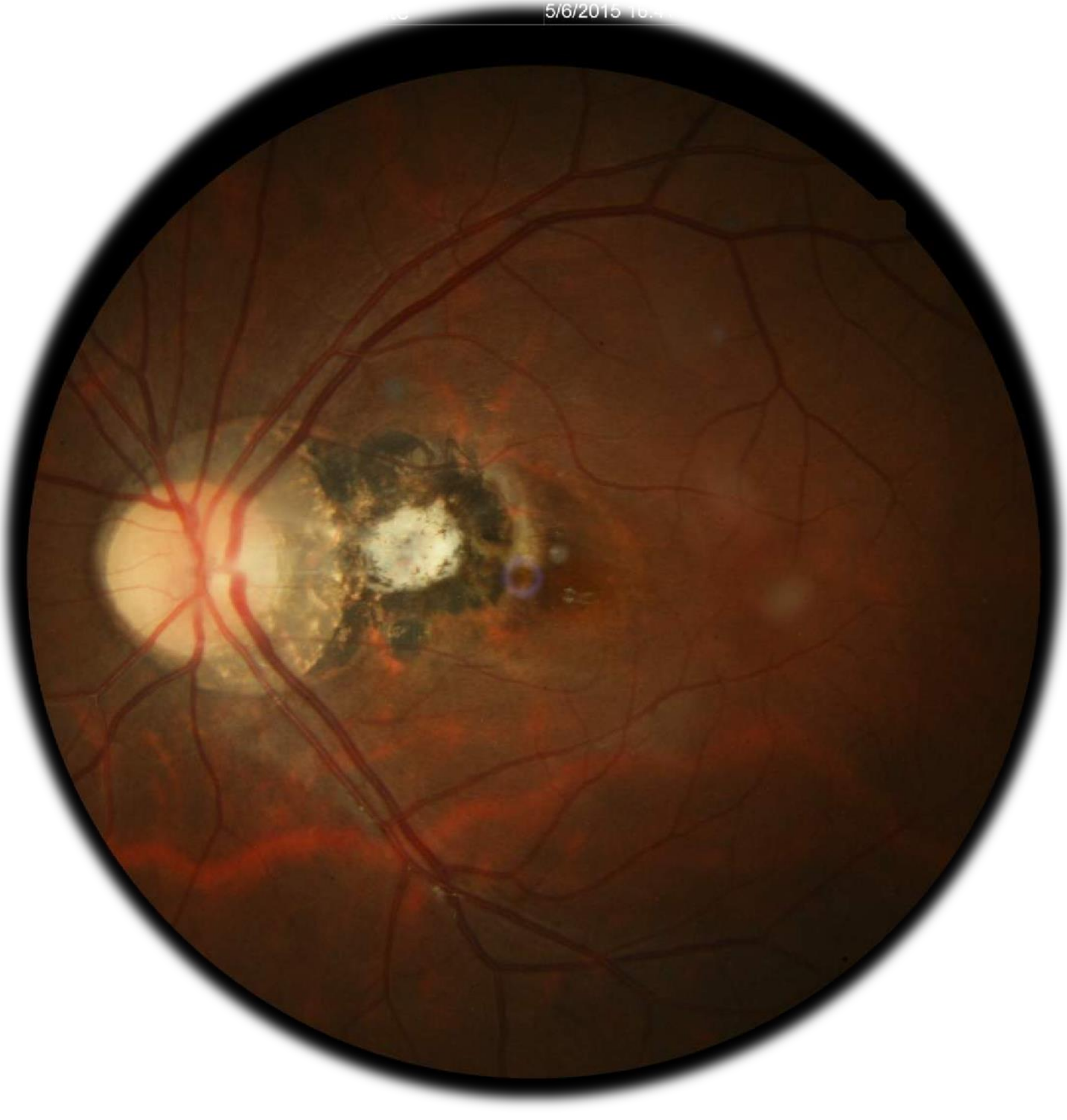

Patient AA

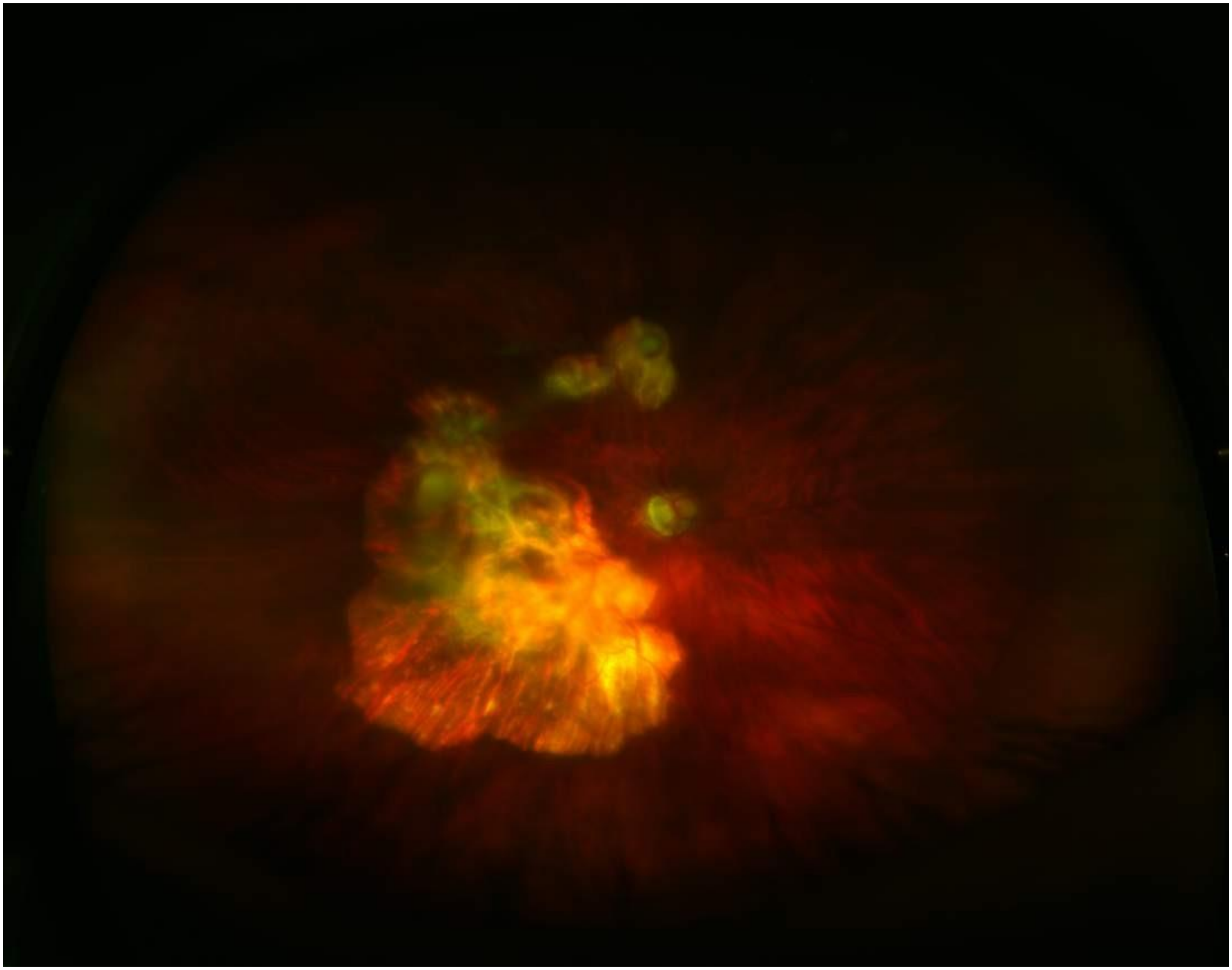

Patient AB

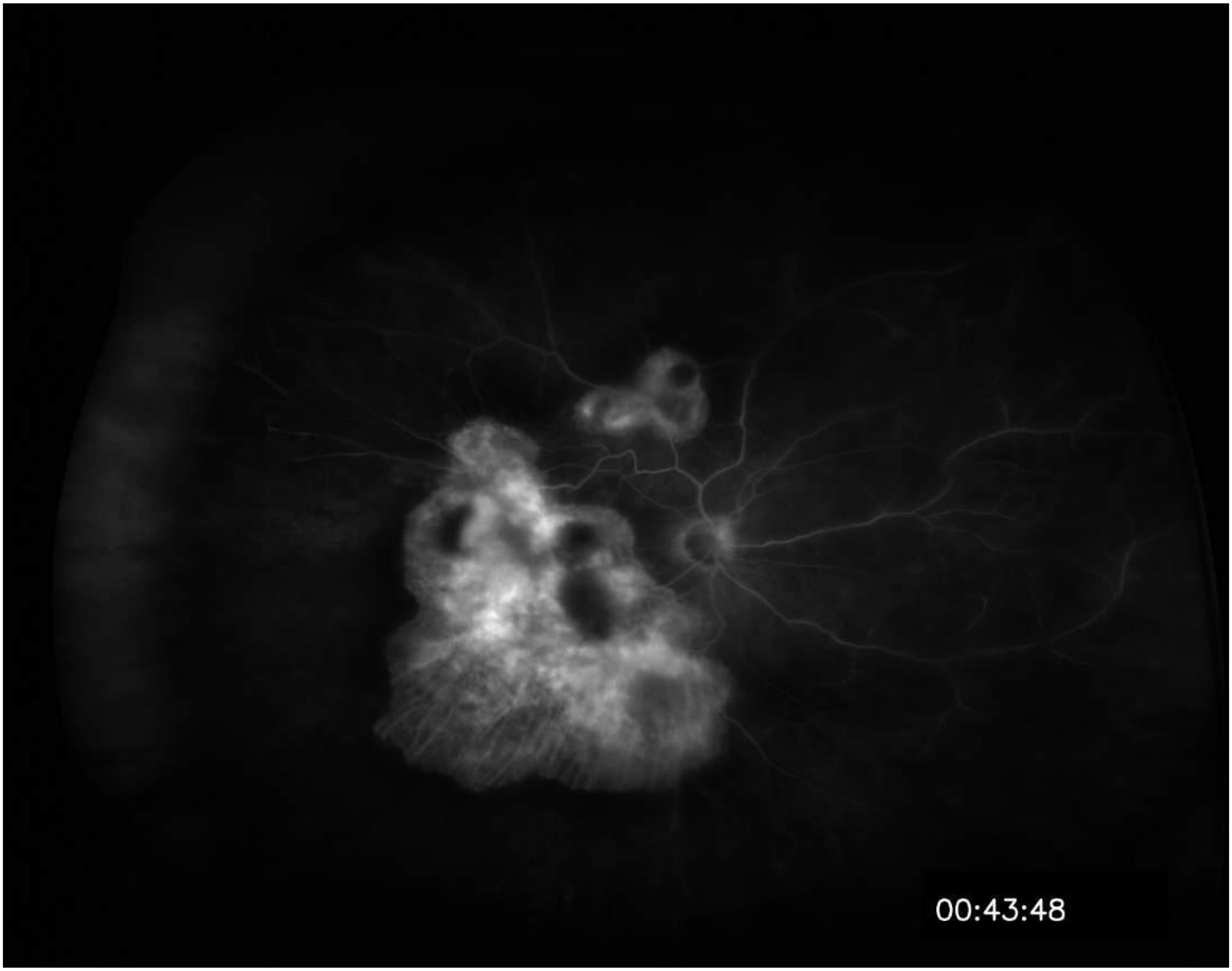
00:43:48
